# Occurrence, Evolution and Specificities of Iron-Sulfur Proteins and Maturation Factors in Chloroplasts from Algae

**DOI:** 10.3390/ijms22063175

**Published:** 2021-03-20

**Authors:** Jonathan Przybyla-Toscano, Jérémy Couturier, Claire Remacle, Nicolas Rouhier

**Affiliations:** 1 Université de Lorraine, INRAE, IAM, F-54000 Nancy, France; jonathan.przybyla-toscano@univ-lorraine.fr (J.P.-T.); jeremy.couturier@univ-lorraine.fr (J.C.); 2 Genetics and Physiology of Microalgae, InBios/Phytosystems Research Unit, University of Liège, 4000 Liège, Belgium

**Keywords:** maturation, iron-sulfur proteins, chloroplasts, microalgae, fermentation

## Abstract

Iron-containing proteins, including iron-sulfur (Fe-S) proteins, are essential for numerous electron transfer and metabolic reactions. They are present in most subcellular compartments. In plastids, in addition to sustaining the linear and cyclic photosynthetic electron transfer chains, Fe-S proteins participate in carbon, nitrogen, and sulfur assimilation, tetrapyrrole and isoprenoid metabolism, and lipoic acid and thiamine synthesis. The synthesis of Fe-S clusters, their trafficking, and their insertion into chloroplastic proteins necessitate the so-called sulfur mobilization (SUF) protein machinery. In the first part, we describe the molecular mechanisms that allow Fe-S cluster synthesis and insertion into acceptor proteins by the SUF machinery and analyze the occurrence of the SUF components in microalgae, focusing in particular on the green alga *Chlamydomonas reinhardtii*. In the second part, we describe chloroplastic Fe-S protein-dependent pathways that are specific to Chlamydomonas or for which Chlamydomonas presents specificities compared to terrestrial plants, putting notable emphasis on the contribution of Fe-S proteins to chlorophyll synthesis in the dark and to the fermentative metabolism. The occurrence and evolutionary conservation of these enzymes and pathways have been analyzed in all supergroups of microalgae performing oxygenic photosynthesis.

## 1. Introduction

Iron (Fe) is a highly abundant metal on earth, and was present at the origin of life in ferrous form (Fe^2+^) associated with sulfur in pyrite (FeS_2_), as well as contributing to electron transfer reactions. In the oxygenic atmosphere encountered nowadays, Fe is more accumulated in the poorly bioavailable oxidized Fe^3+^ form. However, Fe is essential in modern organisms and is necessary in substantial amounts, due to its presence in protein cofactors, i.e., iron-sulfur (Fe-S) clusters, hemes, and non-heme non-Fe-S mono- or di-iron cofactors [1]. Current estimates indicate that 2 to 10% of the proteomes of archaea, bacteria and eukarya are Fe-dependent proteins, representing, for instance, more than 1000 proteins in *Arabidopsis thaliana* (Arabidopsis) (approximately 4% of the proteome) [1]. Around 200 putative Fe-S proteins have been counted in Arabidopsis, the vast majority containing the most frequent rhombic (2Fe-2S) and cubane (4Fe-4S) cluster types as compared to the intermediate (3Fe-4S) cluster forms. More complex and atypical Fe-S clusters are observed in nature, mainly in proteins (e.g., nitrogenases, hydrogenases (HYDs), hybrid cluster proteins (HCPs)) present in prokaryotes and in some primitive eukaryotes including microalgae [2]. All these diverse Fe-S cluster forms confer to the associated proteins a wide range of functions, such as electron, sulfur or iron atom donors, intracellular oxygen or Fe concentration sensors, or else as Lewis acid catalysts, to cite a few examples [3]. As for mitochondria [4], the chloroplast has a very high demand in Fe-S cofactors, notably in the photosynthetic electron transport chain [5]. They are present in complexes involved in the linear electron transfer chain, including one (2Fe-2S) cluster in the Rieske subunit of the cytochrome b_6_f, three (4Fe-4S) clusters in photosystem I (PSI) subunits, and one (2Fe-2S) cluster in the final electron acceptor ferredoxin (FDX) [6]. They are also required for the cyclic electron transfer chain, since (4Fe-4S) clusters are bound by the plastid-encoded NDH-K and NDH-I subunits of the chloroplastic NADH dehydrogenase (NDH) complex, and an Fe-S cluster of unknown nature is also likely bound to PGR5-like photosynthetic phenotype 1 (PGRL1), an actor of the second pathway supporting cyclic electron transport [7,8,9]. In addition, stromal or thylakoid-bound Fe-S proteins participate in carbon, nitrogen, and sulfur assimilation, tetrapyrrole and isoprenoid metabolism, and ensure the biosynthesis of cofactors (i.e., lipoic acid and vitamin B1/thiamine) [5]. The de novo Fe-S cluster assembly, trafficking and insertion into chloroplastic proteins necessitate a protein machinery formed by ca. 15 proteins in terrestrial plants, namely the sulfur mobilization (SUF) machinery [5,10]. This machinery is also present in archaea and some bacteria, including *Escherichia coli* [11,12]. In the green lineage, previous studies of the SUF machinery have essentially been performed using cyanobacteria and Arabidopsis and to a much lesser extent with *Chlamydomonas reinhardtii* (Chlamydomonas) [5,13,14]. Hence, in the second section, after describing the current model for the molecular mechanisms used by the SUF machinery that sustain Fe-S cluster synthesis and insertion into acceptor proteins, we analyze the occurrence of the SUF components in Chlamydomonas, but also in the supergroups of microalgae. In the third section, we focused on the chloroplastic Fe-S protein-dependent pathways that are specific to Chlamydomonas, or for which Chlamydomonas presents specificities compared to terrestrial plants.

## 2. Maturation of Iron-Sulfur Proteins by the SUF Machinery

### 2.1. Lessons from Escherichia coli and Arabidopsis thaliana

**Figure 1:**
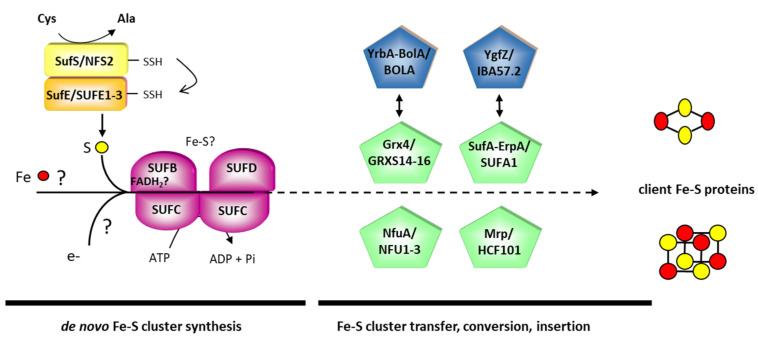
Working model of the SUF system. Both the *E. coli* and *A. thaliana* protein nomenclatures are indicated in this order unless they are similar. The sulfur (S) delivery system is shown in yellow/orange, the iron-sulfur (Fe-S) cluster scaffold complex in violet, the Fe-S cluster transfer proteins in green and associated targeting factors in blue. So far, it is believed that NFU and HCF101 are mostly responsible for the maturation of (4Fe-4S)-containing client proteins, and GRX and SUFA for the maturation of (2Fe-2S)-containing client proteins. Other details are found in the text and are not repeated here for the sake of concision.

From its presence in archaeal genomes and prevalence in (facultative) anaerobe bacteria, the SUF system is considered to be the most ancient Fe-S cluster biogenesis pathway, present even in early anaerobic forms of life [12,15]. Life in aerobiosis apparently led to its complexification, notably adding the *SufA*/*D*/*E*/*S* genes to the “ancestral” *SufB*/*C* genes as it now exists in the *E. coli* SUF operon (SufABCDSE) [12]. In this model bacterium, the SUF machinery operates under stress and Fe starvation conditions when the housekeeping ISC (iron-sulfur cluster) machinery is no longer operative [11,16]. However, in some other bacteria, the SUF system is the sole or major Fe-S biogenesis system, with some genes such as *SufD* sometimes being absent, or new ones such as *SufU* or *SufT* having appeared [15]. During evolution, photosynthetic eukaryotes have inherited the SUF system from cyanobacteria for the maturation of chloroplastic/plastidial Fe-S proteins, but innovations have appeared throughout evolution, notably in the green lineage. The SUF components have mostly been studied in *E. coli* and Arabidopsis by combining genetic analyses with biochemical and structural approaches. This led to the proposition of some unifying mechanistic concepts for Fe-S protein biogenesis by the SUF machinery [5,11,17]. Schematically, this process can be divided into two chronological steps involving (i) de novo Fe-S cluster synthesis on scaffold proteins and (ii) the conversion, trafficking, and insertion into acceptor proteins using Fe-S cluster transfer proteins (Figure 1).

For de novo Fe-S cluster synthesis, a class II pyridoxal 5′-phosphate (PLP)-dependent cysteine desulfurase (CD), SufS and CsdA in *E. coli*, or NFS2 in *A. thaliana* catalyzes the desulfuration of cysteine, leading to the formation of a persulfide group on a catalytic cysteine [17]. The class II CDs usually require the SufE/CsdE or SUFE sulfur shuttle proteins to enhance their activity [18,19,20]. In some organisms such as the Gram^+^ bacterium *Bacillus subtilis*, this role is played by SufU [21]. SufE proteins also possess an ultra-conserved cysteine residue, which receives the persulfide from SufS/NFS2. The interaction between these SufS and SufE leads to a slight conformational change that facilitates the desulfuration reaction, thus explaining the enhancement of the cysteine desulfurase activity [22]. Using *E. coli* proteins, it has been shown that the persulfide intermediate is then transferred to the SufB present in a SufBC_2_D scaffold complex (1:2:1 stoichiometry), in which the ABC-type ATPase SufC catalyzes ATP hydrolysis, and SufB and D subunits are thought to bind the Fe-S cluster, even though both the type of Fe-S cluster and its ligands are as yet unknown [23,24,25]. In vitro assays have shown that a (2Fe-2S) or a (4Fe-4S) cluster can be assembled in SufB alone or in complex with SufC and SufD [23,26,27]. From SufS to SufBC_2_D, sulfur atom(s) are carried as persulfides (0 oxidation state). However, in Fe-S clusters, they are present as sulfide ions (−2 oxidation state). Thereby, electrons are required to reduce sulfane sulfur (S^0^) atoms. It may also be that electrons are required for the reduction of Fe^3+^ to Fe^2+^ or for the reductive coupling of two (2Fe-2S) clusters into one (4Fe-4S) cluster on SufBCD if the binding of both cluster types turns out to be physiological. The origin of these electrons is unknown, but it has been proposed that FADH_2_, which was associated with the *E. coli* SufBC_2_D complex when purified under anaerobic conditions, and/or the NADPH-dependent thioredoxin system may have a role at this step [26,28,29]. In the mitochondrial assembly complex, frataxin has been shown to stimulate persulfide transfer from the NFS1 cysteine desulfurase to the ISCU scaffold, as shown for mammalian proteins [30,31]. From the observation that frataxin is able to interact with the *Bacillus subtilis* SufU-SufS couple [32] and may be present in chloroplasts [33], an involvement in the chloroplastic SUF machinery is suggested. However, in the absence of experimental evidence to firmly associate frataxin with the SUF assembly system, and considering that the composition and structures of the ISC and SUF assembly systems are completely different, such a role so far remains highly uncertain. Finally, how Fe is delivered to the assembly complex and how Fe entry is controlled and coordinated is as yet completely unclear.

Once the newly formed (2Fe-2S) or (4Fe-4S) cluster is assembled on the SUF scaffold system, it has to be delivered to specific acceptor proteins (Figure 1). This step is obviously crucial for generating functional chloroplastic Fe-S proteins and requires Fe-S cluster transfer proteins belonging to the A-type carrier (ATC), IBA57, glutaredoxin (GRX), BOLA, NFU and multiple resistance and pH adaptation (MRP) families. In *E. coli* or *A. thaliana*, the proteins are respectively referred to as SufA-ErpA/SUFA1, YgfZ/IBA57.2, Grx4/GRXS14-16, BolA-YrbA/BOLA1-4, NfuA/NFU1-3 and Mrp/high chlorophyll fluorescence 101 (HCF101). Unlike GRX, SUFA, NFU and HCF101 proteins, the BOLA and IBA57 proteins do not bind any Fe-S clusters themselves. For this reason, BOLA and IBA57 proteins are sometimes referred to as targeting factors and are rather hypothesized to mediate the interaction with or to recruit client Fe-S proteins. However, GRX and BOLA form Fe-S cluster-bridged heterodimers, as notably shown for the Arabidopsis GRXS14-BOLA1 couple [34]. Still, the precise role and hierarchical organization of all these late-acting factors as well as the molecular details involved at this step are far from being understood. The most intensive studies concerning the potential roles and targets of these proteins have been performed for the cases of *E. coli* SufA and NfuA [35,36] and of *A. thaliana* NFUs. In Arabidopsis, with the exception of the essential HCF101 protein [37], the other proteins characterized so far, including NFU1-3, are dispensable [38,39,40]. While the viability of Arabidopsis mutants has helped to study the role of most late-acting factors, this suggests a certain functional redundancy between these protein families and/or the coexistence of parallel pathways that are difficult to characterize. Using Arabidopsis mutant analyses coupled to proteomic, protein–protein interaction and in vitro Fe-S cluster transfer studies, the current model for NFU proteins is that NFU1 is dispensable for growth under standard conditions, possibly acting as a back-up system for NFU2 and NFU3 in specific conditions or for the maturation of some specific client proteins. From the lethality of a double *nfu2 nfu3* mutant, it seems that NFU2 and NFU3 act redundantly for some essential proteins, alimenting in particular HCF101 for the maturation of the PSI subunits, PsaA-C, which require (4Fe-4S) clusters [40,41,42]. According to the capacity of the recombinant protein to bind either a (2Fe-2S) cluster or a (4Fe-4S) cluster [43], NFU2 additionally serves for the maturation of a dihydroxyacid dehydratase, a (2Fe-2S)-containing enzyme involved in the synthesis of branched chain amino acids [40,44]. The precise roles of other Fe-S cluster transfer proteins remain to be delineated in the future, even though GRXS14 and SUFA1 were shown to be competent Fe-S cluster donors for FDXs in vitro [38,45,46] and GRXS14 shown to transfer an Fe-S cluster to SUFA1 [47].

### 2.2. Occurrence of the SUF Components in Microalgae

Microalgae are a phylogenetically diverse group of eukaryotic photosynthetic microorganisms that thrive in various environments. Notably, the phylogenetic classification is still not well established, and it may differ slightly depending on the source. It is accepted that microalgae are split into several supergroups of the tree of life [48,49], which are not exclusively photosynthetic except for the Archaeplastids. The Archaeplastids are defined by the presence of a primary plastid derived from a cyanobacterium, and comprise, in particular, the Glaucophytes, the red algae (Rhodophytes), the green algae (Chlorophytes) and the Streptophytes, where the land plants and their closest algal relatives are found. The other microalgae possess secondary plastids derived from green or red algae and are spread among other supergroups. The Heterokonts include the well-known diatoms such as *Phaeodactylum tricornutum.* The Alveolates comprise the genus *Symbiodinium*, in which many species form symbiotic association with corals and the Chromerida phylum including *Vitrella brassicaformis* and *Chromera velia*, two photosynthetic relatives of Apicomplexes [50]. The Rhizaria comprise amoeboid protists of which some are photosynthetic (*Bigelowiella natans*). The Haptophytes comprise the marine species *Emiliania huxleyi*, which can grow at high density and can be responsible for algal blooms. The Cryptophytes comprise the marine species *Guillardia theta*. The Excavates contain photosynthetic Euglenids such as the freshwater microalga *Euglena gracilis*.

A previous genomic analysis identified known SUF components in *C. reinhardtii* [51]. However, new factors are now proposed to be part of the SUF machinery in eukaryote photosynthetic organisms and other microalgal genomes were not investigated. The recent Phycocosm sequencing project offers a unique opportunity to compare how the SUF system evolved in microalgae with very diverse origins and to determine whether adaptations occurred during evolution [49]. Using Chlamydomonas SUF proteins as references (Table 1), we performed a comparative genomic analysis of 25 organisms representing microalga diversity (Table 2).

Chlamydomonas seems to have an expanded CD family with five putative sequences (CSD1, 2, 4, 5 and ABA3), where Arabidopsis has, for instance three members (NFS1, NFS2 and ABA3). Two isoforms are likely present in chloroplasts. The so-called Chlamydomonas SUFS1/CSD4 was detected in a chloroplast proteomic study and is in fact the ortholog of Arabidopsis NFS2 and *E. coli* SufS [52]. With few exceptions, it is present in all algal genomes analyzed (Table 2), but also in cyanobacteria. The second CD putatively expressed in chloroplasts based on subcellular localization predictions and on the presence of a characteristic N-terminal extension is referred to as NIFS2/CSD2. In this case, we found no supportive proteomic evidence. Similar sequences possessing an N-terminal extension are present in numerous algal groups, but not all (Table 2). Noticeably, this isoform is present in organisms apparently lacking SUFS1. Orthologs are present in many bacteria, but not in terrestrial plants (bryophytes, lycophytes, angiosperms), suggesting that the gene was lost after the divergence between charophytes and terrestrial plants. In current models, class II CDs are associated with a sulfur relay protein, either SufU or SufE. Only SUFE proteins are present in eukaryote photosynthetic organisms. Dicotyledonous plants, such as *A. thaliana*, typically contain three proteins with SufE domains [10], while a single SUFE protein is present in cyanobacteria. From the amino acid sequence and phylogenetic analyses, the *A. thaliana* SUFE1 likely corresponds to the ancestral SUFE protein found in cyanobacteria, to which a BolA domain has been added in the C-terminal part. All microalgae analyzed possess the SUFE1 prototype. However, the BolA domain is sometimes absent in microalgal sequences whereas it is systematically present in proteins from terrestrial plants. The evolutionary scenario is difficult to retrace but this suggests that the BolA domain is dispensable in some organisms but may have become indispensable in terrestrial plants for the SUFE1 function because there is no example of SUFE1 proteins devoid of BolA domain in these organisms. The SUFE2 protein is formed by a single SufE domain. This gene likely appeared after the divergence between charophytes and terrestrial plants since it is absent in all microalgae. In fact, it may have specialized functions in terrestrial plants, since it is specifically expressed in reproductive organs [53]. The SUFE3 prototype is formed by a SufE domain fused to a quinolinate synthetase domain at the C-terminus. It was suggested that the SufE domain is dedicated to the formation of the [4Fe-4S] cluster bound by the NadA domain [53]. If we except its presence in a few heterokonts, the SUFE3 protein is only present in the green lineage, e.g., Chlorophytes, Streptophytes and Tracheophytes. It would be interesting to determine whether both SUFE1 and SUFE3 interact with both class II CDs. Concerning the scaffold SUFBCD complex, all microalgae possess genes coding for these three proteins except in three organisms in which one gene is apparently missing (Table 2). However, from this high and widespread conservation, we suspect this could in fact be due to a mis-annotation, even though we have not yet been able to identify the corresponding coding sequences in the genomic sequences.

More noticeable differences are observed among putative Fe-S cluster transfer proteins. First, there is a set of four genes/proteins, SUFA1, GRX3, NFU2 and HCF101, that is present in almost all organisms (Table 2). We could not identify HCF101 in one species only (*Picocystis* sp. ML), which again raises questions about the validity of this observation. GRX6/GRXS16 orthologs are less evenly distributed, almost never being present outside the green lineage (Chlorophytes and Streptophytes). Concerning the two putative chloroplastic BOLA proteins, here renamed BOL1 and BOL3 (to follow the new nomenclature rules adopted for *C. reinhardtii*), the analyzed organisms possess at least one isoform except in Heterokonts and Alveolates. The absence of BOLA proteins in these organisms is surprising considering the strong co-occurrence existing with GRX [54]. In mitochondria from eukaryotes, the role of IBA57 is closely linked to the ATC proteins (ISCA1/2). Land plants also have this gene referred to as *IBA57.1*, but most of them also have a second *IBA57* gene (with the notable exception of monocots) coding for a chloroplastic isoform according to the presence of an ortholog in cyanobacteria [55]. Although a physiological connection between the chloroplastic SUFA1 and IBA57.2 counterparts has not been documented yet, it is surprising that the phylogenetic distribution of IBA57.2 does not follow the one of SUFA1, being likely absent in several species. The hypothesis of a dual targeting to chloroplasts and mitochondria made for the orthologs in monocot species (but not verified yet) may also be true for some algal orthologs.

Finally, the most striking difference between terrestrial plants and algae is for chloroplastic NFU proteins. As already stated, there is only one NFU2/3 representative in *C. reinhardtii*, instead of the two in Arabidopsis and many other terrestrial plants (Table 2). More importantly, CrNFU1 has an atypical domain architecture formed by an N-terminal so-called GIY-YIG domain, which is the catalytic domain present in some endonucleases, fused to the usual NFU domain possessing the cysteines serving for Fe-S cluster binding. Hence, it lacks the second degenerated NFU domain normally present in chloroplastic NFUs. Proteins with a similar architecture are present in many of the microalgae analyzed, but not all, and with no clear pattern of distribution. Interestingly, orthologs are present in all terrestrial plants (including bryophytes and gymnosperms) except dicots. It is noticeable that a similar GIY-YIG domain of unknown function is also present in the N-terminal region of GRX6. In this case, the additional domain has been conserved in dicots as well.

## 3. Fe-S Protein-Dependent Metabolic Processes in Algal Chloroplasts

Known Fe-S proteins present in plastids of land plants and their associated functions have recently been reviewed [5], and Chlamydomonas orthologs will not be systematically described here. However, generally speaking, most important metabolic pathways occurring in chloroplasts are dependent on one or several Fe-S proteins, not to mention proteins dependent on the (2Fe-2S)-containing FDXs. It is worth noting that Chlamydomonas and other close Chlorophyceae (*Volvox carteri* and *Gonium pectorale*) possess 12 genes coding for chloroplastic FDXs [56,57], which far surpasses the content of *FDX* genes in land plants, with six genes being present, for instance, in Arabidopsis [5]. We direct the reader to the following recent review to learn more about the known functions of some Chlamydomonas FDXs [57]. In the following subsections, we will focus rather on Fe-S protein-dependent pathways that are specific to Chlamydomonas or for which Chlamydomonas presents specificities compared to terrestrial plants.

### 3.1. Fe-S Proteins Connected to the Photosynthetic Electron Transfer Reactions

#### 3.1.1. Synthesis of Isoprenoids: Fe-S Dependent vs. Independent Pathways

The ability to synthesize isopentenyl diphosphate, the 5-carbon structure necessary for isoprenoid formation, is essential in all organisms, because isoprenoid-derived molecules include sterols, dolichol, and coenzyme Q, as well as plastoquinones, carotenoids and chlorophylls, which are particularly relevant to the topic of photosynthesis. Isoprenoid synthesis relies on two pathways, the mevalonate (MVA) pathway, which does not involve Fe-S proteins, and the 2-C-methyl-d-erythritol 4-phosphate (MEP) pathway, which requires two proteins with (4Fe-4S) clusters: (E)-4-hydroxy-3-methylbut-2-enyl diphosphate synthase (HDS, ISPG) and 4-hydroxy-3-methylbut-2-en-1-yl diphosphate reductase (HDR, ISPH). The MVA pathway is present in animals, fungi and archaea, the MEP pathway is present in nearly all bacteria [58]. Plants have usually retained both pathways. Most of the plant-specific isoprenoids, such as phytols and carotenoids, are synthesized in plastids by the MEP pathway inherited from the cyanobacterium endosymbiont [58,59,60], while the cytosolic MVA pathway contributes to sterol synthesis [61]. The situation is variable in microalgae. In Archaeplastids, Charophytes possess both pathways but Chlorophytes and some Rhodophytes rely only on the MEP pathway for isoprenoid synthesis, although the HMG-CoA synthase, the second enzyme of the MVA pathway, is present in some of them [58,60]. For algae with secondary endosymbiosis, diatoms and *Euglena gracilis* kept both pathways [61,62], while only the MEP pathway is present in *Nannochloropis oceanica*, although a HMG-CoA synthase is also present in this alga [63]. Finally, dinoflagellates only use the MEP pathway [64]. In conclusion, for isoprenoid synthesis, microalgae use either the two pathways like in plants, or only the cyanobacterial Fe-S cluster-dependent type. The reason for the loss of the MVA pathway or the presence of both pathways is presently unknown.

#### 3.1.2. Dark-Operative Protochlorophyllide *a* Oxidoreductase

The reduction of protochlorophyllide into chlorophyllide *a* is the penultimate step of chlorophyll *a* biosynthesis. This step is catalyzed by two enzymes, the light-dependent protochlorophyllide *a* oxidoreductase (LPOR) and the dark-operative protochlorophyllide *a* oxidoreductase (DPOR). LPOR is encoded by one or several nuclear genes (*POR* genes) and uses NADPH as an electron donor in the presence of light for the reduction of the C17-C18 double bond (reviewed in [65]). The holocomplex is proposed to be dimeric [66]. The DPOR protein is a complex formed by the L, N and B subunits encoded in the chloroplast genome by the *chlL*, *chlN* and *chlB* genes. A crystal structure of the 360 kDa DPOR complex from the cyanobacterium *Prochlorococcus marinus* indicates that it is a hetero-octameric complex (L_2_)_2_(NB)_2_ containing four (4Fe-4S) clusters [67]. The L_2_ domain is the ATP-dependent reductase component for the catalytic NB domain, where protochlorophyllide reduction takes place using reduced FDX as an electron donor [67]. DPOR is believed to have evolved from a nitrogenase-like enzyme of anoxygenic photosynthetic bacteria, while LPOR arose in cyanobacteria in an oxygenic environment [68]. As a matter of fact, the *Klebsiella pneumoniae* nitrogenase Fe protein gene (*nifH*) functionally substitutes for the *chlL* gene in the green microalga *C. reinhardtii* [69] in which the absence of ChlL or B subunits leads to a “yellow in the dark” phenotype [70,71]. DPOR has been lost multiple times during evolution and is absent in angiosperms and some gymnosperms. In microalgae, its presence is variable among and within the different lineages, as exemplified in Table 3.

The *chlL*, *chlN* and *chlB* genes are not present in any of the chloroplast genomes sequenced in the Chlorarachniophytes, Haptophytes and Euglenophyceae, suggesting that these genes were lost early, while these lineages were settling [93]. Other groups present lineages and species with or without these genes (see Table 3, [90,93]). In Cryptophytes, some species even possess pseudogenes in the chloroplast genome, which suggests that the process of loss is still occurring in this group [90]. When DPOR is present, only one *POR* gene is usually present, while multiple *POR* genes are often present in species lacking DPOR [93]. Concerning the light spectrum, the protochlorophyllide pigment is absorbed in both the blue and the red regions, but its photoconversion by LPOR would be much more efficient when red light is applied. Since red light is rapidly attenuated in water, activity of the LPOR enzyme would be much less efficient in deep, turbid waters, while DPOR is insensitive to the light (discussed in [93]). This would explain why the presence of DPOR would be beneficial for microalgae living in deep, turbid waters, which is the case of freshwater species. Indeed, *chlL*, *chlN* and *chlB* genes are usually found in Chlorophytes such as in the freshwater *C. reinhardtii* (Table 3) [75]. One exception has, however, recently been described in the Chlorophycean class: a psychrophilic Chlamydomonas strain (UWO241) in which the absence of DPOR could be linked to the high oxygen concentration in the water layer of Lake Bonney in Antarctica, where this isolate lives, considering the sensitivity of the Fe-S clusters of DPOR to oxygen [94]. In addition to its proposed sensitivity towards O_2_, DPOR would also be counter-selected in oceans where Fe is limiting. As a matter of fact, diatoms such as *P. tricornutum* and *Thalassiosira pseudonana* do not have DPOR, but other oceanic species, such as *N. oceanica*, do.

#### 3.1.3. Type I NADH Dehydrogenase in Microalgae

The type I NADH dehydrogenase (NDH-1) is a minor component of the photosynthetic chain in land plants, inherited from cyanobacteria. In plants, it comprises 11 subunits encoded in the chloroplast genome (NdhA-K) with NdhI and NdhK binding (4Fe-4S) clusters and more than 19 subunits encoded in the nucleus [95]. Although pioneer works proposed that plastidial NDH-1 would oxidize NADH or NADPH, more recent studies have suggested that the enzyme would accept electrons from FDX [96]. The detailed analysis of its proposed function in cyanobacteria and in land plants is reviewed in [95], and the crystal structure of the *Thermosynechococcus elongatus* NDH-I was recently published, confirming that the enzyme oxidizes FDX instead of NAD(P)H [7,96]. Most of the microalgae did not retain this multimeric complex in their photosynthetic chain. The *Ndh* genes are still present in the chloroplast genome of some Chlorophytes [76,80] and in Charophytes, the closest relatives to land plants (Table 3) (reviewed in [97]). In the green microalga Chlamydomonas, there is no gene coding for NDH subunits in the chloroplast genome, but a nuclear-encoded monomeric type II NADH dehydrogenase (NDH-2) (Nda2) exists in chloroplast. This enzyme is involved in the non-photochemical reduction of the plastoquinone (PQ) pool by using NADH or NADPH as electron donor, and one FMN as cofactor [98,99]. This enzyme would participate in chlororespiration, together with the plastid terminal oxidase 2 (PTOX2), to reduce O_2_ into H_2_O. Chlororespiration would be involved in the dissipation of the excess of reducing equivalents originating from both light-induced processes and carbon metabolism when electron flow downstream of the PQ pool is compromised (reviewed in [100]). CrNDA2 would also be involved in H_2_ photoproduction [98,101].

#### 3.1.4. Photosystem II Protein 33

Photosystem II protein 33 (PSB33) is part of the Greencut, i.e., the set of proteins specific to organisms of the green lineage [102]. It is a transmembrane protein associated with thylakoid membranes, which is involved in the fine-tuning of light harvesting and/or energy transfer around both photosystems in *A. thaliana* [103,104]. Some green algae possess a PSB33 ortholog including Chlamydomonas (Cre09.g411200, TEF5), but also a closely related protein (PSB33-like, Cre02.g093650) assumed to be soluble because it lacks the transmembrane domain. There is no ortholog of this PSB33-like protein in land plants. It is predicted to bind a (2Fe-2S) Rieske-type cluster owing to the presence of CxH and CxxH motifs that are conserved in cyanobacterial orthologs. Although the transcript abundance of this PSB33-like protein is increased under low-Fe conditions [105], whether this protein indeed binds an Fe-S cluster and whether this is important for its function, which is yet unknown, remains to be explored. Finally, it is highly uncertain that transmembrane PSB33 proteins have the capacity to bind an Fe-S cluster since only the cysteine of the second motif is conserved.

#### 3.1.5. Chloroplast Sensor Kinase

The chloroplast sensor kinase (CSK) is a histidine kinase that is proposed to regulate photosystem gene expression in the chloroplast. The recent biochemical characterization of recombinant *A. thaliana*, *P. tricornutum* and *Synechocystis* sp. PCC 6803 CSK showed that it binds a [3Fe-4S] cluster that would be sensitive to the redox state of the PQ pool [106]. It is proposed that upon specific light excitation of PSII, the [3Fe-4S] cluster is reduced by PQH_2_, which would inactivate the kinase. When the PQ pool is oxidized by specific excitation of PSI, the [3Fe-4S] cluster of CSK would be reoxidized by oxygen, and CSK would be activated to regulate chloroplast gene expression. The sequence conservation is so weak among these proteins that it hampered to determine whether orthologs are present in other algae.

### 3.2. Contribution of Fe-S Enzymes to the Fermentative Metabolism

**Figure 2:**
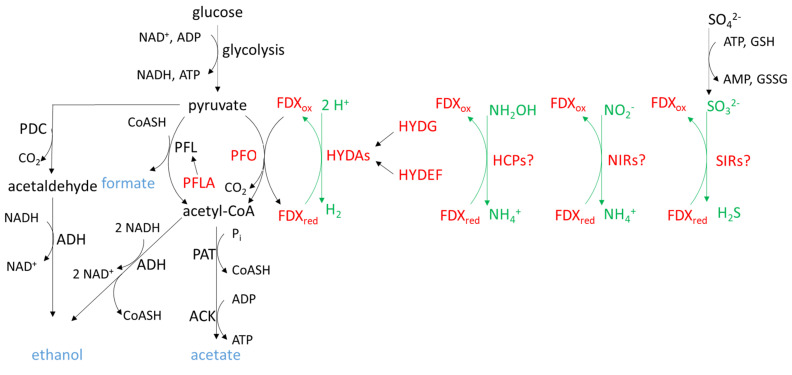
Major fermentation pathways *in C. reinhardtii*. ACK: acetate kinase; ADH: alcohol/aldehyde dehydrogenase; FDX: ferredoxin; GSH: reduced glutathione; GSSG: glutathione disulfide; HYDAs: hydrogenases; HYDEF and HYDG: maturases of hydrogenases; HCPs: hybrid cluster proteins; NIRs: nitrite reductases; PAT: phosphoacetyltransferase; PDC: pyruvate decarboxylase; PFL: pyruvate-formate lyase; PFLA: pyruvate-formate lyase activating enzyme; PFO: pyruvate-ferredoxin oxidoreductase; SIRs: sulfite reductases. Fe-S enzymes are in red. The four putative pathways serving for ferredoxin oxidation are in green. Adapted from [108,111].

Microalgae encounter periods of hypoxic/anoxic phases when O_2_ availability becomes limited. This happens in the dark (i.e., notably during the night), when O_2_ produced by photosynthetic activity decreases because of respiratory activity, or in the light, when heterotrophic microbial communities exceed those that are photosynthetic. In addition, some species of the Chlamydomonas genus even constantly live in anoxic biotopes such as in peat bogs and sewage lagoons [107], which may explain why this microalga has a particularly well-developed fermentative metabolism, essentially taking place in the chloroplast. Basically, glucose generated by starch hydrolysis is oxidized into pyruvate, leading to the generation of ATP and NADH. Under anaerobiosis, in the absence of a functional tricarboxylic acid cycle, NADH must be oxidized by fermentation processes to maintain the glycolytic flux, energy production and survival in the dark. The fermentative metabolism under dark anaerobic conditions has been quite well described in *C. reinhardtii*, where the various pathways (Figure 2) lead to the excretion in the medium of formate, acetate, and ethanol as major organic products, while CO_2_ and H_2_ are generated as minor gaseous products (extensively reviewed in [108,109,110]). Chlamydomonas has two enzymes performing the conversion of pyruvate into acetyl-CoA under anaerobiosis: pyruvate-formate lyase (PFL) and pyruvate-ferredoxin oxidoreductase (PFO) (Figure 2). PFL catalyzes the conversion of pyruvate into acetyl-CoA and formate, while PFO catalyzes the oxidation of pyruvate into acetyl-CoA and CO_2_, with simultaneous reduction of FDX. Reduced FDX can be reoxidized by hydrogenases (HYDs), leading to the formation of dihydrogen (H_2_). It has also been proposed that sulfite reductases (SIRs), nitrite reductases (NIRs), and hybrid cluster proteins (HCPs) [111] could also participate in the reoxidation of FDX under anaerobiosis. The acetyl-CoA produced by PFL and PFO is either reduced to ethanol by alcohol/aldehyde dehydrogenase (ADH) or converted into acetate by the phosphoacetyltransferase-acetate kinase (PAT-ACK) pathway [108]. The PAT-ACK pathway is present in both chloroplast and mitochondria. PAT and ACK are encoded by two distinct genes for the two organelles (PAT1, ACK2 in mitochondria; PAT2-ACK1 in chloroplast), while PFL is encoded by a single nuclear gene (*PFL1*), but is dually targeted to both mitochondria and chloroplast [52,112]. This route provides ATP, but does not eliminate reductants. Another route for ethanol production consists of the decarboxylation of pyruvate by pyruvate decarboxylase (PDC) into CO_2_ and acetaldehyde, which is then reduced into ethanol by ADH. Enzymes bearing Fe-S clusters are shown in red in Figure 2 and are described in the following subsections.

#### 3.2.1. Pyruvate-Formate Lyase Activating Enzyme

PFL is converted post-translationally from an inactive to an active form by a ~20 kDa protein, namely the pyruvate-formate lyase activating enzyme (PFLA). This radical S-adenosyl-L-methionine (SAM) enzyme introduces a radical (H atom abstraction) on the ultimate glycine residue of the PFL [113,114]. Molecular oxygen irreversibly inactivates the radical active form of PFL [115]. Like most radical SAM enzymes, PFLA possesses the usual CX_3_CX_2_C motif by which the conserved cysteines coordinate three Fe atoms of the (4Fe-4S) cluster while SAM coordinates the fourth Fe atom [114]. PFLA is present in an inactive form under aerobic conditions and is allosterically activated by pyruvate [116]. The Chlamydomonas PFLA is predicted to be targeted to the chloroplast, according to the presence of a characteristic N-terminal extension [112].

#### 3.2.2. Pyruvate-Ferredoxin Oxidoreductase

Chlamydomonas PFO is a large enzyme of 128 kDa binding three (4Fe-4S) clusters into a homodimer [117,118]. In addition to the cysteine-binding motifs, there is a YPITP motif at the N-terminal part of the protein [119] that would be involved in the turnover of the reaction and a motif for binding a thiamine pyrophosphate cofactor, essential for the decarboxylation reaction, at the C-terminal part. Because the synthesis of thiamine relies on an Fe-S protein, the synthesis of an active holo-PFO enzyme is doubly dependent on a functional SUF maturation system.

#### 3.2.3. Hydrogenases and Their Maturation Factors

In Chlamydomonas, two genes, *HYDA1* and *HYDA2*, encode a [Fe-Fe] hydrogenase. HYDA1 and HYDA2 amino acid sequences are very close to each other (73% identity), and HYDA1 is the major contributor of H_2_ production (75%) [120]. The catalytic site of the HYDA1 enzyme contains a complex Fe-S cluster named the H-cluster that is composed of a classical (4Fe-4S) cluster bridged by a cysteine thiolate to a unique 2Fe sub-cluster in which two Fe atoms are coordinated by three CO- and two CN- ligands, as well as an azadithiolate bridge [121,122,123,124]. It is suggested that the (4Fe-4S) cluster is inserted by the SUF assembly machinery followed by the 2Fe sub-cluster, whose biosynthesis requires the specific HYDE, HYDF and HYDG maturases, with the HYDE and HYDG being two radical SAM proteins binding (4Fe-4S) clusters [125,126,127]. In Chlamydomonas, the HYDE and HYDF maturases are fused as a chimeric protein, whereas these are separate genes/enzymes in some microalgae [128,129]. HYDE and HYDG synthesize the non-protein ligands, and HYDF is an Fe-S cluster binding and GTPase-domain containing protein that provides the scaffold for the assembly of the 2Fe sub-cluster of the H domain before its insertion in the hydrogenase [124].

#### 3.2.4. Hybrid Cluster Protein

HCPs bear two different types of Fe-S clusters: a classical (4Fe-4S) cluster and a (4Fe-2O-2S) hybrid cluster. Crystal structures have been obtained in two *Desulfovibrio* species [130,131,132] in which the conserved cysteines for the binding of the (4Fe-4S) cluster are present in the N-terminal part while the ligands for the binding of the hybrid cluster are scattered elsewhere. In Chlamydomonas, four genes (*HCP1*-*HCP4*) encode HCP proteins. The phylogenetic analyses suggest that *HCP* genes arose from a single gene of alpha-proteobacterial origin followed by subsequent duplications. These duplications are very specific to Chlamydomonas, since most microalgae analyzed have a single gene, with some species, including *Volvox carteri*, having two genes. Chlamydomonas HCP1, 3 and 4 would be targeted to the chloroplast, while HCP2 would be localized in mitochondria [133]. Nitrate and darkness are responsible for increased levels of the proteins in oxic conditions [133]. The *HCP4* transcript level is increased in dark anoxia [134]. Characterization of a Chlamydomonas *hcp4* mutant obtained by miRNA suggests that the corresponding protein could be involved in FDX oxidation (Figure 2) as a putative hydroxylamine reductase, competing with hydrogenase for electrons from reduced FDX [111].

#### 3.2.5. Sulfite Reductase

In plants, SIR participates to sulfate assimilation and catalyzes the reduction of sulfite to sulfide using reduced FDX as electron donor. It contains a siroheme linked to a (4Fe-4S) cluster, and a series of crystal structures has revealed how FDX binds to the catalytic site of the plant enzyme (reviewed in [135]). While there is a single essential gene in Arabidopsis, three *SIR* genes (*SIR1*, *SIR2*, *SIR3*) are present in Chlamydomonas. SIR1 is predicted to be localized to the chloroplast by Target-P, while the other two proteins would not be localized in organelles. *SIR1* transcript level (Cre16.g693202) is increased in dark anaerobiosis conditions [134], suggesting that SIR1 could also compete with hydrogenase for reduced FDX.

#### 3.2.6. Fermentative Pathways in Other Microalgae

Other freshwater green microalgae also perform anaerobic fermentation of starch [108], but *C. reinhardtii* is by far the best characterized species. Table 4 presents the Fe-S enzymes of the anaerobic metabolism found among microalgae of diverse phylogenetic origin. Fe-S cluster enzymes participating in anaerobic metabolism seem absent in two classes (Rhodophytes and Haptophytes), while the other groups present at least one representative Fe-S enzyme. The complete set of enzymes participating in the anaerobic metabolism is described in [108]. Marine species such as diatoms and dinoflagellates survive in dark anoxic conditions when they sink in the oceans [136]. Diatoms can use nitrate for dissimilatory nitrate reduction to ammonium (DNRA) [137,138,139], which allows NAD(P)+ regeneration and maintains the glycolytic flux [140]. DNRA proceeds as follows: nitrate is accumulated inside the cells converted into nitrite by nitrate reductase. Nitrite can be reduced into ammonium by nitrite reductase (NIR) inside the chloroplast, under dark anoxic conditions. NIR is a siroheme protein bearing a (4Fe-4S) cluster using reduced FDX as electron donor and may thus also be considered as a competitor to hydrogenase for FDX if hydrogen is produced as a fermentative product by the microalga. This could be the case of the diatom *T. pseudonana*, where a gene encoding a hydrogenase has been found in the nuclear genome although the maturation factors could not be identified (Table 4) [108].

## 4. Conclusions

In summary, the SUF machinery, dedicated to the maturation of chloroplastic Fe-S proteins, is present in all supergroups of microalgae performing oxygenic photosynthesis. However, particularities exist compared to terrestrial plants, notably in the sulfur mobilization system with the presence of an additional class II cysteine desulfurase in some organisms, which is surprisingly accompanied by a reduced number and diversity of SUFE proteins. The scaffold SUFBCD proteins are almost ubiquitously present, with no apparent specificity. Some variations exist at the level of Fe-S cluster transfer proteins. While the SUFA1, NFU2, GRX3 and HCF101 proteins are ubiquitously present in microalgae analyzed, differences are visible for IBA57.2, NFU1, GRX6 and the BOLA family. Many algae outside the green lineage lack BOLA proteins and have a single GRX representative. Concerning Fe-S proteins, it appears that the living environment or ecological niche of some microalgae have a strong impact on some metabolic pathways. It has already been noticed that the progressive adaptation of the aquatic (and terrestrial) life at increasing O_2_ concentrations was accompanied by several changes in terms of the oxido-reduction metabolism, such as the appearance of Fe-S cluster-free, oxygen-resistant proteins (whereas “ancestor” proteins had Fe-S clusters) or the transition from a ferredoxin to a NADPH- and flavin-dependent redox metabolism [141]. Still, despite possessing an O_2_-dependent energy metabolism, some microalgae such as *C. reinhardtii*, but not all, have retained a developed anaerobic energy metabolism to cope with anoxic/hypoxic conditions that relies on O_2_-sensitive Fe-S cluster containing-enzymes (Fe-Fe hydrogenases, PFO). Variations among microalgae are also visible when considering chlorophyll biosynthesis. Within supergroups, some taxa have retained an Fe-S cluster-dependent dark-operative protochlorophyllide *a* oxidoreductase in addition to the NADPH-dependent light-dependent protochlorophyllide *a* oxidoreductase. While the three *chl* genes are typically either entirely present or absent from a chloroplast genome, there are remarkable examples of species having lost only one gene or possessing pseudogenes, suggesting that the process of *chl* gene loss is currently operating in these organisms [93]. Hence, analyzing the genomic data recently acquired on very diverse algal species [49] will undoubtedly give additional clues about the evolutionary adaptations (loss, acquisition, duplication) of some important metabolic pathways as exemplified in this review.

## Figures and Tables

**Table 1:** SUF components in *C. reinhardtii*. The sequences of SUF components present in *C. reinhardtii* were retrieved from the currently available genome version (v5.5) found in phytozome using a blast search performed with Arabidopsis sequences. A manual curation was necessary for some gene/protein sequences (GRX3, BOL1 and newly named NFU1). Based on genomic sequence analysis, a gene model was proposed for *SUFA1* (*Cre06g299350 in the coming v6 genome release), which has thus far been absent. Finally, a change in the NFU nomenclature was asked to fit with the names in Arabidopsis and the phylogenetic analysis performed in [40]. The current *NFU3* gene model (Cre17.g710800) will be renamed *NFU1*, whereas the current *NFU1* (Cre18.g748447) will be renamed *NFU2*. All these changes are already integrated here and will be visible in the next genome release.

Chlamydomonas Gene Names	Accession Numbers	Function(s)	Arabidopsis Orthologs
*CSD4* *, SUFS1*	Cre12.g525650	Cysteine desulfurase, sulfur donor	At1g08490 (*NFS2*)
*CSD2* *, NIFS2*	Cre07.g322000		None
*SUFE1*	Cre06.g309717	Sulfur relay system from cysteine desulfurase	At4g26500 (*SUFE1*)
			At1g67810 (*SUFE2*)
*SUFE3* */NIC7*	Cre06.g251450	Quinolinate synthetase A	At5g50210 (*SUFE3*)
*SUFB*	Cre15.g643600	Scaffold protein complex	At4g04770 (*SUFB*)
*SUFC*	Cre07.g339700	Scaffold protein complex	At3g10670 (*SUFC*)
*SUFD*	Cre12.g513950	Scaffold protein complex	At1g32500 (*SUFD*)
*GRX3*	Cre07.g325743	Transfer protein, involved in Fe-S cluster trafficking	At3g54900 (*GRXS14*)
*GRX6*	Cre01.g047800		At2g38270 (*GRXS16*)
*BOL1*	Cre03.g180700	Targeting factor, involved in Fe-S cluster trafficking	At1g55805 (*BOLA1*)
*BOL4*	Cre09.g394701		At5g17560 (*BOLA4*)
*SUFA1*	Cre06g299350*	Transfer protein, involved in Fe-S cluster trafficking	At1g10500 (*SUFA1*)
*IBA57.2* */CGL77*	Cre12.g552850	Targeting factor, involved in Fe-S cluster trafficking	At1g60990 (*IBA57.2*)
*NFU1*	Cre17.g710800	Transfer protein, involved in Fe-S cluster trafficking	At4g01940 (*NFU1*)
*NFU2*	Cre18.g748447		At5g49940 (*NFU2*)
			At4g25910 (*NFU3*)
*HCF101*	Cre01.g045902	Transfer protein, involved in Fe-S cluster trafficking	At3g24430 (*HCF101*)

**Table 2:** SUF components in representative microalgae. Sequences were retrieved in 25 species from Phycocosm (https://phycocosm.jgi.doe.gov/phycocosm/home (accessed on 10 March 2021)) using *C. reinhardtii* sequences and blastp or tblastn searches [10]. The sequences are available as supplementary information. a: The C-terminal BolA domain is missing; b: The C-terminal BolA domain is missing in the studied species but a full-length SUFE1 protein is present in some species of the group; c: the sequence is encoded by the chloroplast or nucleomorph (*B. natans*) genomes; d: the sequence lacks the hallmark KCGxxGQE signature of IBA57 proteins and it was impossible to determine whether this was due to an annotation problem or not; e: the sequence is apparently not present in the species analyzed but a GRXS16 ortholog is present in other red algae; f: the *NFU1* gene seems only present in Micromonas among the Mamiellophyceae available. Notably, we cannot exclude that sequences in some of the automatically annotated genomes analyzed are reported as missing because of problems in the genome assembly.

			Sulfur Supply				Scaffold System			Iron-Sulfur Cluster Transfer								
			NIFS2	SUFS1	SUFE1	SUFE3	SUFB	SUFC	SUFD	SUFA1	IBA57.2	GRX3	GRX6	BOL1	BOL4	NFU1	NFU2	HCF101
Rhodophyta		*Galdieria sulphuraria 074W*		+	+		+	+,c	+	+	+	+	e	+	+	+	+	+
Glaucophyta		*Cyanophora paradoxa CCMP329*	+	+	+			+,c	+,c	+	+,d	+		+	+		+	+
Chlorophyta	Chlorophyceae	*Chlamydomonas reinhardtii*	+	+	+	+	+	+	+	+	+	+	+	+	+	+	+	+
	Ulvophyceae	*Caulerpa lentillifera*		+	+	+	+	+	+	+	+	+	+		+	+	+	+
	Trebouxiophyceae	*Chlorella variabilis NC64A*	+	+	+	+	+	+	+	+		+	+	+	+	+	+	+
	Chlorodendrophyceae	*Tetraselmis striata*	+	+	+,a	+	+	+	+	+		+	+	+	+		+	+
	Chloropicophyceae	*Chloropicon primus*	+	+	+	+	+	+	+	+	+	+	+	+	+	+	+	+
	Picocystophyceae	*Picocystis sp. ML*		+	+	+	+	+	+	+		+		+	+		+	
	Mamiellophyceae	*Micromonas pusilla CCMP1545*	+	+	+,a	+	+	+	+	+		+	+	+	+	+,f	+	+
	Palmophyllophyceae	*Prasinoderma coloniale CCMP1413*	+			+	+	+	+	+	+,c	+	+	+	+	+	+	+
Streptophyta	Chlorokybophyceae	*Chlorokybus atmophyticus CCAC 0220*	+	+	+,a	+	+	+	+	+	+	+	+	+	+	+	+	+
	Mesostigmatophyceae	*Mesostigma viride CCAC 1140*	+	+	+,a	+	+		+	+	+	+	+	+	+	+	+	+
	Klebsormidiophyceae	*Klebsormidium nitens NIES-2285*	+	+	+	+	+	+	+	+	+	+	+	+	+	+	+	+
	Zygnemophyceae	*Mesotaenium endlicherianum SAG 12.97*		+	+	+	+	+	+	+		+		+	+	+	+	+
	Charophyceae	*Chara braunii S276*	+	+	+	+	+	+	+	+	+	+	+	+		+	+	+
Cryptophyta		*Guillardia theta CCMP2712*	+	+	+,a		+,c	+	+,c	+	+	+		+	+		+	+
Haptophyta		*Emiliania huxleyi CCMP1516 v1.0*	+		+,b		+,c	+	+	+		+		+	+		+	+
Rhizaria	Chlorarachniophyta	*Bigelowiella natans CCMP2755*	+	+	+,a		+,c	+		+	+	+	+	+			+	+
Heterokonta	Bacillariophyta	*Phaeodactylum tricornutum CCAP 1055/1 v2.0*	+	+	+,b	+	+,c	+,c	+	+		+		+	+		+	+
	Phaeophyta	*Ectocarpus siliculosus Ec 32*	+	+	+	+	+,c	+,c	+	+	+	+				+	+	+
	Eustigmatophyta	*Nannochloropsis oceanica CCMP1779 v2.0*		+	+		+,c	+,c	+	+	+	+			+	+	+	+
	Chrysophyta	*Ochromonas sp. CCMP1393 v1.4*	+	+	+		+,c	+,c	+	+		+					+	+
	Pelagophyta	*Aureococcus anophagefferens clone 1984*	+	+	+,b		+,c		+	+		+					+	+
Alveolata	Chromerida	*Vitrella brassicaformis CCMP3155*		+	+,a			+	+	+		+		+		+	+	+
	Dinophyta	*Symbiodinium microadriaticum CCMP2467*		+	+,a		+	+	+	+		+	+				+	+

**Table 3:** Genomic analysis of the occurrence of chloroplast-encoded *DPOR* and *NDH* genes in microalgae. Taxonomy is based on [49] and https://www.ncbi.nlm.nih.gov/Taxonomy/Browser/ (accessed on 10 March 2021). a: the *Cyanophora paradoxa* chloroplast genome sequence is found under the NCBI Reference Sequence: NC_001675. For DPOR, the presence of *chlL*, *N*, *B* genes in the chloroplast genome was systematically analyzed. * indicates the presence of 2 *chlL* and *chlN* genes. ♯ indicates the presence of pseudogenes.

Supergroup	Phylum	Class	DPOR	NDH	Refs
Archaeplastida	Glaucophyta	Glaucocystophyceae			
		*Cyanophora paradoxa*	+	−	a
	Rhodophyta	Bangiophyceae			
		*Galdieria sulphuraria*	+	−	[72]
		*Cyanidioschizon merolae*	−	−	[73]
		*Porphyra purpurea*	+	-	[74]
	Chlorophyta	Mamiellophyceae			
		*Ostreococcus tauri*	−	−	[75]
		Nephroselmidophyceae			
		*Nephroselmis astigmatica*	+*	−	[76]
		Picocystophyceae			
		*Picocystis salinarum*	−	+	[76]
		Chlorophyceae			
		*Chlamydomonas reinhardtii*	+	−	[77]
		Trebouxiophyceae			
		*Chlorella vulgaris*	+	−	[78]
	Streptophyta	Klebsormidiaceae			
		*Klebsormidium flaccidum*	+	+	[79]
		*Maesotaenium endlicherianum*	+	+	[79]
		Charophyceae			
		*Chara vulgaris*	+	+	[80]
Heterokonta	Ochrophyta	Bacillariophyceae			
		*Phaeodactylum tricornutum*	−	−	[81]
		Coscinodiscophyceae			
		*Thalassiosira pseudonana*	−	−	[81]
		Eustigmatophyceae			
		*Nannochloropsis oceanica* IMET1	+	−	[82]
Alveolata	Dinophyta	Dinophyceae			
		*Symbiodinium* Clade C3	−	−	[83]
	Chromerida	*Vitrella brassicaformis* CCMP3155	+*	−	[50]
		*Chromera velia*	−	−	[50]
Rhizaria	Cercozoa	Chlorarachniophyceae			
		*Bigelowiella natans*	−	−	[84]
		*Lotharella oceanica*	−	−	[85]
Haptophyta		Prymnesiophyceae			
		*Emiliania huxleyi*	−	−	[86]
		*Tisochrysis lutea*	−	−	[87]
Cryptophyta		Cryptophyceae			
		*Guillardia theta* CCMP2712	*−*	−	[88]
		*Rhodomonas salina* CCMP1319	*−* *♯*	−	[89]
		*Storeatula species* CCMP1868	*+*	−	[90]
Excavata	Euglenozoa	Euglenophyceae			
		*Euglena gracilis*	−	−	[91]
		*Eutreptiella pomquetensis*	−	−	[92]

**Table 4:** Distribution of genes encoding Fe-S enzymes involved in the anaerobic metabolism of microalgae. Sequences were retrieved in 25 species from Phycocosm (https://phycocosm.jgi.doe.gov/phycocosm/home (accessed on 10 March 2021)) using *C. reinhardtii* sequences and blastp or tblastn searches [10]. The sequences are available as supplementary information. a: fused *HYDEF* genes; b: the sequence is not present in *Phaeodactylum tricornutum* but is present in *Thalassiosira pseudonana*; c: the sequence is present in *Chlorella variabilis* but not in all Trebouxiophyceae; d: the *HCP* gene is fused to a gene coding for sulfite reductase. In *Tetraselmis*, a *HYDE* gene exists in addition to the fused *HYDEF* genes [128]. Notably, we cannot exclude that sequences in some of the automatically annotated genomes analyzed were reported as missing because of problems in the genome assembly.

			PFLA	PFO	HYDA	HYDE	HYDF	HYDG	HCP
Rhodophyta		Galdieria sulphuraria 074W							
Glaucophyta		Cyanophora paradoxa CCMP329	+	+	+	+	+	+	+,d
Chlorophyta	Chlorophyceae	Chlamydomonas reinhardtii	+	+	+	+,a	+,a	+	+
	Ulvophyceae	Caulerpa lentillifera	+						
	Trebouxiophyceae	Chlorella variabilis NC64A	+	+,c	+	+,a	+,a	+	+,c
	Chlorodendrophyceae	Tetraselmis striata	+	+	+	+,a	+,a		
	Chloropicophyceae	Chloropicon primus							
	Picocystophyceae	Picocystis sp. ML							
	Mamiellophyceae	Micromonas pusilla CCMP1545	+						
	Palmophyllophyceae	Prasinoderma coloniale CCMP1413							
Streptophyta	Chlorokybophyceae	Chlorokybus atmophyticus CCAC 0220							
	Mesostigmatophyceae	Mesostigma viride CCAC 1140							+
	Klebsormidiophyceae	Klebsormidium nitens NIES-2285							
	Zygnemophyceae	Mesotaenium endlicherianum SAG 12.97							
	Charophyceae	Chara braunii S276	+		+	+	+	+	+
Cryptophyta		Guillardia theta CCMP2712		+					+
Haptophyta		Emiliania huxleyi CCMP1516 v1.0							
Rhizaria	Chlorarachniophyta	Bigelowiella natans CCMP2755	+						
Heterokonta	Bacillariophyta	Phaeodactylum tricornutum CCAP 1055/1 v2.0	b	b	b				+
	Phaeophyta	Ectocarpus siliculosus Ec 32							
	Eustigmatophyta	Nannochloropsis oceanica CCMP1779 v2.0			+	+	+	+	+
	Chrysophyta	Ochromonas sp. CCMP1393 v1.4							+
	Pelagophyta	Aureococcus anophagefferens clone 1984							
Alveolata	Chromerida	Vitrella brassicaformis CCMP3155	+	+	+	+,a	+,a	+	+
	Dinophyta	Symbiodinium microadriaticum CCMP2467		+					+

## Supplementary Materials

Supplementary materials can be accessed at: https://www.mdpi.com/1422-0067/22/6/3175/s1.

## Abbreviations

CD	cysteine desulfurase
CSK	chloroplast sensor kinase
DPOR	dark-operative protochlorophyllide *a* oxidoreductase
FDX	ferredoxin
Fe-S	iron-sulfur
GRX	glutaredoxin
HCF101	high chlorophyll fluorescence 101
HCP	hybrid cluster protein
HYD	hydrogenases
LPOR	light-dependent protochlorophyllide *a* oxidoreductase
MEP	2-C-methyl-d-erythritol 4-phosphate
MRP	multiple resistance and pH adaptation
MVA	mevalonate
NDH	NADH dehydrogenase
NIR	nitrite reductase
PFL	Pyruvate-formate lyase
PFO	Pyruvate-ferredoxin oxidoreductase
SIR	sulfite reductase
SUF	sulfur mobilization
