# Supplementary material for: Occurrence, Evolution and Specificities of Iron-Sulfur Proteins and Maturation Factors in Chloroplasts from Algae
Source: Int J Mol Sci. 2021 Mar 20;22(6):3175. doi: 10.3390/ijms22063175 (PMC8003979; doi:10.3390/ijms22063175)
Supplement: Supplementary file 1 [file ijms-22-03175-s001.pdf]

Supplementary material: Amino acid sequences of algal proteins belonging to the SUF machinery or Fe-S proteins discussed in this study.

Most sequences presented in Tables 2 and 4 have been retrieved by blastp search on the jgi phycocosm portal or on NCBI GenBank (mostly plastid genome-encoded proteins). When sequences were missing only in one or two organisms but present elsewhere, we have performed a tblastn analysis against the genomic sequences to assess whether the absence was not due to a wrong annotation or to a genome assembly problem. If there was no significant hit exhibiting the typical motifs representative of the protein families, the sequence was considered as being absent. Only *Chlamydomonas reinhardtii* sequences have been manually curated and reannotated parts appear in red characters. Additional observations appear in red in the defline.

NFS2/NIFS2 orthologs

```
>Cre07.g322000
MAISCLLRHRDDAASARSLHAAANESTSHESSQAVPQVPTVGLGQALPHLTRTGCIYLDYNATTPIFPEVANEMAPFVFEHFG
NPSSGHVYGRACKSALDTARQRVAALIAAASPSEVHFTSCGTESDNWAVYGAVMAARAAAAGAAGAAGYVPHVVASAVEHPAV
LAHLTHLQEQGLLSYTLVPVDGEGLVSPADVAAAVTPATCLVTVMHSNNEVGAVQPIAHIAAAARAAHARLTAGGGGATSPSP
SLSPSSSTGGSSSGTSGSSWRPRLLVHSDAAQSTGKVDLDVGALDLDLMTLVGHKFGAPKGVAALYVRHGTALPNYFYGGGQE
GGRRAGTENVMQVVGLGAAAAITVRERRQLQTHMGDMAQRLLAAVRAQVAPAEQDKLRLNGPADPTRRLPNTLSLSIRGLNSG
AALAALSGRLAASAGAACHSSGAASVSAVLRAMKVPTDYATGTLRLSTGRHTTTAEVDEAAGLIVAEARRQGVLAH*

>jgi|Cyapar1|22355|g21891.t1
MEKPIYLDFNGSTPLSREVVDAMKSVLDSGLGTGNPSSSHFYGAESKKAIEAARKQVAAALHCAEDELIFTSGGTESNNYAIK
GYFAANKGRGNHIISSTVEHPAVTQVCDFLAETAGARVSYCPVDGTGRLDLAALEALITPATILISVMHANNEVGTLQPIREI
VEIAKRGGRNIAVHTDASQSVGKVPVRVDELGVDMLTVAGHKLCAPKGIGAVYTGRGTAVERQMHGGDHELKRRAGTENVLYA
VALGTAAAAAARDLEKNMAHCRRTRDRLLARFKEAGLDIRVNGHPEHCLPNTLSVGFGRIEAPTLLSELAASVSASAGAACHS
DHKDMSGVLKAMKVPAVYGMGTVRFSTGATTTEEEVDRAAALVVAAVRRLTPGEEAGAGDGEGEGGPEGLPALADVRMTRYTK
GMGCACKLKPQLLEGVLRGLPRATDPRVLVSTETSDDAAVYKISDELAAVHTLDFFTPVCDDPYEFGAVAAANAISDVYAMGA
TPATALSIVAFPSLRLPITALQRILQGAADKCAEAGVSIVGGHSIDDPEPKFGLSVTGYGHPERILRNSSARAGDVVVLTKPI
GTGVLTTATKRGVASAEEAAACAMAAVGPAVHAATDVTGFGLAGRRARLGGRALLPGAFAHAAAGIVPGGTVSNMEYAGRWVR
WGDGVGETTRALLCDAQTSGGLLLAESAEALLAALRERKTLAAAAVARLTAPGPGIITVR*

>jgi|ChlNC64A_1|144931|IGS.gm_8_00259
MPRLGEPLPHIQASGCVYLDYNATTPIFPEAADEMRPFLTAFGNPSSAHAFGRPCKAAVDAARARVAAMVGADPDEIFFTSCG
SESDNWAIWGAVMEARRQQRAAPDTSGAAAFLPHVVSSRIEHPAVIECLAMLAAQGLLEYTLVPVSCEGLVRVADVEAAMTPH
TCLLTFMHSNNEVGSIQPVAQLAALARRAGALMHCDAAQSLGKVDVDVQQLGVDMLTVVAHKFGGPKGVAALYVRRGVRLERL
LCGGGQEGGRRAGTENVVLVAGLGKAAELVSRELPATAAHMAAMRDSLQQQLLAGLPPSTALIHGPADDALRLPNTLSIGIRG
IAAARLLAELSEQLAASAGAACHSGGGHGVSAVLQAMAVPTEHAVGTLRLSTGRHTTQQDVDRAAALILDYVQRHGGSS*

>jgi|Chlpri1|4911|rna-gnl|IITBIO|A3770_07p49110_mRNA_A3770_07p49110
MAKEVASSRRCIYLDNNGTSPIYREVAEEMQPFLFDHFGNPSSSHAYGRVPKQALDLARERVANLLGAKASQVYFTGSGTESN
NWAIRGALEYGSQKRPKRSDFLPHVVTSNIEHPAVAEALAKYKREGLCDYTEVAVDTKSGVLSVEAVEAAFRTGETVLCTVMH
SNNEIGSLQPIAEIAKIAHAHGALMHTDGAQSVGKVDVDVEALGVDLCTVVGHKFGASKGVAALYCSLDSREFPSLLHGGGQE
NGMRPGTENVLLISGIGKASEIALLEGKEIRRNMLKTRNLLRGRLVEALGSHGIDYRINGPAEEANSLPNTLSISIPAVQAQE
ILADLSESVAASAGAACHSGGFHVSGVLKAIGLDDAHARGTLRLSTGRLTTEEEIERASDLIVEAVLRATSSSG*

>jgi|MicpuC3v2|5257|wlab.205949.1
MPCAIVAAPAPSPLASSVARRRGRRRRGPPSHVASAKPSSSSSSSSSTDLAAASASVSVPAAPPKPPALLSAPSTPGLVAGLV
FGGLPLLAAALRRKEESAEEADADRSSRRPRPASPLPAATTASAMTTTTTPPPSTPLPKGEGAIGSKLPGRAHETCAYLDYNA
TTPIYPEVAAAMEPFLWEHFGNPSSGHAFAAPCRDAIATARNRVATVLGCAPDEIVFTSCGSESDNHAIASAVEHARRSAGGR
GGRVPHVVTTAVEHPAILEYLVSEEAKGTLAYDAVRVNAEGIVDPGDVAAAVREETCLVTVMHANNEVGAIQPVAACAAAARA
KNPRVLVHADAAQSLGKIPVKVDLLGVDMCTIVGHKIGAPKGVAALYVKKTAPFSKLFHGGGQESGRRAGTENVMHVVGLGAA
CALVTKEEDALPSYMATLRDAMQRALVAELGGDDGAGVRVNGPANDADRLPNTLSVGVKGVSASVLLQTLSSSVAASAGAACH
TGAAAASISSVLRAMEIPEAYAVGTLRLSVGRHTTTSDVEVGVKRIADAARAQISEIDAMPEGERPAWCVRNVFK*

>jgi|Chlat1|251|Chrsp1S03050
MGGVGGSEVQEVQSDGEAKPIYLDYNATTPIDKEAADAMLPFLYSEFGNPSSSHAYGQRAKAVVESARAELAALIGCHPSEIY
FTSGGTESNNWAILGTALHAREARGGGVQGVHIVTSAIEHPAVLEPCRYLERHHGFDMTVVGVDSQGLVDVDAVREAVRDDTM
LITVMHANNEVGSIQPLAEVAAVAWERGIPLHTDASQSVGKVPVDVGTLGVDMLTIAGHKFYGPKGVGALYIKSSHRLCKFMH
GAGHERGMRAGTENITLIAGLGAAARIARRGLQQNMEHMVRMREALVRELTPPLDGVRYLINGPQDALKRLPNTLSISIEGVS
ASDVLHDIETDVAASAGSACHAGTSTVSSVLKAMNVPVEFALGTLRLSTGKPTTQAEVTEAARIIAQSVRKYTTSSKQQSTSN
*
```

>jgi|Mesovir1|3004|Mesvi192S03833
MSACYANSLNLASVAKIPASFPLLGRGNYSGKNTRKRRVYAKAAMQTSGQPANMPHTVPSDRGAIYLDTAATTPIYEEVAQAM
LPYLLKHFGNPGSRHAFSSPTRSAVLHSREQVAVLLNVDKDDVFFTSCGTESNNWAIFGTVARAERFLASHDSPAADTAAQGT
PRLPHVISSVIEHPAILKCLQYLNACGRIQYDLVPVDAQGLVDPAAVAAAVTPTTTLVTIMHANNETGAIQPIAEIVKHVRER
ASSVLLPGLQLLIHTDAAQSVSKVDVRPQDWGVDLCTLVGHKMGAPKGIAALYIRDGAQIDNFLHGGGQEAGHRGGTENVLLI
VGMGEAAAIAVKEAGVTPKNMRKLKGLFLSLLREGLGAENVRVNGPPVDSPMALPNVLSVGIRGIEASAFLDTLANDVAASAG
AACHAGEASISSVLVAMNVPQEFAKGTLRFSFGRLTTEADVMTAAELVIAARK*

>jgi|Klenit1|2855|rna-KFL_000530350
MDASTGSSSLEHPQRHPLGWEKLEGAPIYLDYNATTPISPEVSAAMLPFLQGFGWGNPSSTHVFGEQAKRAVEYARRQVADLI
GCAEDEVFFTSGGTESNNWAIKGAVEAALAHLPPGRRSGPAFRLVTSAVEHPAVTEVMEHLRDSRGFDLRVAPVDSQGLVDAD
AVIAAAFGGAESGDLGRLESVTSADDSFQHSEASKVTNGIKEEGQAPESVEVAKAIMTDRSAVEETSTTGVETEGAEKAGPVK
VLNGVFDAKVDGKVDGVGQGNGQASQTLIVSIMHSNNEVGAIQPIAEIGRALRKAGVVFHTDASQSIGKVPIKWDELEVDLLT
IAGHKLYAPKGVGALIVRRGTRLAKLMHGAGHERGFRAGTENIMLIAGLGKACEVSRRDMARNVAHVTDVRDRLLSRLQQNLA
SGFQGSKRLALKVNGPIDAAKRLPSTLNISMSGVDGPRLLADLAPLVACSAGSACHSGGHGSPVLKAMGVPPEFAACTLRLSV
GRETTEEEVDAAASCISEAIKKQHEFELPTIAVPDSFVSLFRQLSYSMGSPSPKKGG*

>jgi|Chabra1|325782|rna-CBR_g8345
MAFLFLALLCCLFTPSDSEVAEAILPFLYEEFGNPSSSHFYGKKAKLAVENARREVALLIGCKADEVVFTSGGTESNNWAIRC
GAQANSRMKGKHLVTSSIEHPAVSEVINHLCQNEGFCKTVVPVDLEGRVDAEAVVKVATADLETSLVSIMHANNEVGSLQPIA
EIAASLRGKGILVHTDASQSVGKIPINWRTLGVDLLTIAGHKLYGPKGVGALIIRRGVILPKFMLGAGHESGRRAGTENVALI
VGLGKACEVARKGLDRNMRRSRAMRDRLLEKLEDALPGLRCRGDDIELSRIDFRVNGPADPEKQLPNTLSVSFKGVDGVKLMA
AISPFVACSSGSACHAGHVAVSPVLAAMGVPAEYAQCTLRLSMGRETTRTDVDTAAEVIMSHIKSLFPSGVTCT*

>jgi|Guith1|109959|au.42_g11061
MVYLDYNATTPLDEKVQQAMKPYIEGRFGNPSSSHWYGVKEKEAVAKAREEVADLIGCSSEEVFFTSGGTESINWALKGTAER
LRKTKRHIVTSAAEHVAVLECCKYLEEVHGMEITHVGVNEYGEVDPEQVRAAIREDTALVTIMLANNETGTINDIRSIAQIAR
SKGVLVHTDASQAIGKIPVDVNDLGVDFLTVAGEHAKHVVANTLTDSGTWPGHKLYAPAGVGALYIRHGTELPNFMHGAGHER
GRRAGTENTMHLVALGAACKAIPGIELNGHPERRLPNTLNVLNLPFSCVRSHGDLQIIAQTADRLAFSAGSACHAGQTGHVSH
VLKAMNISKDKAFRCCHLRIRNHH*

>jgi|Guith1|139922|fgenesh2_pg.41_#_133
MFGKGDPIYLDYGGTSPVDPSVFDSMKEFFVSNWGNPSSSHVYGQLTTGRGLKEVSKKREKTWLIALDALLMKWGTESNNWVL
KDSPDMNQGAVSLARKRLQDHFTSNMGGSALAWSSMYVDQLRPHIVTTAIEHPAILEPLKWLETEQQCDVTIIQPGKDGSKDY
DIAGTSPREREPGVVNVQDVVDALRTQTILVSIMHANNEVGAVQPIREISDVNDLGVDLLSMAGHKLYAPKGIGALYVRGGAK
GLDGDGDFRLQRIMHGGGQELGLRAGTENVGLAVGLMRQASLIRSLHVQLQAKFIGQIHLNGPELPASPINGAWPRLPNTLSI
GFRGLKAQEILGKVQDVVSASAGAACHSSNIPKISSVLAAMQVPEDAALGTIRFTVGRYTTEDEINRAAEAVADAANELFKQS
V*

>jgi|Emihu1|424605|estExtDG_fgenesh_newKGs_pm.C_3090007
MSVREQKLATTPVWPEVAAAMTPYLTHHWGNPSSGHAFGRPCGAAIVQARAAVAELIGAEPSEICFTGCGSESDNFAIVGALE
MEEARRRGAGGAALPRPHVVASNIEHPAVELCIEALCAAGRLDATYVPVDEEGLVSAEAVAAATTEQTFLVSVMHSNNEVGSV
QPVRQIADAVRALRPSVLVHTDAAQSIGKVDVDTRALGVDLLTLVGHKFGAPKGIAALYIRSGLALPRMIHGGGQESGRRAGT
ECTPMVVALGTAAAIARAEAAPLREHMRRTRDALESALVERLPAGSTRVNGPADASRRLPNTLSIGLRDVRAAELLETLSERL
AASAGAACHANAASVSAVLRALRVPPEFAVGTLRLSTGRHTTLEEVALAADLIAEEARRQWGQGDGAAGKVSDSGPS*

>jgi|Bigna1|92143|estExt_fgenesh1_pm.C_40030
MGATCKPAKGPSVTYLDYNATTPIDPRVQAVMQPLLSSGWGNPSSSHVYGKVANAALKAARQKVAAAVGCADSNEIVFTSGGT
ESINHAIRGAAFASKRIRYGGNHIIISSIEHVAVSATCRYLQDHHGFKVTTLPVDGSGRVSPEALKKAITRKTVVVSIMMANN
EVGTIQPIAELVKVAKAASKKIIFHTDASQAVGKIDVNVQELGVDLLTIAGHKLYAPKGIGALYIRNELPVTLEPLIHGAGHE
MGRRAGTENILLAAGLGEACQIAKQDLEERMRHMKLCRDHLQRKIMDEFKGALVRINGDEKARLPNTLSISFHGLASHEILHD
IQDLVACSAGSACHSNEVRSISIRISHVLAAMKVPLDFAKGTLRLSTGINTTIEEADKAAKFVCETNIPLLV*

>jgi|Phatr2|2966|gw1.19.75.1
IYLDYNGTTPVYPQILQAMLPYLTTQFGNPSSGHLFHRQPRQAVDRARKQLLTLLGQPEADLSSIWFTSCGTESDNLAIQLAL
QSSSKLISTRFGAHTLPHIVSCNVEHPAISGYLDALVEEKVCEVTYVPVQSDGMVSADAMIAAIQPQTILMTLMLANNESGAL
QPVQKVAQHCSKANILFHTDAAQAVGKVSVDLDDLGHPDMISIVGHKMGAPKGIACLYVRPGCCEQHGRALHNRGILLIGGGQ
EHGRRGGTENVPYIVGFGEAASLATKDWKSNSIKMEGLRTRLLFNLENWLGKDMVRTNGPSNPAHRLPNTLSVGLKGIHSGAL
LAAIGDQVAASAGATCHSASGVSSVLKAMGVPETFARGTLRLSLGTHTTEQEVD

>jgi|Ectsil1|32276|rna4872
MHLLSMFSVAAVAAGMCVGSVTAEVTAAAGIETPATTGNLMRFSDAGFWYIVAMTFFVLVIVDATNKFFSPRACLNTDGMSSK
TGIAVALPYRGTRRCIYLDYNATSPIFPEVSREMFPFLGECFGNPSSGHALGRACKKAVVLARARVASLLGCSPEEVVFVASG
SEADNHAILASLALSSKAATAPPSAASSSLPHVVSSAIEHPAVTKCLNHLVAEGKVEVTFVGVDEEGRVSVEGVVGAFRPETA
LVTIMHSNNEVGSLQPITEIAEACRQRGIMCHTDAAQSIGKVPVKVADLGVDMLTLVGHKFGAPKGVAALFVRRGLALPPMIH
GGGQEAGRRAGTENVLLISGMGKAAEIVDVELERLSKHLAAMRERLLELLMAADLGDGVKVHGPRSPSLRLPNTLSIGLPGVE
ARVLLERVRGA*

>jgi|Ochro1393_1_4|849870|fgenesh1_kg.4_#_1171_#_TRINITY_DN23625_c1_g3_i7
MLKRNRITKKPAAGKSQDSEKIIIPNKNLSEQTDPSNVRTSYITGNSSGKQHEHGKKENTSQVGIPHPYTKRQEYSGCVYLDY
NATTPIFPEVTSAMLPFLSDFFGNPSSSHVFSKPCRAALDQARINVGRLVNAANSAKEIYFTSCGTESDNRAIDIAIHHFRQH
KKKLLAVRGVASANIVSVPHIITCSIEHPAVLVYVRSLEAQGEIRLSIVPVNTEGIVDIAMLKRELSPNTALVTIMHSNNEVG
SIQPIREIANCVREFNQRLSHGEACVLMHSDAAQSLGKVSVDVQGLGVDLLTIVGHKFGAPKGVAALYIRSNVQTTPMLVGGG
QERGVRGGTENVGLVCGLGEASRLAYTEASELLLHFLVLKLQLVTSLVEGFSAQQVAECIRFNGPQRSCDISELSSDLKLLRV
MLKSAPSMNIEQIANQNNGNNSRTAAAASTTSSGSSSRAVGAIDLLEQLPNTVSVSFKNIHSHLLIHHLSSRVACSAGSACHA
VTTPATAVDNTRSDTDKSGTNSVGSGANNSSSTNSSNSGAQTQSSAKAAGMNSRGKVSDVLLAMKVPLDFAQGTLRLSFGRHT
TATDIEAAATHIIAAVKSQWATNGYSG*

>jgi|Auran1|11983
VYLDYAATCPIYPEVADAMVPFLYDHWGNPSSGHAYGAPCRSAVADARGSIARLLGCASEEVAFCSCGSEADNWAILASLGGK
RRHVVVSAVEHPAILACVDALEREGRCDVSRVGVDARGVVDPAAVADAVKPGETALVSVMLANNEVGSVMDVAAISAAVKARD
AGILVHTDAAQAVGKIDVNAKRLGVDYLTLVGHKFGAPKGVAALYHRRGAPLRSMLFGGGQEGGLRAGTEAVPSVVALGAAAA
IWTREGAAIAAHSAAMRDRLRRRLEAALGPERCAVNGPLGAGGEALPNVLSFAVRGARAARVLGDVGDRVAASASAACHSGSD
AVSAVLLAVGVETELALGTLRLSVGRHTTAREVDAAADVL

SUFS1 orthologs

>jgi|Galsul1|1124|XM_005707326.1
MTHCAMNRCGGWKTSCSFVTQCGFKEENVFGKRWCSPSFSVKRLRSLRPQRLLTCATLLSSPYSDTLNNLQCPVKREDFPILQ
QTIENKKLVYLDSAATSQKPLVVLEALQKYYLHNNANVHRGAHTLSSRATEAYERARQILQHFIGAESREQIIFTRNATEAIN
LVAYAWGLHNLKPGDELILAISNHHSNIVPWQLIASRTGAILKYVVLDNQEQDSLESFKMLLESGKVRLVACSHVSNVLGFIN
DIKPITRWAHSAGALVLVDACQSVPHMPVNVVDLDCDFLVASGHKMCGPTGIGFLYGKESLLENMSPFLGGGEMIADVYEDYS
TFAVLPHKLEAGTPAIAEAVGLGAAVEYLQSIGMDVIHSYERCLSEYLYRHLIDIPNVTIYGPKDVKRRVALCSFNIQGIHAS
DLASILDLEGVAIRSGHHCAQLLHRSLGISGSARASLYMYNTKEEIDSFIGSIYRSIDMLKR*

>jgi|Caulen1|89248|g4590.t1, bad annotation
MVQSPSISGSSLADTVRPDFPILTQEVNGKPLIYFDNAATSQKPKSVIEAMDRYYTITNSNVHRGVHALSAEATSLFENARSK
VANLVRAKDRSEIVFTRNATEAINLVAYGWAMSNLKMGDEILISVAEHHSNMVPWQIVAKKTGAQLKFISLTPETEELNMEHL
RELISDRTKLISTFHASNVLGAISPVKEISRIAKKYGSKLLLDCCQSVPNMSVDVGELGADWIVASGHKMCGPTGCGFLWGKY
EVLEDMEPFMGGGEMIEDVFLDHTTYADPPARFEAGTPAIAEAIGLGAACDYLTSIGMENIRSYEEEIGTYLYNKIVGLNAVK
VYGPPPSQGRGPLCAFHLDGVHPTDLAMVLDQYGDANINKGV*

>jgi|Cyapar1|25252|g24670.t1
MDRQAFALSAPARGSFSPASCPTGLASAQNTCDRAPRRAHRSLVAGASLNGASSSEASRRARFVLPSEFVGRSPASRSAGSDQ
RGVSACPPPAPPPTCAAVETRSLGDEVRKDFPILDQEINGQPLVYLDNAATSQKPNAVLDVLRRYYNEDNSNVHRGVHTLSAR
ATDAYEGARRIIANFINAEDPACVIYTRNATEAINLVAYSWGLTTLKPGDEIVLSVMEHHSNLIPWQIVAQRTGAVIRYGKLA
EGGVLDVESVEGLINERTKLVSLAHVSNVLGCFNPVKRIGAAARAVGARMLVDACQSVPHVPVDVQDLACDWLVFSSHKMCGP
TGIGALYGRREVLEAMPPFMGGGEMIQDVSLEHFTCNTLPHKFEAGTPAIGEAIGLGAACEYMSGIGMGRIHEYEMELTRYLW
KRMGELPFEVELYGPHPDAAGPEGRAALCAFNVKGVHSNDLCTILDQDGVAIRAGHHCTQPLHKELGVSHSARASLHFYNTRE
DVDRFIKALADAVDFFTSV*

>jgi|ChlNC64A_1|34562|estExt_Genewise1Plus.C_50023
MPAPQVQDAPAAAAAAADLGTATRADFPILHQSVNERPLMYLDNAATSQKPRQVLQTMDEYYGEGGYNSNVHRGVHALSARAT
AAYEAAREKIAGFINASSPQEIVYTRNATEAINLVANTWGAAQLREGDEVVLSVAEHHSNIVPWQLLAQRRGLVLKFAELTGS
EEVDLEKLAALITPRTRLVSLVHVSNMLGCVLPTQRVAEMAHGVGAKLLLDCCQSVPNMPVDVQTLGADWIVASSHKMCGPTG
IGFLWARYSLLEQMPPWMGGGEMIQEVRLEGSTYAEPPSRFEAGTPAIAEAIGLGAACDYLSGLGMERVAAHERELGAHLYWQ
LRSIDRVRIYGPSPEARLGRAALATFNVEGIHPTDISTILDSTGVAVRSGHLCTQPVHRHLGISSSVRASPYIYNTKAEVDAF
VDALKDAIQFFT*

>jgi|Chlpri1|3067|rna-gnl|IITBIO|A3770_04p30670_mRNA_A3770_04p30670
MTTTTTTTMVQCSRSRWGCPRGGFRGASSVAARSAAQGTSPRLTAEDVAGIRREFPILEREVHGDKRLVYLDNAATSQKPRRV
LGAMEDYYADYNSNVHRGVHALSAKATERYEEARDKVARFVGAESSREIVFTRNASEAINLVAYTWGEENLGEGDEVVLTVAE
HHSNIVPWQILSRRKKFTLKYVRLDEASQTLDLEGLGEAIGPRTKLVTVGHVSNVLGCVNPVGDIVALARSGGHPGCKVLLDA
CQSVPHMPVNFSELQVDFAVCSGHKMCAPTGIGFLWGKMEVLEAMPPFMGGGEMIQDVFESHSTFARPPGKFEAGTPAICEAI
GLGAACDYLSDIGMHRLEEFELEMGTYMYEELEKNIEGIEIYGPSPKQAPRAALAAFNVEGLHATDISMILDQYGIAVRSGHH
CTQPLHHHLGINASARASLYMYNTKEEIDQFVAKLKDTIDFFGQAS*

>jgi|Pico_ML_1|55742|g1387.t1
MRATIPTIATPASRRRNARHAKTSAQATGVVGNSQDLAHRVRADFPILERQVYESFPLVYLDSAATSQKPRCVIEAMSDYYMQ
YNSNVHRGVHALSSQATEEYETARSKVADFVNAETDREIVFTRNASEAVNLVAYSWGLDNLKEGDQVVVSVMEHHSNLVPWQI
ICKRTGAKLCHVPMTEDEELDLNRLEEITKSGQTKLIALAHVSNTLGCITPVEEVVQIAKNCGAKTATKDDRIVQEDLITGRP
DNNVTEHIVEKLGAQLHLRENHPLSILRSAIEEWFAQEHDGKFRCFNDLPPLVTTKANFDDVLVPADHVSRSMNDTYYVDANT
VLRCHTSAHQAEMLRQGERKFLVTGDVYRRDTVDATHYPVFHQMEGVRIFEPEEWERAGTDGTKLAEAELKKTLEGLARHLFG
DVEMRWIDEYFPFTDPSFELEIFFNNEWMEVLGCGVMEQAILEEAGLGNKKAWAFGLGLERLAMVLFEIPDIRLFWSTDERFL

SQFKSGDMSAKFKPYSKFPPCFKDVSFWIPETFTENNLCEVTRGIAGDLVEEVKLIDEFHNPKKGKTSHCYRIAYRSMDRSLT
DEEINQLQENVRSSLVEQLGVELR*

>jgi|MicpuC3v2|917|wlab.201976.1
MATMATLTLAASASAPRASASAASRDGAPPPRGASPASHGSRARVAAASPPNPPRLPGTARTRASVSTAAASTSVRSTLADEL
RGDFPILHQTLPDSGKPLVYLDSAASSQKPNVVIDAHAAYYREINANVHRGVHYLSGKATDAYELARVKVADFIGAETDREIV
FTRNASEAINLVAYTWGVANLKPGDEVIISELEHHSNIVPWQLVCAQTGATLRHVGLAPDGNGIDMDALEASAREGKTKLIAT
AHVSNVLGSEVDVPRLVKLAKSVGAKVLLDACQSVPHMPIDVKTLGVDWIVASGHKMCAPTGIGFLWGRADVLETMPPWMGGG
EMIQDVFMDRSTYAPPPARFEAGTPAIGEAIALGAACDYLSAIGMDRVHEHEVDVGTRLYERLAAIDGVTVYGPTPAKGRASL
AAFNVEGLHANDVCTLLDASGVATRSGHHCAQPLHAALNVPASARASLYLYNTREEVDAFARTLEETIAFFREINAGM*

>jgi|Chlat1|1843|Chrsp14S02228
MVAVAVAGSSGSSAAVAGFRVGNRVAAGVVDGEGVWQVARASLPGRSGRRRTSSRGVAVRLPAAQRGVSAAASVSASQVAEKQ
SQASTASPEVSGKLSPEALRQDFPILHQENDFGVPLVYLDSAATSQKPKAVLDALTAYYEGFNSNVHRGVHTLSAKATTAYEE
ARVKIAKFVNAASDREIVFTRNASEAINLVAYSWGLKNLKPGDEIILSVMEHHSNIVPWQILSQQTGAVVKFVGLTPEETLNM
EEFRSLLSDRTKLVSLVWVSNTLGCFNPVEEVVQLAHAKGARVLVDACQTVPHMPVDVQAIDCDWLVASGHKMCAPTGIGFLY
GKLDVLRGMPPFMGGGEMIADVFLDHSTYAEPPSRFEAGTPAIGEAIALGAACDYLSSLGMDRVHALEEELGLYLYEQLSQVP
KVRIFGPKPAADGTGRAALTAFNVEGLHPTDISTFLDLSFIAIRSGHHCTQPLHRYLNISSSARASLYFYNTKEEIDTFVREL
RNTINMLTGAGDDGFQQA*

>jgi|Mesovir1|5648|Mesvi433S09449
MAAYYEGMNSNVHRGVHYLGTLATEAHNLARTKVAKFVNAARDEEIVFTRNASEAINLVAYSWGMNNLSPGDEIISTVMEHHS
NLVPWQLVAQKTGAKIVHVGLTKDQTFDMDHFRSLLSDRTKLVAIGHVSNALGCTNPVREVCQLAHARGAKVLIDACQSVPHM
RVDVQSIGCDWLVASGHKMCGPTGIGFLYGKYDVLKAMPPYMGGGEMIVDVFLDHSTYADPPMRFEAGTPAIAEAIALGAAVD
YLTGIGMDRVHDYEVELSTYLYHEMEKVPGITLYGPRPEIGRAALCSFNVEGLHGYDVATLLDNKGIAIRSGHHCTQPLHRHL
GIAGSARASLYIYNTKEEVDVFVAALTDVVKFFRELGM*

>jgi|Klenit1|6882|rna-KFL_001880270
MPPACKRAAVRAIAAPERAAATVSAPAKSLGDETRPDFAILHQEVEGNPLVYLDNAATSQKPRQVLQALENYYKGYNSNVHRG
THKLSAMATDEYEKARIKVAKFVNSPTDRQIIFTRGATEAINLVAYSWGLNNLKAGDEIVLSQMEHHSNIVPWQLVAERTGAL
IRFVGLTEHEQLDMEQFRNALSNRTKLVAVVHVSNTLGCLNPAEEIVRLSHSVGAKVLLDACQSVPHMPVDVQALDADWLVAS
GHKMCGPTGIGFLYAKWDLLLSMPPWQGGGEMIRDVYFDHSTYQEPPSRFEAGTPAIGEAIGLGAACDYLSSVGMERIHQYEV
EVGRYLYEKLSDIPEIRIYGPPPSLADRASLCAFTIEGVHATDLASHLNDFRGVAVRSGHHCTQPLHRVLGVNATARASLYFY
NRKEEIDVLVDAIREGIDFFTAPLEPPPGF*

>jgi|Mesen1|2876|ME001742S01985
MLTIKNLHAGIEGKKILKGINLEIKPGEVHAIMGPNGSGKSTLASVLAGRKEYEVSTGSVQFLGKDLLDLSPEERAGEGLFLG
FQYPVEIPGLSTTNFIKTALNEVRKYRGQEPLDAVAFLKLMKEKMALMNVDQSLLSRSLNEGFSGGEKKRNEIFQMAMLQPKL
AILDETDSGLDIDAIRIVANGVNQLRNKDNAVLAITHYQRLLDYIIPDVIHVLYNGRIVKSGPKELALELEERGYDFIKTNGY
SDIVSAHFGHSSGYIKDGIHALNTAFTEGGVFIHLRKTKHEKQPVYIYHLTTAEHEPGFTQPRSLVYLGEQAEMQLVETSMCL
GQQDSLNSQVIEIVLEEAAVLEYYKIQHDHDNASQVNTTHIRQIGKSYSHTVTLSLNGNMVRNNLNVVLEASHSEAHLYGLYF
PKGKSHIDNHTIVDNRVPHCESNELYKGILDEQGTGVFNGKIFVRQPAQKTNAFQSNKNVLLSDTAAINTKPQLEIFADDVKC
SHGCTVGRLSEEGLFYLQSRGIPEKTAQSMLLHGFAEDVLEKIKLEPIRRYADALIRIRYCMITTATIPVFDVYALRKQFPVL
EREVKGKPLIYLDNAATSQKPAVVIDALVQYYTGYNANIHRGIHTLAEEATAAFEATRETAKHFIHAAGREEIIFTRGTTEGI
NLVAYTWGRANVGKGDEIIISAMEHHSNIVPWQILCEEKGAQLKVIPVDDAGELIMEAYEKLLSDKTRLVAITHVSNALGTVN
PVKQMIRSAHKAGALVLVDGAQSTVHLDIDVQDMDCDFFVCSAHKLYGPTGVGVLYGKKHVLEAMPVFQGGGEMIKEVTFEKT
TYNELPYKYEAGTPNIADTIVFKTALDFIEQTGKEQIRQHEHELLQYATAALETVPGLKIIGRAKEKTSLVSFVIDKVHPQDI
GILLDNKGIAVRTGHHCAQPLMQRYGIPGTIRASFAAYNTKEEIDELLLGLEKSIKLLV*

>jgi|Mesen1|4618|ME000236S03869
ARATDEFERAREKVARFVNARSSREIVFTRNASEAINLVAYSWGAAANLQKGDEIVVTVAEHHSNIVPWQLLCQRTGAVLRVV
ELTPGEQRVDVEQLRQLVGARTRLVAIHHVSNTLGAESPVAAAVAAARAHGAAVLLDACQSVPHMPVDVQRLDCDFLVASGHK
MCGPTGIGFLYGKSEVLQAMPPFMGGGEMISDVFMDHSTYAEPPSRFEAGTPPIAEAIGLGAAVDYLTDIGMDRIHAYEMELG
RYLHEQLQAVPGVTTYGPPPDSNGEGRSALCAFNVEGVHATDLSTFLDQQHGIAIRSGHHCTQPLHRYLGTNASARASLYFYN
TKDEIDTFIAGLKDTIDFFTSFSDA*

>jgi|Chabra1|349042|rna-CBR_g45931
MASTATAPSLAGASMLPSPSSPRLASGKVVAAPPITMQWVQRTAQPRAMPDRSTNSFSPCRFAAARSCASSSSSPSSSSSLSS
LSRSASCRSHSGEVVHRVILVPAAAAKERKSAPRRRKEARADPLPSFDLTVTALTAGRCRRRYRSDAVGKERASNRCYPPTGC
MAPALRAAEDMTAHGRGRRRRWCRPSCRSNNVEGTAGIAKGERRDRVGAKGSRFRLPTAAQRVTKGPGFAQGASIPDDVGQDN
AAAAAAAAYCYSKTPYSGFGGVAPPRSRRKVKVINTLIRCAAAISSVDDASSSSPSSPSSSAAAAAAAVSVGTTTSLGEITVK
DFPILSQEVNGRRIVYLDNAATSQKPRQVIDAVQRYYQDYNSNVHRGIHTLSVKATEAYEDARGKIAKFINAERDSEIIYTRN
ATEAINLVANTWGVANLTPGDEVVLSVMEHHSNLVPWQIIAERTGAVLRFVSLSQCESLDMDHLRSLVGNKTKLVALQHVSNA
LGCVNDAVMIGEIAHKYGAKVLLDGCQSVPHMPVDVQALDCDWFVGSGHKMCGPTGIGFLYGKMEVLKAMPPFLGGGEMIQDV
YLDHSTYAEPPNRFEAGTPAIGEAIGLGAACDYLMGIGMGKIHDYEVELSGYLYKKMAELEGMKIYGPKPTDPGGRAALCAFN
VPQIHPTDLSMVLDQSGFAIRSGHHCAQPLHRHFGINASARASLYFYNTKEEIDMFIEGLKEAIEFFSVAGGV*

>jgi|Guith1|121029|au.210_g22131 C-terminal missing

MAGTAPALVMALLVLGGWTTQTEGATNVMCGNCSFTVSDAGLLQRTGSCLAGTGPCVLKAAGIAEIERGTFDGLADMTYLDLS
HNQLTNISQGLFDGLSSLQLLDLRYNQLSSIAPDAFSNCTQLSSIDVGFNQLTCYYASWPLLAMALDSTVLARLCQNVTTDVT
PCYPHIRVELDSTSLPSDMAGEYCEAFNCAHGEGISPGRDDSSHCFRKNGDEAWKILNTGCGWTIGHIEQNGERLYPSRLFQW
QEYARSYIGDCSLIPPSQLDVRALANTSNFYDGGGRIVVGIHTVLVLPPHHSSDADPSADIFNATSAVNSSSASTPLPLVDTS
LLSFPECVEQRDWAAMLELLRNGTAATGGLGLMMEPPLESWVCIREHPCLQRIDGAQPSDMMCFVYHEVDLVFLPWSTNSLLK
LVGSRMSRARVAWAWRMLLLVAGLGVRQVCTFRAPHLTSSMGYRGIHHQLKVSMMRASSTSLSLGKEVRKDFPILKQKVHGGK
DLIYLDSAATSQKPVQVLEAMKKFYEESNSNVHRGSHALSVKATDMYEGAREIVAKFVNARREEIVFTSGATEAINLLNSQQG
FDMDHFRSILSDKTKIVAVVHVSNMLGCINPVQEIVAAAHAVGAKILLDACQSAPHMPLDVRLLDCDFLVASGHKLCGPSGSG
FLYGKKEILESMPPWK

>jgi|Bigna1|147067|aug1.128_g21775
MSMGEALQGEFPILMQKVHEGKPLLYLDSAATSQKPRKVTEVLREASKNATTFLDESDIQSGINKARSKVASLINAEPEEVVF
TRGATEALNLVARGWGDLHVSEGDEIILSVMEHHSNLVPWQNLAERKGAILRFVPLSNDQAFDLEAFKSMLNPGKTKIVAVNH
VSNVLGCVNPVNEIARLTHEAGAVLSLDACQSVPHMPVDVRELGADFISASGHKMLGPTGIGFLWGRKELLETMEPLNTGAQI
FTEVSLDPSAPPPSTAFLPVPWRFEAGGVPYPEAVALGEACRYLQSLDMGRVHSYEVELGRYLWESLSEMEGVRLYGPSPDSA
AGEGRGALVAFNIPGVHANDLAFFLDQEGVAIRSGHHCTQPLHRSLGIAGSARASLYVYNTRKDVDRFCKKLQKTLKMFRDMG
FLVEEKSPCPLVSTADPTLKDPAINRPCTDLWPRVEEEEMGEESAAS*

>jgi|Phatr2|19482|estExt_gwp_gw1.C_chr_50292
MRPSNTILSFVFTLLLLATCLGASHGWTSRVAVGSSRRAVVTFVSVHDGSTENASIGDTHTDRADFPHGYIATRVRPDFKILS
RTVTDRNRPLIYLDSAATSHKPQPVLDKLDEYYQYLNSNVHRGAHQLSREATAAYEASRDKVQAFVKAPSRNEIVFTSGATDA
INLVVQSYGRAFLQAGDEVLLTATEHHSNLVPWQMLAEEKGLVLKFVPVPDPETGGLDWNAFESLLSPRTRFVAFQHVSNVLAC
INPVSDMVAMIRNKSPDAKILLDACQSVPHMPVNVQQLGVDFLVASGHKMCGPTGIGFLWGKEDLLNSMPPWKGGGEMIDQVT
LEGSTWQPSPARFEAGTPAIAQAVGLGAAIDYLNEIGMDNIEAYEHEIGAYLYAQLSTVPGVTVLGPSVNRVALSAFAHTTVH
PSDLSTFLDVEGVAIRAGHHCCQPLHQALGYSHSARASLYFYNTKDDVDDFIRHLKTTIAFFENLESPSEEQVDDDFVPFV*

>jgi|Ectsil1|23950|rna8870
MVSAAAVDGVTSMKEEGDKDGLGLSLGERVRADFPILDQEAYPGKPLVYLDSAATSQKPKVVMDVLRDYYERDNANVHRGAHQ
LSIRATEAYEAAREKVGAFVNAETSREIVFTRGATEAINLVAQTWGLDNLAAGDEIVTTVMEHHSNMVPWQLLAERTGAVLKF
AQIREDMSLDLDHMKSLITDKTKLVAVVHASNALGGVNPVSEIAEAAHAVGAKVLVDGCQSVPNMPVDVQTLGVDWLVASGHK
MCGPTGIGFLWGRMSVLETMRPWQGGGEMIDQVYFDHSTFAEPPARFEAGTPAIAQAVGLGAACDYLSSIGMENGAAYEHEMS
KYLWERLSEVEGLDFYGPPPNASGDNRNPLLAFNSRDVHAHDLSFFMDQEGVAIRAGHHCTQALHRQLGAAGSLRASLYIYNT
KDDVDQFIEALRSTLDMFKAMDGDGGGAGGGLVGGEPSIF*

>jgi|Nanoce1779_2|581086|fgenesh1_kg.5_#_1192_#_TRINITY_DN7801_c17_g5_i1
MSEYYEHSNANVHRGAHALSIRATEAYEATRDKVQNFIHASTREEIVFTRGATEAINLVANTWGIQNLKAGDEILLTVMEHHS
NLVPWQLIAKKTGAVIKFVGLDEEEGLDLPQFHSLLSSKTKLVAFPHVSNTLGCILPVAEMAAAAHAVGALVLVDACQSIPHM
KVDVQALDVDFLAASSHKMCGPTGAGFLYGKLGVLKQMPPWHGGGEMIDEVFFEYSTFAEPPARFEAGTPPIAEVVGMGAACD
YLMEVGMEKVEEHEHQLGKYLYERLSEVEGVRIYGPPPGKEGKGRAALASFNTDQVHASDLAFFLDHEGVAIRSGHHCTQPLH
RMLHAKGSLRASLYVYNTKGDIDVFIARLKETMEFFGNFQSGGAGAATIL*

>jgi|Ochro1393_1_4|872714|fgenesh1_kg.13_#_1149_#_TRINITY_DN19211_c0_g1_i6
MNYGFILISAFLILLQGWSPYVLGFGQKSSPIVKFSRLSRTLAATVAVDEMNAIEREDFPILNQESHPGKPLIYLDSAASSQK
PTFVLDQMDAYYKTSHSNVHRGAHALAVRATDAYEAARQKVQTFINAQHREEVIFAKGATEAINTVALSWGQRLQAGDEIILT
VMEHHANLVPWQMLAQRTGAVLKFVQMTPSQQFDVDHFHSLLSPKTKMVAIAHASNVLGSINPVEEVIQSAHEVGAVVLLDAC
QSVPHMPVDVVHLDCDFLVASSHKMCGPTGIGFLYGKKALLESMPPVIGGGEMIDDVTLEGATYAMPPSRFEAGTPAIAEAVG
LGAACDYLSRIGMARIHEHESKLGAYLYDQLAKIDGLTLLGPSTTEPGAQRTGLVAFNSNTIHATDLSFFLDQEGVAVRTGHH
CAQPLHKQLGIAGSLRASIYFYNDKEDIDNFIVKLKETIHMFENMESIF*

>jgi|Auran1|11607
AEALRAEFPALHQRTNGRDLIYFDSGATSQKPARVLGALEKFYARDNANVHRGAHALATRATDAYEAARDKVAAFVGARRDEI
VWTRGATEAINLVAQAWGPSNLGAGDEIVLTELEHHSNLVPWQLLARRTGAKIRYARLDADGAGVDEAHLLAQINGRTKLVAL
VHVSNVLGSVAPVAAVVAAARTRGAPGCAVLLDACQSAPHAPLDVGALGVDFLAASGHKMCGPTGIGFLYGRREVLAAMDPWQ
GGGEMIADVFLDDGETTFADPPARFEAGTPPIAEAVGLGAAVDFLADVGMEHVAAHEAALGARLYDGLAAFGDRLELYGPTDA
ADRGAALVAFNARGVHAADLATFLDLEGVAVRAGHHCTQPLHARLGAPGGSVRASLALYNTADEVDAFLAALARVLDDFDGAG

>jgi|Vitbras1|1502|Vbra_6995.t1
MTVMQTTVVVPTSTALFCGLLISAAGAAFVGLVAAAPTSTTLGSPLRSDFPILDERIDGKPLVYFDNAASSHMPKPVLEAIER
YYQHQHSNVHRGAHTLALRATEAFESARSSVAAFLNARRREEIVFTSGATEGINLVAQTWGEANIGEGDEIILSVMEHHSNIV
PWQLLAKRKGATIKYIQMDENECLDMSSFESLLSPRTKMVGIVAVSNTLGCSNPIAEIVRQSHAVGAKVLVDACQAVPHKKID
VNALDCDWLVASGHKMMGPTGIGVLYGKYEVLESMPPWMGGGEMIEDVYFDHSTYLDPPSRFEAGTPPIAQAVALGAAVDYLN
KLGMDNIAEYEKDVSSYLYRRLGDIPDLRLYGPDPIAQGHSRAALVAFNHDNIHASDLSTFLDQEGIAVRAGHHCTQPLHRLL
GAAGSIRASLYVYNTRQEIDCFIDALHRVIQMFSDIPEAPGIDLGFAALGMEGLSLLTAGEMGPNDEGEGEVME

>jgi|Symmic1|19232|rna14008
MALLAQGLTLSPPPGLPLARRSNPRKTELASARGVQPTHAAAATTALVAGALTNRRTPGVGARVVSKAAKPGKSSWSAAEAEG
LRSDFPALQQEVKPGVPLVYLDNAATSQKPKQVLDELNRFYSQDNSNVHRGMHTLSMRSTEAFETARQKVAKFVNAPDSHEIV
FTRNATEAINLVARTWGAANIAKGDEIVLTVMEHHANLVPWQLLAESTGAVLRFGRLRDDGTLDVEQFESLIGDRTKLVALCH

VSNVLGCVNPVERVAKLAHSVGAKVLVDACQSVPHMPVDVQTLGADWLVASGHKMCGPTGIGFLWGRGEILRGSPPFLGGGEM
IDQVTLEGSTFNDIPHKFEAGTPAFAEAVALGAACEQYLTDLGMAAVSQRRRAGGEHELAKHLWSRLEALPGIHLYGPSPETQ
PDRASLVCFNVDGCHANDLATLVDQDRGVAMRSGHHCTQPLHGELGVTASARLSAYFYNTIEEIDVAVDALEVAIGLIRTGPA
GDEPELDPAMAALLDA*

>jgi|Symmic1|35087|rna24877
MNSISSRNVLCKVSFFRAGRSGWLAGGIERHRHGKALCLRFLRQLTACRSGKRAKAEAKAACPSHCLPNMACHQRTGPSFLSL
CVLPLRPGQLRPSGQGRPKDGQAKPSFQTAVLVAATAAVQKSRRLRAVAAPLEAPPAPSAEHQGVADRSLASKVRGDFPALST
EAYPGVPLIYLDSGATSQKPTCVLKALTEYYEHSANVHRGAYALAERATEAFEHARSSVATLIGAKGSQEIVFTSGATDAINL
VAESWGSRNLGPGDEVLITVMEHHSNIVPWQLATQRTGATLKHVGVTEEGILDMEEFHRLLSPRTKMVAFVHISNTLGCINPV
KEIAEAAHAVGAKVLLDACQSVPHLKIDVQDLGIDFLVASSHKMYGPTGVGFLWGRPEILEAMPPWKGGGEMIKEVHMDESFY
ADVPARFEAGTPPIAQAVGLGVACDYLLELGMDQVEAYEQFLARYLWESLSAIPGLRLYGPPASAGPRAALVAFNDTEPDIYP
QDVAGVLDVDGVAIRAGHHCAQPLHRALEATYGSARVSCAFYNTTEEIDKFVKALSEALETVRAGEGCVFDPDDPESCNCSSG
RMRS*

SUFE1 orthologs

>Cre06.g309717
MAMISQRQCGLRVASSLSSRSSPAVMPCPMRRQSLGRAMTGVCRAQKVSDLPASLKKIVGAFQMVPDPMARYKQLLFYATKLA
PMPDEDHIPANKVEGCVSQVWVVPEMRADGLIYWRADSDSQLTKGLAALLVTGLSGCTPAEILTVQPEFIEMLGLKQSLTPSR
NNGFLNMFRLMQRKTLELVAASGASASAAAAAAAPAGGESVPQAATAAATAATSSTNGAHASTPAPAPEAPAASAASTSRTPL
QDGMRRKLTEALKPSTLTIIDESAQHAGHAAMMVAKPGKAGGPGETHFKVEVVSEAFEGLTQVKRQRMVYQLLADEFNMGLHA
LALVTRTPAEASK

>jgi|Galsul1|5938|XM_005703315.1
MRMGHFDRMISHLDCLWVTSPVEANFYRLAYTRQGREPNCCFHNKKRLHFPKSTFRLQIRCQVDEKKLGL
TPELARLVKSFAAAPDPKLRVQQLLYLAQTLEPLPFQYKTNENKVPGCLSTVHVVGDCDNEKIFFKGDSD
AQLTKGLLALLIKGLNGCTVEEIERISPEFVTVAGLSVSLTPGRNNGFLNMLQTMKNKAKEAASKLNGVS
REGRNYSDRVPKTQASPSTAETASGPIYSAIVEKLQKLKPSRLEVHDDSFQHAGHVGAKGLRSSETHFSV
YVVSDAFVGLSLVKRHQLVYTLLGQELKEGLHALRIQAKTPSEVSDE*

>jgi|Cyapar1|2397|g2322.t1
MFQVALSQPLSVRSAAPSARGAICTASKPQRPKRAAAAAARSWLGSPAQLSWSACVAAHGPVRSVAPARV
PRVLAAGEVVAAGGMNLTPQLETIVESFQMVPDPKMRYRQLLFFAKELAGCPEEVKKEENKVIGCVSQVW
IVGSLDAEGNVSYVGDSDSELTKGLAALLIRGLSGNKPADILRVSPEFVKELGLNQSLTPSRNNGFLNML
KRMQRIAVELAAGDGGSSSGGAGAAAAAEPAPQAAQEEEQDDGTVYSAIRRKLTAALKPAKLVIHDESAQ
HAGHAGARGYNGESHFRVEVVSAEFEGKNAVARQRMVYQVLADEMRERIHALALSTKTPAEAA

>jgi|Caulen1|93255|g7875.t1
MQFRVSRLSSLYFPPRTFRKRHLIFHSLHSSRRTTIRPQAQQSVMDLPSSLKTIVTAFQTVPDPMARYKQ
LLFFASKLDPLPSEFHTEENRVQGCVSQVWVQATMTDGLVQFRADSDSQLTKGLAALLVQGLSGCSPQEI
LTIQPDFIEMLGLKQSLTPSRNNGFLNMFKLMQKRTLELAAIDLKSQESSGDSPSSNGLQEDGGDQSSSQ
TPVEDAIRYKLTQGLKPKELVIINESSQHAGHMGNPNNDPETHFKVEIISEQFTGANSVKRHRMVYALLK
DELAGPIHALTLSTKTPAEASE*

>jgi|Caulen1|90246|g5535.t1
MAFSMSKRISLNWCTRSFTGAQSISRSFKSTTLLTRRTTFKLDARESAVELPTSLKKIVTAFQMVPDPMA
RYKQLLYFASKLDPLVSEFHTEKNRVQGCVSQVWVTANLIDGLVQFRADSDSQLTKGLAALLVQGLSGCT
PREILSIQPDFIEMLQLKQSLTPSRNNGFLNMFKLMQKRTLELATADIRSQQSTGGDSSSNKGVSSNAGQ
PPSKTPIEDAIREKLTEGLNPDQLLIINESSKHAGHMGNPNNDPETHFKLEIISSEFEGANSVKRHRMVY
ALLQEELAGPIHALTLNTKTPAEVK*

>jgi|Tetstr1|426704|TSEL_016974.t1
MPAAARPVVGNAARCAAGTPAACQQLSQPHRIALSACAARTHLPLQHSVTRPLARCRRSFAMARPVRAAS
RRNSQIVCAQAGAELPPNLKKIVTSFQMVPDPKARYKQLLFFAAKLQPMAAEDHTDDNKVTGCVSQVWVK
PELREGKVFWAADSDSQLTKGLAALLVQGLSDCTPEEIVALDADFIEALGLKQSLTPSRNNGFLNMLRLM
QKKSLQLMASA*

>jgi|Chlat1|2784|Chrsp187S02951
MEVVSMSMPAAALVGTSSVARPPPLPGSHRHACSSSSSPSSPHPSCSLPSSSSASLCCFPLTSSLPSRRL
RRLTRPTRPAHSTTTTASQSVNELPAALQKIVRGFQMVPDPMQRYKQLLFYATKLKPLPAEDHNDGNKVT
GCVSQVWVVPRLEDGKVYFSADSDSQLTKGLAALLVEGLSGASPEEVLRITPDFITDLGLKQSLTPSRNN
GFLNMLKLMQKKTLELSMQVGASS*

>jgi|Mesovir1|1777|Mesvi1412S09175
MMTQEVEDLPEALQAIVRGFQMVPDPMERYKQLLFYASKLPPMPDEDHVEDNKVKGCVSQVWVKPRMEDG
KVYFTADSDSQLTKGLAALLVKGFSGAKPEEIVRVTPDFIQMLGLKQSLSPSRSNGFLNMLKLMQKKTVE
LYMATQKDAA*

```
>jgi|Praco1|14963|PRCOL_00000686-RA
MQRYKQLLFFATKLAPLDDAARTDDNKVKGCVSQVWVVARLDAATGTMLYEADSDSQLTKGLAALLVEGL
SGATPDEVAAVQPDFVEELGLKQSLTPSRNNGFLNMLALIQAKTAALAAGGATGAAAGTDAEPAASAATD
DTDAARPVYAAIVSKVSAALAPSRLELDDESASHSGHAGVNGLRGEETHFRMEVVSSAFEGLNRVKRQRK
VYELLADEFKGSLHALALTCRTPEEDAAAAN*

>jgi|MicpuC3v2|823|wlab.202019.1
MSSATATAAASALASRAAPHRGDHRRAVIATRRNAAAAKSRATTAAAAAETEKLPANLKKIVGAFQMVPD
PMQRYKQLLFFAAKLKGFDEKDRVEDNKVQGCVSQVWVVPRMGEDGLVYFTADSDSQLTKGLAALLCEGL
SGSAPKEIMAVEPDFVELLGLGQSLTPSRTNGFMNMLRLMQKKTLECYMEAEKAAKDA*

>jgi|Ostta4221_3|72643|OT_ostta02g01250T0
MSARLRTPWFRTDAGVARRRDVQERSRWRTRLTPRSIILVRAETGEISPKSLASVVSSFQAVPDPMQRYR
QLLFMAKTLTPVQAERLCDMYKVPGCVSQVWIIPSLKDGLVYYDAESDALLTKGLAALLIKALSGNSPEE
ISSVTPNFIADLGLKSALTPSRTNGLLNMLSLMQNQARSFL*

>jgi|Mesen1|4166|ME000219S03292
MATLVCQQSLAAITVQATRARLDYKEGFRSACFGSFPAAKCSRLAGSLLSSSASVSNNTSYLGLVYTTSN
ARKTHELSICCEQAIEELPEKLQKIVKSFQAVQDPRARYQQLLFYAKKLKPLAKEHQVSENKVTGCVSQV
WVLPSIDVEGRVHFQAESDSALTKGLAALLVEGLSGATPTEIVRITPDFVQMLGLKQSLTPSRNNGFLNM
LRLMQKKTLELFMQWQASQKSSASTNDTQDIVGEAETTLITVGSEKEGPAAGVPQDGSAGISTEPSEGSE
STSSSGSADDVVRDPRKPIYSGILFKLEKALEPVELVVDDVSHQHAGHAGVHRGATETHFNVKVVSPAFD
GLSTIKRHRLIYTLLDEELKAGLHALSLITKTPSELERV*

>jgi|Guith1|66468|e_gw1.13.229.1
MSVNEVKLTPELQKIVQQFSLVPDPKLRYQQLLFFAAKLGAMEEVHKVEENKVKGCQSTVYVHATKDEEG
KIWYTGDSDSQLTKGLCAMLVRGLSGNTVQDILEVSPEFVKEAGLSVSLTPSRNNGFLNMLNTMKAQAKA
LA*

>jgi|Emihu1|58720|gw1.152.59.1
LEKVVRGFKSVPDQKLRYQQLLFLAQKLAPMDASLAVDANKVPGCLSTVYVHGSLGDDDTINFVGESDSQ
LTKGIAALLINGLSGCTNEEIQAVDPSFIQAAGIAQSLTPGRNNGFLNMLKMMKGQAAKLA

>jgi|Pavlov2436_1|1033487|fgenesh1_kg.256_#_63_#_Locus3407v1rpkm48.27
MQLSLSWVLAVGWAGPQASQVLPAVSCARGPAHRVWMGQNPEQNPELGLTPYLEDVVETFKMVPDQKLRY
QQLLFLAQKAKPMDDSLKVPDNKVPGCLSTVFVHARPGEAGRIFYDGDSDAMLTKGLVVLLTEGLSGHTV
EEILAVDPKFIAEAGIASSLTPGRNNGLLNMLALMKKKAQQIASGSAVAAEFASVVAAQQSVVAGPVVEG
GSAPVADSIIRKVCEKLKPTRVKLVDNSAQHAGHKSREGLADGETHFALEVVSEVFAPLSMVQRHQLVYA
LLDAEMKGSVHALQIVARTPSE*

>jgi|Bigna1|92600|estExt_fgenesh1_pm.C_380005
MSTPYHTGASLLLLVVVASSLAALSPTRHLSTGLKPSMVLQRPQQTGVLQQVTTRKLEVSRGLNRAGGFS
RAMEQLSRRSSRQREMHAFATTSSSLNLTPNLEKEVVRLKSAPDNTLRHQQLLYYAKEAQPLPSQFQKAE
NKVPGCLSTVYVVAVKDEDGKVFFRGDSDALITKGLVNLLIRGLSGYSVEEIVAVKPEFIQEAGITQSLT
PGRNNGFLNMLNTMKKKAVQVTRE*

>jgi|Thaoce1|72048|rna32474
MKTMIAGIPVAGIALYSLASTTGGYGGVEAWSPSRQSSSTSRRTASSSTVTRPRRGDTTATALHSSTEID
TDALNLTPELEMMTGAFSSIADEKTRHKQLLYMANQLPTVDDSIRIPENKVPGCLSTVHVDCTTEEKDGD
TLVNYVGDSDGLLTKGLLALLIRGLSGCTAEQIAAVDPQFIQAAKISQTLTPGRNNGFLNMLAVMKKKAS
DAVEGGDESPASGDAAGAENSGEVVTSFDEVEGKPMYNAILSTLVPILKPVSIELVDNSSQHAGHGGAKG
WEESGESHFALDVVADVFDGLPLVKRHQLIYMLLGEVMPKIHALEIRAKSPGEI*

>jgi|Phatr2|7639|gw1.5.347.1
DDKLRYKQLLYMANQLEPIPESSQIPENKVPGCLSTVFVDGTAKYSDNGQDVLIYFRGDSDGLLTKGLVA
LLVRGLSGNTAESIQKIDPAFIQKAGIAASLTPGRNNGFLNMLATMKRKATQLA

>jgi|Thaps3|36605|e_gw1.11a.172.1
LTMMTNAFASIPDEKTRHKQLLYMASKLPDVGDEVRVKENKVPGCLSTVFVDCVLEKNDEDGEYVVNYFG
DSDGLLTKGLLALLIRGLSGCTPQQINEVDAQFIQAAKISQTLTPGRNNGFLNMLAVMKRKA

>jgi|Nanoce1779_2|577053|fgenesh1_kg.3_#_1240_#_TRINITY_DN5402_c0_g1_i1
MRPLSPVPLQLGLVLLIIVALARVTQGFVVVVPRQQVGRPSCRQTVVSMSSTEIDESSLGLTPRLLEVSR
AFQLMDERTRVQHLLAMAGALKPFKDFRKLPENKVPGCLSTVYVHAYLKEGKVYFEGDSDAQLTKGLVAL
LIEGMSGCESHEIQAVKPEFIKFAGIAASLTPGRNNGFLNMLAMLKRKAVEAEAKGEGTGQEEPEIEFEE
IITRPVYSSTMKKLINFLKPKQLILTDDSAQHAGHAGAKGLDGESHFSLKIVADCFEGLSLVQRHKLIYT
VLTAEMQQIHALSIDAKTPAEAGL*
```

```
>jgi|Ochro1393_1_4|727987|e_gw1.239.22.1
MSVKEEEADDFNLTPVLKTYANGLRNVVDDKLRYQQLLFLAAKCPEMDASLKLDSNKVPGCLSVVHVHAD
LDSEGKISYKGDSDSQLTKGLVTLLVKGLSGHTPEEIEAVDPKFIQYAGVAKSLTPGRNNGFLNMLNLMK
VKAKQLADGDVDGDANDSPGSIATPTVTASETTQGPVYSSIQTKLSMLKPSLLEVEDESYKHAGHAGIAG
VTAGSGSGETHFNVKIVAECFDGLSRVQRHQMVYALLAQEMKNGVHALSINAKTPSEV*

>jgi|Pelago2097_1|314629|CE314628_4120
MLSAFVALVLQAGLCAGFSPGSHARRRPSRTGAAPRAAAASLDALPAPLRAIVESLASLPDDKYRYKQLL
FMAGDCDPIADALKTPENKVPGCLSTVHVAATLDDGKVYYTGDSDGQLTKGLLTMLVKGLSGATPAQIGE
VKPDFIRDAGIAASLTPGRNNGFVNMLAVMQKKAQALLDAPAADDAGGQGKLAAAMLEILACLKPEALEL
VDESGGAETDFKLTIVTASFDGFEVEMRQRVIETLLADLIPRCESFRILALTPEER*

>jgi|Vitbras1|825|Vbra_4677.t1
MVISRLAVAVAWQLPSGQHQPRRPALPSSLVHRQQPSFRRSSTVLRESVGDGGEAAGDLPPKLRTLIATF
RRMPDDRFRYQQLLVFAKGLKPMNNAYKTKENKVKGCLSVVHVHAEGREDDSGDMRIYFQGDSDGLLTKG
LVNFLVQGLSGCTPRQIESVPAAAVMEARVTNSLTPGRNNGFLNMLETMKAKARDIAAVQGRA

>jgi|Symmic1|13793|rna10871
MNCTFARCLYCEKLAPTHPHPPPPGIFALSPAMLVVSATPKAPPAPTLSRASRDSRCPETSWASGVRAGA
GLVSLLPAMPAELKLPENKVPGCLSTVHVSARLEEDGTVSFQGDSDALISKGLCALLVLCLSGCTPEEIA
GVKPEFIKASGISAALTPGRNNGFFNMPRPKSVEYRLSQVESALLSN*
```

SUFE3 orthologs

```
>Cre06.g251450 SUFE3
MRLFANTRGCASATHERSRCILVGSTRFIGGGAPRVAHQSSLEQSKPCLISRRGRPGFQLAAVQASAAGDTAQVATPSTGAPG
AEWLPASLEAQLAPITSAPDAKEALRRLLDAGSGLAPLPAAARTASNRVMGCTAQVWLAAETDAAGRMAFQGWSDSEVSRGLV
ALLVRGLSGCTPEEVMQVSASQVQQRLSRVLGRSVLPPGRANGLGNMLESARKRAALAAAAAAGRRLDVFPSLLITADALTPQ
GAFAEAQARYLAPDAAAVSQLVRVCRDKHIGVVAHFYMDPQVQGVLSAAAEEWPHIAISDSLVMADTAVRMAEAGCTTICVLG
VDFMSENVRAILDEAGHSAVQVYRLAESDIGCSLAEAAESDSYSRYLQQAAHTPNSVHVVYINTSLRTKARAHALVPTITCTS
SNVVQTVLAAFADVPGATVWYGPDTYMGANLAQLFADLASGAASDDDVRALHPAHTVDSIRSLLPRLRYFTDGTCIVHHIFGG
EVTELVAAGYGDAYLAAHFEVPGEMFRLAMQAKRSRGMGVVGSTSNILDFIADKLREALSAPHPERLQFVLGTEAGMITSIVR
KVQGLLRQSGRTDVEVEVVFPVAPSAVATPQQRPQEGAAPLTLPTGLALVPGPASGEGCSLEGGCAACPYMKMNTLAALVSVC
ERVGSPAGEASLERYRPRTYGGETVGGRSLAAAGCVPILHMRNFQRSQGRRLGPDLLQDIASRHTAR*

>XP_005850636.1 hypothetical protein CHLNCDRAFT_19680, partial [Chlorella variabilis]
LERLATELLAAAGPQERSRLLLGYARRLAPLPDAARTDANRVMGCTAQVWVSAELDGEGRLRLMADSDSELTRGLAALLVEGL
SGLTLEELLQVDSAVLGQLGLGAAVLTRSRANGFLNMLESLKRRARMLLGDLPRFPSLLIGAERTSAQGAFAEAQNAFLRPDG
AVVDRLVEQLAAKKVGVVAHFYMDPEVQGVLSSAAERWPHINISDSLVMADGAVKMAEAGCTAIAVLGVDFMSENVRAILDEA
GHADVAVYRMSADSIGCSLAEAAESPAYDAYLAEAGDTPNSLHVVYINTSLKTKALAHSVVPTITCTSSNVVQTVLTAFAQVP
DVHVWYGPDTYMGRNLAQLFQSLANLSDEEVRELHPAHTQASIHALLPRLRYFEQGTCIVHHLFGGEVCELVKEGYRDAYLTA
HFEVPGEMFSLAMDAKRQRGMGVVGSTQNILDFIAAKLGAALEQPFPNRLQFVLGTESGMITSIVRKVQGMLRAAGRDDVEVE
VVFPVSPEAITTDRQQQQVRAGLPTGLSVVPGPAGGEGCSLQGGCASCPYMKMNSLQALMTVCQRVGSPAGEALLEAFKPRPY
TELVDGKTMAQAGCVPILHMRGFQKGGKLPEALVADITGRHSA

>QDZ26067.1 quinolinate synthase [Chloropicon primus]
MSQRLGPLSRSSGSCGRRKRRTGFVSAVYKAEAPVEEAGGTGFVENLHVELIADLGVKDDSDTGALLKRLIELGSSSPEFVLP
QHELTEEKRVPGCASRVWIDCEVREEDSLVHFRGYSDSAISKGMCNLLCRNFSGKLTPESFLSIDESFLKEVTRGLGPVLSSS
HRRHGLFSIFDSMRKRVHQLVSGGIDEPFPSLVITKEDLIPQGKYAESQATYLKPDQSKVDDLVRSLKETKCGVVAHFYMDPE
VQGVLAAAQKDWPHIHISDSLVMADRAVDMAAEGGCENICVLGVDFMSENVRATLDHQGYNDVNVYRMDVDLIGCTLAEAAES
ERYERYLEEAARATENALHVVYINTSLKTKAVAQSKVPTITCTSSNVVQTILQSSSQLDGDLSVFYGPDAYMGANIKEMLEQL
CGTSDEEVRKIHKDHDISSIKNLLSRFRYYQDGICLVHDMFGSEVAQTIRESYADAFCTAHFEVPGEMFKLALHASHNGDRGV
IGSTKNILDFILGKVAEANEHNLESERLQFILGTESGMITSVVNAVQESLREAGSNVEVEIVFPVNSSAITSASQANAPAMPF
GLDIVPGPAGGEGCSMEGGCASCPYMKINSLDALMFVLSKLEEDDSDPILQDYAPRNYEESIDGKSFAQVGTVPILHMRHFTQ
HKCMSDDLVADIKARGLQ

>XP_003064062.1 predicted protein [Micromonas pusilla CCMP1545]
MSSAPIAGLRARVVAKRVARRNVDRAIRAVSTERSAANNLGNEKLRSVAGAVAAEKDVSEKLKALIARGGDLPALPESDRAPS
NRVMGCTSQAWIDVSLDADGKVLLRGHSDAQITTGFAGVLAAGLGGLSPEDVLGVDDDVVKDLGLGASALPRSRANGFRNMLE
SVKKQTRLLRDGRDAMPFPSLLVTADGIEARGPFAEAQANYLEPDAEAVAALVKELKAKKIGVVAHFYMDPQVQGVLMAAAKE
YEHVFISDSLVMADAAVKMVEAGCDAVCVLGVDFMSENVRAILDDAGHADAKVYRMAAEDIGCSLAEAAQSDAYYEYLAAADG
VENAVHVIYINTALDTKANADAIVPTVTCTSSNVVQTVLQAAAQVPGVNIFYGPDTYMGGNLAELFTMMSTWTDEEVAAVHPA
HSRATIKALLPRLRYFQDGTCMVHHMFGGDVCDTVRKFYGDAYQTAHFEVPGEMFKLAMEAKRERDMGVVGSTKNILDFVVDR
VDDAIARKHPNGERLSFVLGTETGMVTSIVRAAQARLKKAKADGVPDVECEIVFPVSADAVTATGETQAPSIGGLAVVPGPAG
GEGCSSEGGCASCPYMKMNSLDALMRVCARVGTEGGAATLAPMEPKKYAAAKEGEKSVAARGCVPILHMRDFTRDKKFSDAFV
NDIYGDLNLIITYHITRARASRRAFPPPPSRASLAHALAAMLRSSKSIVSVAFGGISCT
```

```
>jgi|Klenit1|14322|rna-KFL_008730030
MEVATRSAASFPHLLPDGQVLHGQGQARGKPARPAALASSPSPLKIPRRGPLFCRCGKELPRSDFFGTSW
HPDASSQRPQSTRPAFARAEGQAGREGGRKQRGSIGATARERRQVRAAVSVVQDVVTETGGWGLEEVRRR
FEALADGQSKYRLLLQYAAGLPRLGEGDKTADNRVMGCTAQVYVTVCLDDAGLVQLGGDSDSELSRGLVA
VLVAALGGRPPAEVLAVQEDALAGLQLGSAVTSRSRINGFYNMLQSIQRRTRAAVAAAAGEAPPAPFPSL
LITAGGEAVEAQGEFARAQAEYLSPDPAAVTELVNLLREKKIGVVAHFYMDPQVQGVLAAAARQWPHIHI
SDSLVMADRAVSMAAAGCERIAVLGVDFMSENVRAILSRGGYPEVPVYRMAAEHIGCSLAEAAESPAYVD
FLEEAAGTPGSLHVIYINTSLGTKAEAHARVPTITCTSSNVVQTVLQAFAQVPECSVYYGPDTYMGGNLG
TLLRQLATQSDEDVAKVHPAHTQASIRALLPRLHYFQDGACMVHDMFGQAVTQRVRASYGDAYQAAHFEV
PGEMFDLALEAAARGMGIVGSTKNILDFIAERTQAALAAGYAQRLQFVLGTEAGMVTAIVNKVKGILKEA
RAAGNDTPVEVEIVFPVSADAVTATGENGSGGSEALAGLTIVPGVAAGEGCSTAGGCASCPYMKMNSLDA
LLMVCRRIGTPGEALLDAFQPRMVDRTVAGKAAVDLGSDSILHMRHFQTNGKLSDELVADIRTRNQHGRQ
GPL*

>jgi|Chabra1|329227|rna-CBR_g20372
MEAQVVAGAVRMESAWATGIVVGQEGEGRRGRNGCCCSAQAASSSLSMSLCHSSRLQRRSGSLRFSCHGS
SDSGCSHRWESAHVSYGRSRQSSPLSRSDPWGCGSRWVSKSQICGCFSLRVRRKMEGKVREGVVRCALAD
AVVLRHVGEEVILQKIAEEFSRCEDNKDKMRLLLHYAERLPRLDKEDCIPENQVMGCTARVWVTARLDDE
GWVWFSGDSDAELSRGLCAVLVQGLSGLTPEAILDVREETLTSMHLSPIATRSRINGFYNMLKMMQKRAG
ELALLRDGLTPVAPFPSILISANGELQPRGDFAQSQARFLSPDPHAVDTLVSLISSKKIGVVAHFYMDPE
VQGVLAAARSRWPHILISDSLVMADGAVKMAEAGCTTIAVLGVDFMSENVRAILDQAGFSHVGVYRMSNL
EIGCSLATAAQGPSYVQFLDEASKTPNSLHVIYINTSLETKAEAQLKVPTITCTSSNVVQTILQASAQVP
EVSVWYGPDTYMGANLAFVLREMANLSDEEIAAIHPAHNRESIGALLTRFHYFQDGACIVHDIFGSEVVK
RVKAGYSDAYQTAHFEVPGEMFALAMEAKRRQMGVVGSTSNILSFIADKTQEAIARGHDQRLQFVLGTES
GMITSIVGKVREILKSNMEAAGARGPPPQVDVEIVFPVSSDAITASSEATGGNLSSPSLSAPAAGQGLDQ
LKGAVKPASGVLGELGVVPGVQGGEGCSISGGCASCPYMKMNSLNALLRVCQWIGTDSEVMLSAYKPRLV
RDQVGGRSLVDLGCTPILHMRHFQKTGSFADDFVDDIRARKYLSS*

>CBJ28535.1 conserved unknown protein [Ectocarpus siliculosus]
MFGTKAAVLRGARLIGRRSLHRRQPPDAPAAAAAVCAGPGVSQRERGAAHYSRRVAGLSTAAGLKVATAARQVQHGGERFPSL
LITADDITAEGSFAETQATFMTPDPSLVSELDHLLPTKNMGVVAHYYMDAELQGALAALKWEHVSVADSLAMGDAAVRMAEAG
VSSVACLGVDFMAESVRATLDSRGFQGVPVYRLSESEIGCSLAEAAERKAYEAWLRKASATANSMHVVYINTSLVTKALSHSL
VPTITCTSSNVLHTVLQAFAQVPDLTIWYGPDTYMGENLHNMLTELAGMTDGQIREVHPKHDQASVRSLLPRFHYFRQGNCIV
HHMFGDDVVERVRSNHADAFHTAHLEVPGEMFRLAMEAQNKGRGVVGSTSNILGFIKDQTRGAVARAGGAGGAGEEAAGEKLS
FVLGTESGMITSIVRAVQEELRAHNNKTQVEIVFPVASEAMTATGESGDGSMQVVPGVSGGEGCSTAGGCATCPFMKMNDLHS
LLDVVGDANESGAVPDVLKGFLPKRRAFTIDGQDKTELGVVPIMHMRDLMKDGALSDDLVSDVQSRCG

>jgi|Pico_ML_1|52398|g3107.t1
MRAQVRASISRDRGIGTPAKGKVETQEQLQKLLARGARAIAWPEERRKAENRVLGCTTRTWLEARRCRGG
KGVEFEVDSDSQLTRGVAAIACEALRGKDAQQVARIDVQEFGKEIGLSQAFLAPSHANGFQNLVNAMKKR
AAVLQAQDEADGADVPEAVRFPSLRITADDVEAEGSFAAAQAQFLRPDLEQVKELVHVIQVKRIGVVAHF
YMDPEVQGVLSEAALDWPHICISDSLLMADKAVAMVEEGGCKSICVLGVDFMSENVRAILDEAGHKDVQV
YRMASDAIGCSLAEAAESLSYETYLKEASKTPNSVHVVYINTSLRTKARAHGLVPTITCTSSNVVQTVLT
AYAQIPGVTVWYGPDTYMGANIEKLLTDLSEMDDAAIQELHPDHNRETIKSILPRFKYFKDGSCIVHDLF
GGEVCDTVRTYYRDTYLTAHFEVPGEMFILAMEAKQRGMGVVGSTQNILDFICHRLDEAIHDGETDETLR
FVLGTESGMITSIVRHVQAKLKPVVASHPGLAVEVIFPVSTDAISRPEEQPAGLGAVPGTLLRAGAGARA
VRRTKQTTRDACTKNEEGTGNVIIPKKDYSEHILKIQRKKTP*

>jgi|Caulen1|90988|g6234.t1
MMTRYNFASRPIASKSPCVQTQFNRQRALRSPQSLHVSRPRTRYVSNVVCQVTINNPAVASVLTELRDSS
SATEKLKVLLHRGRQLIEFPESNQTIENRVMGCTTKTYLTVELVDGLVEFAGTSDSELTKGFIWILTQLL
NARPVDEVLQFDVSQISEILSMSEIMTPSRTNGFLNLIETLKRKTRMLVRQFPKFPSLIIRSDKLEPVGA
FAEAQAQYLNPNAGAVEELADLLREKKVGVVAHFYMDPEVQGLLTMASDLWPNIVISDSVAMSEHAVKMV
REGGCEHIAVLGVDFMSENVRIVLDRAGFDQIPVYTMSKDHIGCSLAEAADSIAYEDYLRNSMKLTSGPK
LHLCYINTSLKTKAISQSIVPTISCTSGNVMKTLLQSYAQIEDLTVLYGPDTYMGENIRDMFTSIADMSD
EEIKQIHPGHDKESIRSLLKRFEFFQEGTCVVHHLFGGEVCKIVREAYGDAYIAAHFEVPGEMFALAMEA
RRRGMGAIGSTRDIHDFISRIVEAQVNERTTDRLQFILGTESGMITSIVRSVQSIIRKNSTLEDLEVEIV
FPVASSAITTQDQQTEKVISLPDEMVLVPGAAAGEGCSAEGGCANCPYMKMNTLTALKQIVERIGQGGGL
EGFYPQEMDSVVQVNGREESIHDLGFLPVYEMRNFQENGVLSDELVSSVKATT*

>jgi|Tetstr1|432101|TSEL_021572.t1
MAALTRAPLRRPAAAGLHRHVAAGSVPIPPAAGRSTQPRRLREHFRSNLVAAASASSVQVAQLPTAALQE
IAAELDAAADPKQRIKTLVAYGAQLEGLPEEAKVMRNRVMGCTAQVWLTAKLAADGTVQLAADSDSDVTR
GLCAVLVKGLHGLKPQELAEVSTDALLALPLGPAVMAPSRTNGFLNMLETARKLARGLMGEMETFPSLLL
KANSISAQGPFAESQAQYLSPNPEAVDRVAELLSSKKVGVVAHFYMDPQVQGVLSSAAERWPHIHISDSL
VMADTAVRQPARPVPWAPTISTTILTSIITTTTTTTTVITIITTTVANTITTLIIITITATVATTIM

>jgi|Praco1|16967|PRCOL_00006555-RA
```

MARCAPARPAAAAAAVPARGRARPRRAAARAAGGGGGSVAERWEAVAARIAAAGDAKARLALLLERGAAA
PRMADAARTAAANVPGCAARTWVAAHPLDGDASGVRFEADSDSALTRGLAAYFADALEGLPPREVAALDA
GAALRALKLGDAVAANSRGNNLRTLLEAFARRAKELDAEERERGAGAGATAARARAAPFPSLVLRAGAPA
APRGAFALAQAEYLRPDAARARAMASVLSAKRIGVVAHYYMDPEVQGVLSAARDEWPFVHISDSLLMADQ
AVVMVEEGGCESIAVLGVDFMAENVRAVLDRAGHTSVPVYRMDPGDIGCSLADAARGPEYDEYLGAAAAQ
GRSVHVVYINTSLATKAAANATVPTITCTSSNVVRTVLQAFAQVEGVHVWYGPDSYMGANLVELLESLLE
LPEEEVARVHPAHTHASVRDALSRLHYFEDGMCVVHDMFGEDVVRTLEEHYVGERDTFLTAHFEVPGGLF
GLALKERAAGDSVVGSTAEILAFIKAKTADAVEANEDRALRFVLGTEVGMVTSIVLAVQDMLDGARERLG
GDAADVSVEIVFPVSSDAVQGVDAAGAITKSKAGGSSPSLLGLEVVPGAAGGEGCSAEGGCASCPYMKMN
SLDALERVVGAIGTDAEAMLEAHKPRVMFEDASEMDRGVEPIIYMREFTKTQRFPDDLAERIEGSVGAVA
ANAT*

>jgi|Chlat1|7489|Chrsp60S00549
MAAAAMETAALSGAVRGTWVGRSVEIAADEAKQRKKSISVGGKKKPPPQQRVPAVAVASSSGRGGSRRAA
TVSVATVKEGTRIPKDAAAAVAEVVALGRSTPAWSDQERAEAWRVAGCAARTWVRCSVDRATGGIDVRAD
SDSDIIRGVGTLLSKACQGRTPVEVASMSAPELLSLLGPLPPQVLSPSPRSGGARALADAILRQARAIRD
GGDVGTRFPSLVIEADKLSPVGEFAEAQSRYLSPDPAQVQQLASLLASKRTGVVAHFYMGPEVQGVLSSA
REVYPHIHVSDSLVMADSALRMVEEGGCQAICVLGVDFMAENVRAILDRAGHADIKVYRMSNDVIACSLA
DAARTNNYIEWLQHAKDNVSGPHLHVVYINTALDTKAHAHSIMPTITCTSSNVVQTILTAFKQIPNLHVW
YGPDSYMGANIKDLLTRLASESGVEGEKYDLLGHDATSLKSLLERYDYYKEGVCIVHDMFGEEVVKRVRS
AYGDAYQAAHFEVPGGMFSLAMEARARGMGVVGSTSNILDFITEKTRDALERDFDDKLTFVLGTESGMLT
AIVRRVQSLLQQAKLSHPSSPSIQVEIVFPVNSDSITTTSATSPQLSFQGLSIIPGPAGGEGCSSEGGCA
SCPYMKMNTLEALIKVVGRIGTPGEGMLSGYVPEVYGSVGEGGEGGSVADKGCEPIVHMRGFQKSGKLTE
ELVEDILARATP*

>jgi|Mesvir1|12745|Mv20420-RA.1
MTSGLAATYVANAVLPSFVQKVGGCTGARGATVKGEVARKSGFAGRHDTCRLLKSAHQASRQSSAYQTLC
ASPSGAELSRADEQMAMPSSMAKIGKEFQAVEDMQARMKMLIERGSSCPPLLDHERVNENRVMGCSARAW
VAASLDSQGRLHYRADSDSAITRGMCAVLCEALSGMTCEEVIRTDPATVRTLNIPTGVVGLSRTAGLHNI
FESMRRAARKLKEGAAGLAPFPSLLVSAHSIKAQGTFAEAQRAYLQPNPEAVTRLAQLLEKKKISVVAHF
YMDPQVQGVLSSAAELWPHILISDSLLMADRAVRMVADSGCQRVVVLGVDFMSENVRAILDKSGFADVPV
YRMSADEIGCSLADAAISDSYFKYLGQAALTPRSLHVVYINTSLETKARAHALVPTITCTSSNVVQTVLQ
AFAQIPDCTVWYGPDTYMGGNLCEMFKRLAVLSDEEIQAIHPAHNRSTLKALLPRLHYYQEGMCVVHDLF
GEEVVKTTRELYGDAFQAAHFEVPGEMFALAMEAKSRNMGVVGSTANILDFITARTTEAIERDLPDERLQ
FVLGTESGMVTSIVRAVQALLREAEKAREGKPCELEVEIVFPVSSDAISRSPDSSAGGGSKGTVTAFGGQ
LAVVPGVQQGEGCSISGGCASCPYMKMNSLEALMKVCERIGTPGESLLSSYEPRKYQEGLGSRSLADVGV
DPILHMRYFQQNKKFSDELVQDVLTRYTA*

>jgi|Mesen1|9282|ME000060S08723
MFLENGKAFAQVPDLHVWYGPDTYMGGNLVDLLNRLAEQSDEEVQAIHPGHTAASIAALLPRLHYFQEGT
CIVHDLFGSEVVERVRKGYNDAYLTAHFEVPGEMFALAMDARVHGMGTVGSTQTILDFILARTREALARG
FDERLKFVLGTESGMITSIVNAVRKELAAARQAGSTAEVEVEVVFPVSSSAIATTNQEGIASSSLGGGAG
AALSELPIVPGVQGGEGCSVSGGCASCPYMKMNTLDALVRVAKLAGSGGEPLLNAYAPRAFSHQVAGRTV
ADIGCDPILHMRHFQIRSSIEFAEEQVGMVSAQVVEMTLIEKTRSSRYLSAVSVEDKGTGKVEGGVSGAK
GQQSRLEASLYFDGYALLKETSSPTALQTALQTALQTALQSEQNSFSATGSGSSSGSGSGGRGDADSWKE
GVMGVLEQHLGSCSSEGWELEGGMVEQAPGHGCSDEASCPSPWWSASWHVEGFLFREDGGVLLPQQALTV
VVAASNLLGASSGGLAVYPRSHWLVASWLQAALKGEAATSMPPPSALPASAPLQVAMAAGDVLLMHPWLA
SAPAPNASVHPAWRLHARLRPNRDSGCGDDSSASSALLPKAASADDVVRAMWRHRAWPAAAQAPRGPPPV
RGDQVAALEALQDEAAKAQAGNWAVPSLPPLPFSSSSAAAAAAAAAATAEGEELKLPFPCAYASPADVRA
WSLSVEARRFLAEDSWQEALVVLRQLAGLRPLSFSIFYEAGRCSVRQAPRGQLAGRAGRKCLLEDGERFL
TRAIELAPGWPPAYAELALTLMAMGRGREVEVGPLIASLLQVPRLPELCRQHPEAMAAMRQLLQCAQEVA
AQRRDDFQFFEWDLLEGLAGTGEHPSPDLVT*

>jgi|Phatr2|14735|e_gw1.16.187.1
GSFAVAQATYLQPDAEAVQDLVDLLERTQSGIVAHYYMDVELQGVLQAAGARLPGRVGIADSLKMGDMAV
DMAANHNCTAIACLGVDFMSESVQAILQQNGHGHIPVLRATHKPIGCSLAESAERESYRVWLEQESVGQA
ALHVVYINTSLETKAVSQSIVPTITCTSSNVLQTLLQASAQMGPELKILYGPDTYMGENLVTLLSTIEAR
WNDEQIASLHASHSRDTI

SUFB orthologs

>jgi|Galsul1|3981|XM_005704982.1 SufBC fusion
MNQSLDATTLKLEQIINKSYKYGFSTKIQNEDFPKGINEEIVTLISKKKNEPKYMLDFRLKAYKKWKQMYVPKWQYLKFEQIN
YQNILYYSVPKQQKKLQSLNEVDPELLKTFEKLGIPLNEQKRLTNVAVDAVFDSVSIGTTFKQELADQGIIFCSISEAIQKYP
SLIQKYLGSVVPIGDNYFAALNSAVFTDGSFCYIPPDTICPLELSTYFRINNEESGQFERTLIIADRNSYVSYLEGCTAPQYK
KNQLHAAVVEIIALENSEVKYSTVQNWYSGDENGNGGIYNFVTKRGLCAERNSKISWTQVETGSAITWKYPSCILVGNYSKGE
FYSVALTNHYQQADTGSKMIHIGKNSKSKIISKGISTGKSINSYRGKVKISSNASKARNYSQCDSLLIGNSSEANTFPYIEVH
NRSSIIEHEASISKINEEKLFYFMQRGISIEEAISLIVSGFCKEVFTELPLEFALEADKLLGLKLEGTNTEEYILNGINLNVK

```
QGEIHAIMGPNGSGEIIFQEQNLLDYTIEDRANLGIFLAFQYPLEISGVNNIDFLRLAYNSKLKFNQTSTVDPLKFLEIVYPK
LKLVGLDESFLHRKVNEGFSGGEKKKNEILQMALLDAKLAILDETDSGLDIDALKDISNAIKSILKISQFKQSIIIITHYQRI
LNYIQPDYIHVMYKGKIIKTGDASLASELESKGYEWITSE*

>Cre15.g643600 SUFB/NAP1
MLPCGTRTCSGASTSTRTQRAAAPGVPIAARSLRRGPTTCRTATVEEAQISDEKRDIQKILDRPYKYGFKTIIESDTFPKGLN
EDVVRAISAKKGEPEWMLEFRLKAFRKWLTMEEPKWSDNAYPEIDYQDVSYYSEPKFKEKLESLDQVDPELLKTFEKLGIPLN
EQKRLSNVAVDAVFDSVSIATTFKADLLKHGVIFCSISEALKDYPEMVRKYMGSVVPVGDNYFAALNSAVFSDGSFVFVPKGV
KCPMELSTYFRINASETGQFERTLIVAEEGAYVSYLEGCTAPAYDNNQLHAAVVELYCDKDAEIKYSTVQNWYAGDVNGKGGI
YNFVTKRGLCSGSHSKISWTQVETGSSITWKYPSVVLAGDNSVGEFYSVALTNNRQQADTGTKMIHVGRNTRSRIVSKGISAG
NSRNCYRGLVQVQPSARGARNFSQCDSMLIGDNAAANTYPYIQVREPSAVVEHEASTSKISEDQLFYFQQRGIDPEKAVGAII
SGFCREVFNELPLEFAAEVNELMSLKLEGTVG*

>jgi|Caulen1|89441|g4767.t1
MWNWNNIENLCSTGHFAWKGVECDGSGSVIGLDLQELSLQGSITGKLALLDSLVTLRLRGNLFTGQVPDLTELSYLKILDLSS
NELKGEFPFSAHAFWATPAGVSLEEIYVQGNKFSGYFVDDDAVSIEESSIRIISASQNHYHGKLPESWFRLVHLEQMDLSNNR
GLCGEIPSFQNGVSIPEDWLSSQICEIPKSSAQEEATLLLSLKNELAPQSSVEQVLKNWRPEKSEELCESWERVECEEGRVFL
TSTSIGVPCDENLEVSWMATEVLPTNNDQVTQITTSLVVPEVAQAPQEFLNVSQDSEDSPMDAMQSSPAYTETENSAIVPSPE
PELEISGFDGRKLPLTVSTTDSSNGVDSGEDSFEEKKGLSQHAIIWTIVSFLLGILLVILIFLCIHHRNQRDPNKDSKESRTW
KMRYSQFMSSLSGLIANTKTKSGSGKEGSSNGSKKPKEDVESPPTTSSVLASDHDSETNKNVSELGKLASIDSFAPLRRMSFS
FSRKNKAVQLQNTTADRVQDGTTDMALFGIQTDDSRSRLTLDTNDSQQNTYDNPLSSNDEPMATNLDLNDEVFNDCGVMFKNR
AFSEQTLNSEPSVTHTGLQRNYNDFHSIDEEPIEGAKSNHPPERLKTRQPDFVVNMGRCLLTQCSRGYFGDLGTLTQTTSDTM
GLRPHSTDATELNLNSKSTTWSITEDLGGTRTTISEPIMITRELTGSGNDVEDTKSIQIVSGRRIRRPFHTVCQSSTGVADSI
GDKSSAEKADLQKLLNKPYKWGFKTIIESETFPKGLSEDVVRAISVKKEEPEWLTDFRLKAYKRWLTMKEPDWSDNTYPRINY
QDLSYYSEPKQKEKKQSLDEVDPELLATFDKLGIPLNEQKRLTNVAVDAVFDSVSIATTFREELSKVGVVFCSFSEAVKEYPE
LVRKYLGSVVPVNDNYFAALNSAVFSDGSFVYIPKGVHCPMELSTYFRINASETGQFERTLIVAEEGSYVSYLEGCTAPAYDT
NQLHAAVVELFACEDSEIKYSTVQNWYAGDEEGKGGIYNFVTKRGLCHGNNSKISWTQVETGSAITWKYPSCVLRGDNSVGEF
YSVALTNNAQQADTGTKMVHVGKNTRSRIVSKGISAGRSRNAYRGLVKIAPTAKNARNYSQCDSMLLGDNAGANTYPYIQVRD
PSAQVEHEASTSKIGEDQLFYFQQRGIDLEEAVGMIISGFCREVFNELPLEFAAEVNQLMSLKLEGSVG*

>jgi|ChlNC64A_1|29265|estExt_Genewise1.C_10416
MLAKPYKYGFKTIIETEQFPKGLSEDVVRAISAKKEEPEWMLDFRLKAYRKWLTMEEPNWSDNRYPRIDYQDLSYYSAPKVVD
KKTSLDEVDPELLATFDKLGIPLNEQKRLANVAVDAVFDSVSIATTFKEELGKAGVIFCSISEAVKEYPDLVRKYLGSVVPVA
DNYFAALNAAVFSDGSFVYIPKGVRSPMELSTYFRINASETGQFERTLIVAEEGAYVSYLEGCTAPAYDENQLHAAVVELSAA
KDAEIKYSTVQNWWGYAGDAEGRGGIYNFVTKRGICLGERSKISWTQVETGSAITWKYPSVVLKGDHSVGEFYSVALTNNRQQ
ADTGTKMIHVGKGTRSRIVSKGISAGHSVNAYRGLVQVQPTAAGARNYSQCDSMLIGDQAGANTYPYIQAGGGAAAGSGQGAR
VEHEASTSKIGEDQLFYFLQRGIDAEDAVGMIISGFCRDVFNELPLEFAAEVNALMSLKLEGSVG*

>jgi|Tetstr1|442129|TSEL_030283.t1
MAAATASACSSASLRGAPGRPTARFGRSGVLQLPVRRATGRAGRRAAVVRATATPQESAELSEKLAKLENIDSLESEEKREIR
DILKRPYKYGFTSAVESVAIPKGLDEDTVRLISAKKEEPEWLLDFRLRAYRKWLTMKEPEWSDNHYPPINYQDVVYYTEPKQK
KKLNSLDEVDPELLAAFEKLGIPIGEQKRLTNVAVDAVFDSVSIATTFREELAKAGVIFCSISEAVKEYPEIVKKHMGSVVPI
ADNYFAALNSAVFSDGSFCYIPKGVTSPMELSTYFRINSEESGQFERTLIVAEEGSYVSYLEGCTAPAFDTNQLHAAVVELSC
AKDAEIKYSTVQNWYAGDEDGKGGIYNFVTKRGLCAGDNSKISWTQVETGSAITWKYPSVVLAGDNSIGEFYSVALTNNKQQA
DTGTKMIHVGNNTRSRIVSKGISAGESLNCYRGLVQVQPGAKNARNYSQCDSMLIGDKAGANTYPYINVRDPSARVEHEASTS
KIGEDQLFYCQQRGIDMEAAVAMIISGFCQEVFNELPMEFAEEVNQLMSLKLEGSVG*

>jgi|Tetstr1|450808|TSEL_037844.t1
MSDYDIPTLKENIDRDTVDQVLALDVDKYKYGFETIIESDKSPKGLNEDTVRFISAKKNEPEWMLEWRLEAFRRWLTLEEPTW
ARVSYPEIDFQDQYYYSAPKSTTGPKSLADVDPELLRTYEKLGIPLSEQKILAGVQGEDATQGTPFGSNVAVDAVFDSVSVVT
TFREELAKHGIIFCSISEAVREHPELVKKYLGSVVPVTDNFYATLNSAVFTDGSFVYIPKGVRCPMELSTYFRINERNTGQFE
RTLIIAEEGSYVSYLEGCTAPMRDENQLHAAVVELVALDEAEIKYSTVQNWYPGDKDGKGGIYNFVTKRGDCRGKNSKISWTQ
VETGSAITWKYPSCILRGDGSRGEFYSIAISNGKQQVDSGTKMIHLGKNTSSRIISKGIAAGRSDNTYRGQVSSHRRATGARN
FTQCDSLLIGDRCGAHTVPYIESRNSSTVFEHEATTSKISDDQMFYCLQRGLSEEEAVALIVNGFVRDVLQHLPMEFMVETQK
LIGISLEGSVG*

>jgi|Tetstr1|447492|TSEL_034873.t1 (NIFS fusion in Nter)
MAQGRAIYLDHNAGAKLRPAVADAMRELIGVVGNASSVHAPGRAARAQIEAARSAVAALAGVEAANVVFTSGATEANVTALSP
VWVDGRGSRSFDRLLVGACEHPSRLGELTADGRSVLVSVMAANNETGVIQPLEEIGQAVHEVGATLHVDAVQAAGRLPLDLER
WRADSVSLSAHKIGGPQGAGALVLRNAGLHPVSLLTGGGQERRKRAGTENVLAIAGFGVAARIVLEEIADWDRLRVLRDGLER
DLLHIKRETRVFGQNAQRLPNTSCFAVPGMPAETVLIALDLEGIAVSSGSACSSGKVSSSHVLAAMGVDRDLARCALRVSLVQ
ETIDRVRSIDVDQYKYGFSTDIESDKAPKGLNEDIIRLDLGEKRANARVDAGLASTGRSNAGRQMDEPEWAMPVSKGAEGSSP
RKARKVAVEAVFDSVSVGTTFQKELKEAGVIFCSISEAIQEHPELVKKYLGSVVPITDNFFATLNSAVFSDGSFVYVPPGVRC
PMELSTYFRINAENTGQFERTLIIADKGSYVSYLEGCTAPQRDTHQLHAAVVELVALDEAEIKYSTVQNWYPGDKDGKGGIYN
FVTKRGDCRGDRSKVMWTQVETGSAITWKYPSCILRGDDSQGEFYSIAIANNYQQADTGTKMVHLGKNTKSRIVSKGISAGKS
QNTYRGLVSAHRKASNARNFTQCDSLLIGQDCGAHTVPYIDSKNATAVFEHEATTSKISDDQMFYCQQRGLSDEEAVALIVNG
FVKDVIQQLPMEFAVEAQKLISISLEGSVG*
```

>jgi|Chlpri1|6983|rna-gnl|IITBIO|A3770_13p69830_mRNA_A3770_13p69830
MKAKVVTRGGLGLGKGRKPAACAAKASCSYEEAASSLRRCIVARSASTLAKGEDGWVLGGGGRRERGALTRVGCSVEGVEEVD
AVELEKKEIKDILAKPYKFGFVSDIESEAIPKGLSEETVRMISKKKNEPEWMLEFRLKAYRKWLTMEEPDWSDNRYPKIDFQD
IVYYSEPKQKEKKQSLDEVDPELLRTFDKLGIPLGEQKRLANVAVDAVFDSVSIATTFREDLLKAGVIFCSISEAVHEYPDLV
KKYMGSVVSEGDNYYTALNSAVFSDGSFCYIPKGVKSPMELSTYFRINASDTGQFERTLIVAEEGSYVSYLEGCTAPSYDTNQ
LHAAVVELYCAKDAEIKYSTVQNWYAGDETGKGGIYNFVTKRGLCAGENAKISWTQVETGSAITWKYPSVVLKGDNSVGEFYS
VALTNNYQQADTGTKMIHLGKNTRSRIVSKGISAGKSQNCYRGLVQMGGNAENARNYSQCDSMLIGDTAHANTYPYIEVKEPS
SRVEHEASTSKISEDQLFYFQQRGIDAEEAVGMIISGFCREVFQELPMEFAAEVNQLMSLKLEGSVG*

>jgi|Pico_ML_1|52773|g3433.t1
MATLRRPCAAFPTRSRTSRAPLCHSKVHRRVVEARAATDAPAPATETEETTEIRRLLSQPYKWGFKTDIDAVTIPRGLSEDTV
RLISAKKNEPEWMLDFRLKAFRKWLTMKEPDWSDNHYPPIDYQNIIYYSEPKMKEQKQSLDEVDPDLLATFDKLGIPLGEQKR
LANVAVDAVFDSVSIATTFREELAKAGVIFCSISEAIKEYPDLVKNYLGSVVPVGDNYFTALNSAVFSDGSFCYIPKGVKSPM
ELSTYFRINAQETGQFERTLIVAEEGSYVSYLEGCTAPAYDTNQLHAAVVELYAAKDAEIKYSTVQNWYAGDEQGRGGIYNFV
TKRGLCDGDHSKISWTQVETGSAITWKYPSVVLKGDNSIGANTYPYIQVKDPSAKVEHEASTSKIGEDQLFYFQQRGIDQEQA
VGMIISGFCREVFNELPMEFSAE*

>jgi|MicpuC3v2|6075|wlab.223738.1
MTSTSLCAAANAARVGAKSRRAGRAATLSSRGITSPGDVAASVSGRARGGKRVGWRRADATPRAEAISDPAGVVEEEKDAKQE
EVDKIQQMLSKPYKPGFKTDIETDTIPKGLSEDTVRIISAKKNEPEWMLEFRLKAFRKWLTMEEPDWSDNRYPDIDYQAVSYY
SAPKVMEKKKSLDEVDPELLKTFDKLGIPLSEQKRMTNVAVDAVFDSVSIATTFREDLAKVGVIFCSISEAIREYPELIKQHL
GSVVPVGDNYFSALNAAVFSDGSFCYIPKGVKSPMELSTYFRINAESSGQFERTLIVAEEGSYVSYLEGCTAPAYDENQLHAA
VVEISAAKDAEVKYSTVQNWYAGNEDGVGGIYNFVTKRGLCKGDNSKISWTQVETGSSITWKYPSVVLAGDNSVGEFFSVALT
NNKQQADTGTKMIHVGKNTRSRIVSKGISAGKSRNCYRGLVQVSPSAVGARNYSQCDSMLIGDKAGANTYPYISVREPSARVE
HEASTSKIGEDQLFYFQQRGIDPETAVGIIISGFCNEVFNELPLEFAAEVNALMSLKLEGSVG*

>jgi|Praco1|14672|PRCOL_00000395-RA
QFSSPARVRGERRRRATARHGARRVGGGGEVARSAGGRRRWGGGGEARRARGGDGGGVGGGGAPLRGRAAAGARARGRCRRAP
PRAAVAPDSGAAAAPAAEEEELTETERLNKLLAKPYKHGWKTEIESETIDKGLTEDTVRLISAKKGEPEWLLESRLKAFKVWQ
SMEEPIWSDNHYDKIDYQDISYYSAPKTKEKKASMDEVDPELVRTFDKLGIPLNEQKRLANVAVDVVFDSVSIATTFKEDLAK
AGVIFCSISEAVKDYPELVKKYLGTVVPPRDNYFAALNSAVFTDGSFVFIPKGVRCPMELSTYFRINEMETGQFERTLIVAEE
GSHVSYLEGCTAPAYDENQLHAAIVELVCEKDAEIKYSTVQNWYAGDAEGRGGIYNFVTKRGLCAGENSKISWTQVETGSAVT
WKYPSCVLKGDNSVGEFYSVALTNNMQQADTGTKMVHIGKNTRSRIVSKGISAGKSQNAYRGLVQVQPTAVGARNYSQCDSML
IGDNAAANTYPYISVRDPASRVEHEASTSKIGEEQLFYFQQRGIDLEEAVGVIISGFCQDVFRELPLEFAAEVSQLMSLKLEN
AVG*

>jgi|Chlat1|2051|Chrsp17S02525
MAAAMTAAAAAVPAERLLFRAPATRPESRTKTELSAKRSSAFGSPLSLPQSVSTRQQPVSSTRAEVIQEPAPMPVLPKKSASD
DTISKFLEREYKYGFVGNIESETIPKGLNEETVRMISAKKEEPDWMLEFRLKAFRKWQTMKEPTWSDNKYKPIDFQAISYYSA
PKNKPTLDSLDQVDPEMLKYFEKLEIPLGEQKRLANVRQVAVDAVFDSVSIATTFKKELAEAGVIFCSISEAVKEHPDLIKKY
LGSVVPIGDNYYAALNAAVFSDGSFCYIPKGVTCPMDLSTYFRINASETGQFERTLIVAEEGSYVSYLEGCTAPSYDENQLHA
AVVELYCAANAEIKYSTVQNWYAGDKNGVGGIYNFVTKRGLCAGKKSKISWTQVETGSAITWKYPSVVLAGDNSIGEFYSVAL
TNNHQQADTGSKMVHVGKNTRSRIVSKGISAGQSVNCYRGLVSIGPNAAGARNYSQCDSMLIGDNAGANTYPYIQVREPSAKV
EHEASTSKIGEDQLFYMQQRGIDTEQAVAMIISGFCRDVFNELPLEFAAEVNGLMSLKLEGSVG*

>jgi|Mesovir1|3765|Mesvi232S04464
MAAVHLNATAQLAMAASVCAAPAASQVFRRRGEVLSKGGFRGASLASRPRASGRIVYKTSRKCVATSLPEVVEEESEDMKTIS
KFLDREYKFGFVSDIESVSIGKGLTEDTVRLISAKKNEPEWMLDYRIKAYRKWLTMDEPQWSDNKYPKINFQDIIYYSEPKFK
PSVDSLDQVDPELLRTFDKLGIPLGEQKRLANVKPVAVDAVFDSVSIATTFKKDLAKAGVIFCSISEAVKEHPELIKKYLGSV
VPVADNYYAALNAAVFSDGSFCYIPKDTVCPMELSTYFRINASETGQFERTLIVAEENSHVSYLEGCTAPSYDKNQLHAAVVE
LYAASGAEIKYSTVQNWYAGDKDGLGGIYNFVTKRGLCAGARSKISWTQVETGSAITWKYPSVVLAGDNSVGEFYSVALTNNH
QQADTGTKMVHVGKNTRSRIVSKGISAGNSVNCYRGLVQVNPGATGARNYSQCDSMLIGDKAGANTYPYIQVRDPSAKVEHEA
STSKIGEDQLFYFQQRGIATEQAVGAIISGFCREVFNELPMEFAAEVNQLMSLKLEGSVG*

>jgi|Klenit1|10541|rna-KFL_003990110
MEAVASPALHTQAAAAGFGQRTHGTHVQTQVRTQAALLPRQQRHPKLHSFGSAKSIAGQKVCAESEGFRRDRKGRGHARAAGT
QSPTRERPSVKEAAEKIVGGLGGGLDEVKLDEVAGQVAKETGSEKAQISEFLKREYKAGFVTDIQSERIPRGLSEETVRTISA
KKNEPQWMLDYRLRAYRQWLKMEEPRWSDNKYPRIDYQDIVYYSEPKKKDVKASLDEVDPDLLATFEKLGIPLSEQKRLSNVA
VDAVFDSVSIATTFRKELAQAGVIFCSISEAVREYPDLIKEYLGSVVPPGDNFFAALNAAVFSDGSFCYIPKNVECPMELSTY
FRINDSDTGQFERTLIVAEEGSKVSYLEGCTAPSFDTNQLHAAVVELYAHKNSEIKYSTVQNWYAGDEEGVGGIFNFVTKRGL
CEGKNAKISWTQVETGSAITWKYPSVVLKGDNSIGEFYSVALTNNRQQADTGTKMIHVGKNTRSRIVSKGISAGNSVNCYRGL
VQVAPTAHGARNHSQCDSMLIGDQAGANTYPYIQVKDPTARVEHEASTSKIGEDQLFYFQQRGIEAEKAVGMIISGFCRDVFN
ELPLEFAAEVNQLMSLKLEGSVG*

>jgi|Mesen1|10608|ME000086S10145
MGRKDRLQRGNWRCEAAEGGVLAPERTSGREKTSSDETIDNFLKRDYKYGFVSNIESVAIPKGLSEETVRIISAKKKEPEWML
EFRLNAYRQWLKMTEPKWSDNKYPAIDFQVLVLVLVLVLLCLCLWLAVDQGVYGGGFDEVNAGGKTRVSQCCPSTSYTSDWGW
AGGGGGGGGDSAAGGGIQDVVYYSEPKMKEKKASLDEVDADLLDAFEKLGISLSEQKRLANVAVDAVFDSVSIATTFRKDLAK

AGVIFCSISEAVREYPELIKKYLGKVVPVGDNYYAALNAAVFSDGSFCYIPKDVTCPMELSTYFRINAMETGQFERTLIVAED
NSYVSYLEVKDPSARVEHEASTSKIGEDQLFYFQQRGIDSEKAVGMIISGFCRDVFNELPLEFASEVNQLMSLKLEGSVG*

>jgi|Chabra1|342956|rna-CBR_g37549
MAAMAAMAAFAATAGAPKPTPAPALVAYSARGVGGEGASSVSAPVALIARKPGALSVARYEGSACSLCSSSKSSSFLGGSAVF
LPNKDKSKSKSSSLSFTPPSSSSSSSSLSSSSFSSIPRAAAQPQSLIPAESSTPSSSSVLDKPEAEDDVIQQFLKKDYKWGFV
TDIESDSIPKGLSEETVRVISAKKKEPQWMLEFRLNAYRQWLKMREPTWSDNRYPPIDLQNVCYYSEPRQKEKKASLDEVDPE
LLRTFEKLGIPLGEQKRLANVAVDAVFDSVSIATTFREELAKAGVIFCSISEAVREYPELIKKYLGRVVPVADNYYAALNAAV
FSDGSFCYIPENTISPMELSTYFRINASETGQFERTLIVAEKGSYVSYLEGCTAPSYDKNQLHAAVVELYCAEGAEIKYSTVQ
NWYAGDEEGKGGIYNFVTKRGLCEGKGSKISWTQVETGSAITWKYPSVVLKGDNSVGEFYSVALTNNRQQADTGTKMIHVGKN
TRSRIVSKGISAGKSVNCYRGLVQVGPTAHNARNYSQCDSMLIGDTAGANTYPYIQVKDPTARVEHEASTSKIGEDQLFYFQQ
RGIEMEKAVGMIISGFCRDVFNELPLEFAAEVNQLMSLKLEGSVG*

>NP_050730.1 putative ABC transporter (chloroplast) [Guillardia theta]
MSDDLSKRSLRELVSQPYKYGFHTDIENEEFPKGLDEDIIKEISKLKCEPSYMLDFRLKSYILWKKMSLP
EWACLTYLNINYQDIVYYSAPKNSTKLDSLEDVDKKILETFDKLGIPLNEQKKLANVAVDAIFDSVSVGT
TFKQELSNVGVLFCPLSEATNKYSTLVEKYLGSVVPIGDNYFAALNSAVFSEGSFCYIPPNVKCPLELST
YFRINNENSGQFERTLIIADFNSYVSYLEGCTAPMYDKNQLHAAVVELIALENAEIRYSTVQNWYSGDTN
GKGGIYNFVTKRGLCAGKSSKISWTQVETGSAITWKYPSCILVGEDSVGEFYSVALTNNYQQADTGTKMI
HVGRGSKSRIISKGISAGYSKNTYRGQVKININALGSINNSQCDSMLIGPYSQANTYPYIQVSNAMSRVE
HEASTSKIEEEQLFYFLQRGISVEQAISLLISGFCRDVFVKLPMEFAVEADKLLSVKLEGTVG

>YP_277324.1 putative ABC transporter (chloroplast) [Emiliania huxleyi]
MEKQTNDSEKVLEEIVVTPYKYGFKTEIENENFPKGLNQEIVDQISRKKDEPLFMSQFRAQAFSQWQKMP
SPDWAYLGIPPIDYQNIQYYSVPKTKKKLGSLDEVDPELLKTFDKLGVSLNEQKRLTNVAVDAVFDSVSI
GSTFKVELQRAGVIFCSFAEAVERYPELIKKYLGSVVPIGDNFFAALNSAVFSDGSFCYVPKDVKCPMEL
STYFRINNEEAGQFERTLIVVEDRGEVGYLEGCTAPQFSTNQLHAAVVELIAFEEAVIKYSTVQNWYAGD
ENGIGGVYNFVTKRGLCSGRKARIAWTQVETGSAITWKYPSCILAGDSSIGEFYSVALTTNKQEADTGTK
MIHLGSNSKSRIISKGISTGNSKNSYRGLVKVSPRAINTQNYSQCDSLLIGKDSSANTFPYLEIQNSETK
IEHEASTSKIAEEQLFYLLQRGISMEEAVALMVNGFCKEVFNELPFEFASEADRLLNLKLEGSVG

>XP_001712812.1 SufB involved in iron sulfur protein biogenesis (nucleomorph) [Bigelowiella natans]
MITKTNYKAKLTTYTIKKNCIKKISFRTFQQTESYKYKLKSFSECETIRKSLNSNTLKSISLKNLEPLWI
IKFRLDALKSLYNRDTPKWSEFNSYNFDINNSIYYSALIKKDTALVSSNNETSIISSSSKKKTAIDLVLD
SISIDTSYQKELGKHGVIFTNLSNSLKNYSDLISNFMGSVVSLRDNYYTSLNSAVFSEGSFIYIPANLVC
PLDLSTYFRINGNVIGQFERTLIVTEKNASVTYLEGCTAPYFTENQLHAAVVELCAEEGSEIKYSTVQNW
YPGDSMGKGGIFNFVTKRGLCNGSRSKISWTQVETGSSLTWKYPSVILKGDNSVGEFYSIALTKLRQQAD
TGSKMIHIGRKTFSKIISKGISLGNSKNCYRGLVLIHNKSLKSKNTTQCDSIIFGDSSSANTYPYVTVRD
SSAWLEHEASIARIQDDQVFYLQQRGLGFEEAINLIVGGFCKEVIEELPFEFLTEVNELLNSSMIKKIG

>YP_874419.1 iron-sulfur cluster formation ABC transporter (chloroplast) [Phaeodactylum tricornutum]
MVNKSNKVLNKNITNLVNQTYQYGFSTTIEKDIIEKGLNEKTIHLISQKKKETKFLLNFRLKAYKKWKQM
PEPEWAYIKFPQIDYQDVIYYSAPKSQKKLKDLSEVDPELLKTFEKLGISLTEQKRLANVAVDVVFDSVS
IGTTFKEELNKSGVIFSSISEAVTEYPELIEKYLGSVVPIGDNYFAALNSAVFTDGSFCYIPEDTICPLD
LSTYFRINDEKSGQFERTLIISEKNSQVNYLEGCTAPQYDSNQLHAAVVELIALENANIKYSTVQNWYAG
NEFGKGGVYNFVTKRGLCAGSSSKISWTQVETGSSITWKYPSCVLIGDNSQGEFYSVALTNNYQQADTGT
KMMHIGRNTRSRIISKGISAGKSKNSYRGLVNVTSKALGARNYSQCDSLLIGDLSNANTFPFISVQNSTT
KIEHEASTSKIGEEQIFYFLQRGICLEKAVELMISGFCREIFTELPLEFAAEADKLLTLKLEGSVG

>YP_003289159.1 Chloroplast conserved hypothetical protein (chloroplast) [Ectocarpus siliculosus]
MMTETNNATLQQLVNQPYKYGFSTDIEIDTLPPGLNEKIVRNIIKKNNEPDYMFHFRIGALKKWRQMKSP
TWAKLDFLEMDYQKIVYYSAPKTKKTLANLDEVDPEIKDTFDKLGISLNEQKRLANVAVDAVFDSVSIAT
TFKEELEKYGVIFCPISEAIKNYPHLVKQFLGSVVPFGDNFFAALNSAVFTDGSFCYVPKNLRCPLELST
YFRINNKESGQFERTLIIAEEGSYISYLEGCTAPQYDTNQLHAAIVELIALKNAEIKYSTVQNWYAGDKN
GIGGIYNFVTKRGLCIGENSKISWTQVETGSAITWKYPSCVLIGNESQGEFYSVALTTNMQQADTGTKMI
HVGSNTKSQIISKGISSGYSKNSYRGLVKIGTKALDSRNFSQCDSLLFGADAQANTFPYIQVQNSTSKIE
HEASTSKVGEEQLFYLLQRGINAEDAVTLILTGFCKIVFDELPIEFASEVEHLLRIKLEGSVG

>YP_008520066.1 cysteine desulfurase activator subunit sufB (chloroplast) [Nannochloropsis oceanica]
MTTFLDHKQIDLVRQGCRFTERCVKPLRDNTYKYGFATVIESDEIKSGLDLNTIKLISAKKDESGFIIDF
RSRAFKKWQSIKDPAWAELNYPPIDYQAIKYYSSPKVQEKLKSLDEVDPELRDTFERLGIPLNEQKRLAN
VAVDAVFDSVSIFTTFKRELLKLGIIFCSISEAINTYPELVKKYLGSVVPITDNYFVALNSAVFTDGSFA
YIPKNLQSPIELSTYFRINNEQSGQFERTLIIAESGSMISYLEGCTAPKYDSNQLHAAVVELVSHEGAHI
RYSTVQNWYAGDKKGKGGVYNFVTKRGLCLGNESKISWTQVETGSAVTWKYPSCLLMGNASQGEFYSVAL

TTNYQQADTGTKMIHIGKNTRSRILSKGISGGHSKNTYRGLIQFGRKALQAVSYSQCDSLLLAHQSETNT
FPYINVRNSSARVEHEASISRIGEEQIFYFHQRGIPEEDAIKLIVNGFCQEVFRKLPMEFALEAEKLLDL
KVEKSLT

>AIM52780.1 iron-sulfur cluster formation ABC transporter (chloroplast) [Ochromonas sp. CCMP1393]
MKFLFFNNKKFMENSKNQTNFEINQVINQPYKYGFTTEVETEKFSPGLNTEVIKLISKKKEEPFFLTEFR
LKAYKKWLKMKFPQWANLTINPIDFNAITYYSIPKKKKQLNSLDEVDPEILRTFEKLGISLEEQKTLSNV
AVDAVFDSVSIGTTYKEKLAKEGVIFCSISEAIQYYPKLVQKFLGTVVSPGDNFFASLNSAVFTDGSFCY
IPQNVTCPLELSTYFRINDKDSGQFERTLIIAEKNSSVNYLEGCTAPQYSENQLHAAIVELIAFENANIK
YSTVQNWYSGDEKGIGGIYNFVTKRGLCIGKNSNISWTQVETGSSITWKYPSCTLIGKGSSGEFYSIALT
NNYQQADTGTKMIHLGEETTSKIISKGISCGSSKNSYRGLVKIASNANCSRNFSQCDSFLLGSSSLACTY
PYLDIWNSTSIVEHEATISKVSEEQLFYLTQRGISVEQAVGLLINGFCKEVVTMLPMEFATEADKLLSLK
LEGTVG

>ACS36807.1 ABC transporter (chloroplast) [Aureococcus anophagefferens]
MSDVKNKGVSTIDQIVSQPYKYGFETEIEKERIPVGLDENTVRLLSSKKGEPEFMCQSRLQALKIWKNLD
EPNWAELHHPEINYQDITYYSAPKKKKKLDNLSEVDPELLKTFDKLGISLNEQKRLSNVAVDAVFDSVSI
ATTFKKELAKAGVIFCPLSEAVQQYPELIKAYLGKVVPAGDNYFAALNCAVFSDGSFCYVPKNVKCPMEL
STYFRINDEESGQFERTLIIAEQNSYVSYLEGCTAPQRDKNQLHAAIVELVALENAEIKYSTVQNWYAGN
EKGQGGIYNFVTKRGLCSGKNSKISWTQIETGSAITWKYPSCILVGDSSVGEFYSVALTNNYQQADTGTK
MVHIGNNTKSTIISKGISAGNSKNSYRGLVKISPKAKGARNFSQCDSLLIGDKSNANTFPYIEVKNSTTK
VEHEASVSKIGEEQLFYFSQRGIEVEQAISLMISGFCHDVFLKLPMEFSVEADKLLSLKLEGSIG

>jgi|Symmic1|40445|rna28602
MAGPGIFSFLDLPPPEEEPAPQNESRDSSDDSPAIRDAPTEKAANSFFPTISTAGLLHDSDSDGDGLYLQHPGQVPQDVLAVA
CEPPPESFPGLPTHVVLKVLSIEGLPLRKEERNVPGSKGLGYFLEGKDTIDRSGASAAILPEPQSRPPGRHRCAEPRIFAELS
DDHWQAVWDRPGEVRVKLKDGQDSVVVGIVLGQEVVAVTAELDLTGTMRRFFQPQQLYRPAFSGVALGLIEGARAKLALELYP
PGSQLPGRPVAEPEKPSMVRPPKDVPISDCRFCDGYGRRSCDGCGGHGVLVCSTCDGMPALPCPVCRGRGTLHDNLEGIGGPR
SRRAQDGAVLSTVKGRRCQNCWGAPLTCKECFGAAALRCNVCNGAGWSAGSVELKQKQEEAVALRDGQNAELGEVLDQEYSAG
FYTDIESESLPKGLNEDIVRKIWEIKEEPEWMLEFRLNAFRKWKSMKMPEWAQLQMEEIDFQDIVYYSRPKTKKKKQSLDEVD
PELLDTFEKLGIPLTEQKRLTNVAVDAVFDSESIGTTFQKELEEAGVIFCSINEAMKEHPELVKKYLGSVVPVADNYFAALNS
AVFSDGSFCYIPKGTTCPMEISTYFRINNKESGQFERTLLIADESSYVSYLEGCTAPMFDSKQLHAAVVELHAAESAEIKYST
VQNWYAGDKNGKGGILNLVTKRGMCEGKKSKISWTQVETGSAVTWKYPSCILKGDDSIGEFYSVALTNNYQQAVSSVILTEAR
MVTVLSCLVPGSDSLATDPAMLSFDGLLTQVYEAGAEDEEAEESAEAEEYENRGASRRREVEVASPAQVRRREPEPDYPSPRE
LQLPHRYPVWSAGAASSARLKSSSSAPALPRVRSARQGEESLLSPLIDGPGHPAAQRQAAHWKPVRRKLPQQGHLRPLSHLRR
LDTPEDPKKWMPPKVPGGGAWGGILQSQHQRLAGVLAKQHQEMKKMQQQLEVLQQLNRLGSYVGGDARVDGAVHSYMVEMAPK
VGRKAPSWKPLSPIRRSALPVLPEDEEARASLPGSRGQSSEAFVEEAEEVLADPALEAQQVSEDEQGETDAFEQKLEAEGAPE
TEAEGVPAAEACATGQGSLEGEIEVSVQVTLPSDEDEEASLQGLQPSVEAQLASGDVLVPEEVGSHTDGDAVEQLRKEVWPPA
LMLCIQLA*

SUFC orthologs

>jgi|Galsul1|3981|XM_005704982.1 SufBC fusion
MNQSLDATTLKLEQIINKSYKYGFSTKIQNEDFPKGINEEIVTLISKKKNEPKYMLDFRLKAYKKWKQMYVPKWQYLKFEQIN
YQNILYYSVPKQQKKLQSLNEVDPELLKTFEKLGIPLNEQKRLTNVAVDAVFDSVSIGTTFKQELADQGIIFCSISEAIQKYP
SLIQKYLGSVVPIGDNYFAALNSAVFTDGSFCYIPPDTICPLELSTYFRINNEESGQFERTLIIADRNSYVSYLEGCTAPQYK
KNQLHAAVVEIIALENSEVKYSTVQNWYSGDENGNGGIYNFVTKRGLCAERNSKISWTQVETGSAITWKYPSCILVGNYSKGE
FYSVALTNHYQQADTGSKMIHIGKNSKSKIISKGISTGKSINSYRGKVKISSNASKARNYSQCDSLLIGNSSEANTFPYIEVH
NRSSIIEHEASISKINEEKLFYFMQRGISIEEAISLIVSGFCKEVFTELPLEFALEADKLLGLKLEGTNTEEYILNGINLNVK
QGEIHAIMGPNGSGEIIFQEQNLLDYTIEDRANLGIFLAFQYPLEISGVNNIDFLRLAYNSKLKFNQTSTVDPLKFLEIVYPK
LKLVGLDESFLHRKVNEGFSGGEKKKNEILQMALLDAKLAILDETDSGLDIDALKDISNAIKSILKISQFKQSIIIITHYQRI
LNYIQPDYIHVMYKGKIIKTGDASLASELESKGYEWITSE*

>AIU44589.1 sulfate ABC transporter protein (chloroplast) [Cyanophora paradoxa]
MSTEKTKILEVKNLKAQVDGTEILKGVNLTIYSGEIHAIMGPNGSGKSTFSKILAGHPAYQVTDGEIIFK
NKNLLELEPEERARAGVFLAFQYPIEIAGVSNIDFLRLAYNTRRKEEGLTELDPLTFYSIVKEKLNVVKM
DPHFLNRNVNEGFSGGEKKRNEILQMALLNPSLAILDETDSGLDIDALRIVAEGVNQLSNKDNSIILITH
YQRLLDYIVPDYIHVMQNGRILKTGGAELAKELEIKGYDWLNELEMIKK

>YP_009051123.1 sulfate ABC transporter protein (chloroplast) [Galdieria sulphuraria]
MTHNSLLQIKNLHVKLANTEEYILNGINLNVKQGEIHAIMGPNGSGKSTLSKVIAGHSLYKIVKGEIIFQ
EQNLLDYTIEDRANLGIFLAFQYPLEISGVNNIDFLRLAYNSKLKFNQTSTVDPLKFLEIVYPKLKLVGL
DESFLHRKVNEGFSGGEKKKNEILQMALLDAKLAILDETDSGLDIDALKDISNAIKSILKISQFKQSIII
ITHYQRILNYIQPDYIHVMYKGKIIKTGDASLASELESKGYEWITSE

>jgi|Chlre5_6|16639|Cre07.g339700.t1.2

MAHQILRSSRIASSTRVAQRSSRVHAVSVRCAAAPADVMLEVKDLTASIAGTHTPILKGVNLTIRNGEVHAIMGKNGSGKSTL
SKVLVGHPDYEVTGGTAVFKGKNLFEMEPEERSHAGLFLSFQTPIEVPGVSNVDFLRMACNSRRKALGHPELDPLEFYAYIMP
KLEMLNMDPTFLNRNVNEGFSGGEKKRNEILQLAVLEADMAILDEIDSGLDIDALRDVAKAVNQLRSDDTGVLMVTHYKRLLD
YIKPDFVHIMQAGEIVKTGDMSLVDQLEAGGYATL*

>jgi|Caulen1|97221|g3371.t1
MKSMRLVQGSSLRDSGTPVRNVIPSLKRRIRCRSAASAKESTTELFLKVEGLKAEIAASGESVLNGVNLTVNYGEIHALMGKN
GSGKSTLAKVLVGHPDYNVTEGSAIFKGQNLFELEPEERSHLGLFLSFQSPIAIPGVNNVDFLRLACNARRKAKGETELDPLE
FYAYLTPKLEALKMDPTFLNRNVNEGFSGGERKRNEILQLAVLESDMAILDEIDSGLDIDALRDVANAVNGLRTEKTGILLVT
HYKRLLEYLRPDYVHVMQDGKITKTGDMQLADQLEEKGYAPLKN*

>jgi|ChlNC64A_1|37406|estExt_Genewise1Plus.C_290103
MGKNGSGKSTLSKVLVGHPDYEVTGGSAAYKGQDLFELEPERRSHMGLFLSFQSPVEIPGVSNIDFLRIATNARRSANGEPEL
DPLEFYAHVMPKLESLNMDPAFLNRNVNEGFSGGEKKRNEILQLACLEADMAILDEIDSGLDIDALRDVSKAVNGLKAQRPDM
GILMVTHYKRLLDYIRPDQVHIMQDGRIVTTGDMGLVDKLELEGYSVLRS*

>jgi|Tetstr1|432597|TSEL_021967.t1
MNTCKATRGGQALRPAPSSAGRLQARRLPSLRSVAPRRAVRVLAAAGDVVLEVKDLRANIAGTDQAILKGVNLTVRAGEIHAI
MGKNGSGKSTLSKVLVGHPDYIVTGGSVMYKGEDLFALEPEERSHKGLFLSFQSPIEIPGVSNVDFLRLSCNSRRKAQGKDEF
DPLEFYSYIMPKLEALNMDPTFLNRNVNEGFSGGEKKRNEILQMSVLESEMSILDEIDSGLDVDALRDVSKAVNGLRTEDNAV
LMVTHYKRLLDYVVPSQVHVMRDGEIIKTGGIEIVDTLEAEGYAALSTA*

>jgi|Chlpri1|7803|rna-gnl|IITBIO|A3770_16p78030_mRNA_A3770_16p78030
MASTARGMRPSMARTPRATVGTSTRRGIGIGIPAFGMRKGVTAPMQEAGGGGCGSCCSRCSERRRRRKCRVAATTEEAGGANG
CVLEVSGLEASIDDKSILKGLNLRINRGEVHAIMGQNGCGKSTLSKVLVGHPDYKVDGGSVTYKGGNLLELEPEERSHAGLFL
SFQYPVEVPGVNNTDFLRLACNARRAALGETELDPLEFYGHVTPILQELDIDPKFLSRNVNEGFSGGERKRNEVLQLAVMDSD
LAIMDEVDSGLDIDALKNVADALNKLRRKKKDMSVILITHYQRLLNYIKPDYVHIMKDGRCIKTGGAELALELESEGYASFDG
FDGV*

>jgi|MicpuC3v2|90|wlab.202455.1
MSAATISSRTLTTPASLTRARGASSRAARAASRAVGPVVVRAAKGDVLLEVKDLTAKVAETGETILNGVTLTIKEGETHAIMG
KNGSGKSTFTKVLVGHPSYEVTGGTAVFKGKDLLAMDPEDRAREGLFLSFQSPIEVPGVSNTDFLRMMCNERRKARGEEELDP
LEFYGFLTPKLDQLNMDPSFLARNVNEGFSGGEKKRNEILQLAVMESEMSILDEIDSGLDVDALRDVAAAVNVLKEGDKGLLL
ITHYQRLLDAIHPDFVHVMEKGKITQTGDKSIASKLEEGGFASLA*

>jgi|Praco1|20197|PRCOL_00003471-RA
MACLSARGLCAEVEATGKKILKGVDLTVREGEVHAIMGKNGCGKSTLSKVLVGHPAYKVTAGTATYRGDDLFEMEPEERSHAG
LFLSFQAPIEIPGVSNTDFLRMMYNARRKALGMPELEPLEFYGYLTPKLEQLNMDPSFLSRNVNEGFSGGEKKRNEILQLAVL
ESELSILDEIDSGLDVDALRDVAEAVNGLKSDENAVVMITHYRRLLDYIEPDFVHIMERGKIKQTGGIELAEVLEEKGYAGLS
S*

>jgi|Chlat1|3274|Chrsp22S03442
MSAAAALAGGALSWSAAGRAESSSPSASPSLLRPATGLRASRLRCRAVPSSRSISRPAVSAAAGDVILEVKGLCAKVVDSELD
ILRGVDLTVREGEIHAVMGKNGSGKSTLSKVLVGHPQYEVTGGTAMYKGQDLFELEPEERSHNGLFLSFQSPVEIPGVSNTDF
LRLMANARRKALGKPEMDPFEFYGWLTPKLEQLNMDISYLQRNVNEGFSGGEKKRNEILQLAVLEADLSILDEIDSGLDVDAL
RDVATAVNALRSPDRASILITHYQRLLEYIQPDFVHIMESGRIVRTGDKSLAKQLESGGYAAIK*

>jgi|Klenit1|1526|rna-KFL_000240090
MATSAAAKAASIHNLTCGTSRQSRYETNARCPLLPQSPLHKADVVHSGLKCGASRKAVSRVGKTVVFRQPTCVKAAVSTSEAP
TSTSTEPGKVLLEVEGLEAEVADGGRQILKGLNITIREGEVHAIMGKNGSGKSTLSKVLVGHPDYDVTGGSAKFKGKDLFELE
PEERSHAGLFLSFQSPVEVPGVSNTDFLRMAHNARRQAQGLKELDPLEFYAFLTPKLDQLKMDASFLSRNVNEGFSGGEKKRN
EILQLAVLEADLAILDEIDSGLDIDALRDVAEAVNGLRKSNNAVLLITHYQRLLDYITPDFVHIMQSGRIVKTGGKELAKELE
QGGYGALGPM*

>jgi|Mesen1|8851|ME000053S08269
MALCISNAAAIVPVSSELSHNKSSSRTSQKRQALASCRVLRGANSAHLKLNETSLRSDSLRTARKAPSVGRCRRGTSVTSAAL
AEAEAAFAPVAEGDIILEVKGLCACIAESGKEILKGVDLVIRAGEVHAIMGKNGSGKSTLSKVLVGHPDYDVTGGTAVYKGRD
LFELEPEERSHAGLFLSFQSPVEVPGVSNTDFLQMACNARRAALGLPELDPLEVLEAEMAILDEIDSGLDVDALRDVSAAVNG
LRQPTRAVLMITHYQRLLDYIKPQFVHIMESGKIVKTGDSSLALELEAGGYAAVRG*

>jgi|Chabra1|335080|rna-CBR_g26048
MATALRAAAGAATAAASCCCAPSRLHSEVTSTSASCRSRNGPQSWVGAETACAPRALPLAQQRGVCAPQPQQQLVAPRRGKAQ
CSAPLGGGLLKLDRPHSMAVAAMASAAAAASLADQSTSVAEGGEEGEGRKVLLEVKGLTAAIADGGAEILKGVHAIMGKNGSG
KSTLSKVLVGHPDYEVTGGSAHYKGENLFEMEPEERSHAGLFLSFQSPVEIPGVSNTDFLRMACNARRAARGLPEMDPLEFYA
FLTPKLESLKMDISYLQRNVNEGFSGGEKKRNEILQLAVLEADMAILDEIDSGLDIDALRDVANAVKGLQGPQNAVLMITHYQ
RLLDYIEPDFVHIMEAGKIVRTGDKTLANELEVGGYAALKN*

>SUFC G. theta, reconstituted sequence

MKKKILEVTNLHAAVNEIKIVKGLNLVVNAGEIHAIMGKNGSGKSTFAKIIAGHPDYTITNGDITYQHTSILELTPEDRAKRG
IFLSFQYPIEIPGVTNADFLRLACNARRIYQGLPEMEPLEFFEYINSKLPLVDLKPSFLTRDVNEGFSGGEKKRNEILQMSIL
DTKLAVLDETDSGLDIDALRTVANGIKSLANDGNAIILITHYQRLLDYIKPDFVHVMQEGKIIKTGSASLALDLEKYGYDWLK
NELK-

>jgi|Emihu1|443440|estExtDG_fgenesh_newKGs_kg.C_2400030
MLLVASSSGAAAVALGSRPAPSSAARRGGALQMEAGETVLDVSDLCAAVGETPILKGVNLKVRKGEVHAIMGPNGSGKSTLSK
VIVGHPAYEVTGGSALFEGEDLLELEPEERAQQGVFLAFQYPVEIPGVANNDFLRLAVNKRRQAQGLDEYDPIEFFGVLSEKM
SQVSMSPDFLGRDVNSGFSGGEKKRNEILQLALLEPSLAILDETDSGLDIDALKTVAAGVNAYRGEGNGIVLITHYQRLLDYI
KPDFVHVMSDGEIVRSGGPELALELESDGYAFLDAGARSDA*

>SUFC *Bigelowiella natans*, reconstituted sequence
IYKDCRKLNLTVNPGEIHVLMGRNGSGKSTLSKTIVGHPSYEVIDGSITYKNIDITQIDASLRALNGIFMCFQSPVEIQGVKN
LDFLRKISNSRRKFLSQPIFDPLDFYSFISDKIESVGLDNTFLTRNVNEGFSGGEQKRNEMLQLITVQADFCIFDEIDSGLDI
DSIKNLVDIISTLRNRGVGMIIITHYNKLIDLVRPDYVHILKNGKIIKSGDMELSKIIEREGFDAIN*

>sulfate ABC transporter protein (chloroplast) [Phaeodactylum tricornutum]
MTLNSPLLEIKNLQVSINENQILKNLNLTINKGEIHAIMGPNGSGKSTFSKVLAGHPAYSILGGDILFKGSSILDLEPEERSH
LGIFLAFQYPVEIPGVSNEDFLRLAYNSKQKFYNKVEVDPIEFLTIITQKLQLVNMSARFLGRNVNEGFSGGEKKRNEILQMI
LLDSELSILDETDSGLDIDALKIISNGINNFMGPEKSIILITHYQRLLDYVNPNYVHVMQNGKIIKTGSAELAKELEAKGYEW
LET

>D1J705, ycf16, *Ectocarpus siliculosis* (chloroplast)
MTNKTIPLLEIRNLHANINNNKILRGVNLSIFEGEIHAIMGTNGSGKSTLSKVIAGHPSYDVIEGEILFQGKNICDLDPDLRS
HLGLFLAFQYPVEIAGVDNQEFLEAIYNAKQVSMNLPKANPLFFYELLREKLKMVNLDESFISRNVNEGFSGGEKKRNEILQF
ALLDSILGILDETDSGLDIDALKSISKTINILMENKNKSIILITHYKRLLEYIKPDFIHVMQDGQIVKTGDATLANLLEEKGY
DWLKPISN

>AGI99421, FeS cluster assembly protein (chloroplast) [Nannochloropsis oceanica]
MLEIKNLTAATSIKEGKRNVASILKGVNLNLKAGEIQIVMGSNGSGKSTLAKLLAGHPAYNMLHGSIRFRNEKLIRCLPETRA
RLGLFLGFQYPMEVGGVSNQDFLRLAYHSRYKKKDDILNTSLRFANRILNYVKILDMDPSFLPRPLNQGFSGGEKKKNEILQM
AITGCELAVLDEIDSGLDVDALKTLSKTILIYQLDSLFNSRFLQTTKSLILITHYQRLLEYIRPDKIQVMKEGKIVCSGKSNL
GNIVDRKGYDWLSGTGLGSCLIENINKKKLQTLKNSVF

>AIM52779.1 iron-sulfur cluster formation ABC transporter ATP-binding subunit (chloroplast) [Ochromonas sp. CCMP1393]
MNKTLLKLENLEASIQNTQIIKDLNLEVKENEIHVIMGPNGSGKSTLSKILAGHPSYEIKKGSVNFCEKN
LLDMAPEERSHEGLFLAFQYPIEISGVTNYDFLRIAYNEKRKYLQEEELDPIEFMQLTQRLLNKLQMKNE
FLNRNLNEGFSGGEKKRNEILQMLLLDPKLVILDEIDSGLDVDAIKIICEGINNTLSQNSSLIVITHYPR
ILNYLKPTYVHIMIDGKIIKTGNLELVESLEKDGYQAVI

>jgi|Vitbras1|19345|Vbra_4547.t1
MTSLVACAFLSLIISLVALPRPCTAFRMPSPVHRTPRTRVRSRQPSLLRMSASEDASDTIMRIDNVHAEVGEEEEKVVKILDG
FSLEVKKGEVHAIMGPNGSGKSTLSKVITGHPDYRVIEGSITFKGEDVLDMDVDERALAGIFLAFQYPVEIAGVSNSDFLRAA
VNIRRKREGLDDLDAIEFSMKMFEELDKLGLDPSFLDRGINAGFSGGEKKRNEVLQMVMLNPDLVILDETDSGLDVDSLKTTA
EAVNNFKASHPDKTFLIITHYQRLLDLIKADRVSVMKKGRVVLTGGPDLATTIEQWGYGDWLEKEAAGGREAVLA

>jgi|Symmic1|43348|rna30659
MASMSTSVARQLQSMHNFYGPAKLSWGLANLRVRRQPLLYAVATAVQQRLSESQQSSTKIAWCSVVRAIPPVELWVPERQGSF
DACIDEEMLAHFGKNLIPRLMLCYGACGPRPSLNTQADSSRWLQRGIDLGDMSTAVEDLTWICLLQHTSRPGPCQEVIRKYSC
GRELPAWFRVQDGPWSKSKAKYMQRFSNLEKFAQPASGNHWEKLGAVIDFVEHRLSAAAPQVPGTVNEMLRLLDTFAQRREHW
LKVAGGAKAEVALECGTFIGSRTYAKGVDPVQACIARHLVDLAGASFVLSGGCHWTGEVRDIVPKIVELFGATSVGMVFMDYK
EDTGAIGINSRLLADNVALPGAPLLLWNLAFSPSWELTAFAMTEFHEPNTEDWMALGRITCAAEPAVSDLQKETCLERTRTVD
RAARSKKWCLTQDVLPQTTMKPLVAEIGKVARAMRQLAGRLALAAVALASEDYMARVDPSLHTWTRTVRVAEDAFLLLEMHQG
NLKALLNGEAEVDLPQEQEEQLLSAFPAEALTVEARVPQIQSQRWAAKILRHAVGARVDPDCAVNTENHLEKEILAVFRRLRN
PPIGSVLTFGGLYGGVHGEGVDVPDDPAVELMLELQQRGAGKITDSRAGVAALRTLNIPDPLRWADLQLPAEFAGELDLLRLD
GLRHGVSCEVLRQVRPRLVALLVLSQVPPPFQYFPLGVPGYNSPPALMSCSLSGAIEVLAKHDLFLIRLTGPYALFVRQSEWP
EALPINEFDCYRRASVWGLKDIPLSFVREWLREEVDQVLPRLWRNLTHLHSDGGPFTLVLFCKSCRVEWIRKQGGAMDAMEVA
TMAAPLVSSTSSVHPGRWRRGTTLSRSSPATSFGQRSADSASSTWHTGWLASTAIVGLAASRPTRRRLQSRQLRQTRRAGDSP
VVLEVKEVEAKSTDEDEKQILKGLNLTIRAGEVHAVMGPNGSGKSTLAKVLIGDSAYEVTNGSAKLGETDLLEMEPHERALEG
LFLAFQSPPAVGGVSNLDFLRAAYNAQSIFAAVRMSSGIRRRKVPGSEADEGLSNSGALETLRKFDVYNKVHEDYMQKRQLGG
AVTLVTCAILAVLVYCEVCEFFSVEVLHSITVDTNIDRKLPISLDITFPHLRCSEVSVDTVDSAGDTQVDAHGGLDMHNLDAA
GKISTGDPVASKGDCWPCLEAEDAKHKCCNSCQELKDAYNDKELPYFHVLDTAMQCKSSIGCRIKGKVVVNKVSGNIHVALGK
SVRRDGKLVHEFNIEDIGDGFNTSHHIHSITFGEYVYGLQSPLEGIRKIAGAGSWMYHYYVKLVPTVYISRWGTTTYTNQYSV
TDSARNVQVREGELSGLPGVFLVYDFSPFLMQQTEQVKPWSYVFTSMCAIVGGAFSVATLVEMALSSAREEPELDVIEFYGLV
TQKLQDLKINPDFLNRNVNEGFSGGERKRNEMLQMAVLQPKLAILDEIDSGLDIDALKDVAEAIRSVREQDPDRAMLVVTHFE
RFLRYVEADHVHVMYQGRILKSGGKELADKLDEEGYDWVLKEAK*

SUFD orthologs

>SUFD galderia sulphuraria 074W
MKSEEMCWREICFVPCLSQGNEKLLVQRETRFLSSFPYGYSLHPRKCTEFNKRKSPLVTTRKRPTTLTTF
MSSSTIQESAKQTEDKTWLQSILDCRESTANHEDTAVLNSLHREAEEELKTRLRIPGRKDELWRFTNLRN
LFLTRFQTSSHRVDNQLLESLFEKDVQSQRVVLVDGVFDPFLSNISNIPDRIFIGSVTELDVERQRWIYE
LTKKGETGIQETNIFGAINLASFRDITIVWIPQDVSLQDYIHLCCYSSNCLNSSFYGISHPRIVVVVEGG
SHVKILQHHFGASGGYLDNFATSISIGDSATLEYYIVNECPKDALWLGSLNAQLEQYCTFQFRFLSSGSK
VGRLTMTTDLVGRESNTSLHGLAIANEERTLDFHSFVDHRVPSCHSEQLQRNLVADRSQCVFRGLVKIRR
EAPHTCAHQLCRSMLLSNRAQTKTMPMLEIANDEVECSHGATVSEIEEDELFYLLSRGIPEWDARVLLVR
SFSLELLKEFPFPSIVARMESLIAQVVNNSKEEKVIEADTVEEGQWDWTT

>AIU44588.1 putative ABC transporter (chloroplast) [Cyanophora paradoxa]
MVNTQSPKNSGLENLVNQPYKYGFTTDIEIETISKGLTEDTVRLISAKKNEPEFMLEFRLQAFRKWLEMK
NEPEWAHLNYPKINYQDMVYYSAPKQKKKLQSLDEVDPTLLETFEKLGIPLTEQKRLANVAVDAIFDSVS
VATTFKEELAKEGVIFCPISEAIQKYPDLIKKYLGSVVSTSDNYFSCLNAAVFSDGSFCYIPKNVRCPLE
LSTYFRINNGESGQFERTLIVADEGSYVSYLEGCTAPQFDTNQLHAAVVELVALKDAEIKYSTVQNWYAG
DDNGKGGIYNFVTKRGLCAGENSKISWTQVETGSAITWKYPSCVLLGDNSIGEFYSVALTNRYQQADTGT
KMIHIGKNTRSRIISKGISAGHSQNSYRGLVKISPKALGARNYSQCDSLLIGDNSQANTFPHLQIKNPTA
KVEHEASTSKIGEEQIFYFLQRGINAEEAISLIISGFCREVFNNLPMEFALEADKLLGLKLEGSVG

>Cre12.g513950 SUFD/NAP6
MQASTSLGSACRTQLAGKRVVSGHRYPNARIALKPAKSGAVASAVVAEDKWLARTVKEVATPSSLAALREQSKSALATLRMPT
TRNEDYRFTDIAPLLRTNVQPAAAGAAVPSELLAAHELPGAAARLVVVDGVLRPELSSGLSGLPAGTYVGPLAGAPEAVKQKL
GSLSNSRGGPFAVLNGSLVPEVLVVAVPAERQLEGPLFVLHLASGATGADEVAANAPRLLLHAGTGSSAELVEEYVALPGGRR
YLTVAVGELFLEAAASVRHSYVQREGEGSFHFKSTLVHQAERSQYTLAEASVGGAIARHDLVIQQGGPETHTQMSHFLLCGPS
QTHDLHSRLTLDHPHGTANQLHKCIVSHASGRGVFDGNVKVNRLAQKTDAGQLSRNLLLVPLATVNVKPNLQIIADDVKCTHG
CAVSDLRDDELFYFRARGISAESARQALVFSFGAEVIQRMGHPVLQKRVQDDVSRTLLSAEPFAASA*

>jgi|MicpuC3v2|6075|wlab.223738.1
MTSTSLCAAANAARVGAKSRRAGRAATLSSRGITSPGDVAASVSGRARGGKRVGWRRADATPRAEAISDPAGVVEEEKDAKQE
EVDKIQQMLSKPYKPGFKTDIETDTIPKGLSEDTVRIISAKKNEPEWMLEFRLKAFRKWLTMEEPDWSDNRYPDIDYQAVSYY
SAPKVMEKKKSLDEVDPELLKTFDKLGIPLSEQKRMTNVAVDAVFDSVSIATTFREDLAKVGVIFCSISEAIREYPELIKQHL
GSVVPVGDNYFSALNAAVFSDGSFCYIPKGVKSPMELSTYFRINAESSGQFERTLIVAEEGSYVSYLEGCTAPAYDENQLHAA
VVEISAAKDAEVKYSTVQNWYAGNEDGVGGIYNFVTKRGLCKGDNSKISWTQVETGSSITWKYPSVVLAGDNSVGEFFSVALT
NNKQQADTGTKMIHVGKNTRSRIVSKGISAGKSRNCYRGLVQVSPSAVGARNYSQCDSMLIGDKAGANTYPYISVREPSARVE
HEASTSKIGEDQLFYFQQRGIDPETAVGIIISGFCNEVFNELPLEFAAEVNALMSLKLEGSVG*

>jgi|Pico_ML_1|52773|g3433.t1
MATLRRPCAAFPTRSRTSRAPLCHSKVHRRVVEARAATDAPAPATETEETTEIRRLLSQPYKWGFKTDIDAVTIPRGLSE
DTVRLISAKKNEPEWMLDFRLKAFRKWLTMKEPDWSDNHYPPIDYQNIIYYSEPKMKEQKQSLDEVDPDLLATFDKLGIP
LGEQKRLANVAVDAVFDSVSIATTFREELAKAGVIFCSISEAIKEYPDLVKNYLGSVVPVGDNYFTALNSAVFSDGSFCY
IPKGVKSPMELSTYFRINAQETGQFERTLIVAEEGSYVSYLEGCTAPAYDTNQLHAAVVELYAAKDAEIKYSTVQNWYAG
DEQGRGGIYNFVTKRGLCDGDHSKISWTQVETGSAITWKYPSVVLKGDNSIGANTYPYIQVKDPSAKVEHEASTSKIGED
QLFYFQQRGIDQEQAVGMIISGFCREVFNELPMEFSAE*

>jgi|Praco1|14672|PRCOL_00000395-RA
QFSSPARVRGERRRRATARHGARRVGGGGEVARSAGGRRRWGGGGEARRARGGDGGGVGGGGAPLRGRAAAGARARGRCR
RAPPRAAVAPDSGAAAAPAAEEEELTETERLNKLLAKPYKHGWKTEIESETIDKGLTEDTVRLISAKKGEPEWLLESRLK
AFKVWQSMEEPIWSDNHYDKIDYQDISYYSAPKTKEKKASMDEVDPELVRTFDKLGIPLNEQKRLANVAVDVVFDSVSIA
TTFKEDLAKAGVIFCSISEAVKDYPELVKKYLGTVVPPRDNYFAALNSAVFTDGSFVFIPKGVRCPMELSTYFRINEMET
GQFERTLIVAEEGSHVSYLEGCTAPAYDENQLHAAIVELVCEKDAEIKYSTVQNWYAGDAEGRGGIYNFVTKRGLCAGEN
SKISWTQVETGSAVTWKYPSCVLKGDNSVGEFYSVALTNNMQQADTGTKMVHIGKNTRSRIVSKGISAGKSQNAYRGLVQ
VQPTAVGARNYSQCDSMLIGDNAAANTYPYISVRDPASRVEHEASTSKIGEEQLFYFQQRGIDLEEAVGVIISGFCQDVF
RELPLEFAAEVSQLMSLKLENAVG*

>jgi|Chlpri1|6983|rna-gnl|IITBIO|A3770_13p69830_mRNA_A3770_13p69830
MKAKVVTRGGLGLGKGRKPAACAAKASCSYEEAASSLRRCIVARSASTLAKGEDGWVLGGGGRRERGALTRVGCSVEGVEEVD
AVELEKKEIKDILAKPYKFGFVSDIESEAIPKGLSEETVRMISKKKNEPEWMLEFRLKAYRKWLTMEEPDWSDNRYPKIDFQD
IVYYSEPKQKEKKQSLDEVDPELLRTFDKLGIPLGEQKRLANVAVDAVFDSVSIATTFREDLLKAGVIFCSISEAVHEYPDLV
KKYMGSVVSEGDNYYTALNSAVFSDGSFCYIPKGVKSPMELSTYFRINASDTGQFERTLIVAEEGSYVSYLEGCTAPSYDTNQ
LHAAVVELYCAKDAEIKYSTVQNWYAGDETGKGGIYNFVTKRGLCAGENAKISWTQVETGSAITWKYPSVVLKGDNSVGEFYS
VALTNNYQQADTGTKMIHLGKNTRSRIVSKGISAGKSQNCYRGLVQMGGNAENARNYSQCDSMLIGDTAHANTYPYIEVKEPS
SRVEHEASTSKISEDQLFYFQQRGIDAEEAVGMIISGFCREVFQELPMEFAAEVNQLMSLKLEGSVG*

>jgi|ChlNC64A_1|29265|estExt_Genewise1.C_10416
MLAKPYKYGFKTIIETEQFPKGLSEDVVRAISAKKEEPEWMLDFRLKAYRKWLTMEEPNWSDNRYPRIDYQDLSYYSAPKVVD
KKTSLDEVDPELLATFDKLGIPLNEQKRLANVAVDAVFDSVSIATTFKEELGKAGVIFCSISEAVKEYPDLVRKYLGSVVPVA

DNYFAALNAAVFSDGSFVYIPKGVRSPMELSTYFRINASETGQFERTLIVAEEGAYVSYLEGCTAPAYDENQLHAAVVELSAA
KDAEIKYSTVQNWWGYAGDAEGRGGIYNFVTKRGICLGERSKISWTQVETGSAITWKYPSVVLKGDHSVGEFYSVALTNNRQQ
ADTGTKMIHVGKGTRSRIVSKGISAGHSVNAYRGLVQVQPTAAGARNYSQCDSMLIGDQAGANTYPYIQAGGGAAAGSGQGAR
VEHEASTSKIGEDQLFYFLQRGIDAEDAVGMIISGFCRDVFNELPLEFAAEVNALMSLKLEGSVG*

>jgi|Tetstr1|438113|TSEL_026736.t1
MRVSISPIGARSSKPGLATARRATPAARVGRARRVPAVSAAAVQSAVATKDEWLSSTLQALEQSADGASAPLAEMKGAANDKL
LRSSVPTTRDEEYRFTDLAKLLSSKMCAPAAGGVDTSKLDAMAFSEASGSRLAIIDGKLDLSASNLSSIPSNVYVGNLADAPD
WAVSKLNKQASSSRGNVFSTFNSATATDVAVVAVPAGVKMECPLHIMTLASSAPNEGELSASAQRLLVCLGEDAAVEVVEEFQ
TTQGSGGAFTTSVAEIYLDEGAALKHGVLQLSDSGSGVHMKTTLVQQATNSCYKVVESCLGGNITRHDLNIVQLGPDTNTTMR
HFLLAGKDQLHDLHSKLILDHPRGVADQLHKCIAVSPTSRGVFDGNVKVNRAAQQTDAGQLSRNLLLAPRATVNVKPNLQIVA
DDVKCTHGCTVSDLSEEELFYFASRGIDKETARSCLVYSFGAEVLREHGFEGLSSRLQAEMTSMLAAV*

>jgi|Tetstr1|447494|TSEL_034875.t1
MNMEPRTLETKAEAALAEAYETMRDALPGGAGVARLRTEAFDAFRATGLPHRRVEEWKYSDLRARLKDVPAFAARRSEADAKA
VLASAGEAYASVDRFRLVIVDGHFYPELSDVAGLAGEGVTVRLFAEALAADGEALLDLPVVALADAAVGLNAIFCADGLDLAI
ADNTKLTKPVEIVHVSGAEAASTAERVLIRAGAKSEAVILQTFVGGVEGTFSNTLTDIRAGKGAGLTVARLQAEAAGTTHIAT
ATLTLAEEAQAKLICAGIGSGFARHQSFVAFDGENARADLLGITMVRDRQHVDQTLVVDHAVPHCESREIFKTVVDDGAKGVF
QGKIIVRPHAQKTDGQMMTQSLLLSENADMSLKPELEIFADDVQCAHGATSGQIDEDLLFYLRARGIPEKEARTLLVLAFLSE
AVEEINDEIKPQAVIDAVTRAYSHEYANVHRGLHYLSNTATENFEAARETVRRFLNAPSVDEVIFTRSTTEAINLVAESYGAQ
HIGEGDEIVLTIMEHHSNIVPWHFLRERKGAVLKWVYVREDGSFDLDAFEQAIGPRTKMVAITHMSNVLGTVVPIKQVCEIAH
AHGVPVMVDGSQSAVHMPVDVQDLGCDFYCFTGHKVYGPSGIGVLWGKMELLEKMAPFNGGGEMILDVTEDGVTYNAPPHRFE
AGTPPIVQAIGLGAALDYMDAIGRDRIAAHEARLRDYAQEQLKKLNSIRIFGEAPGKGAIISFEMKGVHAHDVSMVIDREGVA
VRAGTHCAQPLLARYGVTSTCRASFGLYNTVEEVDSLVAALQKAQDFFA*

>jgi|Tetstr1|450807|TSEL_037843.t1
MSTPLLEIKNLHVRVEDNEILKGVNLTLNRGEVHAIMGRNGSGKSTLSYVLAGKEDYEVTEGEILLDGENLLEMEADERAAAG
VFLAFQYPIEIPGVATMTFLKAAMNAQRKARGEGELSTPDFMRAVKDAAGLLEIKQDMLKRPLNVGFSGGEKKRAEILQMALL
APKLCILDETDSGLDIDALKVVSEGVNKLRAPERSMLVITHYQRLLNYIVPDVTPVRLSGAEESLVAQLESAGATDAAERLRV
IGLPTRRVESYHYTDLRTLLGQIPPRAKAASDSSAPAVDIPGAYRILIANGVVQSTSTAPAGVIVGKAAGSVLTTRDDVLVRL
NAALVSESLNLELAGSVDPVIHIDHRMEGPAGHVQSGAKIAFGPGAKATVIETVSGSDAAHMGNVGSYVSVGDGAEVTHILVD
LSARQTTHFATVEYRIGAESSFKSVVINAGAHLARTNVFADFVGEGTHGDFFGLNVVDTDEHRDITIDITHGVPNTTSAELYK
QIARGRGKAVFQGKINVAIDAQKTDAKMMTQGLMLSDEAEILSKPELEIFADDVVCGHGATCGDLDETSLFYLMSRGISRADA
ETILIRAFLEEITGAVEDEYVSEALAGVVERWLKKDVLDRMEHAYRHEYANVHRGLHTLANRATEAFEGAREKVRAFLNAERV
EEIIFTRSTTEAINLVASSFAGPRIGEGDEIVISIAEHHSNIVPWHFHRERKGAVIKWVDVADDGSFDLDAFTAALTDRTKIV
AITHMSNVLGTINPAKQIVEIAHARGIPVLIDGSQAAVHTKVDVRDIGADFYVFTGHKLYGPTGVGVLYGKYDLLAAMQPFLG
GGEMIEEVSQDAVTYNAPPHRFEAGTPPIVQAIGLGAAIDYVEAIGRDAIAAHEHEVAVYAGEQLSRINSLRMFGIAPSKGGI
FSFMLENAHAHDVSTILDRYGVATEAEAKSFVEGGNALPTEDVERITADLIAALKTVYDPEIPVDIYELGLIYKVDLDDDRNL
TIDMTLTAPGCPVAGEMPGWVENAARSVEGIQDVEVKMVFDPPWGPDRMSEEAQVALNWW*

>jgi|Caulen1|89441|g4767.t1
MWNWNNIENLCSTGHFAWKGVECDGSGSVIGLDLQELSLQGSITGKLALLDSLVTLRLRGNLFTGQVPDLTELSYLKILDLSS
NELKGEFPFSAHAFWATPAGVSLEEIYVQGNKFSGYFVDDDAVSIEESSIRIISASQNHYHGKLPESWFRLVHLEQMDLSNNR
GLCGEIPSFQNGVSIPEDWLSSQICEIPKSSAQEEATLLLSLKNELAPQSSVEQVLKNWRPEKSEELCESWERVECEEGRVFL
TSTSIGVPCDENLEVSWMATEVLPTNNDQVTQITTSLVVPEVAQAPQEFLNVSQDSEDSPMDAMQSSPAYTETENSAIVPSPE
PELEISGFDGRKLPLTVSTTDSSNGVDSGEDSFEEKKGLSQHAIIWTIVSFLLGILLVILIFLCIHHRNQRDPNKDSKESRTW
KMRYSQFMSSLSGLIANTKTKSGSGKEGSSNGSKKPKEDVESPPTTSSVLASDHDSETNKNVSELGKLASIDSFAPLRRMSFS
FSRKNKAVQLQNTTADRVQDGTTDMALFGIQTDDSRSRLTLDTNDSQQNTYDNPLSSNDEPMATNLDLNDEVFNDCGVMFKNR
AFSEQTLNSEPSVTHTGLQRNYNDFHSIDEEPIEGAKSNHPPERLKTRQPDFVVNMGRCLLTQCSRGYFGDLGTLTQTTSDTM
GLRPHSTDATELNLNSKSTTWSITEDLGGTRTTISEPIMITRELTGSGNDVEDTKSIQIVSGRRIRRPFHTVCQSSTGVADSI
GDKSSAEKADLQKLLNKPYKWGFKTIIESETFPKGLSEDVVRAISVKKEEPEWLTDFRLKAYKRWLTMKEPDWSDNTYPRINY
QDLSYYSEPKQKEKKQSLDEVDPELLATFDKLGIPLNEQKRLTNVAVDAVFDSVSIATTFREELSKVGVVFCSFSEAVKEYPE
LVRKYLGSVVPVNDNYFAALNSAVFSDGSFVYIPKGVHCPMELSTYFRINASETGQFERTLIVAEEGSYVSYLEGCTAPAYDT
NQLHAAVVELFACEDSEIKYSTVQNWYAGDEEGKGGIYNFVTKRGLCHGNNSKISWTQVETGSAITWKYPSCVLRGDNSVGEF
YSVALTNNAQQADTGTKMVHVGKNTRSRIVSKGISAGRSRNAYRGLVKIAPTAKNARNYSQCDSMLLGDNAGANTYPYIQVRD
PSAQVEHEASTSKIGEDQLFYFQQRGIDLEEAVGMIISGFCREVFNELPLEFAAEVNQLMSLKLEGSVG*

>jgi|Chlat1|2051|Chrsp17S02525
MAAAMTAAAAAVPAERLLFRAPATRPESRTKTELSAKRSSAFGSPLSLPQSVSTRQQPVSSTRAEVIQEPAPMPVLPKKS
ASDDTISKFLEREYKYGFVGNIESETIPKGLNEETVRMISAKKEEPDWMLEFRLKAFRKWQTMKEPTWSDNKYKPIDFQA
ISYYSAPKNKPTLDSLDQVDPEMLKYFEKLEIPLGEQKRLANVRQVAVDAVFDSVSIATTFKKELAEAGVIFCSISEAVK
EHPDLIKKYLGSVVPIGDNYYAALNAAVFSDGSFCYIPKGVTCPMDLSTYFRINASETGQFERTLIVAEEGSYVSYLEGC
TAPSYDENQLHAAVVELYCAANAEIKYSTVQNWYAGDKNGVGGIYNFVTKRGLCAGKKSKISWTQVETGSAITWKYPSVV
LAGDNSIGEFYSVALTNNHQQADTGSKMVHVGKNTRSRIVSKGISAGQSVNCYRGLVSIGPNAAGARNYSQCDSMLIGDN
AGANTYPYIQVREPSAKVEHEASTSKIGEDQLFYMQQRGIDTEQAVAMIISGFCRDVFNELPLEFAAEVNGLMSLKLEGS
VG*

>jgi|Mesovir1|3765|Mesvi232S04464

MAAVHLNATAQLAMAASVCAAPAASQVFRRRGEVLSKGGFRGASLASRPRASGRIVYKTSRKCVATSLPEVVEEESEDMK
TISKFLDREYKFGFVSDIESVSIGKGLTEDTVRLISAKKNEPEWMLDYRIKAYRKWLTMDEPQWSDNKYPKINFQDIIYY
SEPKFKPSVDSLDQVDPELLRTFDKLGIPLGEQKRLANVKPVAVDAVFDSVSIATTFKKDLAKAGVIFCSISEAVKEHPE
LIKKYLGSVVPVADNYYAALNAAVFSDGSFCYIPKDTVCPMELSTYFRINASETGQFERTLIVAEENSHVSYLEGCTAPS
YDKNQLHAAVVELYAASGAEIKYSTVQNWYAGDKDGLGGIYNFVTKRGLCAGARSKISWTQVETGSAITWKYPSVVLAGD
NSVGEFYSVALTNNHQQADTGTKMVHVGKNTRSRIVSKGISAGNSVNCYRGLVQVNPGATGARNYSQCDSMLIGDKAGAN
TYPYIQVRDPSAKVEHEASTSKIGEDQLFYFQQRGIATEQAVGAIISGFCREVFNELPMEFAAEVNQLMSLKLEGSVG*

>jgi|Klenit1|10541|rna-KFL_003990110
MEAVASPALHTQAAAAGFGQRTHGTHVQTQVRTQAALLPRQQRHPKLHSFGSAKSIAGQKVCAESEGFRRDRKGRGHARA
AGTQSPTRERPSVKEAAEKIVGGLGGGLDEVKLDEVAGQVAKETGSEKAQISEFLKREYKAGFVTDIQSERIPRGLSEET
VRTISAKKNEPQWMLDYRLRAYRQWLKMEEPRWSDNKYPRIDYQDIVYYSEPKKKDVKASLDEVDPDLLATFEKLGIPLS
EQKRLSNVAVDAVFDSVSIATTFRKELAQAGVIFCSISEAVREYPDLIKEYLGSVVPPGDNFFAALNAAVFSDGSFCYIP
KNVECPMELSTYFRINDSDTGQFERTLIVAEEGSKVSYLEGCTAPSFDTNQLHAAVVELYAHKNSEIKYSTVQNWYAGDE
EGVGGIFNFVTKRGLCEGKNAKISWTQVETGSAITWKYPSVVLKGDNSIGEFYSVALTNNRQQADTGTKMIHVGKNTRSR
IVSKGISAGNSVNCYRGLVQVAPTAHGARNHSQCDSMLIGDQAGANTYPYIQVKDPTARVEHEASTSKIGEDQLFYFQQR
GIEAEKAVGMIISGFCRDVFNELPLEFAAEVNQLMSLKLEGSVG*

>jgi|Mesen1|10608|ME000086S10145
MGRKDRLQRGNWRCEAAEGGVLAPERTSGREKTSSDETIDNFLKRDYKYGFVSNIESVAIPKGLSEETVRIISAKKKEPE
WMLEFRLNAYRQWLKMTEPKWSDNKYPAIDFQVLVLVLVLVLLCLCLWLAVDQGVYGGGFDEVNAGGKTRVSQCCPSTSY
TSDWGWAGGGGGGGDSAAGGGIQDVVYYSEPKMKEKKASLDEVDADLLDAFEKLGISLSEQKRLANVAVDAVFDSVSIA
TTFRKDLAKAGVIFCSISEAVREYPELIKKYLGKVVPVGDNYYAALNAAVFSDGSFCYIPKDVTCPMELSTYFRINAMET
GQFERTLIVAEDNSYVSYLEVKDPSARVEHEASTSKIGEDQLFYFQQRGIDSEKAVGMIISGFCRDVFNELPLEFASEVN
QLMSLKLEGSVG*

>jgi|Chabra1|342956|rna-CBR_g37549
MAAMAAMAAFAATAGAPKPTPAPALVAYSARGVGGEGASSVSAPVALIARKPGALSVARYEGSACSLCSSSKSSSFLGGS
AVFLPNKDKSKSKSSSLSFTPPSSSSSSSSLSSSSFSSIPRAAAQPQSLIPAESSTPSSSSVLDKPEAEDDVIQQFLKKD
YKWGFVTDIESDSIPKGLSEETVRVISAKKKEPQWMLEFRLNAYRQWLKMREPTWSDNRYPPIDLQNVCYYSEPRQKEKK
ASLDEVDPELLRTFEKLGIPLGEQKRLANVAVDAVFDSVSIATTFREELAKAGVIFCSISEAVREYPELIKKYLGRVVPV
ADNYYAALNAAVFSDGSFCYIPENTISPMELSTYFRINASETGQFERTLIVAEKGSYVSYLEGCTAPSYDKNQLHAAVVE
LYCAEGAEIKYSTVQNWYAGDEEGKGGIYNFVTKRGLCEGKGSKISWTQVETGSAITWKYPSVVLKGDNSVGEFYSVALT
NNRQQADTGTKMIHVGKNTRSRIVSKGISAGKSVNCYRGLVQVGPTAHNARNYSQCDSMLIGDTAGANTYPYIQVKDPTA
RVEHEASTSKIGEDQLFYFQQRGIEMEKAVGMIISGFCRDVFNELPLEFAAEVNQLMSLKLEGSVG*

>ALG63564.1 putative ABC transporter (chloroplast) [Guillardia theta]
MEEIQRSANTRTEESDFNVKKLIAQPYKYGFTTNVENEEFPRGINADIVRLISEKKNEPSFMLDFRLRAY
AQWCKMIAPEWACVKHNSIDYQDIIYYSAPKYKKKLDSLDEVDSDILDTFEKLGIPLTEQKRLANVAVDA
IFDSVSIGSTFREELSNVGVIFCPISEALAKYPDLVKKYLGTVVPIGDNYFAALNSAVFSEGSFCYVPED
VVCPLELSTYFRINNQDSGQFERTLIIADKNSKVSYLEGCTAPQFDKNQLHAAVVELIALDNAEIKYSTV
QNWYAGDTKGLGGIYNFVTKRGLCAGKKSKISWTQVETGSSITWKYPSCVLVGEESKGEFYSVALTNNYQ
QADTGTKMIHVGKKTKSKIISKGISAGNSRNTYRGQVKISGKAENSINYSQCDSMLIGDKSQANTFPYIQ
VSNATSKVEHEASTSKIEEEQLFYFLQRGIDLESAISLLISGFCKDVFSELPMEFALEADRLLSLKLEGS
VG

>jgi|Emihu1|454654|estExtDG_Genemark1.C_1180073
MLTACIASVGAAYLPSQRPAPGAPLCGSARGRAAVRCAEAGTLTQSAVDDWLGSAVAARGSVASGLLDEVRSEATDVVPP
EVREAVLESLREIPEREADPRTELGCHTFAALNQAALSDVACVYVPSGVAVDTLRLILLSTPSYPDSGAEAGASPQLAAS
HPNLSLWLGDGASLSLLQQYAGRGAYFCNGLSRLRLGENASLTHAYLQEQSDGAVHVDSVLATLAAGARYDANIVQSGGR
LSRVNLAVRLEGRHSHASLNGIALGSESQLLDLHSAVRHVSPDCSSEQEQRNALAGRARVVFRGAVQVPHGSDNTTANQL
CRSLLLSDSARVDISPNLEILTDDVVCTHGATVADLDDEMVFYLQARGLGRAEARVLLLEGWARSLMGEAMSSI*

>CBN77201.1 FeS assembly protein SufD [Ectocarpus siliculosus]
MERTAQRSRNGGSESRRVSTLLAVAAGLLSLAPTADSFLSAPMKGAGGSGAAQGLRRRSTGEARSAQAGRQGDGVLSATG
VATEDNQGAVAGSLLETQQKGVSNNEMKPFVRVPRRDPRMWFDAIREGAVPRPDSLVELSKAGEAELAELALPTRKQEPY
RYTDLESLYRTDFTSAAPTTTAAETAAAIKPHLLEASQGQQMVFVNGVFSDELSDVSALGGVEGLVAGHIGAIEGAPLDQ
AKEMLSYLPEKDADFRTTQGSLAFASLNQACFADAGVIMVADGVTVEKPIQVIFFSEGEEGPTVSHPRLVVKAGADSHVT
LTQSYLSRGGVCLANGFTRVLVGDRATVTHDYAEEMSASDRLVDTVSVDQTTNSTYSVNQVLSGAFDARANFQIDLLETG
SHCNIFTAALTTNNQRQDVHSTIRHKAEGTTCLQEQRNVVSDTADCVFKGRIVIDQVAQKTDANQLCRTLLVSDKARVTV
MPSMEIIANDVKCTHGATICDLEDEELFYLMSRGISKIQMGFHI

>XP_002184318.1 FeS assembly protein suf [Phaeodactylum tricornutum CCAP 1055/1]
MKFTSVTLACFFFVESSGVSAFTFSTPTQRQRSIPLPIVSSSRRTRSALHVSIGLGPDPNETSGKRSETLVAGVDYEVPN
HDDYRLSRRSKLDQTCDEWFAGLLDETEDGVLGSLVTDARTVLTTPVPLVNDLENPDSSHDDWTPYVNTRLPWTPLVPAF
GLEQFGLPVPRRNAETWRHFDVASMVAESYSSAVKHDHEAKVDKIELFREQLAVAGGWLDDNACEARLVYINGRFVPQLS
KSNDVARNLDSTDGLSKQSVQYLSRLTDGFTDELASPVPNNDILETSYARLSRPNHNLGNATTQLAVNSQQGTACFAALN
TIKTGAVAYVHAPKGYNHIVADEQGDENDEEGPTRPNLKPVLIVNAVTKSGDAADHAKGVACHPRTLIVAEESSLLSVVQ

SCVDLDDNSAHTPKLYNGYTQCFVHQKANVTHSYLEESGGIVTAGVERSDDEFVADETKPREIESRRKDLRDTHLEAIDV
HIMGADAAYEGTIMSLGGSGRVRVALSVALLQPGAHAGVNGFSLAGGAQRVDIKTNIHHVAQGTTSRQVQKNMIGGRATG
AFRGRIRVEQSAQQTESEQLSRTVLLSDKARAWAVPSLEIIADDVQCAHGATVSDLSEEELFYLRSRGMGQAMARNLLMY
AFSGDVSDCVDPAVLGGFDGNAGLRQRVIRRLENLVPQGERAIKGEFQSI

>jgi|Nanoce1779_2|575559|fgenesh1_kg.2_#_2054_#_TRINITY_DN1698_c0_g1_i1
MNMQHRPQRRQWGASYLFATAALLGTVQAFLLPFNTPAQPPPSLAKDSGLWWQQQRQQGRSTKLQAALAERPRSPSVTLD
DSTALIEAETVALKPPSREEQARDWFNSVLSSREPNPLFHAATELGVGRLVDGETRAPTRKEEAWRFTDLSRVYSRKPGK
APAVLQPAQVALIKEKVKEHTLEVCEGRALVFVDGVFHPEFSPAGSVEGLKEEGVREGWKVGSIFDFTGEEQEELLREIE
WSPEVGMRHTLAAGSLPFCSLNQACVKDAACLVVGERGEMAAPVQFLFISTSTEGEGGKEGGKEARVSHPRLVVIAKKRS
AGKFVQSYVDIGEGVGLANACTTVRVDRRAEVQHLHANEMGGKGQLFDAVSVTTKKLSAYRNVFINVGGDMGRINLQATM
NGTEGRVDTEGLTVSGEEQELDIHSYIHHLRKDGKSNMYQCNIVSGDSHCVFKGRMKLEPRARGVTADQLIRSIMLTDSC
TVDAMPTLEVCSGDVDCTHGATVADIDENEAFYLQSRGIGRREARQVLMMGFAAQVYNQVPCERMRKRLSDRLYEVCPEV
GMAESYEYLSI*

>jgi|Ochro1393_1_4|710221|e_gw1.51.30.1
MPRIVLLSLIFLQLTFISRVSGYVIRHSVTPFCNIRSNGRKQAAIAIRGKNSFRIDSTSLLNTSETTDSGEEDTISNFFR
KSSSISSNNEMVNLANVAGKELLSGMKRPYGKDEAWRHTNLRKLFAYNYKRPEVDYAAKLSRSSVEHLIDDACKESCLVF
IDGAYASKLSSISKIPENVGASSLLHGIISSSGGAVQVEEMLKHVPDEKELPRDTFASDSLTALNMANMVDSAYIHIPTN
TVVETPIQVLFCNTVAGSATSNAEEAAEAFGTYPRLMVRLDSGAELQLKQSFATIPVDSASQTPIEIEIEPGGGPGFVGS
FTRIVLAEGAQLTHTYVQEVSQASRLVEVLAASVSGNSSYDVAAVQSGAVIGRINAHIELTDSNANCSLSGVTLAGKRQS
LDIHSNILHDAPAANSEQQQRNVIADRGEAIFKGRIQIPKHAQLTDSAQLCRSIMLGQRARIIAMPTLEITADNVVCSHG
ASVQDLDENSMFYLAARGVDRQEARKLLLRGFVFDLLKENVMDARSSQRLIRKLESMYPKQYSDVVDPSGGSAQKFMSI*

>XP_009032168.1 putative FeS assembly protein [Aureococcus anophagefferens]
MRNRLGAGCWCALNAACAGDGVVVWAREDAAAAAAAAARDDDGARVAAGGAAAAPARLHVVHVRSAGATGVLHPRTVVHV
GDGARVALRESFVAVGPAAEKRSRLTNARTNCRVGAGGVLTHVLDSQEADQVHVHHVQCGVRRDGEFRSRAISDGAAVGR
VAVEAEILGPHARFDFLGLQLGAGDQNLDLRTALRHTAGDAESVQDIRNVAGERAKLTFKGRIAVPVTGQKTNADQICKS
LLLDDGATVNAMPSLEIVADDVKCTHGATIADLDEEGLFYLNSRMLSEKDARKLLLKAFCYDIVQRTDLLPPATVDRLDA
KLSQLTV

>jgi|Vitbras1|16540|Vbra_21980.t1
MVVLEALIGHAVSISDLSSSAPASLRPAFIGTAGGWRSLGHTGGRDLLLRARRRRNQHHQRLFSMSTAEADTAAKEKEQP
PAAKEGGEGEEDGVDTESSSYKLVKRLEQERVSGLINDMVDPNYRREDYADRWAKEDDPEYVDSESPNSWERLRWKEEGK
LRFEDWSQFDPKRLEDPEYSLPEDWSAEPVGGRHNIFQPTLVTSKDLEKAVEQLDGGVVKVDEAPVDESVPPQYRGLMKE
VGFGFGYRNISMNKADIQRLMWWDKMLELQPLPANEDFEKLHENGTAVMKELYVPERKREPWRFQKDLETIYKTDFDVAY
GDLSSTEDISEYVMNDTAALLVIKDGLVDRRLSRLDGLPNGTFVGSIHNMEGALRERFLSELYWYPEYSQQQFKKGAYVK
YKGKLWKNADILPQQIFEMGKFDIGMAKLCALNMAHLRDVACVHVPEGTTIDKPIQVLVVSTSVGDSALPISSPRLQVLI
GDKADVTLSQSHVGLARKGFINSVTRVIMGRESRFNHDYIQEAGRDAWHFEQLSVLNAQAANYTYRSASWGAQSSRINVQ
VVANEKESRTDLRSLLLARQKQLLQTYQTVWHNAKDTYSDSFHRNIVADSATAVWKGRGRLEHMCNGADAKQLCRSLLLN
ERSKAVSIPVLEMMSPQIKAAHGATVSDLSQDQIIYLRTRGLSDEVARYLMVKGFANEVSNPMVDETAKRRLSRHMDSIL
SDPSKKKEPVREEVAAPAA

>jgi|Symmic1|37384|rna26446
MASLTLEDVKRALLNRSNVFVVALWISQACLSVSHKRVSIVMKESPFVLTILQPVWIAVAFGIVWAAMCFRSQEKEESST
DTEAELSESDTSSEEWTAKSIGAVCLLLAQAFPGALSTVFMEYNLQRSHVSFWKLWTLINIWEVVALPPVWVASVPLQGL
TFAQSPKNLLDGFQCLFVGQGSVEGADDEEIWLGTALVVAGMVLFRVKEAVKFGELTGVMLADTKQQLDLHSLIHHSVPS
CRSKQQHKNLVGGSAECIFKGTIRVDAEAGFNMRLFTPRALLPNFARSLLLTKKSKVKAMPSLQIQADDVSCSHGAALTQ
LDKDELFYLASRGLDGRDARRLMLAGFPTDLLDGLKDFAPKVHERVLGKLASMAESDD*

SUFA1 orthologs

>Cre06.g299350 SUFA1
MLVHRQCTPVRVQPPKHLARRAVLLPARRGVAVARAEAAPGLVATEAPPHAITLTPEALVQLRKLRADFKEQSEAMLLRVGVK
QGGCSGMSYVMDFESQDKVTDDDHVISYDDGFRLVVDPKSLLYLFGMQLGYSTALIGGGFQFQNPNATDSCGCGKSFGV*

>jgi|Galsul1|1190|XM_005702494.1
MNTYIINITESALEQLNSISKLNKKDKLYIRIGIKQGGCSGLSYFMNYEKQSNISDKDLVYNYDNFTLVCDNKSILYLYGISL
DYSSSLIDGGFKFLNPNAKQTCGCGKSFS*

>jgi|Cyapar1|19701|g19315.t1
MAFVSVPVPVPALSVPALSVPRAVCMRATTTAAPRRVHAAPGSSSSVFAGVRFEARRSVRARRPNLWLPAAASAEPAAAEAAP
ASDAPEIPEEPMVLAEGTEDMAIKISERAMAHLLTLKEKFTEDTCLRFGVRQGGCSGMSYTMDMVKQGETREDDRVYTYPNGF
QVIVDPKSLMYLFGMALDYSDALIGGGFDFKNPNATSSCGCGKSFSA*

>jgi|Caulen1|96705|g2901.t1
MGHLKKLRAEHQDSTMLFRVGVRSGGCSGLSYVMEFESENKISKDDTVFEYDEGSFRMICDPKSLLYLFGMSLDYSDALIGGG
FQFHNPNATETCGCGKSFGV*

>jgi|ChlNC64A_1|20890|e_gw1.5.685.1
PPITLSDAALAHLKKLREDSGDKQLLLRMGVKSGGCSGMSYVMDFEKAENVKKDDAVMEYDDGAFKLVCDSKSLLYLFGLRLD
YSSALVGGGFQFLNPNSTAECGW*

>jgi|Tetstr1|447496|TSEL_034877.t1
MASKFQVLTLTEAAAGRVREIIENSDKDALGLRVGIKKGGCAGMEYDMSLVEEAKPGDDVIEDKGVRIYVDPSAVLFLLGTEM
DHEVTKFRSGFVFRNPNEVSACGCGESVSLKAADADGFQPANAPG*

>jgi|Chlpri1|5434|rna-gnl|IITBIO|A3770_09p54340_mRNA_A3770_09p54340
MSSRGWTRARSTAGEEQAGHLAPCITLTDDALAHLKQLKEQKKSLLDQGLQLLLRIGVKQGGCSGMSYFMDFETPDKVVGDDA
VMDLEGDMKLVCDPKSLLYLFGMELDYSHELIGGGFKFTNPNAESTCGCGKSFSV*

>jgi|Pico_ML_1|52567|g3254.t1
MDFAEKESVTDEDSVLEFGDMKIICDPKSLLYIFGMELDYKSALIGGGFAFRNPNADKTCGCGKSFSV*

>jgi|MicpuC3v2|1188|wlab.210138.1
MGALGRVLRGALASTSRRLVSAPSRRAVGGDAFSSSSSSSHRAWSSSIAPFASESAPSPSGATPAATARDDLALTEACVRRLR
ELAASSSGITEGGAESKQPNDDGAPLLRVAVDGGGCSGFQYSFSLDSAPGVKPNDRVFERDGARVVVDDVSLTFLKGATVDYV
EEMIKSSFAISDNPNAESGCGCGSSFVAK*

>jgi|Praco1|19821|PRCOL_00003093-RA
MAASNLLEGRGAAFLKLGVRTRGCNGMTYTMNYADEKAKFDEFTFDNPNAAGSCGCGESFNPIENPLKIRR*

>jgi|Chlat1|6140|Chrsp41S00446
MSYVMDFEDPANVRDDDSLIEHEGGFRMVCDPKSLLYLFGMQLDYSDALIGGGFAFQNPNATSSCGCGKSFSA*

>jgi|Klenit1|11643|rna-KFL_004960060
MASTACAMRLGALSAPSINLHPRQYAPHQSIAVLNLNRNTPSGLRSIRSKGRITCSVATPSPPTLPPAISLSEKALAHIKRMR
NESGKDLTLRVGVRQGGCSGMSYVMEFEERANIREEDSLIDQEDFTMVCDPKSLLYLFGMQLDYSDALIGGGFSFQNPNASST
CGCGKSFAA*

>jgi|Mesen1|643|ME000108S10791
MAALTASAGITLSSLSTSALGTSQSVRKCSVDVRRPLALNLTARSQHQSSRRRSFVVRSSDTASAISTLTSPAPAVTLSERAL
AHLVKMRSELNKDLLLRIGVRQGGCSGYSYIMDFEERSNIREGDTIIDHEGFAMVCDPKSLLFLFGMQLDYSDALIGGGFNFS
NPNASSTCGCGKSFSA*

>jgi|Chabra1|353188|rna-CBR_g72684
MGGPRAHSTNIGRSILLRVGEIPTRRDIELAGLEVYMKVATGTAPAGAAAAPAITLTDTALNHLMKLKEGQNNKELCLRVGVR
QGGCSGMSYVMDFEDRAKIRAEDAVIDYEGFTMVCDPKSLLYLFGMQLDYSNALIGGGFSFSNPNASSTCGCGKSFAA*

>jgi|Guith1|90502|estExt_Genewise1Plus.C_1470001
MKSRMLRNPKMTTRLLVPLLLLLLAGYCNAFLSNPLSLTRISLSTKVCSVRREKIHFAGLRMIEDTSTDECEGPAREGKVGNV
DISNSRWNQDEIKTDGIIRLTQPAISQIQKLRTNRGEQEVVLRVGVRAGGCSGMSYVMEFEDAKNVDESDTELKFEGFRVVVD
PKSLMFIFGMELDYSDALIGGGFKFSNPNAASTCGCGQSFGA*

>jgi|Emihu1|61273|e_gw1.3.68.1
MRLILLVLFASLRAASSYLAPPPAALTRTRLAVVSSGGAETALPVRARALLCARPTLLRACPRLDGGAAQDSIISFSERALTQ
IQELREKSGKERACLRMGVRAGGCSGMSYVMDFMEEGEIDEKDTTIDYEQGTIRCVIDPKSLMFLYGLQLDYSDELIGGGFSF
ANPNAEETCGCGKSFGV*

>jgi|Bigna1|50664|estExt_Genewise1.C_880018
MMKEHLLLFTLTTIVTFSKSADATRVRLRSGTMNSLRTQRYIKLGESHFAWPQNRMIERINVPSRQSRMPLDCKCEQLGVGGV
LSAAEAGIIQISPNAEKRIAELKVSKGLDEAFFRVGVKSGGCSGMSYTMDIVGSDAVEADDTVVEYNGFKCIIDPKSLLYLFG
MTLDYNDDLIGGGFKFFNPNAKDTCGCGSSFGV*

>jgi|Phatr2|14867|e_gw1.16.211.1
LVLRMGVRSGGCSGMSYVMDFAKAADIQEDDQVDEYMSDRIQCVVDSKSMLYLYGLELDYSNELIGGGFQFFNPNAEESCGCG
SSFGV*

>jgi|Ectsil1|29259|rna12779
MRGLTVVAFAMLASMIKGFIVAPTPGSSTGSCRSSRVSGATTIRSHASAALSMSSTGVEDEVMEKIVNIRPKAMDHLQELRRS
QGTEGDIFLRMGVRSGGCSGLSYIMDLVKKEEITEDDMVEEYDGFKCVIDPKSVLYLYGLELDFSDALIGGGFQFQNPNAESS
CGCGKSFGV*

>jgi|Nanoce1779_2|596574|fgenesh1_kg.17_#_382_#_TRINITY_DN6595_c0_g1_i1

MHRPFALFSVGHAKAMLMLLLGVLATTQAFLIPSAPFTLSSSTLAPLSSEKNEGTVEVAERVITVSDSALEHLKDLRTKQGVD
HLYLRMGVRSGGCSGMSYVLDVMKKEDVTEEDHIEAYDEHDFSCVVDPKSLLYLFGLRLDFKDQLIGGGFAFLNPNAQESCGC
GSSFGV*

>jgi|Ochro1393_1_4|696524|e_gw1.6.362.1
MTPIQCFATRARPNARARPPPIELTERAAGRIKELLSQKPDSVGVRLGVKRRGCNGYSYTMNYADQEYVESTKDEVVSEHGVQ
VLVDPKAIFFLVGATMDYVEDTLSAEFTFSNPNEKGKCGCGESFNV*

>jgi|Auran1|21736
CSGLSYSMDLCKTEDIVPEDHVELWEDDLKVVIDAKSMLYLFGLELGYSNELIGGGFKFNNPNAEESCGCGTSFGI*

>jgi|Vitbras1|10875|Vbra_585.t1
MWNLFLILGAAAVLPSEAARRFLQSRGSAAFVAPPASVNAPRRPVLLRRALAVRSSGASTSRRAIDITPKALERIAELRAKKQ
RDGDGDLVLRMGVKSGGCSGLSYTMDLIDASDVGPRDTVEAYEDEGFKYVVDPKALLYLFGMQLDYCDDLIGGGFRFDNPNAD
KSCGCGMSFGVPTQLTAMAKEKDGVQSQPGSCTTTKN

>jgi|Symmic1|38402|rna27132 (corrected)
MAAAAAKQIMSVTKSAASRLGTLMARHSDPNAIGVRIGLRQRGCNGMSYTMDYTDKVNKFDEVVEADGGIKVVVDSKAVMFLI
GTEMDFVSNEVGNEFVFNNPNKKSECGCGQHDRQSHGLESTTQ

IBA57.2 orthologs

>jgi|Galsul1|6574|XM_005702813.1
MSSNHFLNSQPFESRWTCFLTSTGRILDVALVLVLKDSILVVSSIEKKQMLWEHFDKHIFPMDNVSVTEENYASFVWIGKQAV
DWIQNWYQQLGMNGDQEQFQVFDHTNHPIYLLQQSTLEPKWKGYLLLCSAEDIHKVHASMSSFQKKGFLLFHMDKEHWECLRI
EMGKGNVLCEWSEQYHPLEAGLWHMVSFQKGCYLGQETILRLKTYGGVKRYLVGWFLEYPVETPSHVYCQKRRVGNITSCKTI
QKTTTRESQPQDMTVVIGLGYLQSEYATLEYEMDKPLERIASIQDLPWESTIFFSKVPGQTSTPVKCIVRRIVFPVWH*

>jgi|Cyapar1|108|g104.t1
MAFSVTPAPTALRPGRALQLSSPAVCSGPTEREATRLWEQRRRFVLGDQPLRLRATAAAAPHQRSFVPANPVMHVEHPHDVVK
TVEVAGVIDGTPEERLWARQEALGAVAGEPNSDSAWVTAETFGEEKDEDAIRAVGMGVALFDRSSWGRLKLSDADCVAFLHNQ
STNDIQKLKSGQGCDTCLVTAQARLIDLALALRTDDNIILLVSPNKQKALAKHFDKYIFFQDKVKVEDLTSKTACFTLIGPSA
RDVLGRMSIDVRDMCGLTYAHKFFSFQRPSGRMDVMLVRNSGLTAEGFTIVVNADQAADLWSFLEAQGARPLGTRAWNKLRVK
DGRPAAGLELTEEYNPLEAGLWHAVSLDKGCYIGQETLARLNTYKGVKTQIWGLTVSGAAAGGRRPAAGDPIFSEEGEKIGVV
SSVAEVEDKEGLACLAYVRTKVRQEGGRSIDRNI*

>jgi|Cyapar1|24870|g24308.t1
MLGSVVRRCAGAAVPKVATAVRPGIRALASAPAVLKKTALHDLHVKLGGKMVEFAGYAMPVQYPDGIIASHNHCRSAAALFDV
SHMGQLTLNGKDRVAFLESLSVGDFASLPEGSGRLSMFTNEQGGVLDDFIATNAGGYLFVVVNAGCKDADIAHMQAHAAKWRA
QGKDVSLTVIEDRSLVALQGPEAGAVLQRLVKEDLSRMSFMTSREMAVDGIPCRVTRCGYTGEDGFEISVPSAKAVAFFERLC
AEKEVKPAGLGPRDSLRLEAGLCLYGHELSPTISPVEAGLTWAIGKRRREQGGFLGAAVIQKQIAEGVTRKRVGLLIEGAPAR
EHTPVFDASGKQVGEVTSGGFSPTLKRAIAQAYVATPLSKDGTALQVEVRGKRSPAVVAKMPMVKTTYYKAP*

>jgi|Chlre5_6|4731|Cre12.g552850.t1.1
MLSMIQQSGPTVTQARRCGGSKSACSWQRRAGGAAACLSAACGAASSSEASVSGRCHIGVVPLAAPRRRRAGSVVRGPEPPRI
NIDDLMLDVPEIDGDIRSLQVEMGAIFNDAGLATTFGKKKQALQALETGLVLVDQSHWSRLRVSGDDRLTLLHNQSTQDFKAL
RPGQGADTVFVTATGRCLDLATALVLPSSVMLMVAEGTSDEAARGARPAGAALLERLNKMIFRGDKVAVQDVSERTAQISLMG
PEAEAVLRELAPDALAAVLGAPAGAHVLVGFRGKPVFVVAGSGLGPGVPGYTLIADESIGGDVYAAFAAKGAIPMGTDDWEAA
RVLAGRPLRGAELTEEHNPLEAGLYGAVSLNKGCYIGQETLAKLHLRDGVNRQLWGLALSGSTAPGTQITSELSKVGVVTSAC
QDAEGEWVGLGYIRSRIEGTQIALEGVRVAVGGTPATVTAIPFATRKLSAAAEAPTSSDTVSGRLEDAKKKKEEEAAAKEAAT
AAKLQAMQERLAAWQAQQKQQTK*

>jgi|Caulen1|96351|g2589.t1
MRTTFGRTTIPVSHPCTPFHKTLNAVFRIPRHSPLVSRSQNFDLPDLEGDVEYYQRELNGRFNSTGILLDFNDNTRVPNTAPL
LYDRSHFGRLKIEGKGVFEFLNNRTTNEFRNLHKGSGLDTLFLQSKGKIIDLATAYVLGNGVLLILSPTIQDALMEELDKFIF
PGDEVEIRDVRTETRIFSLIGQGSQELLSLLNVQDSVLDGSYGTNEISGFQNGPVIVAKGNELNRSEGYTLIVDDSVAGDLYK
NLIDAGGVPIGLLDWERLRVSNGRPGLETELRHASNPFEVGLFHAVSLSKGCFLGQESISRIYTKNTIRRKLWGIQFHSKEAV
VSPEAMIRSEGKDVGRVTSIPVDDVEKDSTQKICLGFLRCKINKTAVDWNGTEVEIEGFKGTVRHLDYTNNDFETGIGSPIPS
TPEGKTNVESVDKEKKLKEMQERLDAFMKQQKQQG*

>jgi|Tetstr1|440026|TSEL_028387.t1
MAVCTAASIGSGVSSNRTHTHAHTHTHTRPTTSAARRIPLAFRRVASRPSLCSGRRMRGSPRRAAEEDFDLDALLDGLEPPAL
EGEPRYYQEAAGGVFEGGIAVSFGKTDKDVPEAMRESVVLVDRSHWGRLRIGGDDRVSFLHNQSTANIAGLAKGQGTDTVLVT
AQARTLDVATALVQESGIMLIVSPGQAAALVERFQKYIFPMDKVEVMDISERCGMLTLVGPGSDELLAELGAGDVVGKPEGSH
TLLNFGGSPVIAAVTTGLRMPGYTLIVGEDKTGELWRQLAMKGAVPMGADAAAPAPGKELTEDYNPLEAGLCHAVSLAKGCYI
GQETIAKVHNLGAGKQQLWGLDAAAACECGEEVLSAGGAKLGAVTSATAKPEGGFFALAYLKCKVKGGSVGLAEGLPVTVGGR
PAKLAPLPYSSRALLPKDLPPSKDAAKEESAAAGEDDAAAAAKEAKLKAMQERLAAYQAQMAAGKKEAK*

>jgi|Chlpri1|3704|rna-gnl|IITBIO|A3770_05p37040_mRNA_A3770_05p37040
MRDLVGRSAGRAPTQRGCSPSCSRSSRRGKGRRASGRRFGSTRFGRSRVDWSARSLEDIDMLEVDGWEEVTLREIQEEGGAVF
AGGASVPTSFAAGDGNEGSGLTLGDLESAECCVFCDRSDSTVLQVAGGDRLAFLHNQSTASVEGAKPGDVVETAFVTSKAGLL
DVAKVLVQGQSALVLASPDGASQIIGHLDKHIFPRDDVRVSDVSFNLSVFAVLGAGSSKVMETLGMGHDMGEGEQVLVNYEGY
PLCVARTTDLGVPGYTWIVDTSISSDAWSLLTSIENVRPIGSEAYERLRILAGRPAFGKELTLDSNPLEAGLYERCVDVEKGC
SIGQEVIKRVHNRNGITRRLGGFSSDKPLRCGDEVLSEGRKVGSLTSVCQDGGGSFLGMGYVKVKPGQPFSSEGKSFTVRGEA
VAVRALVYPKYPQESVENKDAREEEESLEEIEEAKEKKKEEMKARMEEWLKQQSKNK*

>jgi|Praco1|21076|PRCOL_00004673-RA (no typical signature)
MACASDDGLEALLEELDLDIDLDELLEPPAIDGDLGELLSDAGAADVDAATGIALTYHNDVDAFDAARGAEDTSRVAVVNLTH
AARVRVSGADRGAFLRRETTHGGGVGEVALVSHGEGAGSVCGAALDAGVVVSQGQSELLLLPPESRADAAGALGARVRPDEDV
TVADVSGPTAQFALVGEGAADALRSLLGDELVDSPDVAAVLAEGAPAGLSCMAGFQGSPCSLIRAEPLGPSTPQLLVVTDAGV
TAEMFAALVQAGCVPMGSNCWEKLRVRCGRPRRGFELGVRGFERTPLEANLWHACSAGGAAAEEGEGEPSAGVAEVRQRAAAR
SMEYHLWSLELPVYVPPGDAVEVDGVEQGMVTSVVQTPGEGELWALAYVKARAGVSDGGEVRVGTRRAIAHMPEYGSRRLR*

>jgi|Chlat1|7157|Chrsp57S06830
MASWVGLGPSLPMFLSLPVACASRAAFSSLATTAVAPSLNGSQRRGLRSSSVCMPQRRWLPDQASAVRVSSRKGRSGVVANSL
GDALTAPPIDGDIKELQEVAGAVFGDDDIVETYGNDSAALEAMRNGLAISDQSHFGRIRVADADRIRFLHNQSTADFNRLKPG
EGCETVFVTPTARTIDLARAFVQANSVLLVVSPDMRHQLLPMLDKYLFFADKVKLQDVTEQCSMLVLYGPGSHEVITQLQAGD
MVGQPKHSHRLYNVNGSPVVVAVGTGLATEGYTLVVDTAVVADLWATLAGFGAVPMGRRAWTHARVLEGRPVPGAELTDEFNP
LEAGLWHTISTDKGCYIGQETIARLITYSGTKQYLWGVELESPVEAGTLITVDGEKAGKITSCITLDDGVHRGLAYVRTKVGG
SGLQVDAAGVKGVTVDVPYVTRTLPA*

>jgi|Mesovir1|195|Mesvi104S01524
MAVFCHATSFSLGGVQGVAHNSHKVANQNEARSRAYRHCMPARSSGKLCSFSGKASFKLMARKCQQSRREIIYRPGLVVAHSS
ADDLLAGIEMPPIFGDLRDMQEDAGAVFGDEDAGGPLTFGNDKEALAAARGSGFAVYDKSHYGRIRVVGADRISFLHNMTTAN
ISRLKPGQGCETLFVSPQARTIDLASLYVQESSVMLSTHPTKTAEILKIMDKYIFPMDKVKLMDVSDKCFSFALVGKGAAEAL
TSFGAGDILSKPAGSHALFEFQGNPVMLGVGCGLSVEGFTVVADAPIAGDVWSAFVQAGGVPMGEAAYEALRVAEGRPAPGQE
LTADYNPLEAALYSAVSIDKGCYIGQETIARLITYDGVKQQLWGLRLNARVQPGADILDGDQKIGKLTSCCEDIEKPGMFLGL
GYIKRKAEGMGAGVSVMVGTAPATLVDIPYASRSLPTA*

>jgi|Klenit1|9453|rna-KFL_003250090
MNAAAGLCCRFESLHFGSLCNSTPSPLSRKICLPFAVGPNSPSADLATRLPQQKGSFSFFTRDRTTFATTTPRKVLQERSGPV
AAAFGVDLLPPPIDRDLQELQEELGAEFSPEGVVVTFNNDADALEASKSGFAVVDLSHFGRLRVYDDDRIRFLHNQSTADFNL
LKPGQGGDTVFVTNTARTIDLVTAYIKESSVLLVTSPSQRRELLPYLDKFIFFADKVKLQDVTDQCFMLALVGPQSSQVLEDL
RAGDLVGKPYGTHAMFDFQGSPVMIAVGSGLATDGYTVIADAAAAGDFWKTLMQQGGVPMGARAWERLRILQGRPVPGAELTP
DYNPLEAGLWNTISLTKGCYIGQETIARLITYNGVKQHLWGIQLSEAVEPGTVITLDDAKVGKVTSVTETESGFFALGYIRSK
VCSEPGLTVHVGNAVGKVVEIPFVRRTLDA*

>jgi|Chabra1|335283|rna-CBR_g30837
MDAGVEVVDLHAYIAKIDREFKTQRYDDIDAPLLYVRIETGEATCIALIDCGASRNYMSQDFMVRAGLGPRVRRKSQPTQVTL
ADGHTHKSIDRCIDNVPMYFAPHASEAVSFDILDTKFDMILGMSWLRSEDHPVNFYRRTVHIRDRNGVLVPCTVAPPHPSVSC
HVVSAASMRASIIRDYIEEMGVCFLHALPPQDASSTDSSSDPRITRLLDAYNYVFEGPHGVVPDRPICHEIILEDGAVPPRGC
IYRMSEEELSVLRAQLDDLLEKGWIRPSSSPYGAPVLFVRKKNKDLRLCIDYCKLNEQTIRNAGPLPRIDDLLERLGGAKFFS
KLDLKSGYHQLEIRKEDRYKTTRSLEEHVEYLRTVLERLRQAKYKANRDKCEFARQELEYLGHYVTPQGIRPLADKIEALRVW
PEPTNTTDVRSFMGLAGYYQRFITGYSRIAAPMTRLQSPKVPFVFDDDARRSFQTLKTAMLMAPVRSIYDPTLPTRVTTDASD
YGIGAVLEQHDGVDWHPVEYFSHKVPPINSLDDARKKELLAFDMALKRWRHFLLGRRRFTWDIQTKYGAIFDEDSGGNVAVTF
GNDVEAIDAAKTHAVVIDRTHYGRIRVSDEDRIRFLHNQSTADFQILKQGQGCDTVFVTPTARTIDLATAYIMNSSVIVVTSP
SLRHELVTTLDKYIFFADKVKVEDITDRTAMLSIIGPKSGEILEDMKAEAVVGTPYGTNMHFELFGEPVTIASGAGLASEGYT
LIMTPDSAGLVWENLVGKGAVPMGEAAWEKLRILEGRPSPGKELTGDYNPLEAGLWKTISLTKGCYIGQETIARVITYDALKQ
QLWGLELEGLAEPGTIITAANSDEKVGKLTSFAEVDGKFFGLGYIKRTAGGEGMQVSVGGTKGLVVEVPYLTRSLTQ*

>jgi|Guith1|90067|estExt_Genewise1Plus.C_1150008
MLSVRSVAVVLLLSTTTTVSFNVRWSAVRPLSRSGSLPCPSRRNEPRTRMRRAGRQLVACSKDTSEQKDATQSLAAIAEMERR
RQQEEEMRNSGSIGWECLRDTPQSKQELRELQLKFGAEVVPATYPAPEPLMKFMKKGRDRVVDVVSKFSLLPQAEVIAASSSG
VLLADLSHWGALVVTGEDRYKFLSGLCTNRVVGLRPGDVRQACFLSKVGRTVDLCTIAVLSDSLLVLCSPNRVLQLFQDLDAL
IFPKDKVKCLDVSEGLARFHLVGPKAEEFLSQMPGITLPEPFCSSPLVLEGKSMNGLVLHGAGCSVPGYTIVAASGDGRVLWE
EFMKVAEPGPLRVGQEGWEVLRMVDGVPAAGSELTLEYNPLEAGLWSTVSFDKGCYIGQESMARLKTYDGVKQNLWAIQFPAD
KSGAKSSRALVGAKLFAVSACESGQSRRLKLACEGEGRGRKIHRDDHVRPPGALAHPR*

>jgi|Bigna1|83823|fgenesh1_pg.116_#_16
MAVRNCPTLLSPSASFSTVSDEEAQETRTCQGGMLEATCHGRRQHRLRQLALFKALLLLSVVVMVSLGIWSVAAVSVGELRRG
AVRRASLLPCRLASYIRGGVRRARSTSFAAPNAHAGDSKSEPTESKPPPPQITLEQLKERIRLQQEAAKRAMMEVKSGATTLA
GDKNDAELRHVLEGERAAIIDHSNYGIIEVTGRDRLDFLHGMSTNDIAALQPGAYVQTVFTTRTGRMIDSTVAIATEESVLLI
VSPGNAATLSEMLDKYILASNVRVRDASYDYAVFRVLGSVDLRRIAPATPMGKAQENGEARVEFWTPGNGGDDSSGIQNAGGG
KEEEIVAVHGETGGLVLPGMTLLVPTSSVAKVYAEIIKQREEEEEELLQIGLMGWEKLRVHQGVPKLGGEITESYNPLEAQLW
DSLNFIKGCYIGQETIARLKTYKGVKQFLQRFRLDAMPDGFQTAEDGPENDRDGGEIIHSSEGGEKVGVLTSCAAIGGQIWGL
GYVRTKANLEDGSRVVIGETEDAAGISAVMDTPLPRVGDNGFPEWLLQDDEAASPSQ*

>jgi|Ectsil1|18192|rna10292
MAPEPWSKVSSCLLACACIPLAVGLSPWAATTSRSTSFGSSSALGSSARLWAQRSSSSRSSSSGSGSSSCGTVSPGPGGRSTP
TCSLDDLRTAQSENGGVIGDVSYDDGHVLQNVLVSGFEAGTGARQRADAVADAAASGTLLVDKSHWGVIRVEGEDRLRFLHSQ
GTNAFERATVGQVVATCFTNNIGRVVDFCEGVVLDDAVWLISSPHRWQKLLGTMDKFIFPMDKTTVSSLSEELAVFSLAGPKA
AETMAMAKCASCPEPGRSVEWEFEGEKVLIIGHARPLTRTREEEEGVSHDAEGVLSGDGERQGTQQHPYYSVIVPVSVSSKVW
SALSASPSVVAAGEEEWQTLRIKQGFPFPGKELTADYNPLEAGLWHAVHFDKGCYIGQESISRVNAYNAVSKALYGVSFEDST
SPEQGTELFVQETGKSAGVVTSMLDRDATSHPFGLAYIRTKAGGVGLKLSTKEGEPLGTVVQVPYPTRSDAESAMPPAKKEGD
SGDKATAAAAVLGLEEEKAKEKARKAAKLEAMQKKIDALKARRKTS*

>jgi|Nanoce1779_2|600710|fgenesh1_kg.22_#_427_#_TRINITY_DN6865_c0_g2_i5
MTLLPILGLVLLALCLDSTVGFLQPHQPPHSNNRGAQRKCGSILGMLSVDEAMNMKEACGQAQGVLLDLTAGEWGLLSVTGED
RLRFLHNQLTNSFTDLKPGQVLESSYTSSTGRTIDLVTAYVLQDEVLLLSSPSRHEQLLGAFNKYIFPLDRVEVQRRDLSGLF
IWLGPASAAQTVLPQLLGNDDTLAPLPGPSQCQYCSSSSFSMVLAEGTGVALSGYTILALRARAASIPAGDVAAHVGRGGMEE
WQALRVLQGRPYPDKELTMDVTPLEAGLWHTVDLNKGCYLGQETLARQANNKGERQKLYAVRGKGLKERKRVIDAEGNRAGVI
TSCMNDMGLAYIRKTVLNPTTDMLFVDGEAQRIQVLDIPYATRAATFNKMTTPKEAAAAAAAVNTTAADVAAEAERKAKKLEE
MKRRLEAFQAKSRQT*

GRX3 orthologs

>GRX3 Chlamydomonas reinhardtii (correction in red, GRXS14 ortholog)
MASLQCNMRPATTILTRRPAQVARKSIGVARSQTVRVTASGMAPDLKKSIDELIASNKVVVFMKGTRQFPMCGFSNTVVQILN
VMDVPYQTVNILEDDAIRSGMKEYSQWPTFPQVYINGDFFGGCDIMMEAYQSGELKEQLEIALNS*

>jgi|Cyapar1|8129|g7870.t1
MAAFACAIPVGVRGPSSVLAQPARSSAAPAPAVRRHAARAFTASRSQFVGAAVARRSAPAKAFAFRPVEIKAEITPELEK
QIEDLVKNNKVVLFMKGNRLMPQCGFSNTVVQILERCNVPYETCDILADPDMRQGMKEYSSWPTFPQVYIDGEFVGGCDI
MLDLFQSGELQSMLEVAMNS*

>jgi|Caulen1|95844|g2143.t1
MKIVCFGRAPHLARFQRSRLKLPIFTSSPSFRQKRGATFAAAHGPGLPDELKLALDGLISQNKVVVFMKGTKQFPQCGFS
NTVVQILNTVNAQFETVNVLENDVLRAGMKEYSQWPTFPQVYIDGEFFGGCDIMIGNFLLPSIT*

>jgi|ChlNC64A_1|37075|estExt_Genewise1Plus.C_250008
MAAVAAVVGHSRLAVSPVSQPRGSAQLRAPVRQLAAGWPRSRRACRTQAVGQVPGTPAAPGAGLSPELREAIQQFITDNK
IVAFIKGTKQFPSCGFSNTVVQILNNCGAPYVTVNVLEDELLRSGMKEFSQWPTFPQVYIDGEFFGGADILIAAYTSGEL
AEEVERAINN*

>jgi|Tetstr1|427137|TSEL_017326.t1
MALATAPTRATASTARCPSVRRPAPATAPRHSSGLAAGLRLRPASGLARRGGQRSQRRADKPLRVSAMGPGVGLSPELRA
ALDEYVNKYKVVVFMKGTPQFPQCGFSNTVVQIMNNLNVPFQGVNILEDDSLRSGLKEYSAWPTFPQVYIGGEFFGGCDI
MIEEYQNGNLKETVERVLME*

>jgi|Chlpri1|3579|rna-gnl|IITBIO|A3770_05p35790_mRNA_A3770_05p35790
MEARVRARTRAGVGGWKRVACSCACGGREAGGQGRRREGRGVGRRDGIAARNWTGGQGRARTWSWRCEAQQGESPSGFTP
ELRDAVDRLISENKIVLFMKGTKMFPQCGFSNTCVQIFNQLGAPFEDINVLEDERIRVGMKEYSQWPTFPQVYIEGEFYG
GCDILLESFQSGELEEYIERVLAS*

>jgi|Pico_ML_1|54442|g4788.t1
MKGTKAFPQCGFSNTCVQILNTFNVPYETVNILEDESLRQGMKEYSNWPTFPQCYIDGEFFGGCDIMIEAYQSGELQETI
EKAMVN*

>jgi|MicpuC3v2|4834|wlab.217373.1 S14
MKGSKDAPRCGFSSTVVQIFTSMNVPFETVDILDDDGLRAGMKIYSNWPTFPQVYIDGNFYGGCDICIGAYKDGSLKEAVDVA
LMS*

>jgi|Praco1|16206|PRCOL_00004792-RA S14
MAPVVLFMKGNREQPRCGFSATSVQVLNACRANFETVDVLDDPSMRDAMKIFSSWPTFPQLYVEGEFYGGCDILMEMYQDGEL
QELIERVNMS*

>jgi|Chlat1|1422|Chrsp12S01982
MALAAAVAVVGTAARPLSVASSGRPQSARRPAPSARLQQSSGSFTPSLRLPLQRRRQQQEKRSCRAVAAGEPQPGLTSEL
KEALDDFIAKNKVVLFMKGTREFPQCGFSNTCVQILNIMGAPYETVNILEDETLRQGMKMYSSWPTFPQVYIDGEFIGGC
DILIESYQSGELKEMLERATVS*

>jgi|Mesovir1|7736|Mesvi7120S00833
MAVGRGGSRKVGASLPTELKQALDDFVNNNKVVLFMKGTRQFPQCGFSNTCVQILNTLAVPYETVNILEDESLRSGLKVY
SSWPTFPQLYVDGEFVGGCDITIELYQNGELKEIVEKAMAS*

>jgi|Klenit1|3212|rna-KFL_000610430
MSFMGKTQTVRRTAHSGCRASAGASSGGGSVLTPELRTAVEEFLQEHKVVLFMKGNRQFPQCGFSNTCVQILNMVQVPYE
TVNILENETLRAGMKEYSSWPTFPQIYIDGEFIGGCDIAIELYQSGELKELVEKALVS*

>jgi|Mesen1|6838|ME000351S05957
MASITGAVAHIAPSATSLCGSKSRSSVGRTHLSSVSVRSCSLTSSSQDLLTVRVGAGRRNNQRSISCQASIPDFKAPSAL
TPELKETIDNFLASHKLVLFIKGNRQFPQCGFSDGAVKVANALDKPYETVDILADETLRQAMKEYSQWPTFPQVYLGGEF
VGGFDIFYEALSTKEFQEQVEVVFAS*

>jgi|Guith1|84703|estExt_Genewise1Plus.C_80280
MSKAFLLTILGISITSVSSFSLQLPGCTRFAVRNVANSFVQSPRTAAHTSPLQLRMQLDDSNRKGPMWNAIDKVVKENKI
VLFMKGNKDFPQCGFSNTVVQILNACNARYEAVNVLENELLRQGIKEYSNWPTIPQLYVDGEFLGGCDIAIDMYQSGELA
EMIEKVMAE*

>jgi|Emihu1|69804|e_gw1.107.46.1
METTEDRIKAMVEGNKIFLFMKGSKLFPQCGFSNTAVQILNSYNVEYETFDVLADNAVRDGVKTYSNWPTIPQCYIEGEF
VGGCDLLIEMYQSGELSEMVEKAAAS*

>jgi|Bigna1|63721|fgenesh1_kg.58_#_40_#_4739_1_CFAO_EXTA
MKGTKDFPQCGFSNTCVGILNRLGVEFETVNVLENEMIRQGMKVYSDWPTFPQVYIGGEFYGGCDIMLQGYESGDLAETI
ENLFS*

>jgi|Phatr2|9035|e_gw1.1.958.1
MADDFLQETPDSTQNRIKNLVEEKPVLLFMKGNKLFPQCGFSNTAVQILESFKIDFETVDVLSDDKIREGVKLYSQWPTI
PQLYVAGEFIGGSDIMIEMYQSGELGEMIEKAKADMV*

>jgi|Ectsil1|32226|rna4822
MMSEDFGEEDSFGARAMQERTHAELQEMVSSHNILAFIKGNRLMPQCGYSGTLVNILQSLSVPFETVDVLADERIRQGIK
DFSNWPTIPQLYLGGEFIGGADIVIELFQSGELQEMIEVAAAS*

>jgi|Nanoce1779_2|616044|fgenesh1_kg.111_#_63_#_TRINITY_DN7382_c1_g10_i1
MKGNRLFPSCGFSNTAIRILNQLNVEYETVDVLADERIRGEIKRYSAWPTIPQLYLQGEFVGGSDIILEMYQTGELQEMI
EIAAAS*

>jgi|Ochro1393_1_4|696670|e_gw1.6.267.1
MGDVPGLDDKTEKKIQDLITNNKVVLFMKGVNGAPQCGFSDLACRILNTIETPFQSVNVLDDDYIRAGIKVFSSWPTIPQ
LYVDGEFVGGSDIMLEMYESGELAELIEVTNAS*

>jgi|Auran1|28514
MLAGLRVAARAAPRLRAARLLSSGSHSDFETVRAAVPDTPAGVKAMIQEQVDGNTVMLYMKGTPARPQCGFSQQVCRILH
ATGVEFASVNVLEDDALREGIKEFSEWPTIPQLYVGGEFVGGCDIVTESFQSGELDEAFKAAGAK*

>jgi|Auran1|18629
MARTVALIAALAATASAFVAPGAAAPRLSPRRAGDDVEPPPDATTKRIQSMVDDNTVMLFMKGTKIFPQCGFSNMAVQIL
NAIGQDFETYDCLSDENVRSTIKEFSDWPTIPQLYVDGEFVGGSDIMLEMYEAGELQEMIEVAAAS*

>jgi|Vitbras1|7343|Vbra_20848.t1
MQPLSACLTAASIFKLAFVVLSCMAAIADAFSLSLPAHSSSLRQRSPSRALRRHTSALYESLLDSAPSPPEADQLDEATR
ERIAALVRENPVLVFMKGTPEQPQCGFSNTMMQVLQVCGVQRFKAVDVLSDERIRQGIKVYSEWPTIPQLYVNGEFLGGS
DTAVEMYSSGELQELLEVAQAS

>CAE7257650.1 purL [Symbiodinium microadriaticum] bad annotation of the purL protein
MKGTPVFPQCGFSAAVVQILSHLNVKFKGIDVLQDPGIRQGIKDFSNWPTIPQLYVKGEFVGGCDIIREMFQTGELSEFLQTN
GIDIKPAA

GRX6 orthologs

>Cre01.g047800
MQTLSKNAIKASPRPAARMPASVRPFTSSVKPVVSRVSVISHATLAETKAVPVMEKGELSQFPGSAGVYAVYDKNGTLQYIGL
SRKVSASVATHMQELPDLTGSIKYNIVDDASREGLTAAWKSWVEETVAEAGIIPPGNAPGETKWQSRSVARATKPEIRLTAGK
PIQGVTLEELIDRIVKENPIVVFVKGTRQQPQCGFSFRMINMLNTLKADFEVVNVLDEFHNPGLRDAIKNYSQWPTIPQLYIS
GEFVGGADIAEQMVGTGELQTLVRSAAAKDPN*

>jgi|Caulen1|93851|g8441.t2
MTFILKSTLQQLQNFKIETQNAFEDFPEAPAVYAVFDANEALQYIGITRKLRLSIMGHSRDLPDLTHSVK
FETKHGATREELTQSWKEMIEDYVKQTGQVPPGNVTGNDQWKTKRVIRQKPEIRLTPGKGIEDLTISLEK
IIDQIVKTNKVVAFIKGTRLEPKCGFSYQMLTILNRNRVDYEVVDVLDEVYNPGVREAIKNYSQWPTIPQ

LFVNGEFLGGADILTELQDKGEFEQIVKQL*

>jgi|ChlNC64A_1|31055|estExt_Genewise1.C_90387
MQALAAATACMPMARLAAPRQQQRQQRHAPAAASRRQQPCRRSAARRLVMVTAEAGTSGAAAASQLQAFSQTAPQRVLGA
DGKLAEFPTTAGVYAVYDSSGTLHYLGLSRKIAVSLATHVEALPEGLVHSVKVLEVPEASKEELQEAWKAWMQEAVAEAG
AIPPGNASGQTLWQQRRPRAAKPEIRLTPGKGVQDLTCSVEDLVDQVVKTCKVVAFVKGTRTQPQCGFSYKVLTILQESG
ADFEVVNVLDDVYNPGLREAIKNYSAWPTIPQVFVGGEFIGGADIVEEMNGSGELKTALQQAGAVRG*

>jgi|MicpuC3v2|4885|wlab.203974.1
MTTISSDFSVATALHRCPVPRNTRFCRGCRVVHLPSYSWKVRSATGDQLQYQGSSIIPIVSLSAADFSQVIFPEHPGIYA
VYNTTMEIQYIGISRKISSSVKLHKYEMPRYCGFLRFAAIPRATKADLQLLWKQWMQEHLAVSGGLLPPGNVRGNSQWSR
RKHLPPKINILLTNGVNSQVNDSTVFQLCREIVESYNIVAFIKGTRKEPECGFSYRVCAILEELSVDFETVDTLDERHNH
NLRNVLKDFSDWPTIPQVYYRGSLLGGHDIIEDLYKSGQLKHILVPGSTAEAA*

>jgi|Tetstr1|436275|TSEL_025117.t1
MACALTSPGAATFAAMAVAGVARQTPPPPLAAASQGRRLVAIPRASQGETRRWLATFRSGRPSRRPAALG
AAVVCAASTPGMLSINDVEAVPVMENGELTAFPPQAGVYAVYSPEGELQYIGLSRKISASIIGHMQDLPD
LVGAVKVCLLSTDDKQVLMASWKAWVQEIISGTGKVPPGNAPGIKDWTQGGTRTVKPEVRLMKAGTAVED
LTCPVEELIDQVVKEHKVVAFIKGTRTEPACGFSFKMLSLLNGAKVEYEVVNVFDEVHNPGLREAIKSFS
AWPTIPQLYVDGEFVGGADIIVEMSENGELAEVLGAASS*

>jgi|Chlpri1|532|rna-gnl|IITBIO|A3770_01p05320_mRNA_A3770_01p05320
MRGGVGVSVCRGGGRVPNWVGAWVRRGERLVGLRRHGATGGGGGEGGGIPKLEYASSKPLKKAEGLDNWV
PSTPGVYAVFDEREELQYIGLSRRISNSIERHAETELEKMHSVQVLEMPGASKDSLQQMWKSWIESYAEE
TGNIPVGNMKGEGQWVLNRPKVRAKEEIKLTPGKGADDLLVPMDKLIDSLVKTKRVVAFIKGTRTQPECG
FSYQVVTLLNQTHVDYETVNVLDEFYNPGLREAIKVYSDWPTIPQVYVNGEFIGGADIITEMSEKGELVG
ALQEAE*

>jgi|Praco1|15470|PRCOL_00005651-RA
AIPIFADDGAVNELPDIPGVYAVYNAEDELQYVGLSRKIGTSVSSHADEDIRLTPGGNKELTMPIQDLIG
LLVKDYPILLFMKGTRSAPQCGFSYNTADYDVVNCLDEEYNPGLREAIKEFSTWPTIPQLYIEGEFVGGA
DIVQEMALSGELKTMCDAAMASKKK*

>jgi|Chlat1|8811|Chrsp90S08147
MVAMAAAAAAAAFSVSVCSTSAAASSNLATRASSKRQGRPGRGAAFPALPAARVGGGAVGGSVSVKGGSS
RGGLTRRAQAVVDQGGVEEPAASVPRLSEAPSVPAKAAEIPAAPGVYAVYDQAGTLQYVGLSRKIQASAH
VEDLPELTDTIRFATVGASATRADLQGAWQAWVQEHLTETGTVPPGNAGGNTTWTQRRARMSRPEIRILP
GSHVSLTVPVETIIDKMVKEVKVLAFIKGTRTAPQCGFSHRVMQILTESKVDFDTINVLDEEHNPGLREA
IKTYSKWPTIPQLYAHGEFIGGADIVEEMYQSGELKQALSKQG*

>jgi|Mesovir1|4225|Mesvi2744S04823
MACLVRGAVNAVVASNSAVTGVVAKQCNVPLVNSPGQRKLNIPVSQMRSLSLGQCIGRVRASSSRPVNKV
VRATAEPVADAVPALADVPLVSVKEQMAGPSSWKIPPLPGIYAIYNDKKELQYIGLSRKLSSSIQAHAED
VPDQCIYVKAVPVQVATRTGLQDAWKVWVQQHLDAVGDVPPGNVSGNKTWTERKPKATKADIRLTPGNNK
ELTVSMDELLDKLVKDIKVLAFIKGSRTAPQCGFSAKVISILNELKADYETCDVLDEDHNPGLRDAIKLY
SSWPTIPQVYVNGEFLGGADIMVEMYESGELKKLMSKA*

>jgi|Klenit1|10878|rna-KFL_004270110
MAQAAVARISAIPAFHGTTNVRLRTERPPTTPFVPLQAANTSCMGAAPCKIVTVNQVPKTLRVARCGKAFLGGEQELGRQ
QRQRPRATRRPGAVAAELASLAETEAVPIKTADGNLQKFPAAPGVYAIYDSNQDVQFIGLSRKVSVSLEGHAEDLPELVS
SAKVAVVQNVDRSALQAAWKAWVEEYIQQAGKVPPGNVTGNDTWTKRKARGAKPEIRLTPGAHVKLTVPLEEIIDKVVKD
VPVVAFIKGTRTAPQCGFSYRVLTLLNESKVDYEVVNVLDEDHNPGLREAIKQYSQWPTIPQVFVKGEFIGGADILEEMV
QKNELKPLVSK*

>jgi|Chabra1|333276|rna-CBR_g22937
MASSAGAACAAGGPFATVQTRRTSATTGCSLDGDSSSRCFLAVTRASCRTLWQSSVSRCETCPGLATSSGRSGGVTFASA
AAVCRFRSPTAAFSLSFSRRLCSYSNGDDYDNEEDDRMHGRRLYKKKTVFLSDGISTTVGRRVGSGRKKVLGEGGKLSDK
IPPAPGVYAIYDKNGALQYVGLSRKVSFSLANHAARLPDLVATVKIAIVDGPDRDALQAGWKSWVEEHVSAGGAVPPGNM
QGNNTWKESSRRPPKADLRLTPGAHVPLTVPMETLIGAAIKGTPVIAFIKGTRSSPQCGFSHRVLMALNKEGVDYETVNV
LDEEFNPGVREAIKAYSKWPTIPQVFINGELIGGADLLEESVEKGEFKQLLAAIKK*

>jgi|Bigna1|87450|estExt_fgenesh1_pg.C_200153
MKPRSGSRFLILLLAFSVSVVYYQQSSWREARARSDGNSAVLSSPVGTRRYTLPSKTATVTRKLIHQNGI
RARQQKSCNIISPISRQTIVESSSGSQMEFPKLPSVPSVSSEDLSTVPDQTGVYAVLDESQIVRYIGITR
KVASCVELHRRDVSTLTKFIKFHITAPDAPKEELQNLWKAWMQDAIELTGGIPDGNMKGNTMWSLKKEAS
KSSKPELRITSGKGLEDLSVPLDQILKGVVESNKVVAFIKGTRMEPACGFSYQVVSELTKLDVDFEIVNV
LDEMYNPGVRDAIKEMSDWPTLPQLYVKGEFVGGCDIVAEMAENGQLKELVEN*

>jgi|Symmic1|12005|rna10046
MEVPTLSEAPVFVTGVDVLSGEPDVSALPEEMGVYAVYDTGDRLQYIGLSRNIQKNIENHAKAIGLPEAT
DLIASVKCIEMPDESKEVLKQTWEFWLKDHLGDGGEIPVGNLPETAPGADPRWRSRGAQAKPSLNLGGVG
GIASQAEAMEAVKTAVESNPVLLFMKGTPAMPQCGFSARTSGLLREIGVPFETVNVLDEANNPGVREAVK
DFGQWPTIPQLYVSGQLVGGLGS*

BOL1 orthologs

>Cre03.g180700
MIAFASLRSFASRAASMAEHQLGPIAGAIKSKVEAALSPTHFKLINDSHKHAGHYARDGSTASDAGETHFRLEVTSDAFKGLT
LVKRHQLIYGLLSDEFKAGLHALSMTTKTPAEQ

>jgi|Cyapar1|12126|g11718.t1
MRRKITEGLSPTVLNIKDDSHKHAKHAAMQALGGDPKETHFEVEVVSAAFEGKPLDEFKAGLHALALKTK
TPAEAAK*

>jgi|Tetstr1|462346|TSEL_007352.t1
MLRGVFASATSAPGKRPSKLAAAIKRKLKKQLDPVVLRVVDNSAKYGAAAETYYKVRVVSQVFEGKPIMQ
RHRLVYGILDDELSAGVKRVAVEARSPSEELKAPADEEAQAADEA*

>jgi|ChlNC64A_1|15183|gw1.38.73.1
VQEAFQPASLSVENESHKHAGHSGNPGGGPDAETHFKVEIVSAAFEGKKPVQRHRMVYQLLQAEIDAGVH
ALALKTKTPAE

>jgi|Chlpri1|5968|rna-gnl|IITBIO|A3770_10p59680_mRNA_A3770_10p59680
MRLAVMAAREKGYMGPMARRILEKVSNAFDCEVVSLENQSHHHAGHVGNPDGADDAETHFKLKVVSDAFE
GKKLLERHRMVNELLKEELRETGGVHALSLDLKPSKAK*

>jgi|Pico_ML_1|54715|g588.t1
MEERPVRQAIVNKLQEALHPTKLRVEDESHKHAGHAGNPTGRPDAETHFRVEVVSEAFGGRTLVQRHRMV
YKLLDREFQAGSTHWRSKLHA*

>jgi|MicpuC3v2|2592|wlab.211568.1
MLRRAMSTAMRPIYGRIQYKLTDALQPATLVIKDESFQHAGHSGNPSGDPDAETHFNVEVVSEAFSGKLP
VARHRMVYALLDEEIKDGVHALSLKTKTPAEAAK*

>jgi|MicpuC3v2|6012|wlab.170979.1
MAATLLAPRALAPAPAPSPRRARAASTKRAASRPRLAPPPLRAVDPETGEWVSVRDTIEEKIVAALAPTY
VLVRDDSEKHALHKGGQHDGHAGVRSKESHFNVTVVSEQFEGMTPVTRHRTINAILKEEFDAGLHALQLS
TKTPAEYEKSK*

>jgi|Praco1|14778|PRCOL_00000501-RA
MSSATAAATARLPTYRAIVERLGALKPVSLTVTNDSAAHAGHSGNPDGAADAETHFNVEVISEKFDGLRL
IQRHRLIYESLGDLMETIHALSIKAKTPEESGAQ*

>jgi|Chlat1|2063|Chrsp17S02531
MLTSQLVLQRAVAAAAGARLTLRPHPSQHQPARLAASSSSSSRHRAVAMSAAGPMASEMEKKLTEQLRPS
RLEIVNESHMHAGHSGNPGGGPDAETHFRVTVVSDAFEGKSLVARHRLVYGLLDDEMRRAGGVHALALTT
KTPAEMAKAGGS*

>jgi|Mesovir1|8864|Mesvi907S08677
MQTFCTAASAAGLRVRLPGALGISDPRETCLQTPGSVAVGQRRMMHPVRCLSSTKVPGPYQTSINDKIIK
TFKPSLFVIENDSHKHAGLQIVSEAFEGLPTLRRHRMVYEVLAEELDPERVHSVVLKTTKTPKEMEMGPA
PKRKTSPKQ*

>jgi|Mesovir1|4979|Mesvi3629S00501
MGNPEGKADAETHFSLQIVSESFDGVPIVKRHRMVYELLAEELGPGKVHALALKTKTPKELEKGNPA*

>jgi|Klenit1|13179|rna-KFL_006780030
MANNAVGRPLFSLLDSKLRAAFNPVNLTIVNESRMHAGHSGNPSGDPDAETHFRVEMISEAFEKKPLVQR
HRQVYDLLKEELEPGKIHALALKLKTPSEAEKEASKRTL*

>jgi|Mesen1|6426|ME000329S05582
MAAASIRRPMFSTIERKLTEALRPTKLVVTDESHLHAGHTGNPSGSVDAETHFRVEVVSQEFTGKPLVAR
HRLVYNLLDHELKNGIHALTLKLKTPVEQP*

>jgi|Chabra1|352485|rna-CBR_g86666
VEAVSPIFQGMPPVARHRLVYSTLTEELQSGVHALSLVLKTPSELSRKA*

>jgi|Guith1|79024|e_gw1.112.19.1
GALSENFLSCRMFIKNIRLLLRSSVKYPQHIYQSAALAKRSMSTEGKGPIYSSIWNVIDKQLQPTHIELV
DDSAKHAGHEAMKGSTAKETHFRLKVVSSKFEGLSLVKRHQMVYALLNEQFQQGLHALNIVAKTPQEVEK
R*

>jgi|Guith1|97357|estExt_Genewise1.C_990007
MLDDTGFQASDSLFQSKMIITDNSHMHAGHAGVQGRSGETHFAVEVVSEAFVGLRPVQRHQLVYRLLSKQ
MEGGMENGGIHALQIQARTPEESQS*

>jgi|Emihu1|61409|e_gw1.3.105.1
GPVAASITAKLEAAFKPDVLDVVNESASHNVPRGSESHFKVVLVAHAFASVPLLERHRLVNEALADELKG
PVHALSIVAKTPAQWAKASGAVPPSPPCLGGSKA*

>jgi|Bigna1|91348|estExt_fgenesh1_pg.C_980005
MTRRLLPLVLLALVAAALLATMNAGPTLSFVGGSIGIRNNSVGMRVGGPGMAAMKSCRRNTHAGATVEGR
PIYSAMQNKLTEKLVPKKLVITDDSNKHSEHEAMVGKAAESGETHFTVEVVSDAFEGVGLVKRHRMIYEI
LKEELDAGVHALSLKTKTPKEAGL*

>jgi|Phatr2|11230|e_gw1.5.361.1
RPMYDGIINALQKLQPGELRLVDNSQQHAGHAGAKGLDGDETHFDLYITSDAFDGLSLVKRHKLVYMMLG
EIMPKIHALQIKALTLAEADAKK*

>jgi|Pelago2097_1|528065|fgenesh1_kg.88_#_10_#_Locus3199v1rpkm57.49
MFVVAARGLRRASRAPVSRLLSTQPAEVVMKAKLVAALAPTRVDVVDTSGGCGAMYNIEVESAQFKGLST
LKQHKIVYALLHDDIKAMHGLSLKTSASP*

>jgi|Vitbras1|2634|Vbra_11822.t1
MATKGPIYEAIERKLTEKLTPTKLEIVDESHLHAGHAAMKGLDRKQETHFKVMVESAAFANVPMIKRHRL
IYEILDDEIKAGVHALSISAKPPDPGT

>jgi|Vitbras1|18143|Vbra_17762.t1
MGPVYDAIERKLTEELSPTSLEIVDESAGHADHLGMQEEPHKFAGGETHFRIDVTSEMFEGLSRVKRHQL
VYKILDEEIKNAIHAISINAQAPE

BOL4 orthologs

>Cre09.g394701
MASLFARPALALPRIGHQEACCSSSITFARFAARTARRVAPATPVPSAEVSRRSQLLVSRGIPVSGPGATSSSSGGVDGQVTS
VLMESMKAKICEALETDDCSISDVYGDGRHVSIDVVSKLFEGKNSMQRQRMVYKAIWLELQETVHAVDAMTTKTPEEAK*

>jgi|Galsul1|3163|XM_005708841.1
MSSSNDTVVERVEQKLREALSPKFLQVTPAFDDPNGSHVSIRVVSEKFENLTSLKRHQLVYKAIWDELQG
PVHAVDSIDAKTPKEVEQP*

>jgi|Cyapar1|13349|g12896.t1
MSAFALNLAAVAFPRGHISVPRIRPAAAVAVRSVGINEARSFFGQTIFAAAAPRRTFVAQPPRAQADSAP
AHPTVAQVSKKLTDALAPKKLQVIDNAGDASHIVIDIVSSQFEGKNVVARHRMIYKAIWDEMQSNQVHAV
DSIIAKTPAEAGL*

>jgi|Caulen1|96281|g2521.t1
MSSELIDSMRSKIQNALNADSVEVVDVEGDGQHVTISVISSTFEGKSSVDRQRMVYKAIWYEMQNVVHAV
DNMITRTPTEASSSQ*

>jgi|ChlNC64A_1|145612|IGS.gm_9_00419
MAQLFSGRIITAMASASRAAAGSRAWVARPLAAAPLVAARPSQLLIARASSMDNNIGSALMDQMRGKIQA
ALSAELVQVEDMQGDGRHVEIVVVSKEFEGKSAVNRQRMVYKAIWEELQETVHAVDAMVTKTPEEMSG*

>jgi|Tetstr1|458505|TSEL_044911.t1
MARVVLSAAGCRPAVPSRAAQAPRRTAALLLPLLSRPGTHSHYAFRQANAVALAIPVSGPGQQPDAGGDS
GGADGQMTAELMATMTSKIGEALETESVNIVDVYGDGRHVSIDVVSELFEGKSSMQRQRLVYKAIWFELQ
DAVHAVDSMTTKTPTEVA*

>jgi|Tetstr1|435952|TSEL_024833.t1
MARVVLSAAGCRPAVPSRAAQAPRRTAALLPPLLSRPGTHSHYAFRQANAVALAIPVSGPGQQPDAGGDS
GGADGQMTAELMATMTSKIGEALETESVNIVDVYGDGRHVSIDVVSELFEGKSSMQRQRLVYKAIWFELQ
DAVHAVDSMTTKTPTEVA*

>jgi|Chlpri1|6774|rna-gnl|IITBIO|A3770_12p67740_mRNA_A3770_12p67740
MLASFATKRGATGGGPGRIRGEHKGVLGGDLGAGRGSRKRSSPRGRSPTTTTGLRQRGWLATSAGDDRSG
QITEDLMNSMTEKISAGLNPGNDEGERVSVTDASGDGSHVYIEVVSRVFEGKTSVQRQRLVYKAIWEELQ
EAVHAVDGMSCKTPEEV*

>jgi|Pico_ML_1|53206|g400.t1
MRTKIESELEAQVVRVADVSGDGRHVNIDVVSNAFEGKSSVARQRMVYKAIWQELQDVVHAVDAMTCKTP
GEIKN*

>jgi|Pico_ML_1|52689|g3362.t1
MRTKIESELEAQVVRVADVSGDGRHVNIDVVSNAFEGKSSVARQRMVYKAIWQELQDVVHAVDAMTCKTP
GEIKN*

>jgi|MicpuC3v2|9446|wlab.223966.1
MSHHVRRGSGALLAAARQLARARAVRASASQHRHNFVTWQRSETPGGSRAETRGFASSPASRAGEFDRPE
SGLIAGMKSKIQTSLEATFVDVVDMSGDGRHVTIKVVSKAFEGKNPINRQRMVYKAIWEEMQTDKVHAVQ
GIVTQTPEEAQ*

>jgi|Praco1|21015|PRCOL_00004611-RA
MMWFRLRMRARFRGPNARLISAAAAAGSGENMSDNNITGAVIEGMKVKITEALEAQSVEIQDAYGDNQHV
SIDVVSTMFEGKNSVQRQRLVYKAIWQEMQETVHAVDAMTCKTPEEAGK*

>jgi|Chlat1|6644|Chrsp49S06122
MSVSAGLRVLGGARRVLSRPVASAVSAATNAGTGPSTSAAAAGSQLGFVGLRHRSGISAATPSQQQRSRR
SLVTRAEAGGDASAPLDSPTIANMREKISSELEATHVSVQDAYGDQRHVSIDVVSPLFEGKTRVARQRMV
YQVIWEELQNTVHAVDSLTTKTPDEAAASSPPKRL*

>jgi|Mesovir1|4462|Mesvi301S05029
MLSTAAMVSPVTVAVLARPAASSASYAPAMANPCRAMPTKALRASGVRGLSLKQSLSAKPLYMHRRNVTV
CSIPVSGPIDAPLIQSMRQKIQEALEPKELVVKDVSGDQRHVSIYVVADAFEGETSVNRQRKVYKAIWEE
LQQAVHAVDNLVTRTPEEAASKPAPNFDA*

>jgi|Klenit1|3928|rna-KFL_000810225
MAASSSDVGDGGQLTSTMINDMKEKIGQELEAVSVVVQDISGDGRHVSIDVVSPVFEGKSSMNRQRMVYK
AIWQELQGAVHAVDAMTTKTPTEAGVGQQPRQ*

>jgi|Mesen1|8083|ME000434S07328
MATLCYGAATSTVLALGKLPGCLGRDKAQSLSCIKGVANNKSLVSFQPHRFQQRSRGQLLQVRAIEVSGP
GAVNSPLMQDMHEKIKQQLEAESVEIKDAYGDGRHVSITVVSPLFEGKRSLDRQRMVYKAIWEELQGTVH
AVDTLVTKAPSEVV*

>jgi|Guith1|100004|au.3_g1106
MSTDASAPEEDTVMSTIEKKITSELKPVKLEVIPAYGDPNGSHVTINVVSQAFEGKRAVQRQQMVYKVIW
EEMQGAIHAVDQMKCQTPAEAGM*

>jgi|Emihu1|452727|estExtDG_Genemark1.C_670140
MASALPFTVRAARTSRALPSLSPVAAVGWRSLGVRMQSGINDPTVVDRCTAALEAALEPTSVKVMGAYDD
PNGSHITIECVSPKFEGKRSLARQQMVFKAIWAEMDGQGGTVHAVDTMVLKSPSEVKDE*

>jgi|Phatr2|33016|fgenesh1_pg.C_chr_2000557
MWTTASRAFRPVVRVAWARNTATRQFSTANGPDTTIVQTCQQKIQDALSATDVKVTGAYDDPNGSHISIE
VVAEAFAGKRPVQRQQMVYKAIWEELKGPVHAVDAMICKTPDEV*

>jgi|Nanoce1779_2|579356|fgenesh1_kg.4_#_1424_#_TRINITY_DN5163_c0_g4_i1
MTMPCRRSPSSVLLLFLACFMALTCQGFLVASPSSSLISFRRTARTSLTAAADGAVAGEVGAVESCRIKI
QAALSPVELVVKSSNDDPNGSHIALRVISAAFEGKNRVQRQQVVYKAIWEELSGPIHAVDELVCRTPAEE
GM*

>jgi|Ochro2298_1|397071|gm1.8186_g
MAARSLSLRLLRQAARPHALASASRWATSAPSLSAGLRPTPFLSSTRLFSSEGEDTVVSTCTKKIADLLS
PVHLKVTSSNDDPNGSHIQVEVVSAQFEGKNTLARQRLVYKAIWEEMNGPVHAVDSIVAKTPKEVGME*

NFU1 orthologs

>jgi|Chlre5_6|8759|Cre17.g710800.t1.2
MLLRNKTGPAQVGRQAAHATIQRPVLVPRGLAVRALATAAPPAPIDDKTVPEGHKGLHNYLYSGDADKEHEQDAYKVRKD
EDEGGVVMPVPAYLDTRDGEKPLGVYCVYDSAGAAAYVGYSRNMVLAIKGHLARVGPARCAAVRATVYGNKAMASRANLE
RGVASWVEEQLGGVVPPGNADPQQRALWEGVAATSAASGDESGPALDVSHMSPAERAAYEEKRTKMRKAMGEKMVEGDKP

GGQPNEEPPEDLATRRAKLMKAMDRGDWSAVIDQQTQEAVAAQQQDQQQEGSSSSSSSGGGAAGTDGVVSPFIREATMDE
GDLIKERQQAGSSGRSGRELSVEAVTAALDEVRPYLEADGGDVEVVEVENGVVYLRLQGACSSCPSQSATMKGGIERAIR
NAFGDQVRDILQLDAKEPAATAEAVDRQLDMLRGAISNLGGSVEVVSVEAGVATLKYKGPKPVGKGIQNMVRDKFKKQLR
EVRLVEA*

>jgi|Galsul1|656|XM_005707587.1
MLLFLHSCGPIQVGCFYRTRNISHTKKGKLLVVPQCWKASVNKDFKDAPFSSNYIIDDNSIPEGHRSLHETLYSRGEEEH
KTPSKQQVKFRSDGSNWLPVDLFLNGMEDEKVGAVYAVYDANRDIRFIGISRDVCFALKAHREALSDRDVAWLRLKTWDF
PRRVEMEQCQKDWIAELGFTPPGNITTNEESKLWAKSIKEATQLVASDVEPERKSTYEEKKFKLRKAMADPSLIDEESRE
IFERDPTIHAISEGDWSKVIEQQSNKVLQDAVTSPFESVEVVANNNRVDERSNSNTAESGSFLTVENVNLVLDEVRPYLE
SDGGNVKVLSVDTNRNVVLLLQGACGTCPSSTTTMKLGIERILRQRFPNIGEIVAQSEVASVTTIPLEERCESLLEEIRP
AIIGLGGSISVSRVENNQVFLLYQGPDKIKYGIELALKEKLGSSVSIVFE*

>jgi|Caulen1|88997|g4355.t1
MLITNNAVLRPNGVLVPCRIQSCCKLRRSRYRLRTQVSSGGGPPSEALSSQNGVISDSNVPEGHRGLHGALYGEGGAELH
NSSSYEFRDGEDDGEAVMTVSSYLATREGERPLGVYAVYDKSHNIQYIGYSRNIVLALKRHVEKLGEDLCYYLRVMVFMN
RAMSTRENLEREALDWIEREGTLPPGNGAEKDLWDLGSDTLGVSNMSDKERAVYEEKKNKLRKAMGENLHDPVPGESLES
KERRLRLIKAVEGDNWSAVVNEQTNETIEGKPQEQIVSPFAQAPIHRSIGQETDHPDEEMTTESVDTALDEVRPYLIADG
GNVEVVNVEDGNVFLRLQGACGTCPSSTATMKMGIERVLKTSFGDQLIEVIQVDSQDTSASIQSVNDQLDVLRPAIQGYG
GSVEVLKVEDGVCELKYTGPAPIGMGIQAAIRDKFPDIHDVKLIS*

>jgi|ChlNC64A_1|58507|estExt_fgenesh3_pg.C_170017
MSARSLGPSPPGASLGSHRRAAALRPGPGRPQPSTGASGGRRRAPLAHAGGAPTSPAGPVTDAAVPEGHQGLHGFLYGEG
GAEAHDGGIGYTFREGEDDGCSLLATAGYVAAREGEKPVGVYALYDDKRNLQYVGYARNVVLAVKAHLARVGEERCAWVR
VMVFANKAMQSRAALQREADNWLAEAGTLPPGNGAEAELWAQGVGASGASVDVSLMSADELAQYEERKLKLRKAMGENLH
DDVAGEAEDSRTRRLKLIAAVEGDDWSEVIQEQSAEAVVSGEVGSARQQAQQAGGEALAPPAAAPPIVTPFARASVHRAV
GNSSAAAGAGGEAGRPLEMTVEAVDAALDDVRPYLIADGGNVDVVAVEDGRVFLQLQGACGTCPSSTATMKMGIERSLKA
AFGKQLVEVLQVGGQEDNRATAEGVDMHLNMLRGAVGAYGGSVEVVGVEQGVCTLHYKGPEAIGYGLRAAVRDKFPDLVE
VLMMDPDTGEPIKFQA*

>jgi|Chlpri1|5480|rna-gnl|IITBIO|A3770_09p54800_mRNA_A3770_09p54800
MVVAMTMTMMSGPPGRCLRLRAVVSDDNVPEGHANLHNELYGDKSEGGKAHAAVVVNDFDEVLEGELDGTTLFVTSEWLE
KRDGTKPCGLYAIYDEGESLKFVGYSRNIVLAIRGYTEVDAEGKPDEFKHCRVKLISGKRLTSRAYMQQQRDSWVEDLTG
AKGGEGEGVALSSGQGPQPAVKRSEAEEATHEEQKLKMRKAMGDSLDAPASGQETEDKKQRRLNTIKAVEGDDWSEVIDR
QTRETRGQSPATEEGSAPADRVSPFAKPGSAMPRGGEAGGRSVMDADTVNEVLDEVRPYLIADGGNVSVVAVEDGVVKLA
LEGACGSCASATATMSMGIERALRNHFGSLLKQVVRVDPGAGGGSDEGVAPSAVDAHLDMLRSAIENYGGSVRVKSVDGA
VCVVQYKGPPPLSKGIEAAIKDSFPALRQINFVSFD*

>jgi|MicpuC3v2|101|wlab.202714.1
MPSHAVAVAAARALHRAAPPSASTARRVAAPSGGATPRRSRTLPPVAPPRASSSSSSSSTAAAVDAETVHAIRSGPPDDG
TAIVPLRAWLEARDGDPAVASVYAIYGADDVMRYVGYAKDASRAVRSHLERQGDDAASKVRVAAFANKATATRANLRAEQ
DRWIGEWVVSRGGAETEIPVGNQAAGAARWTLTPAESWENEEKPAPAEAKANEAGTPSVGADGEVISPYAAEAAGADAEE
EEALDPNRELLPLTVENVDKALDEVRPYLIADGGNVAVVGIEDGVVAVRMSGACGSCSSSTATLKGGIEKTLRRVFGGEN
VKEVVNLDSDEPGSALTLSKEAVEAHLEKLAGAIHNYGGSVKLLEVIESERALVLEFSGPVALAQSIASSIKGKFPLVAE
CKIKQV*

>jgi|Praco1|15791|PRCOL_00005187-RA
MPCARAEGAARAARAAAAPRRPLLGRRRPSSSVAAASGAGGVPTPAPTTQGVATDDNVPEAHRGLHDFLYGEAGGDVHGG
EARAAAAVAGEGCGPADPALSGAAPEDDGRSCVRADAWLAARDGAKLAGVFAVSDARGDTQFVSYARDVVASVRALRGRL
GPRHVAAVRARIFAASMARREELNALADAWLREELPHVPPGNAQGEDASRWRGHDSATALSGAALASYEDNRLRLRRAMG
ENLADAEAGESASLRERRLAMLSAVHGDDWSEVIGEQTAEAVGLEDPGGQREQAGGSDTGGAPIASPFSAGGAGEATAAA
VGTDGDDGHGAATGGAPDPQSQPQQPALSMSTADECLDAVRPYLVADGGNVEVVGVAGGVVLLRLTGACGTCPSSAATMS
MGLEKALRATFGEQVQEVVQVDGGGAPVDTSPGALSGVTIASVEAHLEVLRPTVEKYGGSCRVVSVSAGGDSVAIEFEGP
APMAMGIEAALKDKFPTLKHVDVDIGG*

>jgi|Chlat1|7988|Chrsp7S07745
MAAATVYLGVVGVVGGSETVVSHPRQQQAACSSAAATGASSPQLGLVLRGGRRRRHGSHAGERLSTRAGAAAPAGSTTTE
RSKDGDASSNGLSRRRPTQEQDTVHAPRAAVDFADETYSQQVPEGHKELHGFLYGDGGAEVHGDASSTSVYVPRDGEDDG
AVVVELEQWASVRELAKPAGVYAIYDEADTLQYVGYSRSMAVAVRAHRARAGPHKAAKVRARVVRDPAMITRTRLEAVRD
EWMTDEFGHVPLGNGSEKELWEGVGSLAAVMTDAERAAYEEKKLKMRKAMGENLYDDVEGEAEDSKQRRLNLLRAVEGDD
WSGVIDGQTKETVAIPSRTPIPSPAPVEQIVSPFARANAPSVGSTALPMLELTPANVDKVLDEVRPYLIADGGNVEVAGV
HDGVVALRLQGACGTCPSSTATMKMGLEKSLRAHFGDILKEVVQVDKTDITATVQKVDAHLDMLRPAITNYGGSVEVVSV
DAAAGTCEVRYSGPPPIGMGVQAAIKDKFPDIRQVTLLQ*

>jgi|Mesovir1|4293|Mesvi284S04891
MAAISATAVNAAVCARIDSISAPARSRNVHHGSWRASSIRRLGESARLGSRNTGSMPLVVAASTAGEAMGITENEKARLN
SLGGVMTDETVPEGHQGLHGFLYGEGGAEDAHGNGKTYAVRPGEDDGAAILPLEAWANARTGERPAGVYAVYATDGTLQY
VGYSRSVVASLRQHLDALGGTMCASVRVKVMTDASLITRARLEAEVEQWVQRLGNGAVPPGNGAQAHLWGRKQATPSAMS

PEELREYEEKKLKMRKAMGENLYDDVQGETPDAKTRRMRLLQAVEGDDWSSVIDEQTQATIAGGAAAPAAAGPASEQIVS
PFARPGAPDMNGPAGAPSAAPRPMNKDTVDLALEEVRPFLIADGGNVEVVGVEDGIIALRLNGACGTCPSSTSTMKMGIE
RSLRAAFGDQLKEVVQVDKAETGATVESVNAHLDILRPAIVAYGGKVEVVSVDAIRGICEIRFEGPPPIGMGIRAAVKDK
FPELRKIELL*

>jgi|Klenit1|8719|rna-KFL_002800020
MKAVQQLSTQAFQACFSSSVSGHAAQSPANSRNAPRGVPRRQPRWQRAPVCRAAGEGVTTLPKGPSAGGSPSSAVRPDLT
ATLGAVVSDAAVPEGHKGLHGFLYGDAGADVHDDGAAFQVRGEEDDGSALVGLQQWLAGREAERPPGVYAVYDQGGDLQY
IGYSRNLVLTLKQHRARVGPERSAGVRVKVYANAALVSRARLEEERQAWLAQQPVPPGNGPERDLWEGTGPATAVMTEAE
RGAYEEKKLKMRKAMGENLYDAVEGEDADARTRRLRLLAAVEGDDWSGVIDGQTQETLAPPQAQRAAAAAAAIEAAKAAV
AAKPQIVSPFARPNAPALGAPAAEKPATVMSVESVDRVLDGVRPYLIADGGNVEVVGVEDGIVALRLQGACGTCPSSTAT
MQMGIEAALKAHFGAALQQVVQVDKQDIGATLEAVNSHLDILRPAITNYGGSVEVLAVDTLKGSADVRFKGPAPIGMGIQ
AAIKDKFPDIKKVNLVE*

>jgi|Mesen1|3635|ME000020S03162
MRAEVAQQSTVTPSTSDEAAQRQRLTNLGGVVTDETVPEGHKGLHGFLYGDGGADVHDSPNGGYTLQEGEDDGTQVVPFE
QYVGARDAQRFAGVYAIYDQQQSVQYIGFSRSVVLSLKGPNGAGTAAMSEEERTQYEEKKLKMRKAMGENLYDDVDGETL
DSKERRMRLLQATEGDDWSSVIDGQTKDTLDPRLATPGTDAAAAVGALEAATAEQEEAAAPPEAQIVSPFMRPGALPLGE
AAAAAAPVEMSVSSVDKALDQVRPYLIADGGNVEVVGVENGVVFLRLQGACGTCPSSTATMKMGIERSLQATFGEQLKEV
MQVDKIDISASITAVNAHLDMLRPAIQNFGGSVEVISVDSSRGTCEVKYKGPPPIGMGIQAAIKDKFPDIRKVVLLDA*

>jgi|Chabra1|324028|rna-CBR_g4584
MEACMGSAFLQAQSQAAGVMVASSSTLVGVAGLMATAQERLQCQTLPGRTVLSSMARSPTLAPSRRVQVGLFGSPQKGSL
RSAVTVIRCSKPEAQPSVQTAISPDMAERLSSLGGIVTDAAVPEGHVGLHGFLYGSGGAEVHDGGAKTYVGRPGEDDGSI
MLAFEDWADPREGQRPAGVFAIYDGDESLQYVGYSRNIVVSLRQFRMRMGVEKTGMVRVKVLTDKAMLTRAKLEEERDRW
MGELTGGNLLPPGNAEEKGVWEGTDGSGNGSALIAMTEEERAAYEETKLKLRKAMGDNLYDDVEGEDEDSRQRRLNLLRA
VEGDDWSGVIDRQTRETQAGPAPSAPTSTAASEKPAEAGQIVSPFARPGAPNIGSAGSAVAPQVPLELTAENVDKVLDEV
RPYLIADGGNVEVVTVENGVVALRLQGACGTCPSSTATMKMGIERSLESKFGERLKQVIQVDKQDIGATVESVNSHLDIL
RPAITNYGGEVEVMAVNTQKRSCDIRFKGPAPIGMGIQAAIKDKFPDILVVNLLEG*

>jgi|Ectsil1|31965|rna4552
MGRYSASLLVLAVLSLEASSFVFPQLASTAAAVSRSRAPHASRSSSIPHRTAAGRGVVLQSTTDETTETAEEVRLEDRDV
PQAHRGLHDFLYSNEEDHGATASDGSVELFDGTQLYEVSKWLKDNAESKHAAVYAVVGKDEKVNFVGVGRNVALSLAAHL
AAEGESMVHMLKVRSFKFPKRDKMEALKAQWIEECGSTPVGNIKGSEWATGSKDAQNAMSPAQKEAYEENKLKMRKAIAD
PGLVEETEQMSSEDIERRKRLESAVEDDDWSSVIDGQTKLTEEPKVEVDAPTATIISPFSSDGMPVGVGPDGMAAPPLPP
GIAAPVPDAPPTPPASECNDLEFTLENVDKVLDEVRPYLIADGGNVRVMGVDIDRRVVKLALQGACGSCPSSTTTMKMGI
ERVLNENFLNMGGVEQVDEASGNAMAEATTAVVEAILEPLRPAMVAMRAKVEVLSVLDGHVKLTYSGHRKVAYGIQMALL
DNPLIQSIDFEYVD*

>jgi|Nanoce1779_2|604355|fgenesh1_kg.29_#_125_#_TRINITY_DN4477_c0_g2_i1 (bad annotation)
MMRRCPTTTAAAMVLLLGGISFFQQQQELVQAFLVLTPKTAPPFLLYHQHGNPPCRLESSASGVSAVTAVAADDNVPEGH
KGLHEFLYGDNNDHGATSAAAAEAEAFTRTQTPLPVAAFLDRFKDVRLAAVYGVYDAKQTLRYVGVTRNVVVSLTGHLQV
HGAEVVSSVALQSFKFPKREEMAAAQETWLAELSAAHIVPDGNREGGKGMVVGAKSIRKRLEQGSGSTAEERAAYEDTKT
KLRTAMADPTLADELDDLDEEEEYGEEAEDERMFRRAQLKKAVEQSDWSDPSNDQEASTTGGVAARAAPAAPAAVIVSPF
NGGDSSSPSVSDGLLALTPENVDIVLEEVRPLPHFRWDAADRSSSTEEEIGGKLEEILPAIRGLGASATIEDVSEEGVVS
LCYKGPEKVKLGVVYALKGQPGVTDVK

>jgi|Vitbras1|7107|Vbra_20791.t1
MECPNWIFALVLVFSLWRIRVDARRISAHTRGAPAAFLHLMPQLPTFGTDRRTGRQRRGHAEAAAAAASSQPSPVDELIA
EQPSLSPIQTDANVPDGHRGLHSFLYDDGKEDDAHAAKPAGSRGRVRFDGVNMYGVDRWLGMHREERVCGVYSVLDEDRS
VQYVGVSRNVAVALESHRRQTPPGTVAFVRIKSWDYPRREEMDALRDEWIRESGRVPPGNEDASLGFAETIKDASTDGMK
DDERQQYHDNKLKLRRAMADNTLIDETPNIIEEEIMNREALLHALKDDDWSAVIDGQTNATLNPQLINAQLQEALKIGRD
DTSTDTQGEANVVSPFAPGRQGDDAVAARTAEPAESLPLTVDNVDRVLDDVRPYLQADGGNVMVVAVDPTHKTISLALQG
ACGNCPSSQMTLKMGIERLLKETWPDLNDVVPVSDPSSPHAAPAPLPFGVELIERHLEEVRWGLNGMGASCDVIEAKDGR
AVLRYAGPDPGRITFGIELMLKDAVPNNQLQVVEFVTDEPQTDDRQQATAA

NFU2 orthologs

>jgi|Galsul1|6376|XM_005702978.1
MLGFLFGFCSYKPHHVAFTCFKNRQRFSYQPTVHKIRRLQISADSALPLTKENVEQVLEELRPYLIADGGNVSLTGIDGA
TVRLTLEGACGSCPSSTVTLRMGIETRLKEKIPEIEAVVQEETMGPELNEQNIDSVLDEVRPFLKIAGGKIDLVGIYGTD
SPSPSVSLKMSGGGAAVDSVRLEIIHRLKRNFPKLVNVHYVKA*

>jgi|Cyapar1|6920|g6698.t1
MEASFVVHAPAVRLRSQAASSSRNSAACCRHAPANDISARRAVGPRRLAVEESRSFFAGRQFLRAAVAVRPRALNTTVRM
AAETLPLTPENVEIVLDEVRPYLIADGGNVQLVEIDGRVVRLQLQGACGSCPSSTMTMKMGIERKMKERIPEVAEVVNVQ
EAGMELSTENVDKVLEGIRPFLSSEGSTIEVDKIEGSAVTLRMTGSGSLVMSLRMEIMRRVQEKIPSVTRVAFSDPDAK*

>jgi|Chlre5_6|19236|Cre18.g748447.t1.1
MASANILRAQAGASVSPRCATLAASLLPARATSSISRRQQLASVSSTPARVWGAAPSTRRGARVVVRAVEQDEMMELTPE
NVEKVLDEVRPYLMADGGNVEFMEIDGLVVKLKLQGACGSCPSSTTTMTMGIKRRLMERIPEILDVEQVTEESLGLELNS
DNVETVLNEIRPYLVGTGGGGLELVAIDGVIVKVKISGPAANVMTVRVAVTQKLREKIPGIAAVQLV*

>jgi|Caulen1|93689|g8283.t1
MLAVCGSVYCLQIIREPLRNSTPKTNAFCLSYGPSSAVLRAPGPVTPLRTTHRYKHSALASYRSPGVFASSVGETLELTA
ENVESVLDEIRPYLMADGGNVELVEIDGLTVLLRLQGACGSCPSSLTTMTMGIKRRLMEKIPEILDVEQIIDEQEGLELN
PYNVDAVLDEIRPYLVGTGGGELMFHRLEGPIVTIRITGPAANVMTVRVAVTQKLRERIPAIAAVKLV*

>jgi|ChlNC64A_1|144603|IGS.gm_7_00545
MTVGSSGAVCQAAATDTLELTEENVETVLDEVRPYLMADGGNVEFVEIDGPVVYLRLAGACGSCPSSLTTMTMGIKRRLM
ERIPLGGWSRAWPYLVGTGGGGLELVELDGPIAKVRITGPAANVMTVRVAVTQKLRERIPAIAAVQLVN*

>jgi|Tetstr1|440027|TSEL_028388.t1
MAAMAATRTVLCGCSDGRPTASRTAQRVGCAVPPRSAGLLARAPAPTPALRSHRSSRGGVAVAAARSFVLAAADTDTLEL
TEENVEIVLDEVRPYLMADGGDVELVEIDGLVVKLRLKGACGSCPSSLTTMKMGIQRRMQERIPEVMDVEQVMDEDGGGL
ELTEENVDAVLEEIRPYLVGTGGGELMCKGVDGVIVKVAITGPAAKVMTVRVAVTQKLREKIPGIAAVQLV*

>jgi|Chlpri1|6335|rna-gnl|IITBIO|A3770_11p63350_mRNA_A3770_11p63350
MVATMTATTVARLPAKAARRALREESGVARGEHSSSARSCGLGRLSPWPEATPSVSSGGKDRGQSQVLAALGGGRWVCGM
ASAEESYTLELTEENVETVLDEVRPYLMADGGNVELLEIDGLTVRVKLQGACGSCPSSTTTMKMGIQRKLMEKIPEILEV
EQVMDEIEGLDLTEENVESVLDEIRPYLVGAGGGAIELEEIDGPIVKVHLSGEAASVMTVRVSVTQKLREKIPLIAAVQL
T*

>jgi|Pico_ML_1|50958|g2072.t1
MAEPQVVVDDGARTFAAFYDVFAPLAARIAAEEDEELLHAFLSSKRVQTAPGARQERCSRARTDLELTEENVELVLDELR
PYLMSDGGNVELVEVDGLTVKLRLQGACGSCPSSTTTMTMGIKRRLMEKIPEILEVEQVEDANAGLELTPENVDSVLGEI
RPYLSGTGGGELELEEIDGPLVKVRLSGPAADVLTVRVAVTQKLRERIPMIAAVQLV*

>jgi|MicpuC3v2|4144|wlab.213321.1
MATAFARVATATAAHRGSTSAALAASAAPGPRFAPARVPASGPRPRFAPRHATDSPSSTSSTTEKLELTAENVEKVLDEV
RPYLIADGGDVELVEIDGLVVRLKLNGACGSCPSSTVTMRMGIERRLLEVIPEIMEVEQVTEEEVGLELNEANVEATLDE
IRPYLKGTGGGELELVDIEEPIVKIKLSGPAASVMTVRVAVTQKLREKMPSIAAVQLL*

>jgi|Praco1|19513|PRCOL_00002783-RA
MADGGNVELVEVDGPVVRVRLNGACGSCPSSTVTMQQGIQRRLMEKIPEISEVEQLIDEQEAGLPLTEENIEATLDEIRP
YLSGTGGGELALDSIDGPIVKIELSGPAADVMTVRVAVTQKLREKIPSIAAVQLV*

>jgi|Chlat1|7859|Chrsp66S00579
MGFAMAVSVGTVVLGGSGCGVSSLRGRDGAGAAASSASTSRQSAEAATPRQAWLRRGGLSGLRHGSSTSRQRRFPTSAGG
RRASTSMTAASFDTLALTEENIESVLDEIRPYLMADGGNVELFEIDGLVVRLKLQGACGSCPSSTMTMKMGIERRLMEKI
PEITEVEQVLDEETGLELNAENVESVLSEIRPYLTGTGGGELQLVKIDGPVVKIRLSGPAAGVMTVRVAVTQKLREKIPM
IAAVQLL*

>jgi|Mesovir1|8422|Mesvi827S08310
MLGIGFLCTFTARVRRVAVASSAGLEGLALTAENVESTLDEVRPYLMADGGNVELVEIDGPIVRLRLQGACGSCPSSVTT
MKMGIERRLIEKIPEILEVEQVMEVVKGMELNEANIDIVLDEIRPYLVGTGGGDLQLVKIDGPVVKIRIEGPAADVMTVR
VAVTQKLREKIPMIAAVQLL*

>jgi|Klenit1|2738|rna-KFL_000500450
MDKAQGFKISRHPTPSIGARMSSFAGTRMRSPGVASVRKGIRGAAQQGRAQVVMDVEGVPLTEENVEKVMDEIRPYLMAD
GGNVELVEIDGLTVRLRLQGACGSCPSSTMTMKMGIERRLMEKIPEIQAVEQVLDEETGLPLTDENVENVLSEIRPYLVG
TGGGELTLVKIDGPVVKVQIDGPAANVMTVRVAVTQKLREKIPGIAAVQLL*

>jgi|Mesen1|7241|ME000373S06309
MAGVAMIGSLAGLTSSQVAHCAGTYGSGATCSSRDATAASTALPSHRQLRVLRADDALSGLRVARGWIPLNPLRNMLRQK
GSPEQHRVVAVATQQGEVYTEGLELTEDNVEAVLDEVRPYLMADGGNVELYEIDGLVVKLKMQGACGSCPSSMVTMKMGI
ERRLMEKIPEIVGVEQIMDQETGLPLTEENVEKVLSEIRPYLSGTGGGNLTLVKIDGPVVKIAIDGAAAGVMTVRVAVTQ
KLREKIPLIAAVQLL*

>jgi|Chabra1|322077|rna-CBR_g3282
MGISSAAWAAGTLPNVGASQAAGCNSPVGGTTASSSSASVETRCATCHALTARRGGLTQVVAARSNGASACRRLGAGYGE
NDLPLGCSGRHLFPSSSSSSSSFSPSFRRTTNPSLDGLDKIERDNASLSPLSSAARRRPRTSPVLAMVSATAGTEGLELT
EQNVEKVLDEIRPYLMADGGNVELYEIDGLSVKLKLKGACGSCPSSMMTMKMGIERRLIERIPEITDVEQVMDEETGLPL
TNENVEKVLSEIRPYLVGTGGGELTLEKIDGPVVKVRIDGPAAGVMTVRVAVTQKLREKIPMIAAVQLL*

>jgi|Guith1|106027|au.22_g7129
MEWVWRPLVLRSAKRSLLALKDTDSIVSPFDPSVDPLLPKETEESPTDLDGPLPLTWENVEKVLDTMRPYLMSDGGNVKI
ADIDGGIVRLKLEGACGTCPSSTMTMKMGLERGLREKIPEIVDVVQDLGDGGPELSPDSVEKVLDTVRPFLKVAGGSIEL
FDLRGVGGMQPVIILKMTGTSAALRSVKNEIVQRLQRNFMLAGLRIEWTD*

>jgi|Emihu1|452262|estExtDG_Genemark1.C_590098
MASCGGRARCLLLLSSWLHSAGSLRVPAVSAVPRVQHRAGAPRCSDPVTSPFEASASNGGETLTGPLPLTAENVDAVLDE
MRPYLMADGGNVAVAEIDGGVVRLELQGACGSCASSAMTMKMGLERGLREKIPEIIAVEQVAPDGPQLTEDGIEGVLEEI
RPFLKMAGGDVELVELIETGVAPSCTLRISGSGSTINSVRAEIAQRLKRNFPSLANVMWDAEGP*

>jgi|Bigna1|48123|estExt_Genewise1.C_220195
MAAGLGKLRSVGFLLLLSASVVFVLLSARSRNYDEKLQLGTTMKAGAQNRVMQVGRRSTIHQPLKALKMCGDFKRGVVPV
RAIASGYRPSVFTVERLDLTPANVEVVLDEVRPYLQADGGSVDFVEIDGPLVKLRLKGACSSCASSSTTMQLGIKRRLQE
RIEGVGEVVDVGVTEEKMEVSLENIEKILDEIRPYLVGASGGSLDYVGGLEQNNVKIKITGPAAKIMTVRVAIQQKIKEK
FPNMVRVELVA*

>jgi|Phatr2|46593|estExt_fgenesh1_pg.C_chr_100324
MKTTAAFLSLYGLSMASSFVPTAYRHPKTVGTTLLRDQIVSPFDSSERDEGSVATTELDAPPTKLVGPLDLTWDNVEAVL
DEMRPYLIQDGGNVIISDIDGPVVKLELQGACGTCPSSTQTMKMGLERGLREKIPEIQEVIQAMPEGPELTSEQVDVVLD
GVRPFLQVAGGSINMDRIDGVDGLQPTIWLKMEGSSASLNSVKLEIAQRLLRHFMVAGLQVQWIV*

>jgi|Ectsil1|20192|rna11414
MPLTMRRGSSFVRGIAVAAVAATSLQNSRAGAFAPPAFVPAGPAYCRSCSSSSTSALARTAAASSSCSSAQQQARGWGAA
PRRTGAAEPRRMSSTDIESPFATPGMADMGDEEEDPEALLTLTLENVETVLDEMRPYLMSDGGNVRVVEIDGPVVRLELE
GACGSCPSSTMTMKMGLERRLVQRIPEISEVVQSIPNGPELTVENVEKVLDGVRPFLSVAGGSINIQSLTGVSSIQPVIT
LKMTGSSASLKSIRMEIMQRIQREFMMSSLRVEFAP*

>jgi|Nanoce1779_2|560105|estExt_Genewise1Plus.C_300063
MARDLSCGAASTFVLVCLVLVLSLAEAFLPAIPKTPATTRRFSSPETITSPFESGVSSGPNDDFLPLTFENVELVLDEMR
PYLMSDGGNVVISEIDGPVVKLELQGACGSCPSSTMTMKMGLERRLREKIPEISEVIQAMPEGPALTTENIDGVLEGVRP
FLMVAGGSISVKEISGLGGLQPRVLLHMEGNNQSLFSVRMEITQRILRNFMVAGLRVDWTDQEGVL*

>jgi|Ochro1393_1_4|919954|fgenesh1_kg.49_#_775_#_TRINITY_DN17531_c0_g1_i2
MTMLFSCLALILFIVVREASSYSHGGLIIRSSRQYRHTYGSWSRRLSSLCMSDSETIISPFDASRSGDNMPMPSSGDDDD
DDEYDLPLTRDNVEKVLDEMRPYLQSDGGDVKLAEIDGPVVKLELVGACGTCPSSSMTMKMGLERKLKEKIPEIAEVVQS
LPDAPELTEEAIETVLDGVRPFLAVAGGKISVDSIKGVGGLQPVIGLKMEGSAAALQSVKVEIMQRIQRHFMLSLRIEWV
D*

>jgi|Auran1|6483
EQFPLTWENVELILDELRPYLMSDGGNVRIAGIEGPVVKLELEGACGTCPSSTMTMKMGLERRLKEAIPEISDVVQYLPD
TPDMDEASVDEVLEGVRPFLSVAGGTVDLVSLSG

>jgi|Vitbras1|20756|Vbra_18723.t1
MISLLLAPICIFVCLSSTRAFFQPHEPPAFVLPPAGITSASRPPASGRQRASHAEIRLHSASPATEAKESPFENLREVEY
RRGAETLPLTPENVETVLDEVRPYLIADGGNVSLKSIEGPDVYLQLEGACGSCPSSVVTVRMGVERRLRERIPEINSVEA
VSEGAENGMAFTKENVEKILDGVRPFIKMTGGDITVVEANKVGSVAATVALKMTGRSAENRSIRLEIQQRLRRNFPLLNS
VTFV

>jgi|Vitbras1|20756|Vbra_18723.t1
MISLLLAPICIFVCLSSTRAFFQPHEPPAFVLPPAGITSASRPPASGRQRASHAEIRLHSASPATEAKESPFENLREVEY
RRGAETLPLTPENVETVLDEVRPYLIADGGNVSLKSIEGPDVYLQLEGACGSCPSSVVTVRMGVERRLRERIPEINSVEA
VSEGAENGMAFTKENVEKILDGVRPFIKMTGGDITVVEANKVGSVAATVALKMTGRSAENRSIRLEIQQRLRRNFPLLNS
VTFV

>jgi|Symmic1|41120|rna29028
MLAPAARTGQRPARVLSAAPTPALAARIEPRDRDFGLALPAIAVAVNCRRGGHLRRIVRRATVVSPFNEAEAKEKEAEKP
RESEKKEKPKLSLTWDNVQEVLDELRPYLKSDGGDCKISDIQDSVVKLELIGACSSCSASSVTMKMGIEKTLKERIPEIS
EVVAIDQEQEPLTESGIEEVLNGIRPFLSVSGGSIEIFELVDGEDAKVVLKMVGPPLKSMAVRVEVQNRIKRKYPAVQNV
GIVGEDGQPPQSA*

HCF101 orthologs

>Cre01.g045902 HCF101
MMKTRAGVQRSGCPAVARTFAATQPSGAAAAAAARGVAAWVSGVAPATAAARPAAASRRGAHVVTAAAAAPPAVATAAAPRT
REEEVLAQLRNVIDPDFGEDIVACGFIKDLAVDEAAGAVAFTLELTTPACPVKEMFQRQSTEFVKALPWVRDVAIKMTSQPAK
PLLPESGRPGGLAKVRHIIAVSSCKGGVGKSTVSVNLAYTLAQMGAKVGIFDADVYGPSLPLMVDPEVKVLEMDPATKAIYPT
QYEGVKVVSFGYAGQGSAIMRGPMVSGLIQQMLTTADWGELDYLVVDFPPGTGDIQLTLCQTVAFSAAVIVTTPQKMAFIDVA
KGIRMFARLVVPCVAVVENMSYFDGEDGKRYFPFGQGSGERIQRDFGLPNLIRFPIVPDLSAAGDGGRPLVVSSPASATASTF

MDLGAAVVREVAKMGAAGRAPRQTAHFDQEQDAIVVALPGEPEFLLPPGVVRANDTSATSIDEWTGQRKREEAPADARPVAVN
PLGNYAVQITWSDGFNQVASYELLDELKSYAVPRRPVSGGSASGNGSVSTSLPGGMQAQAQLA*

>jgi|Cyapar1|4286|g4162.t1
MFASPAVLPASAASSLNAACTASCSTFAGARSRPTRSVQARRFDLATDSGFRRPASFEGSTHYIASSGAQLKRAARRSLG
CGAPACPPAVSPVLASATAAKEEEILAQLKNIIDPDLRTDIVSAGFVKELKIDEAAGRVTFTLELTTPACPIKDLFKADA
EKFVKELPWVKECEATLSASSNPAFSTEAKGLKQVANIIAVSSCKGGVGKSTVAVNLAYTLAKMGARVGIMDADIYGPSL
PVMVSPASTVVSYSPENLIVPLEYEGVKLMSFGFVQKGGQAAVMRGPIVSNVLNQLLTTTDWGELDYLVVDMPPGTGDIQ
LTLCQLVNITAAVIVTTPQKLSFVDVVKGIQMFDKVSVPSVAVVENMAYFLCESCSTPHFPFGKGSKEKLVQQFGFENSF
DLPIQTSVSDASDKGIPYTIMEGADPATLDAYKSLASAVVQEISKLKFGGDLDRPAVEFDKAANLIELKYPTRGENLGRV
RRLNPATVRRKCRCAACVDEFTGAQILQPESVPETIKPVSISPVGNYAVSIAWSDGHPSLFPFRALGTIPEAAPAEAAA*

>jgi|Tetstr1|444905|TSEL_003356.t1
MCRLSASSVAQGDMSKCGHDSSDPNGLSVRGGDAARVAQRVAVRFEFRSRRSRACVARRGRAIPALASAAAVSYEGQVLK
QLERIIDPDFGMDVVACGFIKDLEVDSAAGKVSFRMELTTPACPVKHVFESQAKEYVGELDWVKEVALTMDAQTQTHMME
GDGRPGGLKDVAHVIAVSSCKGGVGKSTTTVNLAYTLAQMGAKVGIFDADVYGPSLPTMVSPELRVLQMNQETKAITPVD
YEGVKVVSFGFAGQGAAIMRGPMVSGVIQQLLQTTDWGKLDYLLIDFPPGTGDIQLTLCQSVAITASVIVTTPQKLAFID
VAKGIRMFAKLMVPCVAVAENMSYFDGDDGKRYYPFGSGSGERIQRDYGMENLIKFPIVAELSAAGDGGKPLVVSDPTGE
VSKVYGELGATVVREVAKLKLMRRNSVRFDKEIGALVVKLPGEAEEEFLLSPALVRRNDTSAASINEWTGEKMLDDAAVP
EGVRPETISPLGNYAVQITWEDGFNQVASFDLLAGLERMALPEGYTGPSSGGGSAAQEILSAATASAGSE*

>jgi|Praco1|16810|PRCOL_00006186-RA
MRRSVGADAPVAESGRTAEQEEVLDALRSIIDPDLGDDIVSCGFVKDLDCDRATGAVSFRLHLTTPACPVKDRFQEDAEN
VTLALPWVESVDVALSAAPPKPLIPQMPEGLQKVSSIIAVYSCKGGVGKSTVSVNLAYSLAQMGARVGIMDADIYGPSLP
TMVSPEVTVLEMDATTRSIKPVDYKGVKCVSFGYAGQGQAAIMRGPMASGVVAQLLTTTDWGDLDYLLIDMPPGTGDIQL
TMCQTVPLTAAVVVTTPQKLAFVDVAKGVRMFARLRVPCAAVVENMAHFDAGGERHYPFGKGSGERICEAYGVPHLLEVP
IEPALSAAGDCGEPAVVSAPTSDTAKVFGELGAAVVKEVAKLRSGRTEVLVGFAGAGEGEECGSGGSTPIISVDAPGFDR
ALVLRAVDVRRSDRGAASVDEWTGEQLTDPASVPEDIEPMEMAALGNYAVQILWPDGLNQVAPLDVLQGLPEVDIPQL*

>jgi|Chlat1|652|Chrsp103S01053
MASVVAGLVQVVPSVTLLSSPSCSTTTAAFSFKRAPAAASAAPAAAASFSGRPLAPPTVGFSSRRRRHRRQHADVVVASA
SANPSAAQTDSAASTSDSGLERDVLGALRRVIDPDFGMDIVACGFVKDLICDASTGQVSFRLELTTPACPVKDMFESQAR
EYVGALPWVKDVSVKMSAQPPKPLLPNDVPPGLSRVSSIVAVSSCKGGVGKSTVSVNLAYSLAMMGARVGIFDADVYGPS
LPTMVSPEVRVLRMDEQTKAIQPTEYLGVKCVSFGFAGQGSAIMRGPMVSGVVNQLLTTTDWGELDYLILDFPPGTGDIQ
LTICQVVPISAAVIVTTPQKLAFIDVAKGVRMFSKLNVPCVAVVENIGREEILSVREGVVSDFGIPHLFQMPIAPTLSAS
GDSGQPAVVEDPTGQVAREFGELSACVVQQVAKLRQAAKNAVVYDADRKAIVVRLPGSQDTFALHPATVRRNDRSAQSVD
EYTDVAEDIEPASIAPMGNYAVSITWPDGFVQVAPFEQLAQIERLILDSATVVNTPPSNTNVSSEADDVSPASAAQAIMK
NAERRREQQTAAA*

>jgi|Mesovir1|2059|Mesvi1495S01046
MPEAKESVLGALRKIIDPDFGMDIVSCGFVKELEVDPATGKVSFVLELTTPACPIKDQFEQQALENVRALPWVTDVKLKM
TAQPPKPLVPTDVPPGLAKVSNIIAVSSCKGGVGKSTVAVNLAYSLAMMGARVGIFDADIYGPSLPTMVSPELRVLQMDA
ETKAIMPTEYLGVKLVSFGFAGQGSAIMRGPMVSGVVNQLLTTAEWGELDYLVLDFPPGTGDIQLTLCQVSGRQAGPSKK
TTPKKATPKKAARKKRPAPSSSSSSEEDKGLQEDGSPSTSSEYVPPSSRKERPPPPVVIPSSSDDEDEDVKKGKAALLPK
AHVFCQATGVARPGRPEDLVWTKPAKPEASPAPSTKPAKPEASPAPPTTKPSKKPAERAEGQLALPALVRLDQHVAQHDL
AKRVRGADTMGDGGGRVHQIKNAVAYDEYRNVIVLKVPGANEEILLHPATVRRNDQSAASIDEWTGEQRLRPEMVAEDIK
PQAITPVGNYAVSIQWSDGFNQTTAPVSIQWSIGVNQDLA*

>jgi|Klenit1|113|rna-KFL_000020280
MAACKALLKPALSCQGFALQQGRRGKLRQPAAPCLRTAYLQTSPSPAPLSSQNVAAKGWRQILEPPAQGFHTNSRPGLRG
DDRGGARCQAQAQSNGAAMGASPEQEVLGKLSQIIDPDFGTDIVSCGFVKEMQIDPNTGVVSFTLQLTTPACPVKDMFES
QARELVSQLPWVKDLSIKMTAQAPRPMTDEDVPPGLRKVANIIAVSSCKGGVGKSTVSVNLAYALHGMGARVGIFDADVY
GPSLPTMVSPEVPVLQMRVEGDDRIIMPTEYEGVKLVSFGYAGQGSAIMRGPMVSGVVNQLLTTTDWGELDYLVLDFPPG
TGDIQLTLCQAVPITAAVIVTTPQKLSFIDVVKGVRMFAKLKVPCIAVVENMCYFEADGKRYFPFGQGSGQKVVDQFGIA
SLFQMPIKPELSASGDTGKPQVIVEPAGEVARAFSELGASVVQQTARLRQQVATAVVYDPARKAIVVRPPGSDEEFLLSP
ALVRRNDRSAKSVDEWTGQQLLQYSDVAEDIVPESIRPMGNYAVAISWPDGLTQVAPYEQLENLERMLEDDDSAFAMPST
TSRVDGSSAADPAAPSSRPSATKAAGTGSVGAQAPLSDGAARILQEAARLKELAKS*

>jgi|Mesen1|2247|ME000153S01474
MVGTAASMAYASLNVGSYATSAAGARMTAGKALLPARGASLCLAGTLVPLGRHVALRRALSTRRAPGGGASCEAASAAPS
AAPASVEEAEKEVLGQLARILDPDFGTDIVSCGFIKNLQVDPQQGSVVFSMELTTPACPVKDDAKEYVQGLPWVRSLSLT
MTAQPPRPLLPDDIPRGLQKVANIIAVSSCKGGVGKSTVAVNLAYSLLGMGARVGIFDADVGELDYLVVDMPPGTGDIQL
TLCQVVPVTAAVIVTTPQKLAFIDVAKGVRMFSKLKVPCVAVVENMCYFDGDDGKRYHPFGTGSGKQVVQQFGIPHLFEL
PIRSELSAAGDCGQPEVVANPAGEVGQLFSDLGATVEFWLSPATVRRNDRSAQSIRLKYSDVEESIVPESIRPLGNYAAA
ITWPDGFTQVAPYDQLAELERLVDVRPSGANLEKNKDAALRIMEHARALRDAVRVNP*

>jgi|Chabra1|337696|rna-CBR_g30185
MALRLAQVILWSPSASPGFTRNDGSPPAVASGAASAAAWLGGEHLQGRSSCSGGSGSGSNYGWNSSRRSSCSNATCSGWT

GNGGEGSFMFVSRMRAAGSLRNSGGGATASTRHLAGSGTAEGAQATAPCTSSWRKGMRAGSCRSCSVQRPNASSSQTPSG
RVTKKVAGATAGAVQTSSSIAIVKYSVGGGLGCRWGEGSTFHSHLPAVGRTGPTPSSSSTVNGLRKRNSRRKNSLCRCEA
PEADGGGGNSAGTATDGSSTTTTTTTDNGSAASTDSSVAPASKVTTSSSAAEKAVLSALAQIIDPDFGMDIVSCGFVKDL
AIDEGTGHVSFRLELTTPACPIKDEFERQARDNVSSLDWVKDVNVTMSAQPPRPLIADSMPAGLRKVSNIIAVSSCKGGV
GKSTVAVNLAYSLAMMGARVGIFDADVYGPSLPTMVNPEVRLQMDPETKSIFPSEYEGVKIVSMGFAGQGSAIMRGPMVS
GVINQLMTSTEWGELDYLVIDMPPGTGDIHLTLCQLVPLTAAVIVTTPQRLAFIDVVKGRMFAKLKVPCVAVVENMCYFE
AGGKRYFPFGEGSGQQVADMFGIPFLAQLPIRPELSASGDSGRPEVIADPVGEIARAFQEVGVAVVQECAKFRQQVATAV
RYDPQLRAIVVKPPGEEEEFYLHPATVRRNDRSARSVDEWTGEQMLRYSDVMDNIEPEGIRPMGNYAVSISWPDGLTQVA
PFDQLRELERLIDPYAQREALASSVLSPNSSERTGKPEEEDGEWEKVVEEEEETRAGRAPAMAGEGEKLTGAQTILRSAS
GLQRERHAAQQD*

>jgi|Emihu1|415743|estExtDG_Genewise1.C_280054
MHMGLHHLHNHATMRIGTSFVRRRCALATARGRARLEEEALAALNGVLDPALARCKRLAVQPDATVAVKLEFRSGAMRRG
AVEAAAAAAIEALPWARRSSVESTLSRPRSFMGSNAPPSLRHVGALVGISSCKGGVGKSTVAVNLAYALATQGGRVGLLD
ADVHGPSLPSLVRLPPGSLPLRQDAQTRLLAPAEADGVRLMSYGFVGKGASSGAVPAAVMRGPMVGKTVGQMLSGTDWGE
LDYLLVDLPPGTGDVHLTLSQTYGLSAAVLVTTPQRLSTVDVLKGVDMLRELGVPMICLVENTAHFTDDTGRTHLPFGGS
QLEAVRRHAGLRESDTFRLPIEPQVGEAVDAGSPLQLLTSQSESAHALRAAARHLVVAIGERQHGAAAGGSGSHLRYDPK
RGLVLRVLSGDHEGREFVLSKAALHALRGGAGAGDASAPLLDTVEPCETEAGQPGAKLYWCGGGATVVPLGDFPHLAAAH
G*

>jgi|Auran1|33254
MGVSANRRGESLRRVKTILAVSSCKGGVGKSTVAVNVALTLAKRGLDVGLADADVHGPSVHVLCGEPKDGAVRLAPEMDA
YGRELLEPFEAHGLKLMSFGYLNDAPAYMRGSRVSGVVQQIVASVAWRDLDVLVVDCPPGTGDAQLTLCQVLDMDAAVVV
TTPSRLSFADVVKGVALFDEVDVPVVAVVENLREFALPR

>jgi|ChlNC64A_1|133105|IGS.gm_1_00241
MSCRASAEAASNSAPAVVSKEGEVRAALSPIIDPDFGMNIVDGGFFKDLAVDGEAGSVAFRLELTTPACPIKDDFEKAAR
EYVTALPWVKSLDLKMDARPPQPLLPDDSRPSGLRSVSHVIAVSSCKGGVGKSTTAVNLAYTLAQMGAKVGIFDADVYGP
SLPTMISPEIRVLQMNPETKAITPVEYEGVKAVSFGFAGQGSAIMRGPMVSGLIQQLLTTSEWGALDYLIVDFPPGTGDI
QLTLCQSVAFSAAVIVTTPQKLAFIDVAKGIRMFAKLMVPCVAVVENMSYFDADGKRYFPFGKGSGERIQHEFGLPNLVR
FPIVPELSAAGDGGRPVVVQDPAGPTSQAFLELGAAVVREVAKLRRVPQNCVRYDQELGALVVRLPNTEEDFLLDPATVR
RNDQSAASINEWTGERTLRDSDIADDIQPVGVQPVGNYAVQINWQDGFSQIAAYELLDALPRLSPDDALARQQLRARLAG
IVSGSGGSGGEPEGGPSAAQQILQNAQGAPSAQSAPQN*

>jgi|Caulen1|91712|g552.t1
MQQSLSLTKKTQIHHLRNSPATVLKRRTLLCAQSQTSESWSGKNKKSQVLDALSTIIDPDLDENIVEAGFIKDLEISGNE
VSFTVELTTPACPIKAEFQQQCQEKVGLLDWVQNVEITMTANTVRSGAPSSDRPGGLKDVAHILGVYSCKGGVGKSTVAV
NLAYSLTQMGAKVGIFDADVYGPSLPTMVTPKHRVLEMDPITKEIQPADYHGVKLVSFGFAGQGKKSPINPHPLLFTCSR
ECYNARGDGKLDYLVIDFPPGTGDIQLTLCQTVALSAAVVVTTPQKLSFIDVAKGIRMFARLVVPCIAIVENMSFFEADG
KTYYPFGTGSGDRIQSEFGLKHLIRFPIVPDLSLSSDDGEPLVVSEPTSQIGRLFTELGVVVVQEIAKLSKKGKSLAIYD
ETRNCLRVKLPEIEFCLDPKTVRENDTSAKSISEWTRNEEALSQREGAFEIQSVSGLGNYAVQILWKDGFNQVAAFDLLM
SLPHLQPVHEDL*

>jgi|MicpuC3v2|3675|wlab.212701.1
MDLVLSPLTLSRRRRTAAQYPRRAAAMAASAAAAAAAASTSIPSSSRGRPSLARARLAATRARRAPRRRDVVDVAVRASS
SSSPSITSAPEGSREADVLDALRNVIDPDFGEDVVNCGFVKDLRITDDGDVTFTLELTTPACPVKEEFDRLSKQHVSAVP
WVKSCNVAMTAQEVTNDAPDTVEGLRNVRHIIAVSSCKGGVGKSTTSVNLAYKLKEMGARVGIFDADVYGPSLPTMTSPE
IAVLQMNKETGTITPTEYEGVGVVSFGFAGQGSAIMRGPMVSGLINQMLTTTDWGELDYLIIDMPPGTGDVQLTICQVVP
ITAAVVVTTPQKLAFIDVEKGVRMFSKLRVPCVAVVENMSYFEVDGVRHKPFGEGSGQRICDEYGVPNLFQMPIVPELSA
CGDSGKPLVLADPAGEVSGAYGAVAAKVVQEVAKLSAGPKGGLAIDEDDVAGAMETHGVKALRVQLADEGGLPFYVRASD
VRKSDKSAAAPGEARKAEFLADGVTPIPDDIAPSETNVVGNYAVQISWPDGFSQVATFAQIAALERLEVKGAGKRAEETA
AAARS*

>jgi|Galsul1|3360|XM_005704422.1
MLFVRSCGISTCSVLSQRNGDALKKCPKCCRKQYYLLDNRFRRNTRQWLGKRLCKLQGMSIQTRLSTRINMCMSDWQKQV
LELLKNIEDPDLKQNIVELGFVQNLERVAKEDGKYDVRFTLQLTTPACPIKEKFQNDAKEWVSSLLWVRNVEIDLRANEI
NRAQAGDRPLNKVKHIIAVASCKGGVGKSTVAVNLAFTLTKLGGKVGIMDADIYGPSLPILVQPENKIVQYKDGRIIPLE
YENVKLMSFGYINPESAIMRGPMIANMMNQLLTETDWGSLDYLVIDMPPGTGDIQLTICQTVSLDAAVIVTTPQQLSFQD
VIKGIQMFGKVSVPCVALVENMAYFEPNDIPDKRYYLFGHGKSQKIANDYGIPFVESFPLDPDLCRWSDNGIPAVLALSE
SKISQLYQSLASAVVQQIAKNAFGNGKRIPQVFFDSDKCIIVISCNDQQGIAWNENNKVEWSPWELRNACSCASCVDEFT
GKRHWKSVDRNVKPLQIQTAGNYAFSVIWSDGHQSLYPFERVMNPKDWSTTTAVKRASTI*

>jgi|Chlpri1|4217|rna-gnl|IITBIO|A3770_06p42170_mRNA_A3770_06p42170
MSAPLECLRAGGRAGRLAGRTACQRATTSRRCGSQDAWCGAGWGWRRRLRKGGRRLCHETRVATACSTGEVEVTPQGGQE
EAEGAELPKGLRGVKHMIAVASCKGGVGKSTTCVNLAYTLSQMGAKVGIFDADVYGPSLPIMVSPDYGKSKLVMDPETKE
IAPVEYENVKLVSFGFTTEGSAIMRGPMAGGLVNQLLTTTNWGDLDYLLIDMPPGTGDIHLTIGQAVPIACAVVVTTPHK
LAYADVAKGIRMFARLDVPCVSVVENMSYFDGDDGKRYFPFGKGSGEQIQGEFAIPHLVRMPIKEEVSSGSDEGVPCVVS
DPLSDVAGSMQELGASVIKEVTKMGTKRKHKVKYDPNMHEMHVSLFEEGSEGGEGRWDSFEIEPLVVRKNDQSAKFIDQL

TGEPINQEFSPHIFPLEIKPLGNYAVQINWSDGFNQIATYDQLRVMERIQ*

>jgi|Nanoce1779_2|579071|fgenesh1_kg.4_#_1139_#_TRINITY_DN2326_c0_g2_i1
MKAALRKKDFVPTIIIIVLALLLGAVLQCRAFTPPQQAPMSSTRRPQVSSFLPPSRRGPSASRSSVLTHTPLPYATLSAE
QSPVDSVSSSAVASSNDENLTQVITQLREVMDPDLGSDVVTLGFIKNLTVAGETGSVRFVLELTTPACPVKDDLKAQAIR
VVEKLPWVSKAEVILSSRPIDADRPASANIKATGLSLVNNVIAVSSCKGGVGKSTTAVNLAFALDRQGARVGILDADIYG
PSLPTMVIPDTEVVEFVESQIRPMVKLTGVDAAGQPKGVKLMSFGYVNPGAAAIMRGPMVIQLINQLLTLTSWGELDYLI
LDMPPGTGDIPLTLCQSLNITAAVIVTTPQRLSFTDVVKGVDMFDTVNVPCVAVVENMAYADASALDDAASRGASDPQGS
ASTVGSKPWKRLRQEIEEVIKAAEDTKTQAGLVDELLATVLRRLQADRVARPLFGRGHRQRLADMWGLTNTVSLPVLEEV
SRSGDSGVPLVLGQPNSAPAQVFMDLAGRVVREVAKLRHDRSRLRPSITYLPEEHMIAVKMHGYSDTGGAEAVAPPQKMS
PAALRRQCRCAMCVEEMTGRQILRPEDVPEEIKPVEIGAVGNYAVGITWTDGHKSLFPYRSFVDGYDKDMKPAKESR*

>jgi|Ectsil1|21518|rna9702
MHMQLKRPCVVAAAVASLQCLCSKAFVQPFSQHRAKQGIWPHQYSCKSTSLPVQQRARSYHVSMMTETTSDTPPGRKDEV
LAVLSAVMDPDLSMDIVSLGFIKELEISGEDEGRQVVTFDVELTTPACPVKAQFQQDCRDLVEALPWVDRAEVTMTAQPV
RDVSDTVPTGLSKVATIIAVSSCKGGVGKSTTAVNLAFALDKQGAKVGILDADIYGPSLPTMVKPDREEVEFVGNQIRPM
TAHGVKLMSYGFVNQGAAIMRGPMVSQLLSQFVTLTSWGELDYLVIDMPPGTGDIQLTLCQVLNITAAVIVTTPQKLSFT
DVVKGIDLFDTVNVPSVAVVENMAYYDAVDQTVFKTGLESNIEDLLTLDGDELSAAAAREGLASPPQDETALRAAVIAEV
MKKRASTKQREYIFGKGHQMRLADMWGITNTIRMPLVADVASSGDSGIPFVVSKPDSDHSESYSQLAEAVVREVAKLKFS
DNDRPMLSFQPAEGTVTIETSGGKQVMAAADLRRQCRCALCVEEFSGKPLLDPASVPENIVPTEFAPIGNYAVSVKWDDG
HSSLYPYKNFAMGYKPRTKRPELSPV*

>jgi|Vitbras1|5621|Vbra_12538.t1
MAHPRQRRQQRQHERPTFLCAVTVACVVSSAAAFVPVQLIGGERGLRPAPAVRQGHHHDSQQRRASTLFLTGLSDEAPGV
GGGVDERRVECLKSLKGVIDPDLGQDIVTLGFVKGLTIDESTGNVAMTVELTTPACPMKDQFRESITSALEVLPWVNGVD
ITFTSQPIGTRPSPLSLDGQRQQHAPGGTSRVRAIIAVSSCKGGVGKSTTAVNLAFALKHMGAKVGILDADVHGPSLPTM
VRPSEPVVTIEEVPAEDGQTVQILKPIDHDGVKLMSFGFINPMAAVMRGPMVTNLLKQFVGYTDWGELDYLVVDMPPGTG
DIQLTLCQSLDITAAVIVTTPQRLAFTDVVKGIDMFDTVNVPCVAVVENMAHLTTDDIDTDRLARDIAAATSMTDQQLRD
QLSIQNEDGESVGREELVRRCVDRALRSPDVRRRKRMQLFGKGHRQRLSDMWGIDNAFSLPLLPSISRASDSGTPFVDTD
SDTADSDSETGIGPAEREAKDTYKNIAQSVVQEVAKLQSLSRDKRRPAVLYLSEGNELVVAWQGQQTAIPPAGLRRKCRC
AACVEEGTGRQLLDPSSVSEDIRPTRLEPVGNYALGVDWSDGHKSIYPYRAFVPGYTSRRDTAYEQLESSRDNGDIVLLH
RVPATRRTESEKRETAAAAAAAAAAAAAAAPVAS

>jgi|Phatr2|1494|gw1.3.92.1
EVLSTLKSVIDPDLGSDIVTLGFVQNLKLDGRDVSFDVELTTPACPVKEQFQLDCQQLVQDLPWTNNIQVTMTAQPSVQE
TATLGMSQVGAVIAVSSCKGGVGKSTTAVNLAFSLQRLGATVGIFDADVYGPSLPTMITPQDDTVRFVGRQVAPLQRNGV
RLMSFGYVNDGSAVMRGPMVTQLLDQFLSVTHWGALDYLILDMPPGTGDIQLTLTQKLNITAAVIVTTPQELSFADVVRG
VEMFDTVNVPCIAVVENMAYYESADPEKIQIFGAGHRDRLSQQWGIEHSFSIPLLNKIAANGDNGTPFVLEFPDSPPAKI
YQELASAVVSEVAKTKFAKSMRPSVQYDAESHLLQVSQNGVGSTDEEHVATLPPAELRRACRCAACVEELTGRQILVPSS
VSDKIAPRNMVPTGNYALSVDWSDGHRSLYPYRQIRAL

>jgi|Symmic1|13436|rna10656
MPAAILLSSKPPAAATCSSSSSSTGQPPATSGTSKGKKVVLESTQKTDQSESKFSLRMCATGDVRYWITLNEPWCACALG
YGSGEHAPGHREDAGKEPYLAAHHMLLAHARAVKCYRHSYQHIHGGSIVSRQKDGTKFAAARIPRHLPRKPTEPIPSLHG
LAKSLLWLKKAMASGMAFASLGRPWPHQVQPGTAAERLQRSAPSGAPARDGSLRSSVATATFAGLAAGLRNSSAGRRGVS
RAAGTAGPTPIQEVMHALEQVKDPDMGTDIVSSGFVGQIKAERITGNISLVLEVEMYKAEVEERLSQIPWAKSVEVRVGA
VRQQTSATNPPRPMPQMPDSLKNVKTILAVSSCKGGVGKSTVAVNLAFSLYQKGYKVGIFDCDVYGPSLPVMLRFQEDTP
KMEMYQDEQKQKHIRPVIDPNTGIKMVSFGFVGHAAVMRGSMVTGVMSQLVTQTDWGELDYLVLDMPPGTGDIHLTLSQV
CQITAAVIVTTPQKLSVIDVERGISMFSQLNVPSVAVVQNMSYMSLPNGERQYIFGQTDAGYAIADTFGIEQVFELPIEA
SVAAAGDSGKPFGNKGDSETAKQMDKLTEAVITEVEKIHDNRQQPELSWDGELRILRLKLAGGEELGIDPRELRLRDKGA
GGVTPPPADLSPDEIVDMGNYAVVIKWSDGVVQVAPHKWPGSLSTETREVPCLMSNWRLPEARTLKSDVAGVIPAYLVSE
TCITLNMDWKEPLSDSAADQAAQRRALDWQLGWFADPIYKGAYPATMRERCGDRLPEFSDEEVAMLKGSSDFFGLNHYST
DFVSQGEDGPPAVPNYFADQDVRNVSDPRWQRTDMGWDIVPWGFEKLLSWIQKEYDPTGGILVTENGCAVRENTEAEAVQ
DTARVEYLQGYLAQLHKAMANGAVIKGYLVWSLLDNFEWAFGYAKRFGIVRVDFTTQQRTPKASAQLISDLCKGGKLKVP
SRVQASSEFFPYNGRGKEPEVEPKKVAAPPALSKADAKRMLEEFVMRYQDDHFQSKMVSCFQQYLIHNDEMRLLKARRSL
CMPIQAEIIPKYGFEPTARGVSRVQATLSAPALTEDPDIKQMNELVVYLTGDFPKTKAAEGATV*

>jgi|Ectsil1|22518|rna7047
MLALPYTCRWHPLAGRARARHLVDVSVRRNKPTSCSTSRRGIKVAGAAAQGRRTAAASDTTSTIAALLSPQILQSRRFIS
TAPGPSLATDVSWHVAHYRNGGNARQLPRRRHMSLWGGWGQGTSGGPNSSPEEELARRWRQEKEEEVLAILSGVVEPCTG
KDVVELGFVQDVRIDEVVHGEQPQDNTGEDSAEAPLAISFTLRVPTLALPGRDTLASECEAALLALPWVASANALTKVRR
PRWRRTVQRRSTPGSILNRSVGTETTPGGGGGGGGGGAPSPGLESVQDVVCVSSCKGGVGKSTVAVNLAYSLASRGAKVG
LLDADVYGPSLPTLVNPDDVALRVSPAFPDLNLLSPIIHRGVACMSFGWVNAKAGVPGAGGHGAAVMRGPMVSKVINQLL
LGTDWGELEYLIIDMPPGTGDIQITLGQALQMSGAVVVTTPQKLSYVDVVKGIDMFAEIKVPVLSVVENMAYFDCSNGER
HRPFGPGHARELVEECGLASGCVFSLPLSPAVARGSDCGDPVSLSSPDGEEAKVYLSLADGVVRETFRAGKTAADVPEVS
FKSGRGIVLRYISEAEAAEFVIPPFELRTRDPATGEPLASEQAAAVSDDVEPVNISVRGNYAVSISWSDGHRGAIYSYQV
LRKIVEQRNR*

>jgi|Vitbras1|22565|Vbra_10576.t1
MDDALVDGGGEGQPRDDRLREEVVHELKKVIDPDLKKDIVTLGFIKNLKIDFTKGAVSFTLELTTPACPVKDQFVQVCTS
LLEALEWVTAGSVQIDITSQKASSSRQQPGSEGIPPGLRQVSFIIAVASCKGGVGKSTVAVNLAYTLQHMKAKVGIIDAD
VYGPSLSYLIPLQCDGVRGTPDGLMVPLEHRGVKLMSYGFIKPGDYAALRGPLASGMVRQMITGTAWGELDYLVIDLPPG
TGDIHMTVAQSVRVHGAVIVTTPQQLSLVDVEKGLRVFDQLKIPTLMVVENMSYFTCGTCHTRHEIFQRPPHTIARLQDE
YGIDRTHWCRLPIDQRLTVPQEATHTQQRREGDDDAYEPLECPPATFPFVLTVDSHDPVWESFRQIAETVARGLSASRFA
DNRPRVRMTDDMMMEVTIPCASPPPPAPPPAAVSNGPAVADNGTSGEGQGATNGHVGALEASELMSGVAGEDVLLVPART
VRLACRCAECIDEFTGKAKLQPWRVPRDVVADRINPTGNYAVTIHWSDGHESIMAYAVLERVARKHADQQRPKGD

>jgi|Ochro1393_1_4|134025|CE134024_10137
MIESTKLVCLLLCMCIYGVSGFHHIRFFRRNTGPIICNPIVHRTVMPIAPVNKCNRKFLSGVVEFKEGGEQENFRNDVVS
ALQNIIDPNSGTDIVEAGLVGEIFVQNNNVEISLLIDKTSVSIADEIKKLCMLEMSMLEWVSDIDVQLKNREAVETATSD
AVAEETNGMAGVKNIIAVSSCKGGVGKSTVSVNLAYTLSQGGAKVGILDADIYGPSLPTMTQPLLRDRMYEDNKLIPLEY
EGVKLLSLGFINQGAAIMRGPMVNQILNQFVLMSSWGDLDYLIIDMPPGTGDIQLTLSQIMNITAAVIVTTPQKLSFVDV
VKGIDMFDTVNVPAVAVVENMAEYSTYSFTDDFYSTLAAKIGVVVTSATESTIAAAAATNTTTTTTNDAAVAIASTIKEA
VEAQRQPRKVFGVGHNRKLEEMWGINNIVSLPLLDEVAASGDSGRPYVLSHPESKLAAQMKALASSLTSELENLAKSTAS
GPTLTFDASTGMMEYRSKKLSSFDLRCDCRCAVCVEELTGEALLDKSQVSRTVRPLGTAPIGKYAISVDWSDGHKSLYPF
RQIERLAETLASA*

>jgi|Guith1|156868|fgenesh2_pm.3_#_26
MSEVLASLAEIDDPVLGESIVSLSAVKNLEVEPSGDVSFTVQLSAPDLQGVVKQACQSALSQLPWVKDIEITVTSLPPND
SLREASRNQATGLKGVKHVILCASCKGGVGKSTTAVNLAYSMHKRGFKTGILDVDIYGPSLPTMVKPERPFDPRRDIVGN
EIMPVNGFGVKLMSMGFINPVDSFVMRGSRVTPLVQQLVSTTAWGELDYLIIDMPPGTGDIQLTLSQMETLRIDAAVIVT
TPQRLSFVDVVKGIEMFDKVGIPSVAVVENMAFFQNDGMQDNIQAFANKYSLPQEAVSELEEILKAKQFLFGQGHKQRLL
DMWGIQNSISIPLFPDLAKQSDSGMPYVLAFPDTDVSRAYSALAESVISEVSVAKMESGKNVMPEIEYDAPNKQFVIDGN
QRISAKEMRRLCRSPANDPNNVKESVEPVDFVPLGRYAISIQWNDGHQSLMPYRSFVNSYK*

>jgi|Bigna1|87067|estExt_fgenesh1_pg.C_160147
MVPPHTWSPTACLLQALIIILGGGRESPWDSRKFSLAIAQRLRRLPSQRASISLPLSRIRDMSKAGRLVSSNPSELASGPV
RRFHHRAHPRCRVENTDSENDASPLVDPELLESLASIEVPDQAKIGGSKASIDIVKSGMVREALGEKALGIASVLVELPL
PVDSKTKQALQQQCENAVKTIEWVTNPLIVITAPEGAPSAVMSSSPSSSASSPVSPFADMGNSEVDTMRKGGLKSVDKVI
AVASCKGGVGKSTTSVNLAFALRAQGYKVGIVDTDLYGPSLPTMVTPENSEVAFENGGSEIIPVEAHGVKMMSMGFINPT
DSMVVRGARAAPLVEQMLTRTQWGELDFLVIDMPPGTGDIQLTLSQSVSIDAAVVVTTPQRLSFADVVKGVEMFDKVGIP
CVAVVENMAFWQNNELSKRVEALLDEGASSSPLLPEATRKSLLDLVSAPQYIFGKGHRKRLAEMWGIENTYALPLEPALS
EAGDSGCPFYLTGASSNPSIASQYDRLASDVVTEIHNLEKTKAARPEVIFDATRNMVVSITKGGEEQIISPVELRRRCRS
PTNIKELITDDVKPIDIFPMGNYAVQILWNDGHQSLMPYASFVEGYRTNDNSVNSQLEAA*

PFLA orthologs

>Cre05.g234636.t1.1, Pyruvate formate lyase activase
MLKAALPRIMNAASMSCAATGLQRSGPLAMNAATTSRTGPASGLPKHSWGASARRAFVAPATISERLQPKLSTNYSVVLPQYE
PTDPSGIPEVFGNVHSTESMSAVDGPGVRFLVFVQGCAMRCLFCSNPDTWTLKGGNKTSSKEIAADIKRVRNYLKPRGGITIS
GGEAMLQPHFVSTVFQEVHALGLNTTVDTTGQGTKAGNWDVVLPHTDLVLFCIKHIDPIKYEALTGMKQRGALRFADQLAERK
IPFYLRYVYIPGYTDGVKDIDKLIEWCKQQPTFQGIELLPYHVLGRNKWEVMGLPYPLDGTNTPPHEQVRAVIKVFNDNDVPV
ICAE-

>jgi|ChlNC64A_1|57698|estExt_fgenesh3_pg.C_90008
MLSLLAQQVTSLGPALLRPAAIPLLHALQRGFACSAAAHQASAEPQPAEEVPPNRRFAARGGFDFATAAPQTPAEVPEVVGSV
HSIESFSAVDGPGVRFLVFVQGCGLRCVFCSNPDTWHMARGKLTSSKDLAKKLERVKPYLSQGDHKGGITISGGEPLLQPEFT
ASVLMEAHTRGLTTCIDTTGQGMKHSHWDKVLPHLDYALFCIKSPIPEKYEWITKRKIGPALGFVDELERRQIPYWLRYVMLP
GKTDQPEDVAALIQFCRNKRSMQAIEVLPYHLLGVEKWASEGKEYPLTGMSSPSAEEVNAFLAPLKEAGIPVLCNKFNREEEE
*

>jgi|Vitbras1|3470|Vbra_11962.t1
MSEIVSYWWQYFVHHLPSHLSVGAVVILRPLWRFLTLWPKLLLPATNRRTAALPHPHSHSSHQPPPLSIPPEPEPSHENTLPK
LIVPESVRHASRLSLSSHLDSMRPAPLDIRVSKKTRHSQTSIGGIASSLTIDHSPHGALRTPQLGPPGSDKADREMMQLLHDL
SIEDVMGSKGELAREAAFMHGDTPRSSEQIPDVTGRVHSVESMTAVDGPGLRYLIFTQGCPRRCVFCANPDSWDPKGGIEMSS
KAVAKQLRKCKPYLKPNGGGITCSGGEPLMQPHFIAAVFEEAHLLGLTTCLDTTGFGNTRSWQITLPHTDLVLLCLKSFDPHK
YKELTKSTHKSAYEFAEELERRGIPWWLRFLFIPGHTDNDFEYTHCENFVKAHSSCKGVELLPYHTLGVEKWKNLGLPYVFED
MQPPPKDKVAALKQRWEAAGVPVMLAQ

>jgi|Caulen1|90458|g5733.t1
MTEVERMLHTGSEVGDPFHSSLVPSVETESPRPAEVFGNVHSLESFSTVDGPGIRYMVFTQGCPSRCLFCCNPDTWNPDGGQR
MSSHDIADKMRRTLPFMKSSGGGITVSGGEALLQPEFVSALFQEAHEMGVDTCLDTTGQGCLAEKLDEVLPHTDLVLLCIKHM
RKEVYKKITRRRINPMLKFVTELNNRRIPFWVRYVLVPGLTMESSDIDLLCEFCIKQSFIKGIELLPYHTLGVEKWKQMGLEY
PLADVVPPSRNDVEQIIQQVESYGLTVKCDLSTARQTSS*

>jgi|Bigna1|43713|e_gw1.83.28.1

MLGYTSKQQAYSCRPFFPSSVISSVKPPFSFSKTTRSLSSETLAQIGRVHSLYDFTTVDGPGIRYMIFLQGCLRRCAFCFNPD
SWDINGGTRMTADDIMKNLKDHVGYMRPNNGGVTISGGDALLQPDFVAEIFRRAHEELGVTTCLDTSGLGHESGWNKVLPHTD
LVLLCIKSANPRTFFDLTESTNESMIKFAGHVATAKARDIPLRIRHVLIPGITDTEQETKALVRFARNQPTLEMIEVLPYHTL
GVSKWEEMNLKYRLADARTPTREEKLNFVKSLEDEGFKVLCEE*

>jgi|MicpuC3v2|4310|wlab.223208.1
MDRDDDATPRAAGASAATRRDDAPTRAKKTADEDDERRREKDRKDDPAMKTSSSPRPPPAPRDIEDLGDVRGRVHSVDSFTAV
DGHGIRAIVFLQGCEKRCVFCCNPDSWSARSGASMTAKQVFTRIQRNARYYAASGGGITLSGGEPLLQPAFAAELCDLVRARG
LTAAIDTAAGGTSAGWDVVLPRVDLVLLCVKSSIASKHARITGSADDRPFRQMLSFLAATRARGIETWIRFVLMSPPERWAPE
DEDLVGGSSGPTPPPTPPPPEDRAAFFRAIATDGDDEIAGVAALCDAHANVQGVELLPYHRFGEYKWKEMGVTYPMRGGRTPS
RETIASVKRKFEARGVRVIA*

>jgi|Thaps3|263830|thaps1_ua_kg.chr_11a000085
IIGSVHSLETFTSNDGPGIRTLVFLQGCSKRCMYCSNPETQCIVDPFSCPEVAVSDEEVANVLKRYEHFLRPNNGGITFSGGE
PLLQPNFVGSVFKKAKSIGLTTCVDTSGHGNPAIWNKCLPHTDYVMLCLKGMDMKLASFISGVSESNNQRAREFAKYVRDNYK
NIKLSIRWVLLKDMTDTDEELDALAAFTKDLSPVFTHVELLPYHILGKDKYEAMNSLYPMEGMEP

>jgi|Cyapar1|19601|g19218.t1
MVFLQGCRLRCIYCQNPDSWNASSCAGKKMTVEEIIAKVKRCLPYLRPNRGGITVSGGDPMLQPEFVRDLFTEIKKLGVTTCL
DTSGIGTSKDAWNHVLPVCDLVMYCPKAFNPETFRKVTRTDARVATTSAEFAEEMMKRNVPMQVRYVILRDHTDTEEEVEHMV
QFCKKHRSHLAAIEILPYHTLGVNKYKVLGIPYSDALEVPPKEKIQAIVQRLKAEGFPVLVP*

>jgi|Chabra1|321999|rna-CBR_g55228
MRFGHSSQSAIGRNCKHLRQHVVWEKAPIWKSLVPKSLVPKPVCERARSRLGEEPVLHRLCVVRKVLTARAAKGARVSVPDGA
ISSTVGESISDSRYVRPFARTATCLLTHSGRCATSVGKEENVEICVGLTRNDTGVRPASKEIGLRQASKDLGGGMAMATSAIS
RGINLLCPSTASASRHVDAVAMRNRWPDGAQIQLHSSPLSFANSSRPLHYRPTSSALSWSLPAATLEREMDSYCLHGLPEGLP
RKRCGGPICGAASASSSSSFYTDAAGGAVRELAKRDVVLPKRDSLGPGLDPDMKPEELEDSEGGGIGDIPMVWGRVHSIESFS
AIDGPGIRSVMFLQGCWRRCLFCSNPDTWSPTTKETKRMSSKDIAKLLERNLPYYSGGGGVTLSGGEPLLQPDFCAAIFREAH
ALGISTALDTAGYGRPEEWAKVLPYTNVVLLCLKAMDKAVYHKLVGLSNTVVMKFVEAIQKYKVQLVIRFVVLPGYTDTEQEI
EGLIAFAKTQPTLIGVEILPYHTLGVNKWEELGMNYPLKGVSPPSKEFMMEVKQRIENEGIKTLM*

>jgi|Tetstr1|421159|TSEL_012202.t1
MANTAAVPRRRWETRSRVARPEAPEQDGARDMTVTFETLQEMHESKEWEHVQAYESHHVPEMVPEIRDASYMYRTYYELDTNQ
DTDDWFWLKEEQAIPNIVGTVHSVESFTAVDGPGVRYLIFMQGCAMRCKFCSNPDTWDFKAATGKLIESREMANRIRKVKNYL
RPNNGGVTCSGGEAMLQPHFVGALFQEVHEMGLTTCIDTNGQGTKEGNWDIVLPHADYVMFCIKHMDPDKYHALTGLNLSVAL
NFAKELKARKIPFQLRYVLIPGFTDAPEDVDALISWAAEQPTMQFIELLPYHELGLAKWKELGYKYPMEGVPTPSSEQITEFM
DKCRAQAVPVKCLNFSNEA*

PFO orthologs

>Cre02.g095137.t2.1
MEAHLKAPAVGKICRQGQVARALVPAAAGRATNAKRAAVTVMATPPEAPGAGTASAPGDYKTAAATTTTTTTTGQTASNLV
VFPSPAVVSSAGKPGGKMAPMDGNEATALIAYAVSDVSFIYPITPATNMGEMVDQWASEGRTNVFNNVMSVTEMESETGAAGA
LHGALAAGALATTFTCSQGLLLMVPNMYKIAGELTPCVLHVTARALAKHALSIFGDHQDVMAVRQTGWAMLCSHSVQEAHDLA
LVSHLATLRASVPFVHFFDGFRTSHEINTIHLIDNEAIKSILAKPIYQDAIKHHRARALNPTHPHQRGTAQGPDVYFQCAEAA
NPFYDRAYDHVAAAMEEVSAVTGRVYKPYEYVGAPDADKVIVLMGSGSAAVEEAVNYLNAQGKKVGVLKVRLYRPWNSKVLLA
QIPATVRKVAVLDRTKEPGSGGEPLYQQIAMTLHEAEMADPALPRRYIVGGRFGLASKEFLPSHAAAVFENLDAKKPLRDFSV
GIMDDVTHRSLPDSRWLPLGTRTLPKGTFECLFWGMGSDGTVGANKEAIKIIASSAGMSAQAYFSYDAHKSGGVTVSHLRFGP
SPIDSPYLVQQADYLAVNHQSYMAKYDTLASLKPGGVLVLNTVFTSPDSLGKYLPDKVKKQIAALKPQLYVIDAQSVAKASGL
GKHVNMVMQTVFFNLSGVLPMEKALALLKKSITKAYERKGPEVVAKNHSAVDMAVAALKKLDIPASWSSLPTHVVNPNPPAKG
NTSRWEFIETVAKPMLALEGDKLPVSVFSPEGFVPPGTTVIEKRAIAAQVPIWKAENCTQCNICAFVCPHAAIRPALASPADL
VGAPATFGTIQAKGPGMGDLKYRIQVSPYDCTGCDLCTHACPDDALQSVPINSVLEVETANWDFAGTLPARTDIMDKATVKGS
QLQPPLMEFSGACEGCGETPYVKLLTQLFGDRLIIANATGCSSIWGGSAPSNPYTTNADGYGPAWANSLFEDNAQFGLGIAMG
TMQRRKTVRKHVQEVLAVEAEKMPLSFGLRNALTRWDEHFDEPEVANVVAKELQPLLEREKDVHPWIRQLYDERDMLHKASIW
IVGGDGWAYDIGFGGLDHVLASGENVNILVLDTEVYSNTGGQRSKATPKSAVTKFAAGGKERPKKDLGAIAMSYGDVYVASTS
LHANYGQVTKAMAEAEKYGGVSLVLAYAPCIMHGISSGMCSAIDESKQAVETGYWPLYRYNPAVKEDATHHRFQLDAKKLKGD
VEEWLSHENRFQILERKNPEVAHKLHHELDDAVHERFDRMKHMAAGGHMEPGSPPPPAAAAPQAQAPDHGHQHNTKESGCGGH
*

>jgi|Cyapar1|23463|g22964.t1
MSAAAPAAKAPTFKSLDGNEAAASVAYAFSDIAFIYPITPSSPMAEHADEWSAMGKPNLFGQVVQVKEMESEAGAAGALH
GALAAGALGTTFTASQGLLLMIPNMYKIAGELMPCVMHVAARSLAGQALCIFGDHSDVMAVRQTGWALLSAATVQEAYDL
AIVSHLSTLKSRVPFVHFFDGFRTSHEVQKIDATPLSHFKTFMDMKAIEDHRGRALNPAHPHMRGTAQGSDVFFQNVEAA
NQFYDKVPAIVEQTMNEVASVTGRPFKLVDYYGAPDAELVIVSMGSSCPVIEETIDALKGQKIGLIKIRLFRPWPHAQFL
ETLPKTTKRICVLDRTKEAGAQGEPIYLDVCTSLHEGEVTGKLVVGGRYGLGSKEFTPSMVKAIYDNLALPKPKNHFTVG
IVDDVTHTNLPLGPPMRTVPEGTVQCQFWGMGSDGTVGANKDAVKIIGDNSELFTQAYFSYDAHKSGGLTVSHLRFGPKP
ITSTYLVTEADYVAVHLESYVTKYNFLATLRPGGAVVLNTVWTAAELEKKLPASVKRQLHELRARLYIINARAISDSVGL
GKRINMVMQTVFFYLSGVLPFERAIALLKKSIEKTYGKKGPEIVAMNHRCVDAAVAHLVTVPIPDEWKTAQDDTRRRAPG

PVATDFVKNVVKPMLAMEGDSLPVSAFVPGGVVPPGTTQYEKRAIASKVPTWKADKCSQCNYCAFVCPHAAIRPVLVTEE
ELEGGAPEAFETRKAKGSGEITNFKYRVQVSPYDCTGCELCIHACPDEALEAAEINDILDTEAANWDYFTTVPPRGELFD
RNTVKGSQFQQPLLEFSGACEGCGETPYVKLLTQLFGERMIIANATGCSSIWSGSAPVNPYTTNKDGRGPAWANSLFEDN
AQFGYGIATGVIQRRAGLRKVIDDVATTPEGQPGFVPMSSELRSAMKAWLDVWQDADKCESASKRLETVLGAECRRPGSS
AMLREIYKQKDLLTKASIWIVGGDGWAYDIGYGGLDHVLASGENVNILVMDTEMYSNTGGQQSKSTPMASVAQFAMSGKK
QNKKDLGILAMQYGSVYVASVALGANFSQVTRAMVEAERYPGVSLIIAYAPCMLHGIREGMSYALDESKMAVDTGYWTLY
RFDPRIKVDAEHTQFQLDSKKIKAELTTFLKHENRFGILARSCPSEAKKLQVNLQEFVIARHEKYKHLAETDAERKANLA
AQFAKLAGGLSSDQPWPSSVTVAYGSQTGNAEGVAHVIAGQLRARGVAHVKCCEANDLEIGDLPAISHLVVVVSTAGQGE
QPDNIKDFWKALENPALPADYLANTTFAVFGLGDSSYCFFCKSAIEIDERLGQLGAQRVLARGIGDDQDEDKYETGFSNW
VPELYEALKLPEGKKETGVPNKHYKVVLGGKAHLDFDLTKQLAPTPILTEGCARHVRMLSNGRLTSLDYDRNIRHMVIDL
EGSGLRYGVGDALAIYPRNAPDRVADFLGFMGLDPLECIDELQFIEAEGQTATKQKPPVPAHLPLAQLFTDVLDLFGRPS
KRFYERLALYASDPAEKAALERVCERSAAGGGAFAKLLEETPTYADVIRMYPSTRAALSIEHLVDILPVMKPRYYSIASS
PHHLPGKLELCIVVVDWKTKSGADKFGTCTGYLKDLLDFGADGKASYTIAATMKPSAGMCMPAKPEFPVICAGLGTGLAP
MRAMVQERLHQIKAGKKIGETVLFFGCRFRAKDYLYGEEWEQALKDGNLTHLRVAFSRDQKEKIYIQNKIEQEPALIADL
MLNKNGYFFFCGPARQAPNDIRAAMEKALQRGAGLTEQQAAQKITELIIAGRYVVEAWS*

>jgi|ChlNC64A_1|134349|IGS.gm_11_00195
MPHPSASPGDRGALPVTCQAAPAGPGRDKEPARGDLLRPHAVAAPPPPAPLAQPPAAKPAAARKQAPLDGNEATSRIAYA
TSDVSFIYPITPATPMGEFVDQWSIEGRKNLFGNVMSVTEMESEAGVAGALHGALAAGALATTFTCSQGLLLMIPNMYKI
SGELMPCVLHVSARALAGHALSIFGDHQDVMAVRSTGWTMLSSHSVQEAQDLALVSHLATLAGSVPIVHFFDGFRTSHEI
NKASAAGIDLISDEQMKPLMDELAPAIAAHHARAMNPSHPTQRGTAQGPDVYMQAIEAANPYHKAMPGIVQAAMDKVAAV
TGRQYHLFDYVGHPEAENVVVAMGSGCEALEAAVQNLTRDGHKVGVLKVHLFRPWSPEHLLAALPASAKRVCVLDRTKEH
GSQGEPLLLEVASSLQRARRQVECVVGGRYGLGSKDFTPAMAIGVFQNLAAPRVEDVRDRFVVGIVDDVTFSSLPYDAEP
EVLPPEITECLFWGMGSDGTVGANKEAVKIIANQPGMNAQAYFSYDAHKSGGVTVSHLRFGPKQIRAPWLVDMAHYVGVH
QQQYWSKYDVLAGLRRGGTVLINAPWKSWEEVEQFMPVKTKARVAALRPKAIPLLKESIKKAYGKKGDKIVNMNYAAVDK
ALEHLVAIDIPADFGKGVERLGAAEHAAASTGSRTAAEFLNNVVMPMLAMEGDKLPVSVFTPGGFFPPGTTAVEKRSIAQ
VVPAWNSANCTQCNICSFICPHAAVRPALATPEELAGAPAGFATVPIKGGGPALKGFQYRIQVSPTDCTGCELCVHACPD
YALTSTPTAQMLEAETTNWDFFKQLPVRGELFAKDTVRGSQFQPPLMEFSGACEGCGETPYVKLLTQLFGTRMLIANATG
CSSIWGGSAPANPYTTDFKGRGPAWANSLFEDNAQFGFGIAMGVKQRRAALEAAAKVVVGEGAGTPELRAALAEWLPIKD
NGALAGTAAAAVAAALPAANGNGNGNGAVVSEAARGALAYIQNNTDLLDKPSVWIVGGDGWAYDIGYAGLDHVMSTGEDV
NVLVLDTEEYSNTGGQKSKSSPLGSVLKFAAAGKNRPKKELGLMTMQGYPDVYVASISLEANYNQVVKAMSEAEAHKGPS
LVIAYAPCAMHGISSMSESQTDAKMAVDSGYWPLYRYKPGATEADGKLTLDSKKIKGALEDFLKRENRFMVLTRKDPEAA
GKLHHQMEEHIQLRQERLINMAKGTNGAAPPTPPPAEEEEKK*

>jgi|Tetstr1|465369|TSEL_010055.t1
MPPDGEKAAAGAATGAVVVPRGMYAMDGNEATATVAFAMSEVSFIYPITPSSTMAELADAWAVAGRRNVFGQVPEIFEME
SEGGAAGALHGALVSGSLATTFTASQGLLLMVPNMYKIAGELLPCVIHVAARAIAGQALSIFGDHSDVMLCRSTGWALLA
SHTVQECVDMALVAHLATLKASVPFVHFFEGFRLSHEVSKIDVIDMLAAKPLVDAAKLARHRSRAMNPNHPNQRGTAQGP
DVFFQAVEAANTYYEKCADVVEEVMAAVGALTGRHYKLFEYEGCPDAEHVIVIMGAGAPVVKEAIDVLTARGQKVGLLKV
RLFRPWSPERFVAEVPRTTKRIAVLDRCKESGALGEPLYLDINSTMHEEAQREGQEWLSSIAIIGGRFGLGSKDFTPGMA
LAVFAELDKPKPKNHFTLGIHDDVTFHSLAVLEEPNCVPAGTVECLFWGMGADGTVGANKEAIKIIGTSTELYAQGYFAY
DAHKSGGVTVSHLRFGPSPITSQYLVRAADYVAVHHHSYFHKYDWYSAIKPGGKLVLNCEYPADEVALRLPVDVKRHLAE
LGVEVYSINALEISRSVGLGKRINMIMQAVFFYLAGVLPFERAVALLKAAINKAYSRKGPEVVEMNYAAVDKAVAGLRKI
AYDRAAWAAGNTQYVALHARHQNDAVPAYVREIVMPGDELPVSAFTDYGGLVGGVVPPGTTQFERRQIAASVPKWVNTDK
CTQCNECSFVCPHAAIRPQLTTAEEREHAPEGYHSEQAKGAEASGLNFRVQVSPYDCTGCEMCAHACPQECLEMRPADEM
IEAEDEHWEWGKSLPSRGYLFERGTVKGSQFQPPLLEFSGACEGCGETPYVKLLTQLFGERMIVANATGCSSIWGGSAPV
NPYTTDAKGRGPAWANSLFEDNAQFGLGIATGIIQRRLALKDNVRELLAAGGGAPEVPEELALALAAWLEDADEAEGSKK
AGEEVEAELEKVRAAGGGELPAPLDEVYAAKDLLTKPSIWVVGGDGWAYDIGFGGLDHVLSSGIDLNVLVMDTEMYSNTG
GQRSKSTPMGAVVQFAAGGKRRVKKDLGAMAMAYGDVYTASVCLEADFAQVVRAFTEAERYKGTSVILAFAPCILQGLRG
GLSQSLAYAQEAVSTGYWTLYRFHPERTADPNTPPLMLDSKKVKGELQSFLDKQNRFVALKRRLPQVAAQLSAGLKASLT
AKRCSTESCAQSPEEHKKALLESFRKLVEGAEGGAAGGGGGGGAVEEVVVLYGSETGNAENLAHTLAANLKARSVRSVSC
KECDETAAEDLEEIGARGAVVVVVISTAGQGEFPKNSATWFQELARARPAGWLSKLRFAVFGLGDSAYCHFCLAAAEVER
RLGELGAVPLLPRGVGDDQSPDKYETAWMEWQPELFDRLQLAEPERLVGAPPPEYTVQLTKGHHPEWQSAPEVYPFAPIA
TDRRAFPIKLVENKRMAAPDHDRDVRNLVFDISGAKLRYFPGDCLAIYPRNALQPAAALCQWLGLEPDAMTRVKCLDAGR
ASKAKRDLPRGMTVLQLFTDVLDVFGRPLRSFYEAFYCYVDDPAERAERFPSCKANLDIAHILDLVPTIKPRLYSIASSQ
RERVGTCTSFLRSLSCDPEHDDAELNTLMVYVQPTGPGGIFHPQDAKVPVMMVGLGTGLAPFRAMVQERKALARAGVELG
ESALYFGCRTRKDDYLFGEEWDAAHKEGYLSELRVAFSRDQPQKIYVQDKIREDPALVWRLLVENEGYFFFCGPARRAPQ
QVREGVEAAFVAAGNLTKQEAAEKVDKLVGLGRYVVEAWA*

>jgi|Vitbras1|12792|Vbra_21448.t1
MKLDIVDGNTAAAHVAYAFSDVAFIYPITPSSTMAELVDLWASQGRKNLFGQKVDCREMQSEAGAAGAVHGSLAAGALTT
TFTASQGLLLMIPNMYKIAGELMPCVFHVSARAVAGQALSIFGDHSDVMAIRQCGWGMLSSHSVQEASDMALIAHLATLE
GSVPFVHFFDGFRTSHELQKIEILDYEQMQPLFDDMKSELERHRAKALNPTHPHQRGTSQGPDVFFQCVEASNPFYAKLP
EIVEGVMAKVAPVIGRTYRLFMYYGHEQADRCVVVMGAGAPVLEECVNYLNSKGEKVGLIKVHLFRPWKADALMAAIPDS
VQRIAVLDRTKESGSFGEPLFLDVAATLQDRGDGRLVVGGRYGLGSKDFTPAMAKAVYDNLAAEKPKRRFTVGIKDDVSN
MSLELGPEFRAVPEGTVQCMFWGMGSDGTVGANKNAIKIIGSNTPLYVQGYFAYDAHKSGGVTVSHLRFGPHPITSQYLL
QDADYVAVHHQSYVKKFKVLQHIRHGGNFVLNCPWTRDELETELPASLKRTMAAKSVQFYTMDAAGVARETGMGKRINNI
LMVAFLKLSNVLPFEQAVNLLKSAIQKTYGRKGEAVVHANWRAVDAALDAIVKVEYDASEWASATDKKVVDVLENAPTFV

TEVMMPVERLEGDTLPVSKFQPGGFMPLGTSAFEKRNIALQVPTVDMDKCTQCNYCSFVCPHAAVRPFLITEDEADDAPE
HFVTKKAKGGAEMAGFKYRIQVSPMDCTGCELCVHACPDNALTLYPVDNVIAQENPNWDFALTLPSRGELFDRSTVKGSQ
FQKPLLEFSGACEGCGETPYVKLLTQLFGERMLIANATGCSSIWGGSYPAVPYCVNDKGQGPAWGNSLFEDNAEYGFGMA
VALNQRRQRLHELVQSVLQGGLDISDALREQLVGWEKEWRHPAKSQAYYETLPPLLEKESGKHPLLERIYTLRDLLPKFS
HWIVGGDGWAYDIGYGGLDHVLASGENVNILVLDTEVYSNTGGQMSKATPLGAIAKFGQSGKKYNKKDLGMMAMAYGNVY
VASCALGANYSQVIRAFVEAESHEGPSLIMCYAPCSEHGYLVSTWAHQADDVKIAVESGYWLLYRYNPELEDKGENPLQL
DAKKLKVDVRQFMERENRFASLMRAEPEVATKLQENMSKFTKERFNRMKSRSQAPGGIDSAQLNATFEALQQGLATGGVQ
AGPGRFDVLFGSETGNAEEVAKRVGGLLKGRGYKVKVRELDEVEVDELAELKFVLVICSTAGQGEFPQNAKDFWTAVSDP
TLDDTVLKDLHYGVFGLGDSSYCFFNQAAKMIDEQFAKLGATRLLPCGLGDDQDEERYDTALGEWLPDFWTEIKAPAPLH
ESDELPPPNFAATPLDAATCTYEQCVPQGAVLLRLTENTRITPDDYDKDVRHLEFDLTGTNFRYGLGDSLAIFPQNDPKE
VEAFCNYMGYDADLWVQFSKSDPTQSNARYDALFKKPMTIRQLCVECLDLFGRPTRHFYESLQKCCTDSEEKSRAKHLVS
PEGKPEMRKLTDETVHCFDVLQMFPSAKPTLEQLIDMVPLLKPRYYSIASSQRYLGGNRLQLCIGIVDWKTPSGKYRVGE
CTGYVTRTPVTETSPPIFYACSLKATTFNLPPNDRVPVIMASMGTGLAPFRAFVQHRAFLKRQGKQVGPMTMYFGCRYSK
KDYLYGDELQQFAEEGVIDELRVAFSREQKEKFYVQHHITRDPALLYRRFIEERGYFYLCGQSRQVPIDIRNAVLQALQS
EGGMTDKEATDKLIDMQIRGRYNVEAWS

>jgi|Symmic1|517|rna30934
MRLLSEDGPASLFKMEQSASGDMAPSSGGIGYLYFFLRRRMQRALQDETGRTTRLLWDAPGPVLEQRRLVEEPPGTRVHE
FVVLSLLCMTSGDGGAECWLQPSETEPNWATILETVVDGNAERDDVFVSVLSLEMVQALEVPFKMVATYSHSGRKATEYC
HRDLRARVNAHRKEGAATVEEIFNKLCDICVAVIPRDGKFATMPFKEARKKRAIVLWCTKHYTKEKPDDATDISDGEVEA
IVAQAAPPPEPGFYRTNTGLLCVCSPANAWWECPWCKPGISKKQHTSALRNDSRCFADSSSPTGDTVAKCRHGRLSMGAG
ACRLQALKFGVRRWLLVAQVGTTLQKTSPFGPYGGLCRQFLMRDQERQEETPQRLEQLGAGSGAADVEYEQFMKAKEVQR
VMFQQENKTIVHQCADTDIIEATTGSIACIDGNQAAAHVAYALSDCAFIYPITPSSPMGEMVDEWAAQGLINCYGQKLSV
TEMQSEAGAAGALHGALKAGQGLLLMIPNMYKIAGELLPCVMHVAARALAGQALSIFGDHSDVMACRSTGWAMLASESVE
MAQVNAIVAHLVSMERRVPVQHFFDGFRTSHEVNKIKLIDYNTMKSFINWDAVKEHHDLAMNPRHPHVQGTSQGPDIFFQ
CVEAGNSFYDGLADLFEEKGKLVQEQTGLHFALYGYEXXXXXXXXXXXXXXXXXXXXXXXXXXXXXXXXXXXXXXXXXXX
XXXXXXXXXXXXXXXXXXXXXXXXXXXXXXQPCQKRRSVASMLHASAHSEIVVVGGRYGLGSKEFTPNMVLSVFENLAKD
SPKPRFVVGIEDDVTHLSLPVGPWLNVLPEGTTECMFYGLGSDGTVGANKSAVKMIALGTELHAQAYFEYDAKKSGGVTI
SHLRFGPKPIHAPYNVRAADYMAIHKSSYVHNYDMTRYLKQNAVCVINCSWDVKELEQQLPAKMRRDLAEKKAKLFIIDA
TKIAVKAGLGKRINMIMQTVFFKLFGPKPIHAPYNVRAADYMAIHKSSYVHNYDMTRYLKQNAVCVINCSWDVKDLEQQL
PAKMRRDLAEKKAKLFIIDATKIAVKAGLGKRINMIMQTVFFKLSGRLSAVMPYEEAVEMLKKSIKKMYGKKGDKVVKMN
IDGVDASIAGIVECEVPAAWASLAVDTEASDAKTSTVAYAKGPRMFPEVQNASQFAAQVQKPCNNLDGNSLPVSAFVPGG
RVPCGTSQYEKRGIAIQVPKVDMDKCTQCNKCSLICPHAAVRPFLMTSQELGKAPTSFKEGSRSAIGGGVLDNYQYRIQV
SPWDCTGCELCVRICPADALTLGPAEKAIQEEEANWNFAVALPDRGDEIDKTTVKGSQFQKPYLEFSGACEGCGETPHVK
LMTQLFGDRLVIANATGCSSIWGGSNPSFPYTTNSKGEGPAWANSLFEDNAEFGFGMRKAFKQRRDYLALQVEDTLADQS
VKMSDELRQALQQFLVMRKEQMHDLLLPKGRSIYHQIMEKLVPLLEKEKGTHPKIHNLYDLEDMFGRSSFWIVGGDGWAY
DIGYGGLDHVIASEEHVNILVLDTEMYSNTGGQASKATPKGAMAKFAEGGKLTQKKDLGQMAMTYKNVYVASICVHVNPQ
QAVRALIEADAYPGPSLILSYTPCISQGFPMAESIQHCQMAVDSGYWPLYRYNPEAAAHGNNPFQLDSRKIKGDMFKLLA
KENRFAAVMRRDPKHAEELDNKLQENVVQKNHLLQVLNKEDLEGQFSKLVEGLSDAKSSGDSITVLYGSETGNAEEQAKN
LMQDLIARGLKSTVSSLDDFEFDELPNQKIVILVVSTCGLGEYPANCKQTWLKLQSPDLPMSWLAGVKFCVFGLGDSTYS
QFCVAAAGFDTHLGELGGQRMLKRGVGDDRDEDRYYTGWEAWLPELWKVLGAPALPLSQDIPAPSYRVDASAGDLAKPPI
SDDDIIPPGANRLTLLTNNVLTGDAKYDRDIRHYEFKIEGTNVAYKTGESLAIWPRNPEDKVMEFCQMMGFDAAQHLRIL
PLEGARNWCPTELSVRQLFSHVLDIFGKPNRKFFQTLALFAKDEAEKKQLQSVVENSQEGAALYRDLTHDFAHHADVLKK
FASARPPIEHLLQMMPVLKPRSYSIASSPLMHPDKIQLCVVLVDWTVESTKELRLGECTGYMQQQKPGAQIMCAVRSSAI
VLPKEPEKPVIMAGMGTGLAPWRAVTQERVMQARQGLKVGKCMLFFGARYKQEWLYREEFESYEKEGVLQMHTAFSREQA
RKIYVQHRIVEAGADVAERMLNQGGHF*

>jgi|Guith1|159505|estExt_fgenesh2_pm.C_220012
MIKNTTWSCIDGNQAAAHIAYALSEVSFIYPITPSSPMGEMVDEWAANGLKNIFGQTLSVTEMQSEAGAAGALHGSLKAG
TFASTYTASQGLLLMIPNMYKIAGELLPCVMHVSARTLCAHALNIFGDHSDVMAARTTGWVMLASENPQMVMDQALVSHL
ATMDMRVPVLHFFDGFRTSHEVNKVRVINYDDIKKIFPWDSVKTHHDSALSPMNPHIQGTNQGPDVFFQAAEAANAIHNT
IPAYMQKWSEKVAEITGRRYGFFSYEGHPEAENVIVIMGSGAVTVHETVKYLNSHQNQKVGVLKVRLFRPWITERFMAAL
PASVKRIAVLDRVKEVGAIGEPLFCDVCTTLHLSGMDHIKVYGGRYGLGGKDFTPGMVLSVFKNLEAAHPKTKFTVGIVD
DVTNLSLNVEQEVDMLPSGTIQCLIYGLGSDGTVGANKSAIKTIAQNTDNFAQGYFEYDSKKSGGLTVSHLRFGPHPINA
PYLVKNADYIGIHKESYLSRLDMLRNLKENGTVVINCTFGPEKVENMLPPRMKYQLASKKASLYLINAVKVGRETGMGKR
INMVMQSVFFKLSRVLPFEKAIELLKKDVQKMYGKKGEEVVKRNWDAIDRSIENLVEVPVPAQWAQIPVTTNKEIRKSTL
EFVDEIVEPVNKFEGNDLPVSAFVPGGRVPTGTSAFEKRGIALQVPRVDMDKCTQCNYCSLICPHAAVRPFLFTKAELEG
APEGIKKGSRKAIGGGVLDNYNFRVQVSPLDCTGCELCVRICPADALFFEETDKAVELEAENWNYAISVPNKGEEIDKTT
VKGSQFQQPLLEFSGACEGCGETPYVKLLTQLLGDRLVVANATGCSSIWGASAPSFPYTVNARGEGPAWANSLFEDNAEF
GLGMRRAFKQRRNQLMFLVDDALEDKTVPLSDKLRKLLSQYSVMRHEDKLDMLLPKGRSFYNQLREKIIPLLREEAKNHE
KIQELWDQADIFGRHSHWIIGGDGWAYDIGFGGLDHVLASEEHINVLVLDTEMYSNTGGQASKSTPRGAQAKFAETGKLT
AKKDLGQYAMTYKNVYVASICIHVNHQQAIRALLEAEAYPGPSLVICYSPCISQGFPMAEAIQQCRDAVESGYWPLYRYN
PLLAEGGNNPFQLDSKKISGDLFTFLAHENRFASVMRRNPSYAQAHQDRLQRQIADRAKALNVLSLDELPASLKAAGPSE
SVTVLYGSETGNAEEQAKSLFADLKARGTSATLSSLDDFDFEELPNQSTVLVVISTCGQGEFPANSHKFWMKLSDPTLPM
SFLEGIKFSVFGLGDSTYSLFCVAAERIDVRLAELGATRILNRGIGDDRDEDRYYTGWDNWTPQLWNALHVPQKPLERKI
PKPAYKVTRTQGEATPSVSNDKLVPPGSNPLKLMENTLLTPEGYDRDIRHYVFKIKDTNVEYKVGDVLAIYPRNHVDQVE
EFCKMYGLDPNEELNVVSTPEARNQIPEELNVRQLLQCVLDIFGKPNRRFYDTLSLFATDPAEKQKLELITSEDPDGKAL
YRELSHDMANHADVLKRFPSARPPLEQLMDMIPVIKPRSYSIASAPSMHPDEIELCIVAVDWEVPSTGEKRFGQCTSYLR

TTKPGDTIMCSVKPSSIVLPEDNKAPLLMAGMGTGLAPWRALTQHRIALKQQGIDVGPCTIYYGARKGATEYLYREEFEK
YEKMGVLRMVTAFSRDQPQKIYVQHRIREDYENVFRQLMKEQGSFYVCGSSRNVPEDIYNAMKEVMMLGGGMQEADAEAA
LASLKMDGRYTVEAWS*

>jgi|Thaps3|31316|e_gw1.1.16.1
AVANASYRMNDIAYIFPITPSSVMGELVDEWSATDDGQGKKKFLNLWNQELKVVEMQSEAGAAGALHGALNSGSLATTYT
ASQGLLLYIPNLYKISGELLPTVIHVAARALTGQALSIYGDHSDVMLVRGTGLAMVSSFNVQEAHDMAVITQVATLNSRV
PFLHFMDGFRTSHEISKIQLVSDEQLKELMPWEKVEEHRQRALSPLHPSQRGTAQAPDVFMQLVESSNKHYQAVNSIFGK
AMEDFARVVGRHYKPFEYHYFGSTTPTIAVVTMGSSVEVVKETLKCMNMEQACVIGVRLFRPWSPADFCKVLPKTVKRIA
TLDRTKESGAQGEPLYMDVCASLMSCNRQGIFVAGGRYGLASKDFSPRMALAVVQNLMRKDVGSIQHPFTVGIIDDVTHL
SLNLGKDVNPLPENVTQCVFWGFGSDGTVGGNKETIKIIGDSRTDMSVQAYFEYDAKKSSGWTISHLRFSDSHTLSAPYK
VQDCQANYVACHNESYAAANKFDVVRFSKKGGTFFLNTKIASIDSPDKRIKEFYSSCRALRKVKFYIMDADRLASTYGLK
GKINMICMSAFFRLSGVLPIDEAVALLKKSITKSYSYKGDDVVKKNHDILDAVCSDPRYLLEVEVPSRWRRATLTDERRA
YANRHIALIDDEKTRKFMEEIAEPVSHLEGDNIPISKFLSNEMLGGTMQPGTTRYEKRRPNPSALIPKWNASACTQCNQC
VAVCPHAVIRPFIITRDEAEQAPHPDQFQTVKALGTELAGKRYTLQISPLDCTGCNACVEACPEAPKALEMQDVEDVLSK
GGEDNWNFAMTLPERGDLVDKYTVKGSQFQTPLMEFSGACSGCGETPYFKMLTQLFGERMIIANATGCSTIWGGSFPSNP
YTVSKKTKRGPAWANSLFEDNAEYGFGMFSAMKHRRERLIQLVQEYWLECRDEKSDKCTYLFDKMQPLFEVLLSNKSNGR
QSKLEMIYSDRDMFPKLSQWIVGGDGWAYDIGYGGLDHVEAFEANDVNVLVVDTEMYSNTGGQASKSTPAGAAVKFAMGG
KQQTKKNMGEIFMTYEHVYVASVCLSNQTQLLQALVEADRHNGPSFIVCYAPCIQQQVRPEGLNDMFDECRFAVDSGYWP
LYRYNPNLINEGKNPFILDSKKLRREVASFLKREGRFINLKKAHPEIANDLFNKMNYDVHHRMENMLSRAAG

HYDA orthologs

>jgi|Chlre5_6|12867|Cre03.g199800.t1.1
MSALVLKPCAAVSIRGSSCRARQVAPRAPLAASTVRVALATLEAPARRLGNVACAAAAPAAEAPLSHVQQALAELAKPKDDPT
RKHVCVQVAPAVRVAIAETLGLAPGATTPKQLAEGLRRLGFDEVFDTLFGADLTIMEEGSELLHRLTEHLEAHPHSDEPLPMF
TSCCPGWIAMLEKSYPDLIPYVSSCKSPQMMLAAMVKSYLAEKKGIAPKDMVMVSIMPCTRKQSEADRDWFCVDADPTLRQLD
HVITTVELGNIFKERGINLAELPEGEWDNPMGVGSGAGVLFGTTGGVMEAALRTAYELFTGTPLPRLSLSEVRGMDGIKETNI
TMVPAPGSKFEELLKHRAAARAEAAAHGTPGPLAWDGGAGFTSEDGRGGITLRVAVANGLGNAKKLITKMQAGEAKYDFVEIM
ACPAGCVGGGGQPRSTDKAITQKRQAALYNLDEKSTLRRSHENPSIRELYDTYLGEPLGHKAHELLHTHYVAGGVEEKDEKK*

>jgi|Chlre5_6|18188|Cre09.g396600.t1.1
MALGLLAELRAGQAVACARRTNAPAHPAAVVPCLPSRAGKFFNLSQKVPSSQSARGSTIRVAATATDAVPHWKLALEELDKPK
DGGRKVLIAQVAPAVRVAIAESFGLAPGAVSPGKLATGLRALGFDQVFDTLFAADLTIMEEGTELLHRLKEHLEAHPHSDEPL
PMFTSCCPGWVAMMEKSYPELIPFVSSCKSPQMMMGAMVKTYLSEKQGIPAKDIVMVSVMPCVRKQGEADREWFCVSEPGVRD
VDHVITTAELGNIFKERGINLPELPDSDWDQPLGLGSGAGVLFGTTGGVMEAALRTAYEIVTKEPLPRLNLSEVRGLDGIKEA
SVTLVPAPGSKFAELVAERLAHKVEEAAAAEAAAAVEGAVKPPIAYDGGQGFSTDDGKGGLKLRVAVANGLGNAKKLIGKMVS
GEAKYDFVEIMACPAGCVGGGGQPRSTDKQITQKRQAALYDLDERNTLRRSHENEAVNQLYKEFLGEPLSHRAHELLHTHYVP
GGAEADA*

>jgi|Cyapar1|17198|g16919.t1
MTPPVLRVCPAPLLTFDQAGRPNSLAALNGIGFASRAVQRQGSQGSMKPPADPKMQHLIDVVLAAMPTILKDSNDDEEALSNL
IAKLRMEMKPEDDDHKSKDERPSEKVKKDAQVVLRRIEDKRLEKRVHCAQIAPATFVSIAEEFDLPPGSIKPAQLVQALHKIG
FDHVFDLRFAADVTIMEEASELLEKIKSGGKFPMFTSCCAGWQRLVEEDYPDLIDHVSSCKSPMGMNSALIKSMFAERSGIDV
SQIVVTSIMPCSIKKVEAQRPQLQDPISKTDHVITTREIGQIFRAMNIDWDELDGSGPTGQFASWMGAGTGAGAIFGVTGGVM
EAAVRTAYEFATGKTLEKVEVRDARGLFGVKEGERAPAPRRPPAPDAAPAPRAGALDVPGVGELKFCVVCGLTSIKRIMEMTR
AGECPFHFVEIMMCQNGCISGPGQPKSKDKGIAKKRMQALYTSDERAVVRKSHENPAVKALYQEYLGKPLGEKSHHLLHTEFW
PNHVKPENGHGHGHGHENGNGNGHAHDHMEKKALDASFSALTAGMHEEKEEAAHAEEAKPAAVVEYPPTVEWTLPPKAIRIIY
GSETGNAESLARVVAKECAARGIAVKCTEADELEMDDLATVSTLIVVVSTAGQGEIPDNAKDFWTGISSKKHPPGWLRNLHYS
VMGIGDSSYAHFNRAAVEIEERLAELGAHRFLARGVGDDQDEERFETGFEKWAPEAYKTLKLPPVKDDGSIPLPSHHVFLTAG
PKEGGARKFDETWKPIVTDPMGQLIRVESNVRITPDGYDRDVRHIVFEVDQVPEPLKYTVGDALAIYARNDPEEVKAFLKFYA
LEPSDLITFKLNEHFKGDAKAKPPMGPQTSLLDYVRPPRRPAPLSSPAEACAERGLSGGAQFCDCVDVFGRPNLRFFGALAKF
ATDVHQRDELDSIARKTWRGTAAFSVLLKDLGHVTYAGVLQAYPSARPPLAQLLDMIPAIKPRYYSIASSPHVAVGKLELSIV
IVDWEKPDKSVGFGTCTGYLRQLVDGPHWISVALKPGVGICMPPHPSTPIVMAGLGTGLAPMRAMVQERSFQVDHGKRVGWSV
LFFGCRHSKGDYLYSDRWDEFVAKGALSHLRVAFSRDGPKKVYIQDKIAAEPELLYEAFVKRGGYFFFCGPAGRVPQDIRAAI
ETAMSEGGKEDGFTREMASKYIDEMRKTSRYIVEAWS*

>jgi|ChlNC64A_1|24040|e_gw1.12.53.1
MATIHINGHTVSVPEGTSILTAATQLGIHIPTLCTHPRLPTTPGTCRLCLVEVAGGALKPACATPVCHGLEVTTDSPQVKDSI
RGVLALLKANHPADCMTCDVSGRCEFQARPGFWGGAWAGQRMACPRQRPMLPMLGCRCLKCGRCVTACGLVQEMDVLGMKGRS
RERHPAVLTEAMDLSKCISCGQCAVMCPVGAITERAEWREVEDQLDAKRKAGRGAGRAGLMVCVTAPAVRVAIGEELGLAPGT
ITTGQMVAAQRQLGFDYVFDVNFGADLTIMEEGTELLQRLRHAWGLDLPAEGSGGAGAGPLPMFTSCCPGWVTACEKSFPELL
PHLSTCKSPQQMMGAVVKSHFAAKLGKRAQDICLVSVMPCTAKKYEAERGEMVREGEGPDVDYVITTREFGRLLRERHIPLAS
LPESAFDNPLGEGSGAGVIFGNTGGVMEAALRTAYELAAGQPLPKLEEEAIRGLRGIKQATVTLPPTAPAGMASRQLRVAVAS
GIGQARHLLERMHTGHSPHFDFVEVMACPGGCVGGGGQPKSADPLVLLKRMGAVYSIDERSAIRKSHENPSIQKLYKACAEFL
GEPGGSLSHQLLHTTYINRSTASQPTYTAFQRMDEPCNPKLQQAAAAGRGSSSSALPGTSA*

>jgi|Nanoce1779_2|558506|estExt_Genewise1Plus.C_190151

MLGPFLFAWARIGPTAASAAALRRLPKPRTFAAASFSTTPPAIPTTSPTATFPAEEQLVNVEINGRSIAVPAYATILEAARAA
GAFVPTLCAHPSIPACATCRICLVELETGQGTKKVVGPASPTDARRVEKLVPMSKLVPACITQVKEGQRIHTHSPDVLENVRF
ILQLMRAKHPNTCQTCDADGNCTFQDLLYRYQVEDIAPTEIRKNFEHHHEWDGGASIGTPTDSQTPAPSKDTPYGAITFDPDK
CVKCGRCVAVCQKIQGVGAIGFVGRGADMHLSTMQGLPFDQTKCIECGQCVAVCPTGAMRERHDLFHVIDALQNKEVVKVAAV
APSVRVAISEEFDLPPGRISMGQIVVGLKQLGFDYVFDTNFAADLTIMEEGTEFLSRLKKEPGAGPLPMFTSCCPGWINLVEK
NYPYLIPHLSSCKSPQGMMGAAVKNVWAPRVGRDPADVVNVSIMPCTAKKGEAHRPADRAATYSAKLDTTVPNNDYVLTTREI
GRLFRLHKISLPALESQAPDDPLGESTGAAVLFGATGGVMEAALRTAYELASGGQPLPKIEMSDIRGLQGVKSARVCIPNPDG
KGPGAVVRVGVVHGTAETRALLERMKSGDESAQFDFVEVMACRGGCIGGGGQSKSDDPFVLQKRIAHVYNLDVESAIRKSHEN
PSIKKLYQEALGEPGGHLSHQLLHTQYTDRSQDTK*

>jgi|Vitbras1|16169|Vbra_650.t1
MLTNRSLQTLASAPLRLPAGSLALALPPLRAISTATVAAKAHDRTHQQSVAASAARRTPLFVSNSRVLGKGLFFHRPPLVTAT
STLPSYAPWTLTWRLPYAAVAAAQTQPKQQEIHVDVGEVARLRDECRAVNPECGYKPPLAFDPTKCLECWACEKACPVGDKFL
FNQSTCLLCGKCAQVCPTGAISSPPMPIRETLSKLKEEGKVTIAQVAPAIRSVLTEEQICYGLHDLGFDMVFDTNMAADICIM
EEAAELVTRITAPSQESPLPMFTSCCPAWINMVEKHYPHLIPHLSTCKSPQAMLGALVKHHWASQKGVSADDIFMVSVMPCIA
KKDEVQRVQLEHAVDAVITTKELMDQGSFDLEAAKNNTQKLHFDQPFAEHSGGAVIFGASGGVLEAAARTAYKMVTGNEPPHA
SLQPARQQGDVKELVLPVKEGVEIKVAIVNGMDHLSEVLPHMDKYHMIEVMNCEGGCVGGAGNPRALDLKEIQKRQQELYKRD
EKATYFASHETPAVERLYKGELKDEHLRHHLFHTHYNHRFEKDQWHLPATKLIHVKPATTPVSPPAMEHCKTLIPIGFDEIHV
KDRVRVTPEGYKPAQYVFKFTVPSGAAGGPSTYRTGDHFGLVPCNPPELVDRLLMWLGKDPMRAHEELVEVTSEDPLRAFPGM
PMTLHRLLSQFVDLNGPVPYYLRRLVSPDTGFVPPEAVAHKNAAEAIMSLPKGKYSFSEIVSACDIIHPRFYSIASSDTRCPD
EIELLVVDVPGGLCTGHICRQPLEYIQTTPIAGKLRKGVFNYPMDGNTPMVCVTVGSGFAPVRAFLQERKDRGLSGDFVLVHG
CRHASKDFVCQEDIQDGGVSASFYAFSRDDPKKKVYIHDKIAENGAAVWELLQRPDCQFFYCGSSGKILNQMETALIKCSEGI
CDGGSIIRQMKGEHRYHLEAY

>jgi|Thaps3|264905|thaps1_ua_kg.chr_23000023
IANALVSLRVDGRPVKVAEGATLLDAINTSGSHVPTLCYHPEFQPKAVCRMCLVNVKEANATATAGKLLPACRTKVEEGQEVT
TNSEDIKAFRRRDLQFLLNRHPNDCMRCEAAGNCKLQSLVQEECVEDMWPTKTSRGSDEHPHLLLDHTSPSIWRDMSKCIECG
LCVDACSAQKINAIGFAERGSGMLPITAFDKPLSETGCISCGQCILRCPVGALIERPDWHRVLDVLDDRKRTTIVQTAPATRV
AIGEEFGLEPGSVSTGRMINALRELGFDYVTDTNFSADLTIMEEAHELLQRLQGKREGALPLFTSCCPGWINYVEINRPDLIP
HLSTTKSPQQMHGAIARNGPMAKQIAAQTSESEEPYIVSIMPCTAKKDESVRPGNRGDIDAVLTTRELAKLIRHRDIPFASLS
NDGEYDSPMGESSGAGAIFGASGGVLEAALRTAADTLGLDGKNVDTLQHEQLRGVDRGIKVASIKGVGSVAAVSSIGSAIELL
NTDHWKKFLMIEVMACPGGCLGGGGEPKSDDKDILKRRMAGIYSIDKNAPIRKSHENKEVQQLYKDFLSFPLSEISERLLHTS
YAPR

>jgi|Tetstr1|462877|TSEL_007826.t1
MTFSGTVKLSGLDDHITPSQACVVALDGTKIDPTDMSEEVQIHSREDKPAPAMKEGADGKPVTISLQDCLACSGCITTAETVL
LQQQSVDEFRSRLAEPGTVTIASVSPQTRASLAEYYGLSAKEAGARLAGYLKSLGVQYVLDLSSARDVSLMEAAAAEFCQRFRF
ASSNAAGQMEIDGTEAQSATAGPLPMLASACPGWVCYAEKTHGEYVLPYISTSKSPQAVMGTLLKRQVAACHGMDPAKAFHCS
FQPCFDKKLEASREDFETEAKVKETDCVLSTLEVVEMLNSQGLDLKDCPAGSVDSLLALIGGQPAAGAEDDGALYGWPGALEV
VLTVNDEAVLRFATAYGFRNIQTLMRKIKRKQCEYQYVEIMACPSGCLNGGGQMRPKEGQSVQELIQKLDETYHHQDVRPRWP
ADNPLVRYVYTSPNLGSCYGPQARNLLHTQYHQREKSVSTAIVDW*

>jgi|Chabra1|335382|rna-CBR_g26339
MLRQLWQRGRGDRTGARCASWAIRRLTTAAEGTMRSVSAHEEAFSSRAGSAGGELRRKMCTAAAIPQNARQLWTAGCACGGSS
SRAAVSGGSDMGRNRQQQPRALLHSESAAAAAAVEEAKELVGIKVDGVEYRVPKGMTLLQACRDLGIYIPAVCYHPALKPVGT
CRLCLVDTGGWRMEPACTKEVAEGLTVRTHTDEVFDTVKGNLALMRLKHPNACSTCSVNSRCEFQDLLLRYGVEDPPMLEKQK
RVDHHVEEAGIAHEKDESSPALSMDFDKCILCLRCVRACNELQGMNILGATARGDHETVAPLYGLPLGETSCLSCGQCAAVCP
VGSITEKSHVRQVEELLQMREAESLPPRTEKDWDQEMPKTRLLWGPRQMVSASQEVRASSSSSSSSGGGGGRGYATTSSSRSS
SSSAGSEAAAAAEATAAAAVAPRTGKRPVMVAQTAPAVRVTIAEAFGMEPGSISTGHMVAALRKLGFDYVFDTNFSADLTIME
EATELLGRVKSGGPFFPMFTSCCPGWINMVEKTYPELIPNLSSCKSPQQMLGAVVKSHFAASVGRHPSEVKFVSVMPCVAKKDE
AARKEMHNEDLGGPDVDYVLTTRELASLIERARPRINFASLKEAEFDSPLGQSSGAALLFGVTGGVMEAALRTAVAVTAPADA
PPMPKLDFTEVRGLDGVKEANIDVNGTELKVAIAHGGVHLRSLVEDILAGKRTYHFVEMMACRGGCIGGAGNPKDSVDEKLLQ
HRAQAIYKGDEASAIRMSHENKEIAQLYEQFFGEPNSHKAHKLLHTHYTPRKVHKSGEIDRFSEYSPEGPSELCKF*

HYDF orthologs

>jgi|Nanoce1779_2|132847|CE132846_2156
MVLGLIKLPGPYTSKLLRPSRPIYDTSLDRASSLSPFFASPCASLCSCSTNSSSSSSSFSTTSSSYSSGLHRLLLGLVGEMNA
GKSTVLNALTQQATSIVDATPGTTSDIKTAIMEIHGLGPIKLLDTAGVDDTGRLGDKKRNKTWEAMREVDAALVVIEPFNKAK
VATQGMDEEEGRAGRRRVAENGCGGGEARITAISNSSSRSSSSRISTITICGRDSRKSSCRENASGAGCLYSLLCLPPSSHFP
CSKHGQATPSLHAHLQY*

>jgi|Cyapar1|10289|g9962.t1
MASPVLARALSRATAPLVFGAASVVPRPGMLQVARLVGSALLSRRFLVSSATAFAAHQAPSAHAQAAPKPPGGPLASTQTPRV
NIGIFGSMNVGKSSLMNLITQSNTSIVDSTPGTTADVKAALMEMHALGPAKIFDTAGLDERGLLGEKKREKSVNAAKESDVVV
LVVNPRVGHSEYDMEILDVAKRREKKILVLLNEFGGDPKWSGPGYTERAEYLGLPALRVKLNQPGNMQAVVDFIESEYQRGAV
KSPPLIPPRYMGPDKTVFMVVPMDAETPSGRLLRPQARVQEFCLSHYTTTVAFRLDLAAGRSKDAAAVAKERARFEAAVRAAD
PALVITDSQAMDLVHPWLDPAKFPNADLTTFSIVMINDLSGGRIREFADGLQQLARLRQGDKVLIAEACNHNRLSVEVCADIS

TCQLPRKIESMYGKDVVKVDHAFGREFPASELKDYSMVIHCGGCMVDKQKIVARIDDCKELGIPVTNFGVMLSYMASPAAAQR
VLRPLAH*

>jgi|Chabra1|336334|rna-CBR_g28613, extended N-terminal region
MSDREEQQPDDRILRSARPPIAHLALGPEGDRYLFRQGRERLQQQRRARAAEASRTLVSEAASSEAMATSAQQTSQAGSSGSV
GSTVAQTQSQSTGGMASQPLVLTFDEIAIQEATLKEAQLQHALGQIKAEKERMIRRRVRMLRREADVSELEEMDLTHATDDVR
IIRSVLLNVVEMQDQQTSILQGIPQSEANINMHNFPSFSKKALDLEAKIGHGQPPTTDGRRKTLPPNWKAKERIMFVDNDGST
IELDEHFQEGVGSEAGSAEASKGGVVVAVAQKAKLGGGYVPSLSLLQAPVKMSPEIEGVVAKYPDLFEEPIGVVEREVVHAIE
IIPQSSIPKGRIYRMSPGELDELRRQLKELLEKGWIRPSVSPYGSPVLLVPKKEGTLRMCIDYRGLNAITVKNREPLPRIDDL
LDRVQGCRYFSKIDMKSGDQHKTAFQTRYGLYEFVVMPFGLCNAPGTFQHAMNRIFHDYLDKFVIVYLDNILIFSKTVEEHIA
HLDKVLSLLRQHKFKINGEKCEFGRTRVLYLGHEISAEGLKPDNAKAVSIRDWPRPRSVTEMRSFLGMTGYYRTFVKNYSIVA
APLTDLTRLDTPWEWTVTTDASQYGIGVVLAKQEGPKLRPVEYMSKRMSSQKLAKSTYEKELYAVYKALTHWRHYLLGRFFIL
RTDHQTLKWMRTQSVLSDALKRWIEVIEQYDFDPQYLKGEYNKVVDALSRRPDFSGALITEFDLTDNVTQSLVEAYREDQFMS
EIIRRLQAKVKKTSAEFELVNVLLFLEKVGNKRLCVPNSESLRSLFLGECHDATGNFGYKKTAANLLQRFWWPTMMRDAQLYV
ETCQVCQRDGPRTQAPLGLLKPLPIPERPGESLSMDFMDTLITSKSGMRYIYVIVDRFSKFARLVAMSATANTEYVIKMFKEN
WVRDFGLSKSIQHEGPKLLVTDSQAMDIVHPWTLDRSTGKPIIDITTFSIALIQRQTDGRLGLLVKGLEAYKSLRPGDRVLVA
EACNHNRITELCNDIATVQIPQQISKENPKGGVIIDHAFGREFPDLDGVDGLRQYQLAIHCGGCMVDRQKILARITDLEEAGV
PVTNFGLVLSYAAAPEAFARVLEPYDVEIPKSVFLQSHAQCAA*

HYDEF orthologs

>jgi|Chlre5_6|15537|Cre06.g296750.t1.2
MAHSLSAHSRQAGDRKLGAGAASSRPSCPSRRIVRVAAHASASKATPDVPVDDLPPAHARAAVAAANRRARAMASAEAAAETL
GDFLGLGKGGLSPGATANLDREQVLGVLEAVWRRGDLNLERALYSHANAVTNKYCGGGVYYRGLVEFSNICQNDCSYCGIRNN
QKEVWRYTMPVEEVVEVAKWALENGIRNIMLQGGELKTEQRLAYLEACVRAIREETTQLDLEMRARAASTTTAEAAASAQADA
EAKRGEPELGVVVSLSVGELPMEQYERLFRAGARRYLIRIETSNPDLYAALHPEPMSWHARVECLRNLKKAGYMLGTGVMVGL
PGQTLHDLAGDVMFFRDIKADMIGMGPFITQPGTPATDKWTALYPNANKNSHMKSMFDLTTAMNALVRITMGNVNISATTALQ
AIIPTGREIALERGANVVMPILTPTQYRESYQLYEGKPCITDTAVQCRRCLDMRLHSVGKTSAAGVWGDPASFLHPIVGVPVP
HDLSSPALAAAASADFHEVGAGPWNPIRLERLVEVPDRYPDPDNHGRKKAGAGKGGKAHDSHDDGDHDDHHHHHGAAPAGAAA
GKGTGAAAIGGGAGASRQRVAGAAAASARLCAGARRAGRVVASPLRPAAACRGVAVKAAAAAAGEDAGAGTSGVGSNIVTSPG
IASTTAHGVPRINIGVFGVMNAGKSTLVNALAQQEACIVDSTPGTTADVKTVLLELHALGPAKLLDTAGLDEVGGLGDKKRRK
ALNTLKECDVAVLVVDTDTAAAAIKSGRLAEALEWESKVMEQAHKYNVSPVLLLNVKSRGLPEAQAASMLEAVAGMLDPSKQI
PRMSLDLASTPLHERSTITSAFVKEGAVRSSRYGAPLPGCLPRWSLGRNARLLMVIPMDAETPGGRLLRPQAQVMEEAIRHWA
TVLSVRLDLDAARGKLGPEACEMERQRFDGVIAMMERNDGPTLVVTDSQAIDVVHPWTLDRSSGRPLVPITTFSIAMAYQQNG
GRLDPFVEGLEALETLQDGDRVLISEACNHNRITSACNDIGMVQIPNKLEAALGGKKLQIEHAFGREFPELESGGMDGLKLAI
HCGGCMIDAQKMQQRMKDLHEAGVPVTNYGVFFSWAAWPDALRRALEPWGVEPPVGTPATPAAAPATAASGV*

>jgi|Vitbras1|16622|Vbra_296.t1
MLARPLFWALPSSVEPMRALSILPSRFLSTLASQASSSVPRASTSPFLAIPKAAQINTNNDGAPLYAPMMDAHAAAGVLGDVL
KRGIGLGEVVCLGQNEIHGLMEMCWKEGSRDERVREALFAHANEVTRRFFGNKVFYRGLIEFSNVCQNDCLYCGIRKNQPNVE
RYTISKKQLLSIALWAHDNGYGSVMLQSGELNHPARMKYLTDVITAIRRETVLTNLEKEKRHRRHSNQAEDPAIDELIEEVRS
ESFIGSDPRFKDKGIGIALSVGELSEADYRRLHEAGGHRYLLRIESSNPELYSKIHPPTQPFAKRLACLRLLKKVGFQVGSGV
MIGIPGQTLEDLARDIVFFRDEDIDMVGMGPYITQEDTPVADMWQAQYGHVDKKEHMAEMFDLTTVLISICRITMGNINIAAT
TALQAINANGREVALRRGANVLMPILTPTQFRENYQLYEGKPCITDTAEQCKECLNFRLKSIGKEAAKAVWGDPPHFYQHVRS
HHTLARRSFSSKPSPAAPKSPGASELPRINIGFFGAMNAGKSTLMNKLTRSNLSIVDATPGTTADTKITLMELHDIGPTKLFD
TAGIDERGELGEKKRRKTISVLKESDIALLVVDPLQKLSPTEPTLASPDALQWERYLIEMGEKRHKEVSIVFNIKEQEGATQG
WTRRQIEDGVVRLLEMLDPSSMHRVLCLDLGAPEASDRLVKFIQSSPAHGSMYHDDLPCVPESYLTADSVFFLNIPMDAETPA
GRLLRPQAMVQESIIRHYGTTVAYRMDLAKARSSDPAEVQEEKSRFLKMLNMVLQQTAGPKLLITDSQAMDIVHPWTLDPVTN
RPLIDITTFSIALLHRQSGGQLPTFVEGLERFKHIKQGDRVLVAEGCNHNRITQLCNDIATVQLPGHIRAQGGASIAVDHAFG
REFPDIEDQSDKGLGAYSLVIHCGGCYIDKQKVQARVDDLREMGVPVTNFGLLLSYVAAPEAVDRALQPWGLTSV

>jgi|ChlNC64A_1|142788|IGS.gm_5_00117
MLGSSRALQAAAAAAVSMQKALPWENAVTAEEAVKVLGSFWGTTFAPGDVLDLSVDQMEGLLSATSTGAGSKVLDNALFAHAE
SVTGRFFGRDVYYRGIVEFSNVCENDCGYCGIRKHQRRARRYTMPRDEVVEVADWAFRHRLGTLMLQSGELNTPQRMKYLTDV
VAAVKERTRALDAEQRLVDPEALPKDAAGVGLCVALSVGELPREQYQQLKDAGADRYLLRIESSNPQLYASIHPPAQARASGP
ARFHKWENRTRCLRDLKDIGFMIGTGVMVGLPGQTLRDLAGDIMFFRDLGADMIGMGPYITEAGTPVADMWEQQFGHVDKKKH
MQDMLTLTTRMNALARITLGNVNIAATTALQAIDAVGREVALRRGANVLMPILTPTKYREHYTLYEGKPCITDTAEECQKCLN
ARLAMVDKKLKPGVWGDPPSFRDSVHPVPMQAQAAAAASATGGSRGLRTWAPVSAAAARDGGLHTCSATGQQAAAAPAPGRGA
VLGMGGGPAKGSDVPRTNIGIFGCMNAGKSSLMNRVTRSETSIVDSTPGTTADTKVVLMELHDVGPAKLFDTAGIDEEGALGE
KKRRKVLSVIKETDVAVVVVDVGRFLDTPADRLPAALAWERLLLDKAAAAGSSPVLVYNTKLGGAGAGAGPEAAAAAVDRLQA
ALNPTGAIMSRQLQLSREEASDALAEFLQEAAAAAKQHDAPRSLPDEFLSEDAMVFLNIPMDAETPSMRLLRPQALVQEEAIR
HFASTIAYRMNLAHARSSDPELVQRERARFLRALQPVLAHQGPKIIITDSQARRAGSGRPCGTAVDILHPWTLDPATGDELVP
FTTFSIAMIHRQSRGQLPLFVRGLERFKSLRAGDKVLVAEACNHNRITDICNDIGMVQIPEKIEAQGGRGVVVDHAFGREFPE
LDAEEHCVNAVLQEAGVPVTNYGLLLSYAHSPAALERAMKPWGLRM*

>jgi|Tetstr1|460551|TSEL_005809.t1
MSEDEILKVAHSAFAHKMGTIMLQSGELRTPQRLRFVNRVVRRIREETIASELSATGRLPGVPPSQMCKVVPVETADVGLCVA
LSIGELSLPRLQELRESGAARYLLRIESSNPDLFTRIHPPAQTFEQRLRCLQDIKAAGLQLGTGTPPPPPPARPRMALFPGVL

SASVMIGLPGQTMSDLASDLCFFRDIGADMIGMGPYIPEPNTWVADDFHRRHPDLPALQPYLEEMMELTTTMNALARITMGNI
NIAATTALQAINPLGRELALARGANVVMPIMTPTEERANYQLYPGKPCVSHTADGCASCLKMRIASVGKNLQTDGAWADPPHY
RNPVAHSFAAAPLAGRTPATGGEQRRSAHRAAAASALPEAGPPPAGPSKGSDVPRTNIGVFGCMNAGKSTLVNALTRQETSIV
DSTPGTTADTKIALMELHEIGPVKIFDTAGIDEAGELGSKKARKTLAALKETDVSVVVVDAAAVAAAGEGSLAWESDLVAMAR
KYGSMPLLVVNARGGRGSEALAAAAKAAVSPTEDIIALQADLATEAGRSAITGFLQEEILKAKRGAAGVPSLPPQFLSDRAMV
FLNIPMDAETPSMRLLRPQARPRPTGAGPPTPYPSMPCLGRGSCGAGRVASRPAALVQEEAIRHFATTVAYRMNLNMARSADP
AEVAAERRRFMRALRPVLEHDGPKIIVTDSQAVDILHPWTLSPDGAPLVPFTTFSIAMVNRMSGGQLRTFVDGVAALETLKEG
DAVLVAEACNHNRITDQCNDIGMVQIPQALARLTGGRGPVVEHAFGREYPEVQEGAGAGLGRFKLAIHCGACMIDRQKMRARL
MDMKEAGVPVINYGLFLSYMQSKQALARAIEPWNL*

>jgi|Tetstr1|436239|TSEL_025083.t1
MEALITRVGSSLLTSLLRPALVAASARLAGPALRSAASLRSHSTDGEMPTFDAAHTRRVNQMLAAKDTLIPEEAAAVLTSHWG
APVLPGQVLSDLTASQLAATLETAHPVGTGNINVDDGHLLRSALYMHAESVSTRFFGDKVFQRGVMIGLPGQTMSDLASDLCF
FREIGADMIGMGPYIPEPNTWVADDFHRRHPDLPALQPYLEEMMELTTTMNALARITMGNINIAATTALQAINPLGRELALAR
GANVVMPIMTPTEERANYQLYPGKPCVSHTADGCASCLKMRIASVGKTLQTDGAWADPPHYRNPVAHSFAAAPLAGRTPATGG
EQRRSAHRAAAASALPEAGPPPAGPSKGSDVPRTNIGVFGCMNAGKSTLVNALTRQETSIVDSTPGTTADTKIALMELHEIGP
VKIFDTAGINEAGELGSKKARKTLAALKETDVSVVVVDAAAVAAAGEGSLAWESDLVAMARKYGSMPLLVVNARGGRGSEALA
AAAKAAVSPTEDIIALQADLATEAGCSAITGFLQEEILKAKRGAAGVPSLPPQFLSDRAMVFLNIPMDAETPSMRLLRPQARP
RPTGAGPPTPYPSMPCLGRGSCGAGRVASRPAALVQEEAIRHFATTVAYRMNLNMARSADPAEVAAERRRFMRALRPVLEHDG
PKIIVTDSQAVDILHPWTLSPDGAPLVPFTTFSIAMVNRMSGGQLQTFVDGVAALETLKEGDAVLVAEACNHNRITDQCNDIG
MVQIPQALARLTGGRGPVVEHAFGREYPEVQEGAGAGLGRFKLAIHCGACMIDRQKMRARLMDMKEAGVPVINYGLFLSYMQS
KQALARAIEPWNL*

HYDE orthologs

>jgi|Cyapar1|17193|g16914.t1
MSFDPKLVGAKPRNFKPIEERILPFARFLGKPDLKPGESLDLSRDELTALLTTTDAALEASLYAHANSVTERVFGNKVYFRGI
VEFSNVCQKNCEYCGIRKSMPAAQLRRYTMTAEEIVDCAEFCYRQGYGTLMLQSGELDTERRLNFVIDVIKQIRERCRELERE
HLGLPADTPLDKLKGLAVALSIGELSADKYQRLFDAGAVRYLLRIETSNPALYAKLHPADHKWETRHRCLLDLKRIGFQIASG
VMVGIPGQTYQDLANDLLFLKELDVDMIGMGPYIYQENTPVGEQWKKEFGSRPKDDHNAWLLSTASRMYSLARILIPDCNITA
TTALQAIHPSGRELGLNRGCNVLMPILTPLKYRENYQLYQGKPCVDEGATECRKCLVSRVEWSGKDLALGIWGDPPHFFRRAG
LELPSETPILPKPALTDKKAHH*

>jgi|Tetstr1|460551|TSEL_005809.t1
MSEDEILKVAHSAFAHKMGTIMLQSGELRTPQRLRFVNRVVRRIREETIASELSATGRLPGVPPSQMCKVVPVETADVGLCVA
LSIGELSLPRLQELRESGAARYLLRIESSNPDLFTRIHPPAQTFEQRLRCLQDIKAAGLQLGTGTPPPPPPARPRMALFPGVL
SASVMIGLPGQTMSDLASDLCFFRDIGADMIGMGPYIPEPNTWVADDFHRRHPDLPALQPYLEEMMELTTTMNALARITMGNI
NIAATTALQAINPLGRELALARGANVVMPIMTPTEERANYQLYPGKPCVSHTADGCASCLKMRIASVGKNLQTDGAWADPPHY
RNPVAHSFAAAPLAGRTPATGGEQRRSAHRAAAASALPEAGPPPAGPSKGSDVPRTNIGVFGCMNAGKSTLVNALTRQETSIV
DSTPGTTADTKIALMELHEIGPVKIFDTAGIDEAGELGSKKARKTLAALKETDVSVVVVDAAAVAAAGEGSLAWESDLVAMAR
KYGSMPLLVVNARGGRGSEALAAAAKAAVSPTEDIIALQADLATEAGRSAITGFLQEEILKAKRGAAGVPSLPPQFLSDRAMV
FLNIPMDAETPSMRLLRPQARPRPTGAGPPTPYPSMPCLGRGSCGAGRVASRPAALVQEEAIRHFATTVAYRMNLNMARSADP
AEVAAERRRFMRALRPVLEHDGPKIIVTDSQAVDILHPWTLSPDGAPLVPFTTFSIAMVNRMSGGQLRTFVDGVAALETLKEG
DAVLVAEACNHNRITDQCNDIGMVQIPQALARLTGGRGPVVEHAFGREYPEVQEGAGAGLGRFKLAIHCGACMIDRQKMRARL
MDMKEAGVPVINYGLFLSYMQSKQALARAIEPWNL*

>jgi|Tetstr1|436239|TSEL_025083.t1
MEALITRVGSSLLTSLLRPALVAASARLAGPALRSAASLRSHSTDGEMPTFDAAHTRRVNQMLAAKDTLIPEEAAAVLTSHWG
APVLPGQVLSDLTASQLAATLETAHPVGTGNINVDDGHLLRSALYMHAESVSTRFFGDKVFQRGVMIGLPGQTMSDLASDLCF
FREIGADMIGMGPYIPEPNTWVADDFHRRHPDLPALQPYLEEMMELTTTMNALARITMGNINIAATTALQAINPLGRELALAR
GANVVMPIMTPTEERANYQLYPGKPCVSHTADGCASCLKMRIASVGKTLQTDGAWADPPHYRNPVAHSFAAAPLAGRTPATGG
EQRRSAHRAAAASALPEAGPPPAGPSKGSDVPRTNIGVFGCMNAGKSTLVNALTRQETSIVDSTPGTTADTKIALMELHEIGP
VKIFDTAGINEAGELGSKKARKTLAALKETDVSVVVVDAAAVAAAGEGSLAWESDLVAMARKYGSMPLLVVNARGGRGSEALA
AAAKAAVSPTEDIIALQADLATEAGCSAITGFLQEEILKAKRGAAGVPSLPPQFLSDRAMVFLNIPMDAETPSMRLLRPQARP
RPTGAGPPTPYPSMPCLGRGSCGAGRVASRPAALVQEEAIRHFATTVAYRMNLNMARSADPAEVAAERRRFMRALRPVLEHDG
PKIIVTDSQAVDILHPWTLSPDGAPLVPFTTFSIAMVNRMSGGQLQTFVDGVAALETLKEGDAVLVAEACNHNRITDQCNDIG
MVQIPQALARLTGGRGPVVEHAFGREYPEVQEGAGAGLGRFKLAIHCGACMIDRQKMRARLMDMKEAGVPVINYGLFLSYMQS
KQALARAIEPWNL*

>jgi|Chabra1|336355|rna-CBR_g28634, bad annotation?
MCINYRGLNAITVKNAETLPRIDNLLDRVQSCRHFSMIDLKSGYHQIEVLEKLKEANFKINAKKCEWAKTEVLYLGHVLDEDG
IKPEDSKIAAIRAWPTPRTLTELRSFLGLANYYRKFARNFSIIVIPLRRLLKKEAIWQWDKDCTFALKKLKRALIEYLVLKVA
DPSLSFVVTTDASRYGIGAVLQQDDDNGYKPVEFMSTRMPSEKDCYYCGIRKHKEGVHRYTIPKEDVVSVAMWAFDHKYGTLM
LQAGQLNSPQRMEYLVDLIREIRRKTIAKDLEQRRARGVNTDRLKATELGLCVALSVGELSEETYKRLYDAGASRYLLRIETS
NPELFSDLHPGDQMFEKRLKCLRTAKRAGFQIGTGVMIGLPGQTLRDLANDIVFFRQEEADMVSAKGWVRCKGWLEPRKHNGG
HSVPHFRPLDTGLQTIFWDVLERGARVQHGNRSRRAHSFPAMPHVLDNMAETRKGTITAPYTAKEQEKMAALVKENRERKERE
KQAKLKAIAEEQATKMKEIGEEMEKKKKAAEKEVAAEEEKERMRTESREGSSGTKEDKDAEMEKKISEWVANLSLGEDEEAQM
YVPEEEKEAFARALAVIGDPLERQEAEEEKRLEWKLRMKREKKRREEANWLTAEVEKVLEKFREANFKINSKKCEWAKTQVLY

LGHVLDGDGIKPEDSKIAAIRDWPTPRTLTELPSFVGLANYYRKFVRNLPTIAAPLRRLLKKEAIWQWDKDCTSAVKKLKRAL
IEYPVLKVADPSLPFVVTTDASQYGIGAVLQQDDDNGYRPVEFMSARMPCEKTHSGKDTSPYSEEQQEKMVALVRENKERKEL
EKQAKLKAIAEEHAAKMKRLEEEMKRIQQEEEEVAEAEFVIVYLDDILILSKTVEEHVVHLDKLLSLLRQHKCKINGEKREFG
RTRVVYLGYEIFAEGLKPDDAKVANIRDWPRPQSVIEMRSFLGMTSYCRNFMKNYSIVATPLIDLTRLDTPWEWTETCEAAFR
HLKHALRHYEVLKLPDPDKPFIVTTDASQYGIGAVLAQQEGKKLRPIEYMSKKMPSQKLAKSTYEKELFAVYKALTHWRHYLL
GRFFILRTDHQTLRWMRTRAVLSDTLKHWIEVVEQYDFEPEYIKGEYNKVVDALSRRPDFSGALITEFNLADNVTQPLVEAYR
EDPFMAEIIRRLEAKDKGTSPEFELVCQSDKPRTQAPL
GLLKPLPITERPGESLSMDFMDTLVTNKSGMRYIYVIVDRFSKFARLVAMSATTNTEYVIKMFKENRVRDFGLPKFIVSDRDV
QFTSELWKAAAAEQGMQLQMTSGNHPEANGQAEQMNRAVQHLLRHYIKPNQVDWDKKLTLIASLYNNVVHSATGVSPNSLLPT
FTPVLPLDFLLPDNQPTAAPGT*

>jgi|Nanoce1779_2|632220|estExt_fgenesh1_pg.C_190091
MLLLKKNVAVTGSTVAAFFRKRPRLPVAPLERLASSSPSPPTVTFHRTRPPAATAQCQHDAQASFAPLAHFLGVEKLEPGRII
DLSKEQLMELLTTQDGALVQALYSLAEAATTAYFGSKVYARGIVEFSNVCTKDCLYCGIRKHMKVHRYTMTKQEIVTCAEFAY
TQGMGTLMLQSGELPTPERINFMLDVVRAVRLRTVEMDLEVHPSCSSSLPSASLSVTPNNLAASSQRGLAVALSLGELPSETY
KAFYEAGASRYLLRIESSNPVLYHKLHPPSHSFESRRQCLNELKRIGFQVGTGVMVGVPYQDVEDMAGDLLFFKEFDPDMIGL
GPYIVQAKTPLGQVYLAERQHHSAEELEKERIDLLERTTRMYALARLILGNVNIAATTALQALDPQGRELALRRGANVVMPII
TPQRLREEYQIYENKPGIDQDALESTACLDLRVQLAGKELLLDGRWHDPPHYLTRKVR*

HYDG orthologs

>jgi|Chlre5_6|15536|Cre06.g296700.t1.2
MSVPLQCNAGRLLAGQRPCGVRARLNRRVCVPVTAHGKASATREYAGDFLPGTTISHAWSVERETHHRYRNPAEWINEAAIHK
ALETSKADAQDAGRVREILAKAKEKAFVTEHAPVNAESKSEFVQGLTLEECATLINVDSNNVELMNEIFDTALAIKERIYGNR
VVLFAPLYIANHCMNTCTYCAFRSANKGMERSILTDDDLREEVAALQRQGHRRILALTGEHPKYTFDNFLHAVNVIASVKTEP
EGSIRRINVEIPPLSVSDMRRLKNTDSVGTFVLFQETYHRDTFKVMHPSGPKSDFDFRVLTQDRAMRAGLDDVGIGALFGLYD
YRYEVCAMLMHSEHLEREYNAGPHTISVPRMRPADGSELSIAPPYPVNDADFMKLVAVLRIAVPYTGMILSTRESPEMRSALL
KCGMSQMSAGSRTDVGAYHKDHTLSTEANLSKLAGQFTLQDERPTNEIVKWLMEEGYVPSWCTACYRQGRTGEDFMNICKAGD
IHDFCHPNSLLTLQEYLMDYADPDLRKKGEQVIAREMGPDASEPLSAQSRKRLERKMKQVLEGEHDVYL*

>jgi|ChlNC64A_1|30311|estExt_Genewise1.C_50128
MLSQAASSLGGLASGGLGPLLPAVAAVGARLLGRSGLLQQQRGLQWSVEKEPQSYKAVDDIIQDHVIVAALEKTQQAAKDPAR
VRDILTAAKERSFLTNHKPGPSEYVQGLTYEECATLLNVDVGNEQIMSDIFDTAFAIKQRIYGNRIVLFAPLYIANYCVNNCR
YCAFRQGNKSLERSALTDKQLREEVAALQQQGHRRLLVLTGEHPKYTFDSFLKAIDTISSVRTEPCGNIRRINVEIPSLSLSD
MRRLKATDKIGTYTLFQETYHRPTFKHMHIAGPKSDYDNRVMTHDRAMRAGLDDVGLGTLFGLYDYKYEVLATLMHANHLERE
YGAGPHTISVPRMRPADGSDVSLAPPYAVDDANFKKLVAILRIAVPYTGMILSTRESPEMRTELLRVGMSQMSAGSKTDVGAY
HRDDSKATEDNLADLNGQFTLADHRPVQDIVVDLMKEGYVPSWCTACYRKGRTGEHFMKIAKAGNIHNFCHPNSLFTLQEYLN
DYGSEEAKQIGQDLIERERVVGLSDSAQNLTKRKLAKVNAGEHDVYI*

>jgi|Vitbras1|16623|Vbra_297.t1
MLSVVRHISAPSAALPALSCLSSRLASPCAARTQRFVRHFGLWSKELEKGHVYKDANEIINHDLIFKHLEATKGAAKDKNVIK
DILERAKERSFLKDGPATGGSEYVRGLELDECATLLNVNPDDTDIMEEIYNTAFDIKNRIYGNRIVLFAPLYLANYCVNGCAY
CAFRSSNKAIQRTKLSDQQVTEQVSILERQGHRRILALTGEHPAYTFEDFLHALKLITEVRTEPCGSIRRINVEIPSLSVSDM
KRLKAADCVGTYTLFQETYHKPTFHTMHPWGPKSHYENRVLTHDRAMRGGLDDVGIGVLFGLYDYRFECMAMLMHSNHLEEEY
RAGPHTISVPRMRPAMGSGVASDPPYPVDDAHFKKLVAILRIAVPYTGMILSTREAPEMRAHLLRVGMSQMSAGSSTEVGGYH
RHATQRTDESLGDLTMQALLPEKYGKAAAGDTAMDHEHTDPNLGGQFQLEDTRSSEEIVHDLLESGYVPSWCTACYRKGRTGE
HFMKIAKAGNIHNFCHPNSLLTLQEWLNDYASPEIKELGNKVIAREKEIDLSDSAKRLLDRKMEAVNKGQHDVYI

>jgi|Nanoce1779_2|598502|fgenesh1_kg.19_#_588_#_TRINITY_DN7538_c0_g1_i7
MLRLAQWCCRPRVSSSAAPSTTSPARLNLAIWPHQQRGLAQWSVETERHHKYEDPAKIIDEVAINRLLEETKEKAKDPAHVKA
ILVAAQDRARLQSPPPGHPNADPPLNEFVQGLTLEEAATLLNVDSADKEMMQLLYNAALNVKQQIYGNRIVLFAPVYLANWCV
NTCQYCAFRGANKQMQRSMLTMDELRQEVEALEAIGHKRLLLLTGEHPKYSFDRFLEAVQVVGTVKTPNPCGEIRRINVEIPS
LSVSDMKRLKATQYIGTYTLFQETYHRETFKKMHPGGPKSDYNHRLLTMDRAQLGGLDDVGIGALFGLADYRFEVMGLLMHAH
HLDRTYGTGPHTISIPRMQPALNAPAAEHIPMPVSDDDFKKLVAVIRCAVPYTGMILSTRESAATRSELLRLGISQISAGSRT
DVGAYHRDTTCDAANFISNTASSSSTAGRPAKTQLPLAAIAAATAVHEVEEEEDTDEYKRRRGQFSLQDHRPLDTVIKDLLHD
GFIPSFCTACYRKGRTGAAFMKIAKSGRIQDFCQPNALLSLQEYMEDYASPQTKAVGDEVIARESQMMGDNAKRAYERKRQQL
LRGKRDLYF*

>jgi|Cyapar1|17192|g16913.t1
MLSAATARAAALAGAAAPRIASSIAPALARTLPSLAKRLMSAKTAGLKGRGSLFSPEREPQHVFKPANEIIRAAEIDAALERA
KALAKDESVIRDILGRARDRALLKDHGMQPLGGGEYVQGLTVEEAAILLQVDSNNQELMQMLYDTAFAIKNLIYGNRIVLFAP
LYIANYCVNSCTYCSFRAPNTSMPRAVLTDEELRQEVEALQRMGHRRLLLLTGESPRYTFDQFLNALKIASEVRTDPCGSIRR
INVEIPSLSVSDFRRLKETNCVGTYTLFQESYHPETYARAHPAGPKSDYEWRVQTMDRAQTAHVDDVGVGVLYGLHDYKWETL
AMLQHAQHLDQTYGAGPHTVSIPRMQPAEGAPDAMNIPHPVNDEDFKKLVAVIRCAVPYTGMILSTRENPEMRRQLLRLGVSQ
MSAGSKTDVGSYHQNQVKPGAKPVAEHEHFTAGPEPSAQPATCTPKEAKDAAGQFTLQDHRSLDDVVGDLLELGFVPSWCTAC
YRKGRTGEAFMKIAKKGDIQALCHPNALLTLEEYLIDYASERTREIGKKVLEVETGHIPSERAKHAFERKLKKIDEGQRDLYF
*

>jgi|Tetstr1|436240|TSEL_025084.t1
MALCAGALSTVLRSAAAPLAVGALRVLGGCGARSMALWSKELEKGQTYKSADDIIQHDVIERELEATKSSAKDPRAVGAILEA
ARERSFLTGYNPAGGGSEYVQGLTYKECATLLNVDVANSPLMEQIYETAYNIKERIYGNRIVLFAPLYIANHCVNNCAYCAFR
ADNKNIARSILTDQQLREEVVALEKQGHRRLLVLTGEHPKYTFDQFLNALKIIAETRSEPCGQIRRCNVEIPSLSLSDMKRLK
ATNVVGTYTLFQETYHRPTFAEMHISGPKSKYNNRLLTHDRAMRAGLDDVGIGTLFGLYDYKYEVMAMLMHANHLEAEYGAGP
HTISVPRMRAAEGSDISVAPPYEVDDPNFKKLVAILRIAVPYTGMILSTRESPEMRRELLKVGMSQMSAGSRTDVGAYHRQDS
TETRENLGNMAGQFSLLDERPVNQVVLDLMEQGYVPSWCTACYRKGRTGEHFMKIAKAGNIHNFCHPNSLLTLQEYLNDYGTP
AEKAAGQKLIKDESERGFMSDQAVKLMNRKMKKVNAGEHDVYI*

>jgi|Chabra1|346080|rna-CBR_g46723
MGWGSALVRTAGRAVGRLPRGSLSLGLPAKCMSAATMMQTRGLMWSVEKEQGHTYKPVEEIIDHNRITLAMESTMEASKDSSR
IKEILEAARERSFLKDHTPTKSEYVQGLTVEEMATLLNVDPANEEIMNEIYDTAFAIKNRIYGNRIVLFAPLYIANYCVNQCT
YCSFRAPNKTIARTVLTDEQIRQEVEALEKQGHRRLLVLTGEHPKYTFDSFLHALHVVSEVRTEPCGNIRRINVEIPSLSVSD
MKRLKATDVVGTYTLFQETYHQPTFKVMHPAGPKSDYPHRLLTMDRAFRGGVDDVGLGVLFGLYDYRYECLAMVMHANHLEAE
YGAGPHTISVPRMRPAEGAETSVNPPYPVDDAHFRKLVAILRIAVPYTGMILSTRESPEMRAELLKVGMSQMSAGSRTDVGAY
HRQADDTTEQNLGNLAGQFTLSDHRPVAEIVEHLIRNNYVPSWCTACYRKGRTGEAFMKIAKAGNIHNFCHPNSLLTLQEYLQ
DYASEDLKKIGEELIAKESSTGLSDSAQRVLDRKMSKITDIQTLTIGYGKHPASLGKGARLVWIAQDQRIAAMFKEATEGSAS
GDNIC*

HCP orthologs

>jgi|Chlre5_6|18301|Cre09.g391650.t1.2
MLRAPVMSSAASRKVAAPAVAARAGCRRVGVMRVFAFQKAASCDNLHDKNALHERIQKSKELLDAESAMMCYQCEQTKSG
TGCTDIGVCGKTPEVSALQDLLIYSVKGLGSLAHVARTSPAKIEDAAVNTFINGAIFSTLTNVNFADDRFLEFVTDCRKL
HAQLAAKMAAAGVAVPAAETAHQPWFGSMPHPLAWNSDTHVALAGVGDMLEVASKTGVKERQHVLGETLAGLQELLMYGL
KGLCAYAHHAEALGHTDPAVYADVQAYLHFLCSPAAADVGQVLDACFRAGATNFRVMEMLSNAHTDTFGHPVPTPVTLNP
VPGKAILVTGHDMHDLHMLLEQTAGKGINVYTHGEMLPAHGYPGLKKYPHLVGHFGGAWYRQKIDFAAFPGAVAVTTNCV
LDPLTAYKDNIFTINETGLSGVPHIRPDATGHKDFTPIINRALQLPGFTPESVAKMEKKRDVTVGFGHKSVLSVAPQVIQ
AIQEKRLEHIFLVGGCDGSEPQRKYYSKLYQFMPTNTMVLTLGCGKFRIFDQDFGTLPGTDLPRLLDMGQCNDAYSALVV
ATELAKVFKTDVNSLPLSLDLSWFEQKAVAVLLTLLHLGVRNIRLGPRLPAFLTPEAVGVLVDRFGLIPANVADPAADMQ
MMMECK*

>jgi|Chlre5_6|18305|Cre09.g391450.t1.2
MLRAPVMSSAASRKAAVPAVAARAGCRRVGAMRVFAFQKSTGSVHDKNALHERIQKSKELLDAESAMMCYQCEQTKSGTG
CTDIGVCGKTPEVSALQDLLIYSVKGLGSLAHVARTSPAKIEDAAVNTFINGAIFSTLTNVNFADDRFLEFVTDCRKLHA
QLAAKMAAAGVAVPAAETAHQPWFGSMPHPLAWNSDTHVALAGVGDMLEVASKTGVKERQHVLGETLAGLQELLMYGLKG
LCAYAHHAEALGHTDPAVYADVQAYLHFLCSPAAADVGQVLDACFRAGATNFRVMEMLSNAHTDTFGHPVPTPVTLNPVP
GKAILVTGHDMHDLHMLLEQTAGKGINVYTHGEMLPAHGYPGLKKYPHLVGHFGGAWYRQKIDFAAFPGAVAVTTNCVLD
PLTAYKDNIFTINETGLSGVPHIRPDANGHKDFTPIINRAMQLPGFTAESVAKMEKKRDVTVGFGHKSVLSVAPQVIQAI
QEKRLEHIFLVGGCDGSEPQRKYYSKLYQFMPTNTMVLTLGCGKFRIFDQDFGTLPGTDLPRLLDMGQCNDAYSALVVAT
ELAKVFKTDVNSLPLSLDLSWFEQKAVAVLLTLLHLGVRNIRLGPRLPAFLTPEAVGVLVDRFNLIPANVADPGADMKMM
MECK*

>jgi|Chlre5_6|18620|Cre09.g393543.t1.1
MLSRCLGMAGTTLGGSLASGAQSAVSGMFRASGRRATSLQVLAWQLPNLFAGDQQARNAASIKAKMAEANKALESDKMLC
YQCEQTKSGTGCTEIGVCGKTPEVAGLQDLLVYSVKGLASLAHIARNSPAKIEDPAVNTFINGAIFSTLTNVNFADDRFL
EFVSEARAHHARLSAKMAAAGVQVPASATEQQVWFGSMPHPLLWNSQAAALGGVGDMLEVAAKTGIAERQKVLGETLAGL
QELLVYGLKGVCAYAHHAEALGFTDPTVYAEIQGALHFLNTPGAKDVGQVLDACFKCGATNFKVMEMLSNAHTDTFGHPV
PTPVTLNPVPGKAILVTGHDMHDLHMLLEQTAGKGINVYTHGEMLPAHGYPGLKKYPHLVGHFGGAWYRQKIDFAEFPGA
VAVTTNCVLDPLQVYKQNIFTINETGLSGVPHIRPDANGHKDFTPIINRALQLPGFTPELIEKRPKKKDVTVGFGHKAVL
SVAPQVIQAIQEKRLEHIFLVGGCDGSEPQRKYYSKLYQYMPTNTMVLTLGCGKFRIFDQDFGTLPGTDLPRLLDMGQCN
DSYSALVVATELAKVFKTDVNSLPLSLDLSWFEQKAVAVLLTLLHLGVRNIRLGPRLPAFLTPEAVGVLVDRFNLIPANV
ADPGADMKMMMKNK*

>jgi|Chlre5_6|18619|Cre09.g393506.t1.1
MLARCMSMASTTVRGHLVRGSSGSLSASVARGTGAVRVMAWEWNPFAGDQQARDASIKAKMAEANKALEHDKMLCYQCEQ
TKSGTGCTEIGVCGKTPEVAGLQDLLVYSVKGLASLAHIARNSPAKIEDPAVNTFINGAIFSTLTNVNFADDRFLEFVSE
ARAHHARLSAKMAAAGVQVPASATEQQVWFGSMPHPLLWNSQAAALGGVGDMLEVAAKTGIAERQRVLGETLAGLQELLV
YGLKGVCAYAHHAEALGFTDPTVYAEIQGALHFLNTPGAKDVGQVLDACFKCGATNFKVMEMLSNAHTDTFGHPVPTPVT
LNPVPGKAILVTGHDMHDLHMLLEQTAGKGINVYTHGEMLPAHGYPGLKKYPHLVGHFGGAWYRQKIDFAEFPGAVAVTT
NCVLDPLQVYKQNIFTINETGLSGVPHIRPDANGHKDFTPIINRALQLPGFTPELIEKRPKKKDVTVGFGHKAVLSVAPQ
VIQAIQEKRLEHIFLVGGCDGSEPQRKYYSKLYQYMPTNTMVLTLGCGKFRIFDQDFGTLPGTDLPRLLDMGQCNDSYSA
LVVATELAKVFKTDVNSLPLSLDLSWFEQKAVAVLLTLLHLGVRNIRLGPRLPAFLTPEAVGVLVEKFNLIPANVADPGA
DMKMMMKNK*

>jgi|Cyapar1|5425|g5263.t1
MPPMGLDLSKFALWDEVFHPQSGPRRIVILYGTQTGTSEGFAKAVFASASARNYDVRISCMDRYDCSKLAEEDVIVCLTS
TFYNGEFPDNARSFWAFVNSADRPADSLSKTSFAVFGLGNKKNVENFNSAGKMLDERLAALGSKRLVPIGLGDEYDQNGH

ESAYRPWVKSLWAALGSSEAKRHLPITMNCELSSDKSAPAAAAVPAGFASLKVVSNDLLSATGYDREERMLVLDLAGSPF
ASYELGSQILVQPRNSAQKVARTAAALKLDLDAVVSVSAVAGQHSQSEEMPIAASLTVRDLLSSYLDVSSAVSRTLLEGL
SILAADKKEAEEMEKIATDMLPGNEYGRLMTTESFSLADVLERWRSVSITLSELVSNVPRVGPRHYSVASSPLVKPKSIE
ILYVLDTWATKKGPAFEGLCTRHMASLKAGDTVLAKIARGGIALPSDHGLPLVGVALGSGIGVFRGILEHRASLQAKGKK
VGRVTLFYGCRHRTKDYLFEKDFQEWAKKGLADVVPSFSHDQAEFIHPGMKIAENPHKVADTLDANGVYVYCGIGGSVPG
IVLDSLIAAVRSKKQLSDSQGAELLAKMRKEKRIVEEAYSRSIDAENALGSVQLRKGGHADAADESKPIAESCGDAKMFC
FQCEQTAFGKGCTTVGICGKTPEVAALQDLLVHRVQVMSWYAHQLRKLAEEHKNLTAEETEIPAGNRYTLIGMFSTLTNV
NFSAQRMLEYIAECRDFTTKYVAMYKAACKKAGVKERQCPAPVQTGAEADGDIEDAMVEKGRSVGVLSRFRATKNDALVG
CQEMLVYGIKGVCAYADHAVMNKHESNKIYEFVHHGLSFLLEKESKDLNEVLGMLLKCGEANLVTMALLAQANGTYGEQT
PAVVPVKPVPGKCILISGHELTDVEAILKQTEGTGINVYTHGEVLPAHGYPKLKAYKHLVGHFGQAWQRQSVEFGFFPGA
IVMTTNCITAPREEYKDRIFTAGAVGWPGIPHIANQDFSAAIKRALELPGFSEKDTDFKYPSIASMPRVSQFTTGFGSKL
VISLADKVIGGIKAGAITRFYVIGGCDGFEGERSYFTDFTKQLPETSVVLTVGCGKYRINHLDFKTIGDTGIPRLLDLGQ
CNDSYAAIEIALALAGALNCTVHDLPLSIVLSWFEQKAIAVLLTLLHLGLKNIRVGPNLPAFLRPSVVGVLSDMFGLKLV
SDPTADLTDILGGAPKAGAPCVVNSPAA*

>jgi|Cyapar1|8063|g7806.t1
KNDALVGLQEMLVYGIKGICAYADHALMNKFESAHVYAFVHRGLAFLLEKEARDMGKVLDMLLDCGEANLVTMDILHKAN
TTYGPQTPYVVPVKPVPGKCILVSGHEMSHIEAILKQTEGKGVNVYTHGEMLPAHGYPKLREFKHLVGHFGQAWQRQSVE
FGWFPGAIVMTTNCLTAPREGYLDRIFTSGAVGWPGVPHIDGNDYTAVIKRALELPGFLEADSEFKYPSIAAMPRVPEFT
VGYGSETVIANADKVIDGIKNGAITRFYVIGGCDGFAGERSYYTDFTRMLPETSVVLTAGCGKYRVNHLEYKTIGATGIP
RLLDMGQCNDSYSAIQIAVALAGALNCSIHDLPLSIVLSWFEQKAVAVLLTLLHLGLKNMRVGPTLPAFLRPSVIKVLNE
KFGLKLVGDPVSDLTDILGFKPDPEAPTVATMRAPNP*

>jgi|ChlNC64A_1|29581|estExt_Genewise1.C_20445
MASLRCATRLASLTRVSQSVFARLPGFAGGQAHTAVRASPLLVRACAAAAELAKQPVLDNKMFCYQCEQTSKGTGCTTVG
VCGKTPEMAFLQDLLTYSLKGLGCWADFARQQGVEVPRKVYSLLNAATFATLTNVNFHDARFKEYISTCQSLREGLEKSL
RDKGIDASPPAPVNLPWFDLLAHPAAWKLNQSYLAAASMDQLTQLGKMTSLEHRRHVVDPTLLGLHEMITYGLRGLAAYS
HHAEVLGHRDPEVDEFMARCYAFLCSEDALDLGKTLGMVDELGAVGVKGMQLLDKGHTTKFGHPEPTQVRITPKKGKAIL
ISGHDLQDTHDLLAQTEGTGINVWTHGELLPAHGYPALKKRFPHLVGNYGGAWYRQQKDFAEFPGAVLMTTNCIVEPRQS
YADRIFTTGEVGWAGVRHIEGEMGRTKDYSALISKAQELPGFEYEPPESEAKFVTTGFARNAVLGVAGEVVKAVQEGHLK
HIFLIGGCDGSEPDRKYFGRVADATPQDTMVLTLGCGKFRFYDHDFGMLPNTPLPRLVDMGQCNDAYSAVVVASKLAEVF
GTDINGLPLSLDLSWLEQKAVIILLSLLHLNVKNIRIGPRAPAFLTPEALKVIVDKWNLQIVDTKNPESDVQKMMAGQ*

>jgi|Tetstr1|441518|TSEL_029748.t1
MAMLLRCSALRAVSAAALRHHHRVSTAAALLPPGRLMRLRAFGAVTPPLDSPNHGRCFSASAAAEAHPAEEDKMFCYQCE
QTTKGVGCTTVGVCGKTPETAALQDLLLYRLKGLAGIATHAKATAGIVDETINEFFNAAIFSTLTNVNFDSARFGEYIRT
ANRHISSLKERVAAAGAPPLQAPAVPWFQGIPHPFAWDMQADSAEDLEVLVDLGHQIGIQARRAMINNDTLLGLHELITY
GLKGAAAYHHHAAVMGKRDQDINHALQQYLVFISSPEAADAGAVLGKALELGATNLAIMGKLEEAHTSTFGHPTPTIVSM
TPKPGKAILVSGHDLSDLKALLDQTEGMGIDIYTHGEMLPAHGYPGLSKYPHLKGHYGGAWYRQKIDFDKWPGSILITTN
CVLDPPPASYAANLFTTGETGVEGVQHVAPKEFGAVIERAKQLPGFGPEAADAEERTHLVGFGREAMLGAAPALLDAIKA
GQLEHIFLIGGCDGSEGSRRYYKNVAQKMPETSAILTLGCAKFRLLGHDYGNLPGTELPRLMDVGQCNDSYAAIQIAVAL
AEALGTKDGVNGLPLSLDISWFEQKAVAVLLTLLHLGVKDIRLGPALPAFITPDALKILVDTFGLKPADLKNPEEEPGAH
AHARAMSIPVRSRGCGAITYLTNTFRTAPSNAPSAVLPGQTGSLTMNKA*

>jgi|Mesovir1|5003|Mesvi365S05478
MATRGALRLLRSSARLGAASTPLANPIRASPAVLAILQNGIHKSEVCHTSTNWLRSFRSSALAAGGVIDPSTVVGTELDP
KMFCYQCEQALGQKGCVKIGQCGKTPETAALQDLLIYAVKGLGSWAHYARTKHGIEDDAVNNFVHAAMFATLTNVNFDSQ
RFMEYLTVVESLRNQIIKQVHAKGDNKGPDAPKLPWFDRLPPPAEFSIAEAMGPGVTDETKMVALAKLASVMHRREVINN
DTLVGIHELLTYGLKGMMAYYHHAIRLGQHDASVVAFMQEALAFLCSEDSKDLDKALDFAFRCGENNVKTLALLDTAHDK
VLGTPYPAEVRTTPVAGKCILVSGHDLWDLKTLLEQTEGSGINVYTHGEMLPAHGYPELRKHKHLVGHFGGAWMRQQTDF
VKFPGAILMTTNCIMEPSSFYRKRIFTTGEVGVSNAAHITDGKAKGRPETDFSELIECAKKEPGFRATEENTKPVTTGFG
HRFVLSIVDKVLGAIQSGDLKHIYLIGGCDGSEMDRSYFTEVAEGLPKDSIILTMGCGKFRLNQLNLGTLKQSGLPRLLD
MGQCNDSYSAVVVAQALAKALNTDVNSLPLTLIVSWFEQKAVAVLLSLLHLGIKGVRIGPRLPAFLTPEALALLNQKFDL
KPTNSFDAQTDLKAILAGK*

>jgi|Chabra1|328584|rna-CBR_g19808
MAAATAFSAGSRHAVRGLMRRAISRLSAVSADISSSSSGSSPGMSAGNSLIPPNFKEFLGARTRTPAINVVPMPPQRSTY
RTYRSQAAGTTVTKEKVHEMFCFQCEQTKDGVGCTTVGVCGKDPKTAALQDVLLHSLKGLSQYAVRAKAEGISDPEMDSY
VQEAIFATLTNVNFDPKAIQEFINTTIAHRENIKAKYEAACQRAGRQPDMLDNAFTKFLPSGDVDALAQQGTLLGLDMRR
AGLNEDIFSLQELLTYGIKGMAAYAAHAHAVGEADAKIGGFIHEAFDYISKPTTEQTLEELLALCLKCGEVNMTAMEILN
KGHIHNFGTPEPTKLRTTPVKGKCILVSGHDLKDLYELLKATEGMGINVYTHGEMLPAHGYPEDTLVMTLACGKYRFNKQ
FDSFGTLEGSDLPRLMDIGQCNDAYSAIRIAAALAQAMNTDINSLPLSMVLSWFEQKAVAVLLTLLHLGVQNIRIGPKLP
AFVSPNILNIIVEKYKLTPISGPEFVEDDIRAMLSLSSSSGAAKAAASASPV*

>jgi|Guith1|152637|fgenesh2_kg.34_#_20_#_133_1_CCFI_CCFN_EXTA_EXTB
MAFAATSKVMHPMHLSTTSSLAPMSKLQPPTMAACSKSSLKGLKMCSGQRSMAIANQQTLQSEHYPENMACYQCSQTAHG
KGCFSNSGVCGKQMGTSALQDLIVEINKEIGQLANAGKLTPEMRAFFLESMFSTLTNVNFDDSRMVDYIRKGQVLLAAAK
GESKSVNDFASEPDHWLEVAETYSHAARQKNLDPDAFGLIELATYGIKGACAYMFHAEAVKKQAPDAYPDQEANQVIDGL

LKIMAAMLEPTKHDLLALSLEVGALNVSVMKMLSDSHRALLGIPSPHEVSCKLKPGPCILVSGHDLADLKILLEQTEGKG
VNIYTHGEMLPAHSYPELRKHKHLAGNWGGAWWEQHGDFKAFPGAILMTSNCLTAPTRSYRDRIFTAGPVGWDGIPHVQG
HDFSQLVDCAVNKAKPATGDEVSTHPAEKVTVGFGYETVEGVTDTLLGAIKGGQLKDVFLVGGCDGREAGRSYFTDLVKQ
SPDSSVILTLGCGKFRFNDQELGNLGESGIPRLLDIGQCNDAYSAVVIANGLADKLNVKMSDLPLHFAISWLEQKAVAVL
LSMLHLNMKNIYLGPNLPAFLTPNLVKALVDNYNLKLTGDAEKDLKEMLQ*

>jgi|Phatr2|12416|e_gw1.8.44.1
MFCRQCEQTADHYACTTQGICGKTSETAACQDALIESIKSVSAWCVQARKEGSVSEQDLEAANIWTLQATFSTLTNVNFS
DDRIADYIHQGQAIRAELQKKVLAAPTEFIGQLNLAGKSTAELEDFGHTVSIPKRAAAMGNEDAFSLNEIATYGLKGLCA
YAAHCQALGKMDPEVNAAVHEIFTKLASAEPDVDGLLATVLRVGQVNGTVLAMLDSAHADLLGVPEPTPCRMTAVEGKAI
LVSGHDMVDLYELLKQTEGTGVNVYTHGEMLPAHGYPKLKAFDHLKGNYGTAWQNQKFEFASFPGPIIVTTNCIVEPRRM
YKDRLYSMNEVGVDGVQHIPNRDFTGVIEQAQQMKGFVRTIDPPTFHTVGFNHRVVLPLAEQIIDAAKSGTLSRIFLIGG
CDGSQWDRSYFTDLAEETPDDSLILTLGCAKNRIIHQEKLMDAKLANGLPRVIDMGQCNDSYSAVVVATELAKALDCSVN
DLPLSLCLSHLEQKAAAVLLTLLSMGVKNIRLGPSLPAYVTPNVLSILQSEYNLMGTGDAKKDLAAMMEGN*

>jgi|Nanoce1779_2|599317|fgenesh1_kg.20_#_499_#_TRINITY_DN7124_c0_g1_i6
MMLSSSHRRFLPLGLLSLQQFFSSARPNAAVAVGFVRPLHSSPHPLKCAATNIAPRGAATTAVDAAASANKDMMCFQCEQ
TKHGRSCSPAEGSTQGVCGKTPETAALQDLLIHYTFGVSQYAHSARVHGGLSDVAVDRFLLESLFSTLTNVNFDPDRFVA
FMAEAQEMRDRAKALYEQAVRGKPEANTCLEGPAAMDLRALQGHTDKLETIGHMVGVNYRKDIAANIDAWSLGELLIYGL
KGTAAYADHARILGKESNHVYSFIHEAMATLADLPNQTTENLFGMALRCGEINLEVMELLDAGSTGRYGHPTPSEVRTTP
VKGKCIAVSGHDLRDLEEVLKRTAGKGINVYTHGELLPAFGYPKLRETYPHLVANYGGAWQAQKVEFSRFPGPIVVTTNC
LIEPQKAYKDRIYTRGVVGWKGVKHIKDWDSQFPAVIEQALALPGFPEDTPKNVTMTGYGRNAVLGMAGDIVNAINTGDI
KQIYLIGGCDGAEANRTYFRDIAVGLPRDSIVLTLGCGKYRMNKIPFEPTLGGIPRLLDMGQCNDAYSAIKVATALADVY
KCDVNDLPLQYAISWFEQKAVAVFLTMLSLGLKNIKLGPQLPAFLTPNVLQILSDSYGVRQVNLADHDADLQEMINRNPR
*

>jgi|Vitbras1|20179|Vbra_18549.t1
MAAEIEEFQPMNKMFCYQCEQTSHGMSCTKTGECGKTAEVAMLQDLLIHKLKELSFFVDAFRANKKSIKFADQPPFPSEE
MSDVVEVPFDVHKMNVFLLDSIFATMTNVNFDDDRFIDYLRETEGYIAQVKAAANTHLGLTDQFFAKACGKQYRQLPSRY
ASVLAEGDIFELTDIAGSTGVEQKQYDVDNEDVVGAQELVVYGLKGAAAYAAHAQLIGKEDSSVYSFAYEALAFLTSPDA
LDLSKCLEMALRVGSINYLVMKLLSEANATFGEPEPTEVPVRPKPGKCILVTGHDLSMLHDVLEATKDRGIQVYTHGELM
PAHTYPKLKAYPHLAGHFGGAWQNQRRQFSKFPGAILVTTNCLMPLIHKSPYKDRIFTTGVCGGEDIQHLESTKDLSKLI
QAAEAAPGFTEADTEFNVRDPVQKHMPPSFHVGHSYQWIVKNAQTVLKLIQEGKIKRFYLVGGCDGSEGERSFYTDLVKQ
LPQTSVVMTLGCGKYRINHLEEGMGKIDGTLPRLLDLGQCNDAFGAIQVALTLADALNCGVNDLPLSIVLSWFEQKAVAV
LLTLLFLKVKPLYIGPKLPGFLTKNVLDVLSRDFGLVPLATEHEVEAKMSPPEWGTFQQLVSLAA

>jgi|Symmic1|35194|rna24970
MAKNAEQLALPVKVKCRSTGHLILRGGTLMYNEDMQMVEIEFSKTTQEAYSYKGPFKGEPLALWLGPSTTIMKLSSISSV
KAMDYIEGTRVNCVLIIRLRPEPSDDLMQVAPDAGKEEEVIVQFARVEDRDNWDTGLRYLINALEVTVAKDQVEVPTRSF
SRIKKVRLEEPRAGILVRGRFELASGEEPELEIPESMADGKDLNTFVVDWVQRNCVQPSETTSLYRLVKSLVHRATLESK
TADIIQRINDTHFDKLLKEHPGDPQATLEISKAYLREVGHDIPKLIGQQGTASAMVIQILQRNVEKMKVINDMAYRIHIG
ETDQHVTDFLKIWQGCARFGVEIVLRPQFGFKSEQTNAGKGCTTTGVCKKSPTTAGLQDLCLAKAPGKSFMLCASASWRL
LLVLRTHLRLTNVNFDNARFEAYLKQSAALAEKMEKKLADHSGPYRKDIFGVLEMCVYGLKGVMAYFYHAEHLQARSRLA
GEELVKVNDQHPAVQEDKAAAYDEVERTEVYQACNPKKELLRGLWVEPPQELYRIGAFLCSAGTRTRRIEPQLHENLKVM
KLLDAGHNAVLGTPEPTQVKQEPPKGPAILVSGHDLSILGKLLEQCKGRGVNVYTHGEMLPAHSYPGLKNLGVRL*

>jgi|Symmic1|10117|rna37234
MVVWAVQKPWLKQNLPYRAAHFPAFPGLGGLGGNPWQQQRRPPPTVPADFQVDSNARHFGTCVYYHKWRGYGFLEPTHVG
TVPTNKVFVHWKQLTSDDRFPFLVKGMEVEFSLMVSKDFHRGLNTLRAKNVTLVGGESIALQDELDATEKQFVGGQHLRY
TGTLKFYSPRHGFGYVMMDEGYDVEASVPLELRVDHEEVNAAGQQPVHMRDIAVEFGIYRTDRGQYKVYNMTLQGGHPLT
QDALENRISMGMGMYRGQIAFWNWRQGYGFIRPDPTAILPEKVVARLVEQAEAARKRGKRIAEDSLLYFRRPDVTPMLIP
QQGMQVGFQVYIDDKGAGACDDDSIILTLGCGKFRLNGRDYGTIGGIPRLLDVGQCNDAYGAVVIATKLAEALGCGVHDL
PLHFAVSWFEQKAPAGWGGAKTNII*
